# Selective preparation of tetrasubstituted fluoroalkenes by fluorine-directed oxetane ring-opening reactions

**DOI:** 10.3762/bjoc.16.160

**Published:** 2020-08-07

**Authors:** Clément Q Fontenelle, Thibault Thierry, Romain Laporte, Emmanuel Pfund, Thierry Lequeux

**Affiliations:** 1 Normandie Université, Laboratoire de Chimie Moléculaire et Thioorganique LCMT UMR 6507, ENSICAEN, UNICAEN, CNRS, 6 Bd. du Maréchal Juin, 14050 Caen, France

**Keywords:** acyclonucleotide, fluorine, monofluoroalkene, oxetane, selective ring-opening reaction, tetrasubstituted alkene

## Abstract

The selective ring-opening reaction of fluoroalkylidene-oxetanes was directed by the presence of the fluorine atom, enabling a two-step access to tetrasubstituted fluoroalkenes with excellent geometry control. Despite its small van der Waals radii electronic, rather than steric influences of the fluorine atom governed the ring-opening reaction with bromide ions, even in the presence of bulky substituents.

## Introduction

**Figure 1:**
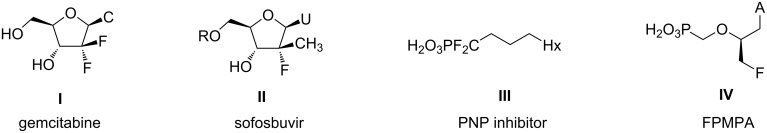
Representative fluorinated nucleos(t)ides and acyclonucleotides.

The introduction of fluorine atoms into organic compounds is known to modify their biological and physiological properties and can enhance the half-life of drugs in vivo [1–4]. During the last decade, fluorinated nucleoside analogues have received increasing interest, as is illustrated by the two pharmaceutical leads gemcitabine (**I**) and sofosbuvir (**II**), potent anticancer or antiviral agents, respectively (Figure 1) [5–6]. The field of acyclonucleotides (ACN) has been explored less, however, the introduction of fluorine atoms showed remarkable effects. The most representative examples are phosphate analogues such as the nucleoside phosphorylase inhibitor **III** and acyclic nucleotides such as the antiviral agent FPMPA (**IV**) [7–9].

**Figure 2:**
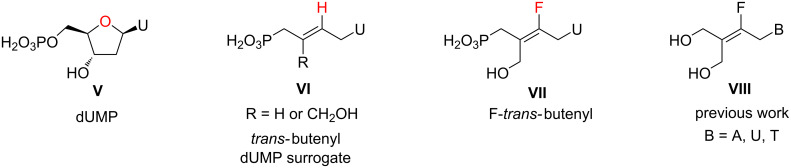
Acyclonucleotides as nucleotide surrogates.

Other main structural modifications of ACN relied on the introduction of a hydroxy group into the aliphatic chain to improve hydrogen bonding with enzymes [10], or of a carbon–carbon double bond to constrain the aliphatic chain and to limit conformational changes [11–13]. For the latter, nucleoside analogues (Figure 2, **VI**) containing a *trans*-butenyl moiety where the endocyclic C–O bond was replaced by a C=C bond are recognized by kinases as dUMP surrogate (**V**) [11]. However, there is no existing data for the corresponding fluoroalkene (**VII**), as the latter was not yet synthetized. It is expected that the introduction of fluorine into the carbon–carbon double bond, in a position equivalent to the ring oxygen of the naturally occurring nucleotide, will improve molecular recognition and activity. In addition, the polarity of the nucleotide and hydrogen-bond accepting capacity with proteins or enzymes would be restored [14].

**Figure 3:**
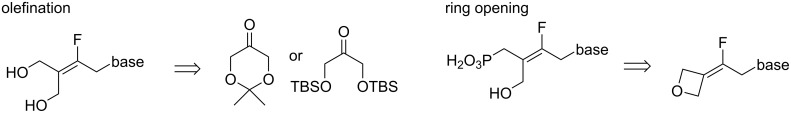
Olefination approaches and ring-opening of oxetane derivatives.

The synthesis of fluoroalkene precursors of modified acyclonucleosides (**VIII**) has been explored by Choi, and more recently by us [15–17]. Nevertheless, it was reported that no antiviral activity for compounds of series **VIII** was observed due to the difficulty of phosphorylation of the substrate by kinases [16]. The first kinase phosphorylation step is generally rate limiting, and the prior introduction of a phosphate or phosphonate function can circumvent this problem. The preparation of diols **VIII** was realized by olefination of a protected 1,3-dihydroxypropanone (Figure 3). However, the selective introduction of functional groups is not possible in these diols as the two hydroxy groups present similar chemical reactivity. Other approaches are available for a selective preparation of monofluoroalkenes including olefination or defluorination reactions or a sigmatropic rearrangement, but these approaches are limited and do not allow the synthesis of tetrasubstituted fluoroalkenes with good control of their geometry [18–21]. In order to develop a selective synthesis for tetrasubstituted fluoroalkenes we envisioned an alternative approach starting from fluoroalkylidene-oxetane derivatives and to the end we have studied the selectivity of the oxetane ring-opening reaction (Figure 3).

## Results and Discussion

**Scheme 1:**
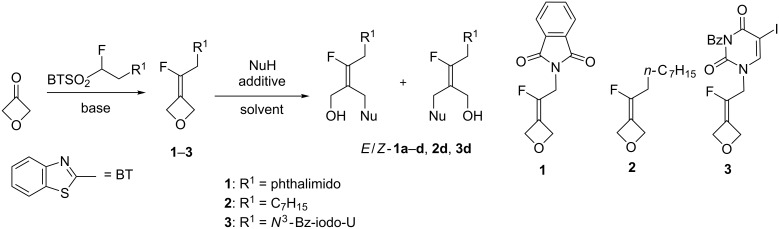
Preparation of fluoroakylidene-oxetanes and their ring-opening reactions.

The preparation of a series of fluoroalkylidene-oxetanes **1–3** was previously reported from 3-oxetanone through an olefination reaction with benzothiazoyl sulfones (Scheme 1) [22]. With these fluoroalkylidene-oxetanes in hands, we studied the selectivity of ring-opening reactions with heteroatom nucleophiles in order to access tetrasubstituted fluoroalkenes. A control of the geometry of these reactions would allow ready access to novel fluorinated ACN precursors.

At the outset the opening of the oxetane ring of **1** by a range of nucleophiles was trialed under acidic conditions. Inspired by Yadav et al. [23], methanol (20 equiv) was used as nucleophile in the presence of camphorsulfonic acid (CSA, 1 equiv) in dichloromethane. The reaction was slow and required heating (50–65 °C) for 26 h to reach a 75% conversion and afforded a mixture of *E*/*Z*-**1a** in 60% yield. However, a low *E*/*Z* selectivity (40:60) was observed (Table 1, entry 1). When using neat benzylic alcohol, completion was achieved after 20 h at 80 °C but again no substantial selectivity could be observed for product **1b** (Table 1, entry 2). Inspired by the work of Müller and Wang [24–25], substitution by the more nucleophilic 2-mercaptobenzothiazol (BTSH, 1.4 equiv) was also possible in the presence of CSA (1 equiv) at 20 °C and an 80% conversion was reached after 24 h. An improved *E*/*Z* ratio of 25:75 was determined for this reaction and compound **1c** was isolated in 65% yield (Table 1, entry 3). In this case the nucleophilic ring-opening reaction appeared to be controlled by steric repulsions between the bulky benzothiazolyl and phthalimidoyl substituents affording preferentially the *Z*-isomer of **1c**. However, heteronucleophiles such as sodium azide, secondary amine and cesium fluoride were unsuccessfully tested. Finally, using the conditions developed by Burkhard and Carreira [26], the opening of the fluoroalkylidene-oxetane ring was investigated with hydrobromic acid (HBr 33 wt % in AcOH, 2.3 equiv) in diethyl ether (Table 1, entry 4). This reaction proved faster and reached completion after 45 min at 20 °C, giving product **1d** with an excellent yield of 94% and an *E*/*Z* selectivity of 89:11. The isomers could be separated and crystals of the major isomer were obtained by recrystallization. The X-ray diffraction analysis clearly showed that the bromine atom was located on the carbon *trans* to the fluorine atom resulting in the major product with the *E*-geometry (see Supporting Information File 1). It should be noted that longer reaction times resulted in slow solvolysis of alcohol **1d** with acetic acid giving mainly the corresponding acetate (not shown). Product **1d** was also obtained in a similar *E*/*Z* ratio (88:12) when the ring-opening reaction was performed in dichloromethane with tetrabutylammonium bromide (TBAB) as the bromide source and boron trifluoride diethyl etherate as an activator (Table 1, entry 5). Nevertheless, the isolated yield of the product decreased to 71%. Next, the reaction performed with HBr/AcOH was extended to alkylidene oxetanes substituted by an alkyl chain, and a pyrimidine base. The presence of the alkyl chain in place of the phthalimido group did not affect the selectivity observed with **1**. The ring opening reaction of the *n*-octyl substituted oxetane **2** resulted in an excellent selectivity of 94:6 towards the *E*-isomer of bromoalkylated product **2d** (Table 1, entry 6). The *E/Z* mixture of **2d** was isolated in moderate yield (53%). In this case the corresponding acetate was observed as a minor product (15%) but with a similar selectivity of 92:8, although it could not be isolated in pure form. Unfortunately, the introduction of a nucleic base such as *N*^3^-benzoyliodouracil instead of the phthalimido group gave a complex mixture of products (Table 1, entry 7). In contrast, the ring-opening reaction was successful when performed in the presence of TBAB and BF_3_·Et_2_O and afforded the *E*-alkene product **3d** with good selectivity (*E*/*Z* ratio > 96:4) and 76% yield (Table 1, entry 8). The geometric assignment of compound **3d** was corroborated by 1D NOESY experiments in which after selective irradiation of the protons α to the nitrogen atom, a response was observed only for the protons α to the bromine atom, thus indicating their spatial proximity.

**Table 1:** Oxetane opening by various nucleophiles.

entry	oxetane	Nu (equiv)	additive (equiv)	*t* (h)	*T* (° C)	solvent	product	*E*/*Z* ratio^a^	Yield (%)^b^

1	**1**	MeOH (20)	CSA (1)	26	40	CH_2_Cl_2_	**1a**	40:60	60
2	**1**	BnOH (24)	CSA (1)	20	80	neat	**1b**	45:55	n.a.
3	**1**	BTSH (1.4)	CSA (1)	24	20	CH_2_Cl_2_	**1c**	25:75	65
4	**1**	HBr (2.3)	AcOH^c^	0.75	20	Et_2_O	**1d**	89:11	94
5	**1**	TBAB (2.5)	BF_3_·Et_2_O (1.1)	2	−20	CH_2_Cl_2_	**1d**	88:12	71
6	**2**	HBr (2.3)	AcOH^c^	0.75	20	CH_2_Cl_2_	**2d**	94:6	53
7	**3**	HBr (2.3)	AcOH^c^	0.75	20	CH_2_Cl_2_	–	–	–
8	**3**	TBAB (2.5)	BF_3_·Et_2_O (1.1)	2	−20	CH_2_Cl_2_	**3d**	96:4	76

^a^Determined by ^19^F NMR of the crude mixture; ^b^yield of isolated product; ^c^HBr 33 wt % in AcOH solution.

In order to elucidate the selectivity control in this ring-opening reaction displayed by bromide ion despite the presence of bulky substituents (phthalimido and alkyl groups), a comparative study was initiated using non-fluorinated alkylidene oxetanes as substrates. Since the oxetane ring was attacked from the side of the bulky phthalimide or alkyl chain, it appeared plausible that the selectivity observed for the reaction did not originate from steric hindrance. Accordingly, the electronic influence of fluorine was explored. Thus, the non-fluorinated analogues bearing a phthalimido group (**4**) and an alkyl chain (**5**) were synthesized, and submitted to the ring-opening reaction [27–28]. Pleasingly, when subjecting compound **4** to the previous reaction conditions, a single isomer of bromoalcohol **4d** formed (determined by ^1^H NMR of the crude) and the product was isolated in an excellent yield of 94% (Table 2, entry 3). The geometry of the product was confirmed to be *E*-**4d** by X-ray diffraction analysis (see Supporting Information File 1). The attack of the bromide ion this time occurred from the side of the alkene hydrogen atom (*cis* attack) and away from the bulky phthalimido group giving solely product *E*-**4d**. Likewise, the reaction of the alkyl-substituted substrate **5** proceeded with excellent *E*-selectivity (as determined by ^1^H NMR of the crude). As in case of the fluorinated analogue, a substantial part of the newly formed alcohol **5d** reacted with acetic acid forming the corresponding acetate **5d’**. Interestingly, it appeared that only the *E*-isomer of **5d** reacted with AcOH giving pure *E*-**5d’** in 44% isolated yield. This left an 81:19 *E*/*Z* mixture of **5d** from which the pure alcohol *E*-**5d** could be isolated in 34% yield (Table 2, entry 4). The overall *E*/*Z* ratio for the ring-opening reaction of **5** was 91:9. 1D NOESY experiments were performed with alcohol **5d** and the outcome indicated that the bromide attack took place away from the bulky alkyl chain, resulting in the observed *E*-selectivity. These outcomes were opposite to those obtained with the fluorinated substrate series, consistent with steric hindrance governing the non-fluorine containing oxetane reactions but electronics influencing and reversing the regioselectivity of the fluoro-oxetane reactions.

**Table 2:** Selectivity in the presence or absence of the fluorine atom.

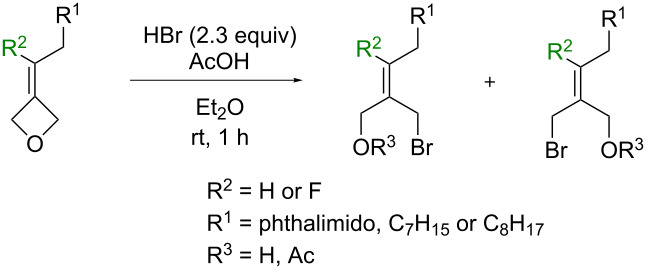							

entry	substrate	R**^2^**	R**^1^**	R**^3^**	product	*E*/*Z* ratio^a^	yield (%)^b^

1	**1**	F	phthalimido	H	**1d**	89:11	94
2	**2**	F	C_7_H_15_	H	**2d**	94:6	53
3	**4**^c^	H	phthalimido	H	**4d**	100:0	94
4	**5**	H	C_8_H_17_	H	**5d**	81:19	34^d^
				Ac	**5d’**	100:0	44

^a^Determined by ^19^F NMR (**1**, **2**) or ^1^H NMR (**4**, **5**) of the crude mixture; ^b^yield of isolated product; ^c^substrate **4** was contaminated with 1 molar equivalent of phthalimide; ^d^yield of isolated *E*-**5d**.

**Scheme 2:**
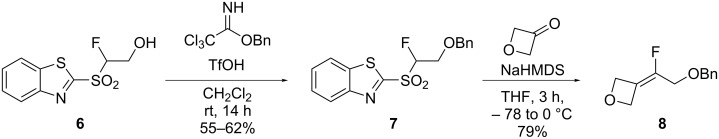
Synthesis of benzyloxy-substituted fluoroethylidene-oxetane derivative **8**.

The reaction was then extended to fluoroalkylidene-oxetane **8** to expand the range of tetrasubstituted fluoroalkenes accessible via this method (Scheme 2). A variety of conditions were explored to prepare the protected alcohol **8** including an unsuccessful reduction of the corresponding ethyl ester (vide infra, **12**). Then we turned our attention to the modified Julia reaction since the reduction of the ester functionality could be achieved at the sulfide stage [29], prior to its oxidation to give **6**. Alcohol **6** was not stable in basic medium, as a Smiles rearrangement occurred leading to fluoroethylene and benzothiazolone. Therefore, its benzylation was explored under acidic conditions with benzyl trichloroacetimidate (1.5 equiv) and a catalytic amount of trifluoromethanesulfonic acid. This gave benzyl ether **7** as a 2.5:1 mixture with *N*-benzylbenzothiazolone (not shown, Scheme 2). After purification, benzyl ether **7** was successfully subjected to the modified Julia olefination conditions with 3-oxetanone, to give the corresponding alkene **8** in 79% yield.

With alkene **8** in hand, the ring-opening reaction was explored in the presence of hydrobromic acid (HBr 33 wt % in AcOH) in diethyl ether (Table 3). As observed with the phthalimido group, the reaction led to alkene *E*-**9** as the major product, together with alkene *Z*-**9** and a third product that was identified as the 2,5-dihydrofuran derivative **10**.

**Table 3:** Opening of the benzyloxy-substituted fluoroethylideneoxetane derivative **8**.

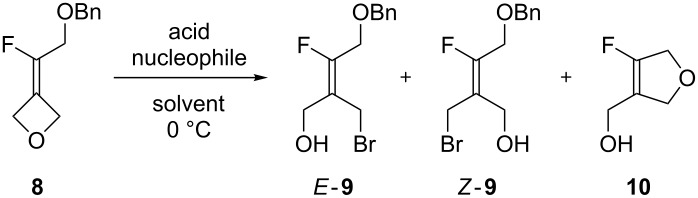								

Entry	Nu (equiv)	Additive (equiv)	Solvent	Reaction time (h)	Selectivity^a^			Yield of **9** (%)

					*E***-9**	*Z***-9**	**10**	

1	HBr (2.3)	AcOH	Et_2_O	0.5	62	8	26	45
2	HBr (1.2) TBAB (2.5)	AcOH	CH_2_Cl_2_	0.5	81	15	4	74^b^
3	TBAB (2.5)	BF_3_·OEt_2_ (1.1)	CH_2_Cl_2_	1	90	10	0	66

^a^Determined by ^19^F NMR of the crude mixture; ^b^yield for the 92:8 *E*/*Z* mixture.

Using the standardized conditions, but at a temperature of 0 °C instead of 20 °C, for 30 min, complete conversion was achieved and the three products *E*,*Z*-**9** and **10** were present in a 62:8:26 ratio as determined by ^19^F NMR, the remaining 4% being attributed to acetylated analogues of **9** (Table 3, entry 1). After purification, two products were obtained as a 95:5 mixture and identified by NMR as the desired bromoalcohols *E-***9** and *Z*-**9**, respectively. The selectivity of the oxetane ring opening (crude *E*/*Z* ratio: 89:11) was again governed by the presence of the fluorine atom and not by steric hindrance.

In order to limit the competitive formation of the heterocyclic ether **10**, the addition of TBAB as a bromide source was explored. To our delight, after 30 min at 0 °C, the crude ^19^F NMR showed that only 4% of **10** and 96% of **9** as an 84:16 *E*/*Z* mixture had formed (Table 3, entry 2). Separation of the two *E*/*Z* isomers proved challenging by column chromatography and a 92:8 mixture of *E*/*Z*-**9** was obtained in 74% yield. Finally, the reaction performed with TBAB in the presence of BF_3_·Et_2_O afforded only alkenes **9** with an excellent *E*-selectivity and in 66% yield (Table 3, entry 3). In this case, we presume **10** was not obtained because the benzyl ether is not nucleophilic enough to react due to it complexation by BF_3_·OEt_2_.

**Scheme 3:**
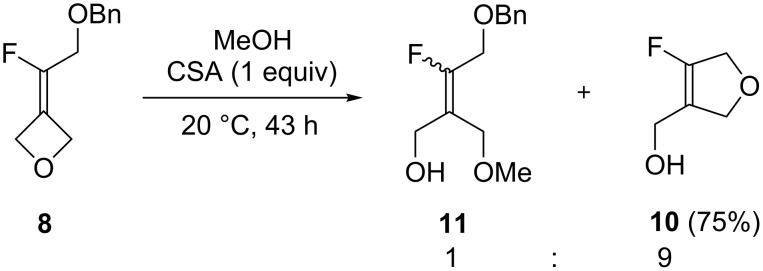
Effect of the medium on the selective formation of derivative **10**.

To understand in more detail the competitive formation of linear versus cyclic products in the reaction, methanol was explored as the nucleophile instead of bromide ion (Scheme 3). The reaction (43 h, 20 °C) realized in MeOH as a solvent and in the presence of CSA (1 equiv) afforded a mixture of **10** (85%) and the expected methoxyalcohols **11** (10%, 1:1 *E*/*Z* ratio) in addition to starting fluoroalkylidene-oxetane **8** (5%). After purification, compound **10** was isolated in 75% yield.

**Scheme 4:**
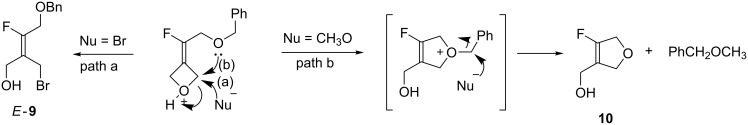
Mechanism for the formation of dihydrofuran **10**.

The results from the acid-catalyzed results support the reaction outcomes which depended on the nucleophile (methanol or bromide ion) (Scheme 4). In the presence of excess bromide ions, the direct intermolecular nucleophilic attack of the oxetane (path a) is preferred leading to bromoalcohol **9** (path a). On the other hand, in the presence of the weaker methanol nucleophile, an intramolecular ring-opening reaction by the benzyl ether oxygen is preferred leading to the 2,5–dihydrofuran **10** (path b).

Finally, we turned our attention to a last series of reactions exploring the ring-opening reaction of fluoroalkylidene-oxetane **12** (Table 4).

**Table 4:** Ring-opening reaction from acetate derivative **12**.

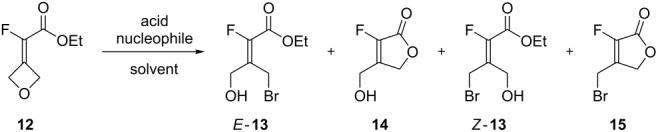										

entry	Nu (equiv)	additive	solvent	*t* (h)	*T* (°C)	selectivity^a^				product (yield)^b^

						*E-***13**	**14**	*Z*-**13**	**15**	

1	HBr (2.5)	AcOH	Et_2_O	3.5	20	–	87	1	8	**14** (58)
2	HBr (1.2)	AcOH	Et_2_O	3.5	0	3	76	7	10	*–*
3	TBAB (2.5)	CSA (2)^c^	CH_2_Cl_2_	30	20	–	7	6	79 (8)^d^	**15** (61)^e^
4	TBAB (2)	BF_3_.OEt_2_ (1.5)	CH_2_Cl_2_	4	–20	–	–	78	22	*Z-***13** (72)^f^ **15** (21)^f^
5	TBAB (1.5)	–	CH_2_Cl_2_	16	20	–	–	–	–	–
6	–	BF_3_·OEt_2_ (1)	CH_2_Cl_2_	2	20	–	100	–	–	**14** (67)

^a^Determined by ^19^F NMR of the crude mixture; ^b^isolated yield; ^c^the 2nd equivalent of CSA was added after 24 h; ^d^chlorinated instead of brominated products; ^e^yield of an 87:8:5 mixture of products **15**/**19**/*Z*-**13**; ^f^*Z*-**13** and **15** were obtained as a crude mixture, yields were calculated from the crude mass and ^1^H NMR.

**Scheme 5:**
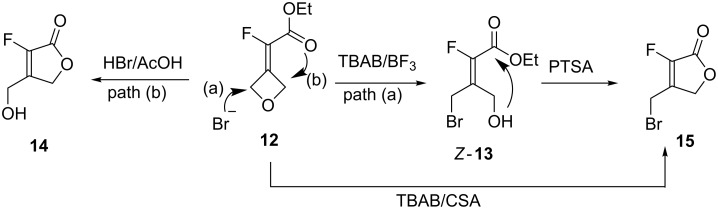
Mechanism for the formation of unsaturated lactones **14** and **15**.

Given the previous results, in the presence of the ester function we expected the ring-opening reaction to proceed with the formation of additional products to alkenes *E*-**13** and *Z*-**13**. In fact, two byproducts formed and were identified as β-hydroxymethyl-α-fluorolactone **14** and β-bromomethyl-α-fluorolactone **15** (Table 4) [30]. This ring expansion has been already reported in the literature from oxetane-containing α,β-unsaturated carbonyl derivatives through a Lewis acid-catalyzed rearrangement [31]. When the previous conditions (HBr/AcOH) were tried (3.5 h, 0 °C to 20 °C), hydroxymethyllactone **14** (87%, Table 4, entry 1) was the main product with traces of the corresponding acetate (4%, not shown), the bromomethyllactone **15** (8%), and alkene *Z*-**13** (1%). Only lactone **14** could be isolated in a pure form (58% yield). The amount of HBr/AcOH had little influence on the selectivity (Table 4, entry 2) of the reaction. However, in the presence of TBAB (2.5 equiv) and CSA (2 equiv) an 84% conversion into mainly brominated lactone **15** (79%) with traces of hydroxylated lactone **14**, and of bromoalkene *Z*-**13** was determined by crude ^19^F NMR. The remaining 8% appeared to be the β-chloromethyllactone **19** (see Scheme 7 below), an analogue of **15** as determined by NMR analysis and supported by HRMS. All three halogenated products were purified and isolated as a mixture (≈61% determined by NMR). The contrasting result observed with HBr/AcOH and TBAB/CSA highlighted the importance of the acidity of the medium on the reaction course of the ring-opening reaction (Scheme 5). The former, using an excess of acid (33% HBr in AcOH solution) favored the direct nucleophilic attack (path b) leading to lactone **14**, whereas the latter in the presence of TBAB/CSA allowed bromide addition on the same side of the fluorine atom (path a) leading to lactone **15** [31]. However, the reaction can be stopped at the alcohol stage when performed in the presence of BF_3_·Et_2_O instead of CSA to afford alkene *Z*-**13** as the major product. The use of boron trifluoride etherate as an activator combined with TBAB afforded exclusively alkene Z-**13** as evidenced by TLC, but after work-up, 22% of the bromomethyllactone **15** was observed (Table 4, entry 4). It appeared obvious that *Z***-13** could cyclize under acidic conditions and during the purification gave β-bromomethyllactone **15**. This was later confirmed when various ester/lactone mixtures obtained after the ring-opening reaction were treated with acid (PTSA) in Et_2_O giving pure lactone **15** or at least enriched mixtures depending on the reaction times (Scheme 5).

**Scheme 6:**
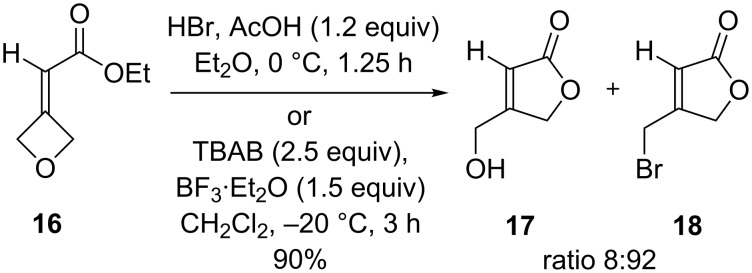
Opening reaction of ethyl 2-(oxetanyl-3-idene)acetate (**16**).

As a control, **12** was shown to be unreactive to TBAB on its own (Table 4, entry 5). Once again, the effect of the fluorine atom was highlighted by an investigation with the non-fluorinated alkylidene oxetane **16**. The latter was subjected to the HBr/AcOH ring-opening reaction conditions (Scheme 6). Remarkably, the cyclic products β-hydroxymethyl and β-bromomethyl-γ-lactones **17** and **18** were obtained in an 8:92 ratio [32]. This complete reversal of selectivity in comparison with fluoroalkylidene-oxetane **12**, where the β-hydroxymethyl-γ-lactone **14** was obtained, confirmed an electronic influence of the fluorine atom on these ring-opening reactions (Table 3, entries 1 and 2). In the case of fluoroalkylidene-oxetane **12** and, in contrast with **16**, when subjected to HBr/AcOH, the electronic repulsion induced between fluorine and bromine limited the intermolecular ring-opening reaction by bromide in favor of a faster intramolecular reaction involving the ester group leading to **14**. Indeed, a competitive cyclization reaction occurred forming **14** with HBr/AcOH and confirmed when the reaction was performed in the presence of BF_3_·Et_2_O only (Table 4, entries 1, 2, and 6). Of note in contrast to **8**, when TBAB/BF_3_·Et_2_O was added we cannot exclude a control by steric or electronic repulsions between bromide ions and the ester function leading to *Z*-**13** and **15** instead of expected alkene *E*-**13** and to the lactone **18** from alkylidene oxetanes **12** and **16**, respectively.

Having established selective approaches for the preparation of halogenated allylic fluoroalkenes, their use in the synthesis of highly functionalized tetrasubstituted fluoroalkenes was explored. The derivatization of the three brominated products, lactone **15** and alkenes *E*-**1d** and *E*-**9**, was studied either on the bromomethyl (CH_2_Br) or on the hydroxymethyl (CH_2_OH) arm, when applicable.

**Scheme 7:**
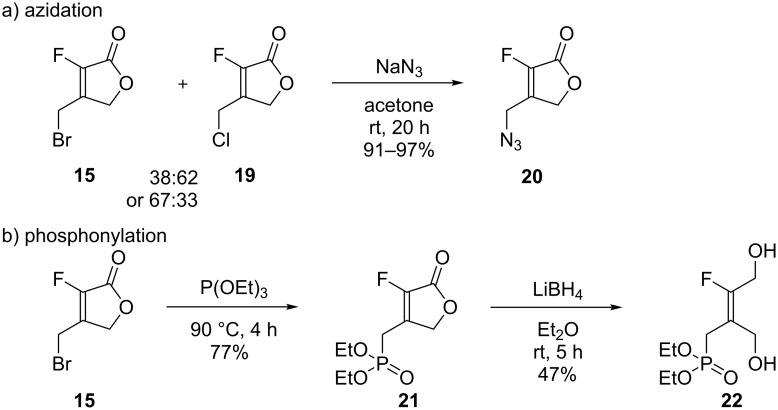
Functionalization of bromomethyllactone **15** and its analogues.

First, from a mixture of lactones **15** and **19** substitution on the bromomethyl arm was performed using sodium azide (Scheme 7). The reaction proceeded smoothly in DMF but it proved difficult to extract product **20** from water. When the reaction was performed in acetone this allowed for a simple filtration of the sodium chloride and bromide salts formed and resulted in very satisfactory yields (91–97%) after column chromatography. An Arbuzov phosphonylation was performed on the crude lactone product **15** (containing 10% of ester *Z*-**13**) and proved successful with phosphonolactone **21** being isolated in 77% yield after column chromatography (Scheme 7). To access tetrasubstituted alkenes, reduction of **21** to generate diol **22** was explored with lithium borohydride in Et_2_O. However, the reaction was slow at 20 °C and did not progress beyond 50% conversion even after the addition of excess LiBH_4_. After purification by flash chromatography starting lactone **21** was obtained in 49% yield and the desired diol **22** in 47% yield. When the reaction was carried out in refluxing THF, a complete conversion was achieved but also with impurities. This route was not investigated further, and instead functionalization of alkene *E*-**1d** was explored.

**Scheme 8:**
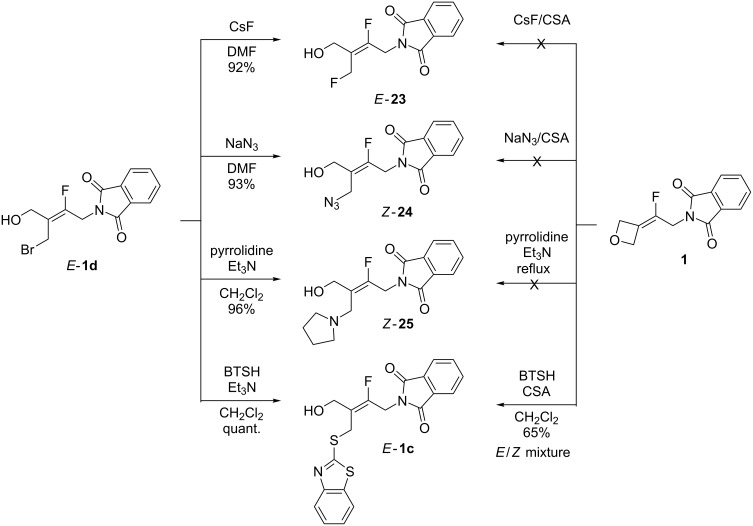
Functionalization by substitution reaction of the bromide *E*-**1d** vs ring-opening reaction of the oxetane **1**.

Direct ring-opening reactions of fluoroalkylidene-oxetane **1** with heteronucleophiles were previously explored and success was only possible with thiols, such as mercaptobenzothiazole (Table 1). However, the reaction was not selective and afforded a mixture of *E*/*Z* alkenes **1c**. The functionalization of alkene *E*-**1d** via displacement of the bromine atom (Scheme 8), with nucleophiles such as CsF and NaN_3_ was then studied. When the reaction was performed in DMF products *E*-**23** and *Z*-**24** were generated in 92% and 93% yield, respectively. Reactions with amines and thiols such as pyrrolidine and 2-mercaptobenzothiazole, gave rise to the products *Z*-**25** and *E*-**1c** in 96% yield, respectively. These reactions were carried out in dichloromethane in the presence of Et_3_N. It should be noted that following this two-step method, pure *E*-**1c** could be obtained while direct ring opening of **1** with BTSH and CSA resulted in a 25:75 mixture of *E*/*Z*-**1c**. A crystallographic analysis of crystals of *Z*-**25** confirmed the nature and geometry of the obtained product (see Supporting Information File 2). Addition of carbanions or alcoholates was also attempted but the starting bromide *E*-**1d** degraded under these conditions.

**Scheme 9:**
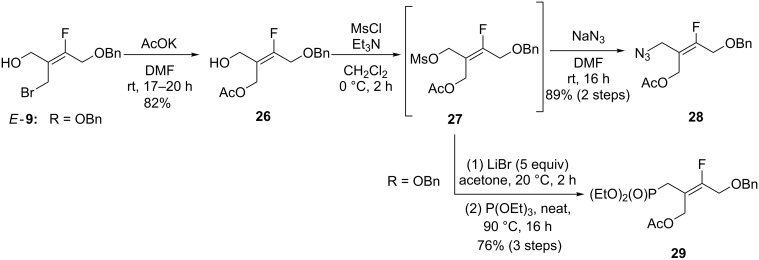
Preparation of tetrasubstituted fluoroalkenes.

Finally, this expeditious synthesis of tetrasubstituted fluoroalkenes by sequential ring-opening and nucleophilic substitution reactions was applied to test the robustness of a selective preparation of precursors of ACN (**VII**) bearing different functional groups (Scheme 9). A particular focus was applied to the preparation of the phosphonate **29**, a precursor of **VII** that is not accessible from diol **VIII**.

First, starting from pure alkene *E-***9**, the introduction of a protected alcohol as a mimic of the naturally occurring 3’-hydroxy group was achieved by allylic bromine displacement with AcOK to efficiently afford alkene **26**. The phosphonate was introduced in three steps through the formation of intermediate mesylate **27**. This mesylate was progressed without purification, albeit contaminated (10%) with the corresponding chloride (not shown). An Arbuzov reaction was performed directly on the allylic bromide obtained by treatment of **27** with LiBr (5 equiv), to give the phosphonate **29** in 76% overall yield. Finally, azide **28** was obtained in 89% yield in two steps from the non-isolated intermediate mesylate **27**. After deacetylation, **28** was readily converted to *E*-**24** (see Scheme 8). These transformations of alkene *E*-**9** illustrated how the geometry can be controlled for the preparation of tetrasubstituted fluoroalkenes. The synthesis of nucleotide mimics from either phosphonate **29** or azide **28** is underway and will be reported in due course.

## Conclusion

The selective synthesis of tetrasubstituted *E*- or *Z-*fluoroalkenes was achieved by ring-opening reactions of fluoroalkylidene-oxetanes, with the presence of the fluorine atom governing regioisomeric attack of the bromide ion. Functionalization of the resultant bromoalcohols with nucleophiles led, in two steps from oxetanes, to a series of highly functionalized tetrasubstituted fluoroalkenes with excellent geometric control. This method offers ready access to novel fluoroalkenes as potential precursors of important drug mimics.

## Supporting Information

The experimental section describing the preparation of all new compounds, the copies of the NMR data (^1^H NMR, ^13^C NMR, ^19^F NMR), HOESY and NOESY experiments and crystallographic data for compounds **1d**, **4d** and **25**. The CIF files of **1d**, **4d** and **25**.

File 1 Experimental section and copies of spectra.

File 2 Crystallographic data (cif) for compounds *E-***1d**, *E-***4d**, and *Z-***25**.
