# Supplementary material for: Selective preparation of tetrasubstituted fluoroalkenes by fluorine-directed oxetane ring-opening reactions
Source: Beilstein J Org Chem. 2020 Aug 7;16:1936–46. doi: 10.3762/bjoc.16.160 (PMC7418098; doi:10.3762/bjoc.16.160)

BEILSTEIN JOURNAL OF ORGANIC CHEMISTRY

Supporting Information

for

Selective preparation of tetrasubstituted fluoroalkenes by fluorine-directed oxetane ring-opening reactions

Clément Q. Fontenelle, Thibault Thierry, Romain Laporte, Emmanuel Pfund and Thierry Lequeux

Beilstein J. Org. Chem. **2020,** *16,* 1936–1946. doi:10.3762/bjoc.16.160

Experimental section and copies of spectra

License and Terms: This is a supporting information file under the terms of the Creative Commons Attribution License (http://creativecommons.org/licenses/by/4.0). Please note that the reuse, redistribution and reproduction in particular requires that the authors and source are credited.
The license is subject to the *Beilstein Journal of Organic Chemistry* terms and conditions: (https://www.beilstein-journals.org/bjoc)

Table of Contents

Table of Contents S1

General Information. S2

Experimental procedure and data for compounds S3

1. Usual work-up procedure........ S3
2. Preparation of fluoroakylidene oxetanes 1-3 .... S3
3. Ring-opening reaction of fluoroakylidene oxetanes 1-3 .... S5
4. Synthesis of oxetane 4-5 .... S8
5. Ring-opening reaction of oxetane 4-5 .... S9
6. Functionalization of compound 1d S10
7. Synthesis of benzyloxy substituted fluoroethylideneoxetane derivative 8. .... S12
8. Ring-opening reaction of the benzyloxy substituted fluoroethylideneoxetane 8 .... S13
9. Synthesis of the acetate derivatives 12 and 16 .... S14
10. Ring–Opening reaction from acetate derivatives 12 and 16 .... S14
11. Opening reaction of ethyl 2-(oxetanyl-3-idene)acetate 16 .... S16
12. Functionalization of bromomethyllactone 15 and its analogues S16
13. Preparation of tetrasubstituted fluoroalkenes S17
14. Structure determination S19

a) X-Ray structures for compound E-1d, E-4d and Z-25 .... S19

b) NOESY and HOESY experiments for compound E-3d, E-5d and E-2d S25

^{1}H, ^{13}C, ^{19}F and ^{31}P NMR for new compounds *S29*

General information.

All commercially available reagents were used as received. For anhydrous conditions, the glassware was flame dried under a continuous nitrogen flow and cooled to 20 °C before running the experiment.

Anhydrous solvents (acetone, CH_2Cl_2, THF, CH_3CN) were dried in a solvent system, which uses an activated alumina column to remove water. Et_3N and MeOH were distilled under CaH_2 or molecular sieves (4 Å). DMF (99.8%, extra dry) was purchased from commercial sources and stored over 4 Å molecular sieves.

Products were detected by thin layer chromatography and the spots were visualized by a 254 nm UV lamp and/or $KMnO_4$ solution. Deuterated NMR solvents were either used as purchased (methanol-d_4) or stored over 4 Å molecular sieves ($CDCl_3$). NMR spectra were recorded with a 400 MHz, 500 MHz, or 600 MHz apparatus in deuterated solvents at 25 °C. All ^{13}C NMR spectra are decoupled from the ^{1}H ($^{13}C\{^{1}H\}$). The chemical shifts (δ) and coupling constants (*J*) are expressed in ppm and Hz, respectively. The following abbreviations were used: s: singlet; d: doublet; t triplet; q: quadruplet; quint: quintet; sext: sextet; sept: septet; m: multiplet; br: broad signal.

Experimental procedure and data for compounds

1. *Usual work-up procedure*

The reaction mixture was cooled to rt, diluted with Et_2O or EtOAc (1 vol.) and quenched with sat. aq. $NaHCO_3$ (1 vol.). Layers were separated and the aqueous layer was extracted with Et_2O (2 × 2 vol). The combined organic layers were dried, filtered, and concentrated.

2. *Preparation of fluoroakylidene oxetanes 1–3*

N-[2-Fluoro-2-(oxetan-3-ylidene)ethyl] phthalimide **(1)**

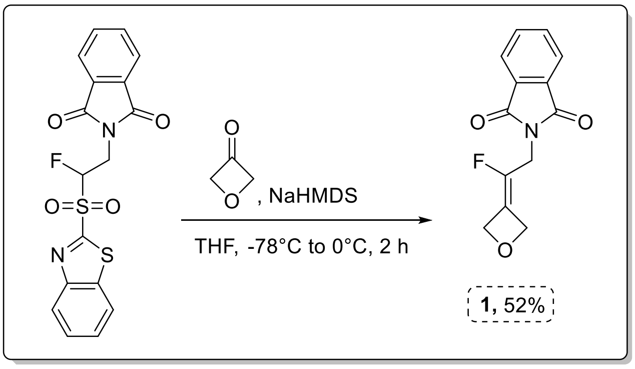

To the sulfone[i] (200 mg, 0.51 mmol, 1 equiv) in THF (5 mL) was added 3-oxetanone (0.035 mL, 0.54 mmol, 1.05 equiv) and the mixture was cooled to −78 °C. Then, NaHMDS (1.0 M in THF, 0.77 mL, 0.77 mmol, 1.5 equiv) was added dropwise. The reaction mixture was slowly allowed to room temperature over 2 h after which the reaction was quenched by the addition of NH_4Cl (sat. aq., 10 mL). The mixture was extracted with Et_2O (3 × 20 mL) and the combined organic layers were washed with brine (20 mL), then dried, filtered, and concentrated. Column chromatography (CH_2Cl_2/EtOAc 99:1 then 98:2) gave 66 mg of **1** (0.27 mmol, 52%) as white solid.

^1H NMR ($CDCl_3$, 400 MHz) δ 7.87–7.72 (m, 4H), 5.29–5.28 (m, 2H), 5.19–5.18 (m, 2H), 4.23 (d, $^3J_{HF}$ = 16.4 Hz, 2H).^{13}C NMR ($CDCl_3$, 100 MHz) δ 167.2 (2C), 143.5 (d, $^1J_{CF}$ = 251.4 Hz), 134.3 (2C), 131.7 (2C), 123.5 (2C), 116.2 (d, $^2J_{CF}$ = 21.7 Hz), 75.1 (d, $^3J_{CF}$ = 6.9 Hz), 74.5 (d, $^3J_{CF}$ = 11.1 Hz), 35.2 (d, $^2J_{CF}$ = 31.8 Hz).^{19}F NMR ($CDCl_3$, 376 MHz) δ –111.2 (tquint, $^3J_{FH}$ = 16.4 Hz, $^4J_{FH}$ = 3.6 Hz). HRMS (ESI) m/z $[M + H]^+$ calcd for $C_{13}H_{11}FNO_3^+$ 248.0723, found 248.0720.

3-(1-Fluorononylidene)oxetane **(2)**

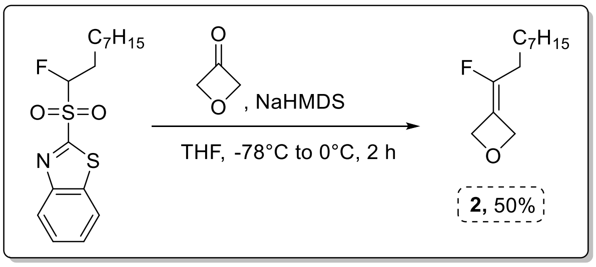

To the sulfone[ii] (400 mg, 1.17 mmol, 1 equiv) in THF (12 mL) was added 3-oxetanone (0.082 mL, 1.28 mmol, 1.1 equiv), and the mixture was cooled to −78 °C. Then, NaHMDS (1.0 M in THF, 1.75 mL, 1.75 mmol, 1.5 equiv) was added dropwise. The reaction mixture was slowly allowed to room temperature over 2 h after which the reaction was quenched by the addition of NH_4Cl (sat. aq., 10 mL). The mixture was extracted with Et_2O (3 × 20 mL) and the combined organic layers were washed with brine (20 mL), then dried, filtered, and concentrated. Column chromatography (pentane/Et_2O 100:0 then 98:2) gave 116 mg of **2** (0.579 mmol, 50%) as colorless oil.

^1H NMR (500 MHz, $CDCl_3$) δ 5.22 (br s, 2H, CH_2O), 5.17 (br s, 2H, CH_2O), 2.04 (dt, $^3J_{HF}$ = 19.4 Hz, $^3J_{HH}$ = 7.3 Hz, 2H, CH_2CF), 1.52 – 1.42 (m, 2H), 1.36 – 1.21 (m, 10H), 0.89 (t, $^3J_{HH}$ = 6.4 Hz, 3H, CH_3). ^{13}C NMR (126 MHz, $CDCl_3$) δ 150.4 (d, $^1J_{CF}$ = 248.8 Hz), 110.1 (d, $^2J_{CF}$ = 25.7 Hz), 75.8 (d, $^3J_{CF}$ = 8.0 Hz), 75.0 (d, $^3J_{CF}$ = 13.0

[i] Calata, C.; Pfund, E.; Lequeux, T. *J. Org. Chem.* **2009**, *74* (24), 9399–9405. doi:10.1021/jo901540c
[ii] Habib, S.; Larnaud, F.; Pfund, E.; Mena Barragán, T.; Lequeux, T.; Ortiz Mellet, C.; Goekjian, P. G.; Gueyrard, D. *Org. Biomol. Chem.* **2014**, *12* (4), 690–699. doi:10.1039/C3OB41926F

Hz), 31.8, 29.2, 29.2, 29.1 (d, $^2J_{CF}$ = 26.6 Hz), 29.0, 25.4, 22.6, 14.1. ^{19}F NMR (471 MHz, $CDCl_3$) δ -116.9 (br t, $^3J_{FH}$ = 19.4 Hz). HRMS (ASAP$^+$) *m/z* calcd for [M+H]$^+$ $C_{12}H_{22}OF$ 201.1655, found 201.1653.

3-Benzoyl-1-[2-fluoro-2-(oxetan-3-ylidene)ethyl]-5-iodo-pyrimidine-2,4-dione **(3)**

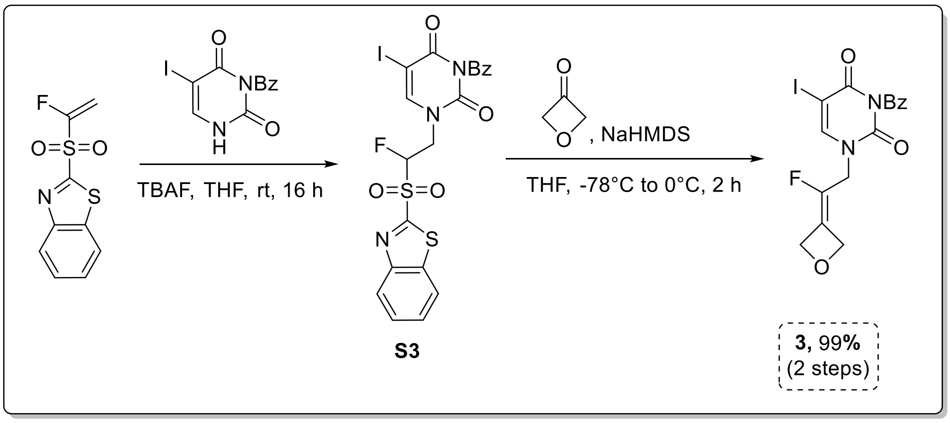

Tetrabutylammonium fluoride (1.0 M in THF, 0.34 mL, 0.34 mmol, 0.2 equiv), was added dropwise to a solution of sulfone[i] (414 mg ,1.7 mmol) and *N*^{3}Bz-5-iodouracil (698 mg, 2.04 mmol, 1.2 equiv) in THF (8 mL) at 0 °C. The mixture was stirred overnight at 20 °C and quenched with a saturated NH_4Cl solution. The aqueous layer was extracted twice with CH_2Cl_2. The combined organic layers were washed with brine, dried with $MgSO_4$, filtered, and evaporated under reduced pressure to give crude sulfone **S3** which was used without purification. NMR data ^{1}H NMR ($CDCl_3$, 400 MHz) δ 8.46 (s, 1H), 8.43-8.41 (m, 1H), 8.36-8.34 (m, 1H), 8.02 (d, $^3J_{HH}$ = 7.6 Hz, 2H), 7.83-7.74 (m, 3H, H-1), 7.62-7.58 (m, 2H), 6.55 (ddd, $^2J_{HF}$ = 46.4 Hz, $^3J_{HH}$ = 6.4 Hz, $^3J_{HH}$ = 1.6 Hz, 1H), 4.75 (ddd, $^3J_{HF}$ = 31.2 Hz, $^2J_{HH}$ = 15.2 Hz, $^3J_{HH}$ = 1.6 Hz, 1H), 4.54 (ddd, $^3J_{HF}$ = 16.2 Hz, $^2J_{HH}$ = 15.2 Hz, $^3J_{HH}$ = 6.4 Hz, 1H).^{19}F NMR ($CDCl_3$, 376 Hz) δ -182.8 (ddd, $^2J_{HF}$ = 46.4 Hz, $^3J_{HF}$ = 31.2 Hz, $^3J_{HH}$ = 16.2 Hz).

To the crude sulfone **S3** obtained (1.0 g, 1.7 mmol, 1 equiv) in THF (50 mL) was added 3-oxetanone (150 µL, 2.5 mmol, 1.5 equiv), and the mixture was cooled to −78 °C. Then, NaHMDS (1 M in THF, 2.5 mL, 2.5 mmol, 1.5 equiv) was added dropwise. The reaction mixture was slowly allowed to room temperature over 2 h after which the reaction was quenched by the addition of NH_4Cl (sat. aq.). The mixture was extracted with Et_2O and the combined organic layers were washed with brine, then dried, filtered, and concentrated. The purification by flash chromatography (pentane/EtOAc, 6:4 to 4:6) afforded compound **3** (747 mg, 99%) as white solid.

^{1}H NMR ($CDCl_3$, 400 MHz) δ 7.92-7.90 (m, 2H), 7.76 (s, 1H), 7.69-7.66 (m, 1H), 7.53-7.50 (m, 2H), 5.23-5.22 (m, 4H), 4.29 (d, $^3J_{HF}$ = 18.8 Hz, 2H). ^{19}F NMR ($CDCl_3$, 376 MHz) δ -124.5 (tt, $^3J_{FH}$ = 18.8 Hz, $^4J_{FH}$ = 4.1 Hz). HRMS-ESI (m/z) [M+H]$^+$ calculated for $C_{16}H_{13}N_2O_4FI^+$ 442.9904, found 442.9919.

[i] Calata, C.; Pfund, E.; Lequeux, T. *J. Org. Chem.* **2009**, *74* (24), 9399–9405. doi:10.1021/jo901540c

3. Ring-opening reaction of fluoroakylidene oxetanes 1–3

N-[2-Fluoro-3-(hydroxymethyl)-4-methoxy-but-2-enyl] phthalimide **(1a)**

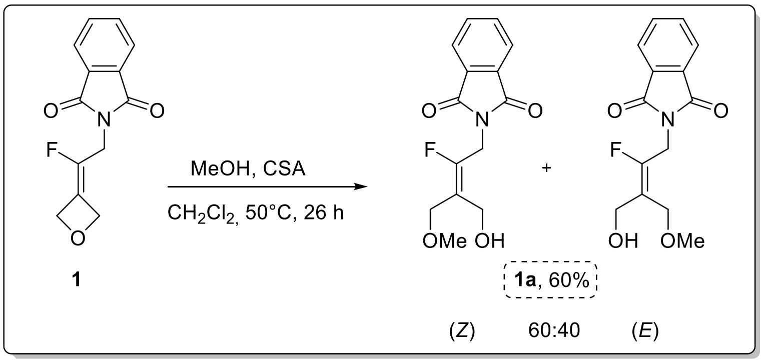

To the oxetane **1** (60 mg, 0.24 mmol, 1 equiv) in CH_2Cl_2/MeOH 1:1 (1.2 mL) at 0 °C was added CSA (56 mg, 0.24 mmol, 1 equiv) and the mixture was stirred at rt. After 2 h, TLC showed no conversion so CH_2Cl_2/MeOH 1:1 (1.2 mL) was added and the mixture was heated at reflux for 6 h. Then usual work up procedure was followed using Et_2O. NMR analysis of the crude mixture showed a 75% conversion into the desired products **1a** with a 60:40 ratio. Column chromatography (pentane/acetone 80:20) gave 41 mg (0.147 mmol, 60%) of a *Z*:*E* (60:40) mixture of **1a** as white solid.

^{1}H NMR (500 MHz, $CDCl_3$) δ 7.89 – 7.82 (m, 4H, H_{Ar}, *Z*+*E*), 7.78 – 7.70 (m, 4H, H_{Ar}, *Z*+*E*), 4.61 (d, $^3J_{HF}$ = 21.4 Hz, 2H, CH_2N, *Z*), 4.53 (d, $^3J_{HF}$ = 20.4 Hz, 2H, CH_2N, *E*), 4.40 (br s, 2H, CH_2OH, *Z*), 4.32 (d, $^4J_{HF}$ = 1.9 Hz, 2H, CH_2OMe, *E*), 4.30 (br d, $^4J_{HF}$ = 2.4 Hz, 2H, CH_2OH, *E*), 4.13 (d, $^4J_{HF}$ = 3.2 Hz, 2H, CH_2OMe, *Z*), 3.43 (s, 3H, OMe, *E*), 3.32 (s, 3H, OMe, *Z*), 3.10 (br s, 1H, OH, *Z*), 2.29 (br s, 1H, OH, *E*). ^{13}C NMR (126 MHz, $CDCl_3$) δ 167.7 (2C, *E*), 167.4 (2C, *Z*), 153.0 (d, $^1J_{CF}$ = 258.6 Hz, *Z*), 152.7 (d, $^1J_{CF}$ = 258.4 Hz, *E*), 134.3 (2C, *E*), 134.2 (2C, *Z*), 131.8 (2C, *Z*), 131.7 (2C, *E*), 123.6 (2C, *E*), 123.5 (2C, *Z*), 118.5 (d, $^2J_{CF}$ = 9.1 Hz, *Z*), 117.8 (d, $^2J_{CF}$ = 12.1 Hz, *E*), 68.9 (d, $^3J_{CF}$ = 8.1 Hz, *E*), 66.2 (d, $^3J_{CF}$ = 8.3 Hz, *Z*), 58.9 (d, $^3J_{CF}$ = 7.6 Hz, *Z*), 58.4 (*E*), 58.2 (*Z*), 57.6 (d, $^3J_{CF}$ = 9.5 Hz, *E*), 35.1 (d, $^2J_{CF}$ = 30.1 Hz, *Z*), 35.0 (d, $^2J_{CF}$ = 30.2 Hz, *E*). ^{19}F NMR (471 MHz, $CDCl_3$) δ -111.9 (br t, $^3J_{FH}$ = 21.4 Hz, *Z*), -110.1 (br t, $^3J_{FH}$ = 20.3 Hz, *E*).

N-[3-(Benzyloxymethyl)-2-fluoro-4-hydroxy-but-2-enyl] phthalimide **(1b)**

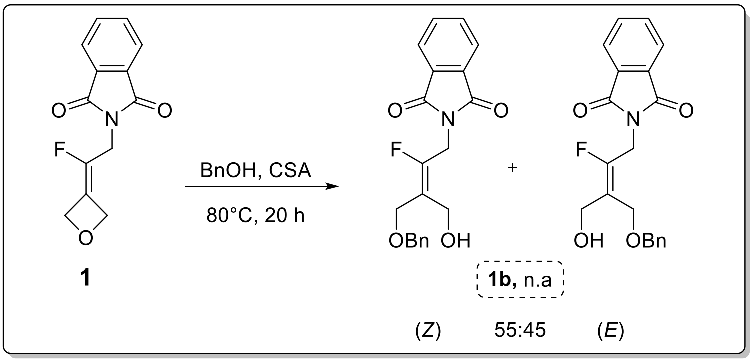

To the oxetane **1** (50 mg, 0.20 mmol, 1 equiv) in benzylic alcohol (0.50 mL, 4.9 mmol, 24 equiv) at 0 °C was added CSA (47 mg, 0.20 mmol, 1 equiv) and the mixture was stirred at rt. After 2.5 h, TLC showed no conversion so the mixture was heated at 80 °C for 20 h. Then, usual work up procedure was followed using Et_2O. NMR analysis of the crude mixture showed a complete conversion into the products **1b** with a 55:45 *Z*/*E* ratio. Column chromatography (pentane/EtOAc 80:20 to 60:40) gave two analytical samples of different ratios: 1st fraction: *Z*:*E* (85:15) and 2nd fraction: *Z*:*E* (6:94).

^{1}H NMR (500 MHz, $CDCl_3$) δ 7.91 – 7.84 (m, 4H, H_{Phtha}, *Z*+*E*), 7.78 – 7.71 (m, 4H, H_{Phtha}, *Z*+*E*), 7.42 – 7.25 (m, 10H, H_{Ph}, *Z*+*E*), 4.62 (d, $^3J_{HF}$ = 21.5, 2H, CH_2N, *Z*), 4.63 (s, 2H, CH_2Ph, *E*), 4.51 (d, $^3J_{HF}$ = 20.3, 2H, CH_2N, *E*), 4.51 (s, 2H, CH_2Ph, Z), 4.44 (dd, $^3J_{HH}$ = 6.7, $^4J_{HF}$ = 1.7, 2H, CH_2OH, *Z*), 4.43 (d, $^4J_{HF}$ = 1.8, 2H, CH_2OBn, *E*), 4.34 (dd, $^3J_{HH}$ = 6.3, $^4J_{HF}$ = 3.0, 2H, CH_2OH, *E*), 4.26 (d, $^4J_{HF}$ = 3.1, 2H, CH_2OBn, *Z*), 3.05 (t, $^3J_{HH}$ = 6.7, 1H, OH, *Z*), 2.10 (t, $^3J_{HH}$ = 6.3, 1H, OH, *E*).^{13}C NMR (126 MHz, $CDCl_3$) δ 167.8 (2C, *Z*), 167.4 (2C, *E*), 153.0 (d, $^1J_{CF}$ = 258.7 Hz, *Z*), 152.8 (d, $^1J_{CF}$ = 258.6 Hz, *E*), 137.8 (*Z*), 137.6 (*E*), 134.4 (2C, *Z*), 134.2 (2C, *E*), 131.8 (2C, *E*), 131.7 (2C, *Z*), 128.5 (2C, *E*), 128.4 (2C, *Z*), 127.94 (*E*), 127.88 (2C, *E*), 127.82 (2C, *Z*), 127.77 (*Z*), 123.7 (2C,

Z), 123.5 (2C, *E*), 118.8 (d, $^2J_{CF}$ = 9.0 Hz, *Z*), 117.9 (d, $^2J_{CF}$ = 12.4 Hz, *E*), 72.8 (*E*), 72.7 (*Z*), 66.7 (d, $^3J_{CF}$ = 8.3 Hz, *E*), 64.2 (d, $^3J_{CF}$ = 8.5 Hz, *Z*), 59.1 (d, $^3J_{CF}$ = 7.5 Hz, *Z*), 57.8 (d, $^3J_{CF}$ = 9.4 Hz, *E*), 35.1 (d, $^2J_{CF}$ = 30.0 Hz, *E*), 35.1 (d, $^2J_{CF}$ = 30.2 Hz, *Z*).^{19}F NMR (471 MHz, $CDCl_3$) δ -111.6 (br t, $^3J_{FH}$ = 21.5 Hz, *Z*), -109.9 (br t, $^3J_{FH}$ = 20.3 Hz, *E*).

N-[(*Z*)-3-(1,3-Benzothiazol-2-ylsulfanylmethyl)-2-fluoro-4-hydroxy-but-2-enyl] phthalimide **(*Z*-1c)**

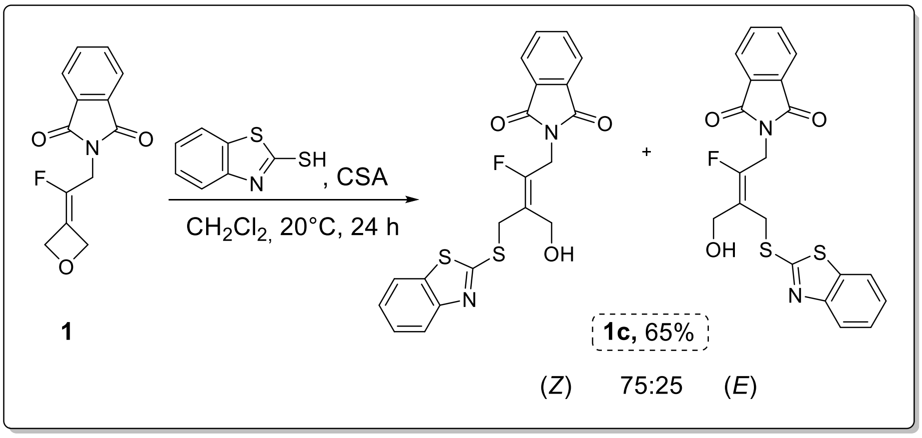

To the oxetane **1** (50.0 mg, 0.202 mmol, 1 equiv) in CH_2Cl_2 (2 mL) at rt were added 2-mercaptobenzothiazole (47.5 mg, 0.234 mmol, 1.4 equiv) and CSA (47 mg, 0.202 mmol, 1 equiv). The mixture was stirred at rt for 23.5 h, then usual work up procedure was followed using Et_2O. NMR analysis of the crude mixture showed an 80% conversion into the desired products *E*-**1c** and *Z*-**1c** with a 25:75 ratio. Column chromatography (pentane/acetone 84:16 then 80:20) gave 55 mg (0.132 mmol, 65%) of a 25:75 mixture of *E*-**1c** and *Z*-**1c** (contaminated with 3.5% of 1) as a white solid. An additional purification gave a pure analytical sample of *Z*-**1c** for characterization. The compound *E*-**1c** was obtained and described page **S12**.

***Z*-1c**: ^{1}H NMR ($CDCl_3$, 400 MHz) δ 7.91-7.82 (m, 3H), 7.78-7.71 (m, 3H), 7.46-7.38 (m, 1H), 7.34-7.29 (m, 1H), 4.69 (s, 1H), 4.58 (d, $^3J_{HF}$ = 21.6 Hz, 2H), 4.50 (d, $^4J_{HF}$ = 1.5 Hz, 2H), 4.24 (d, $^4J_{HF}$ = 2.0 Hz, 2H). ^{13}C NMR ($CDCl_3$, 100 MHz) δ 167.9, 167.6 (2C), 152.4, 152.3 (d, $^1J_{CF}$ = 259.8 Hz), 135.3, 134.3 (2C), 131.8 (2C), 126.3, 124.7, 123.7 (2C), 121.3, 121.1, 118.6 (d, $^2J_{CF}$ = 8.4 Hz), 58.6 (d, $^3J_{CF}$ = 6.6 Hz), 35.2 (d, $^2J_{CF}$ = 30.2 Hz), 28.7 (d, $^3J_{CF}$ = 8.6 Hz). ^{19}F NMR ($CDCl_3$, 376 Hz) δ -108.9 (t, $^3J_{HF}$ = 21.6 Hz). HRMS (ES+) *m/z* $[M + H]^+$ calculated for $C_{20}H_{16}N_2O_3FS_2^+$ 415.0586, found 415.0591.

N-[3-(Bromomethyl)-2-fluoro-4-hydroxy-but-2-enyl]phthalimide **(1d)**

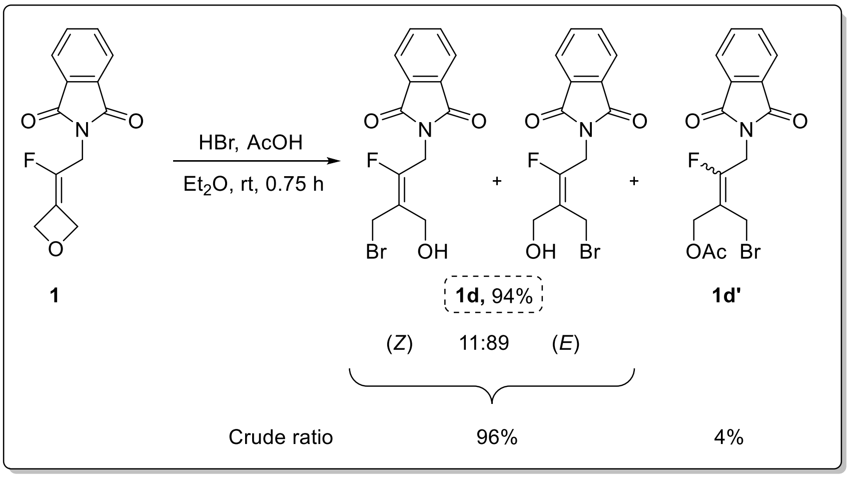

To the oxetane **1** (330 mg, 1.34 mmol, 1 equiv) in Et_2O (13 mL) at 0 °C was added a solution of HBr (33 wt % in AcOH, 0.547 mL, 3.33 mmol, 2.3 equiv) diluted in Et_2O (2.2 mL). The mixture was stirred at rt for 0.75 h, then usual work-up procedure was followed with EtOAc (1 vol. = 25 mL). NMR analysis of the crude mixture showed a complete conversion into the products *E*-**1d** and *Z*-**1d** (*E*/*Z* ratio 89:11) together with 4% of *E*/*Z*-**1d'**. Column chromatography (CH_2Cl_2/EtOAc 98:2 to 96:4) gave three fractions of *E*-**1d** and *Z*-**1d**: 17 mg (0.052 mmol, 4%, 5:95, *E*/*Z*), 110 mg

(0.335 mmol, 25%, 80:20, *E*/*Z*) and 286 mg (0.872 mmol, 65%, 98:2, *E*/*Z*) all as white solids. Further crystallization gave an analytical *E*-**1d** sample for X-ray analysis page **S19**.

^{1}H NMR ($CDCl_3$, 400 MHz) δ 7.89-7.85 (m, 4H, *E*+*Z*), 7.77-7.73 (m, 4H, *E*+*Z*), 4.59 (d, $^{3}J_{HF}$ = 21.7 Hz, 2H, *Z*), 4.55 (d, $^{3}J_{HF}$ = 20.4 Hz, 2H, *E*), 4.44 (d, $^{4}J_{HF}$ = 2.0 Hz, 2H, *Z*), 4.41 (d, $^{4}J_{HF}$ = 1.5 Hz, 2H, *E*), 4.37 (d, $^{4}J_{HF}$ =3.1 Hz, 4H, *E*+*Z*), 1.78 (s, 2H, *E*+*Z*).^{13}C NMR ($CDCl_3$, 100 MHz) δ 167.8 (2C, *Z*), 167.4 (2C, *E*), 153.3 (d, $^{1}J_{CF}$ = 262.5 Hz, 2C, *E*+*Z*), 134.6 (2C, *Z*), 134.4 (2C, *E*), 131.8 (2C, *E*), 131.6 (2C, *Z*), 123.8 (2C, *Z*), 123.7 (2C, *E*), 119.2 (d, $^{2}J_{CF}$ = 15.6 Hz, 2C, *E*+*Z*), 59.1 (d, $^{3}J_{CF}$ = 6.6 Hz, *Z*), 56.6 (d, $^{3}J_{CF}$ = 8.5 Hz, *E*), 35.2 (d, $^{2}J_{CF}$ = 30.4 Hz, 2C, *E*+*Z*), 27.5 (d, $^{3}J_{CF}$ = 9.6 Hz, *E*), 25.5 (d, $^{3}J_{CF}$ = 10.1 Hz, *Z*).^{19}F NMR ($CDCl_3$, 376 MHz) δ -108.0 (t, $^{3}J_{HF}$ = 21.7 Hz, *Z*), -106.5 (t, $^{3}J_{HF}$ = 20.4 Hz, *E*). HRMS (ESI) m/z $[M + Na]^+$ calculated for $C_{13}H_{11}NO_3FNaBr^+$ 349.9804, found 349.9818.

2-(Bromomethyl)-3-fluoro-undec-2-en-1-ol **(2d)**

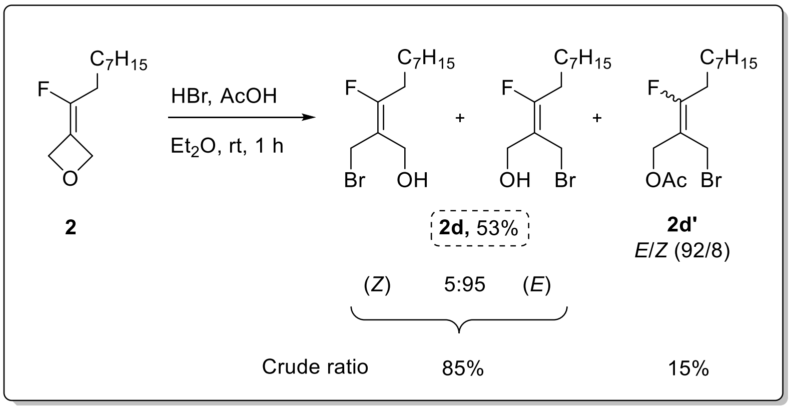

To the oxetane **2** (100 mg, 0.499 mmol, 1 equiv) in Et_2O (5 mL) at 0 °C was added a solution of HBr (33 wt % in AcOH, 0.204 mL, 1.15 mmol, 2.3 equiv) diluted in Et_2O (1 mL). The mixture was stirred at rt for 1 h, then usual work-up procedure was followed (1 vol. = 10 mL). NMR analysis of the crude mixture showed a complete conversion into the products *E*-**2d** and *Z*-**2d** (*E*/*Z* ratio 94:6) together with 15% of acetate *E*/*Z*-**2d'** (*E*/*Z* ratio 92:8). Column chromatography (pentane/Et_2O 98:2 then 80:20) gave 16 mg of impure **2d'** (*E*/*Z* ratio 95:5) and 73 mg (0.26 mmol, 53%, *E*/*Z* ratio 95:5) of **2d** as a colorless oil. The product is not stable and degrades at rt overnight.

^{1}H NMR (500 MHz, $CDCl_3$) δ 4.37 (br s, 2H, CH_2O, *E*), 4.26 (br s, 2H, CH_2O, *Z*), 4.23 (br s, 2H, CH_2Br, *Z*), 4.16 (br s, 2H, CH_2Br, *E*), 2.34 (dt, $^{3}J_{HF}$ = 24.1, $^{3}J_{HH}$ = 7.5 Hz, 4H, CH_2CF, *E*+*Z*), 1.63 – 1.48 (m, 6H, *E*+*Z*), 1.42 – 1.21 (m, 20H, *E*+*Z*), 0.89 (t, $^{3}J_{HH}$ = 6.4 Hz, 6H, CH_3, *E*+*Z*).^{13}C NMR (126 MHz, $CDCl_3$) only *E*-2d visible on spectra: δ 161.7 (d, $^{1}J_{CF}$ = 261.8 Hz), 114.5 (d, $^{2}J_{CF}$ = 18.1 Hz), 56.7 (d, $^{3}J_{CF}$ = 9.5 Hz), 31.8, 29.9 (d, $^{3}J_{CF}$ = 10.9 Hz), 29.2, 29.07, 29.05, 28.9 (d, $^{2}J_{CF}$ = 27.4 Hz), 26.0, 22.6, 14.0. ^{19}F NMR (471 MHz, $CDCl_3$) *Z*-2d: δ -99.4 (t, $^{3}J_{FH}$ = 24.1 Hz, *E*), -97.6 (t, $^{3}J_{FH}$ = 24.2 Hz, *Z*). HRMS (ASAP+) *m/z* calcd for $[M-H]^+$ $C_{12}H_{21}OF^{79}Br$ 279.0760, found 279.0773, $[M-F^-]^+$ $C_{12}H_{22}O^{79}Br$ 261.0854, found 261.0859.

(2-(Bromomethyl)-3-fluoro-undec-2-enyl) acetate **(2d')**

E/*Z* (92/8) ^{1}H NMR (500 MHz, $CDCl_3$) only *E*-2d' visible on spectra: δ 4.79 (br s, 2H, CH_2O), 4.07 (br s, 2H, CH_2Br), 2.35 (dt, $^{3}J_{HF}$ = 23.7, $^{3}J_{HH}$ = 7.4 Hz, 2H, CH_2CF), 2.09 (s, 3H, CH_3), 1.68 – 1.48 (m, 2H), 1.42 – 1.17 (m, 10H), 0.89 (t, $^{3}J_{HH}$ = 6.5 Hz, 3H, CH_3). ^{19}F NMR (471 MHz, $CDCl_3$) *Z*-2d': δ -95.1 (t, J = 23.9 Hz), *E*-2d': δ -95.9 (t, J = 23.8 Hz).

3-Benzoyl-1-[3-(bromomethyl)-2-fluoro-4-hydroxy-but-2-enyl]-5-iodo-pyrimidine-2,4-dione **(3d)**

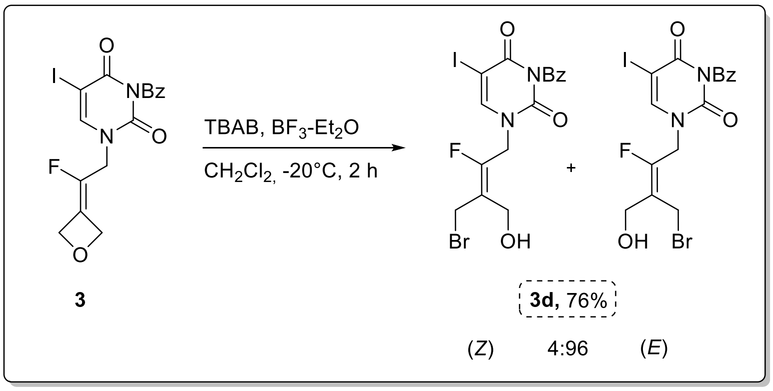

To the oxetane **3** (43 mg, 0.097 mmol, 1 equiv) and tetrabutylammonium bromide (78 mg, 0.243 mmol, 2.5 equiv) in CH_2Cl_2 (3 mL) at −20 °C was added $BF_3·Et_2O$ (0.013 mL, 0.107 mmol, 1.1 equiv). The mixture was stirred at −20 °C for 2 h, then usual work-up procedure was followed with Et_2O. NMR analysis of the crude mixture showed a complete conversion into the products *E*-**3d** and *Z*-**3d** (*E*/*Z* ratio 96:4). Column chromatography CH_2Cl_2/EtOAc (80/20) gave a *E*/*Z* ratio (96:4) of compound **3d** (39 mg, 76%) as white solid.

***E*-3d.** ^1H NMR (600 MHz, $CDCl_3$) δ 7.93 – 7.87 (m, 2H), 7.83 – 7.77 (m, 1H), 7.68 (tt, $^3J_{HH}$ = 7.4 Hz, $^4J_{HH}$ = 1.3 Hz, 1H), 7.54 – 7.47 (m, 2H), 4.62 (d, $^3J_{HF}$ =21.1 Hz, 2H), 4.41 (d, $^4J_{HF}$ = 3.0 Hz, 2H), 4.26 (d, $^4J_{HF}$ = 1.3 Hz, 2H), 1.67 (s, 1H). ^{19}F NMR (471 MHz, $CDCl_3$) δ -109.45 (t, $^3J_{FH}$ =21.1 Hz). ^{13}C NMR (151 MHz, $CDCl_3$) δ 167.51, 159.13, 151.71 (d, $^1J_{CF}$ = 258.2 Hz), 149.47, 147.92, 135.71, 130.78 (2C), 130.76, 129.47 (2C), 121.81 (d, $^2J_{CF}$ = 15.1 Hz), 68.80, 56.53 (d, $^3J_{CF}$ = 8.3 Hz), 45.66 (d, $^2J_{CF}$ = 28.3 Hz), 26.56 (d, $^3J_{CF}$ = 9.3 Hz). HRMS ES+ (m/z) $[M + H]^+$ calculated for $C_{16}H_{14}N_2O_4FBrI^+$ 522.9157, found 522.9154.

4. Synthesis of oxetanes 4 and 5

N-[2-(Oxetan-3-ylidene)ethyl] phthalimide **(4)**

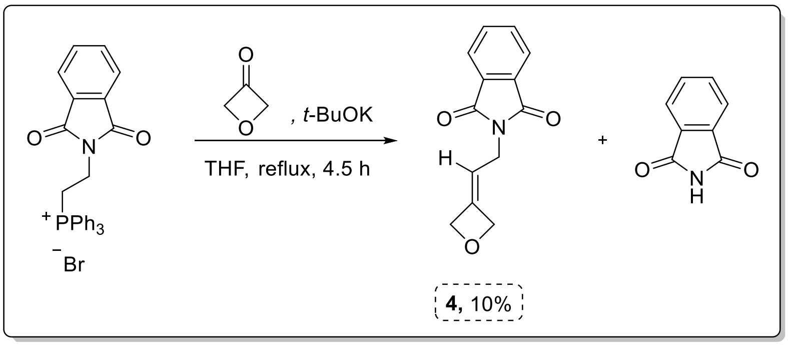

To a mixture of 1-(2-phthalimidoethyl)-triphenylphosphonium bromide[i] (380 mg, 0.736 mmol, 1 equiv) and 3-oxetanone (0.043 mL, 0.736 mmol, 1 equiv) in dry THF (2.5 mL) at 0 °C was added *t*-BuOK (91 mg, 0.809 mmol, 1.1 equiv). The reaction mixture was refluxed for 4.5 h then cooled to room temperature. NH_4Cl (sat. aq., 8 mL) was added and the mixture was extracted with EtOAc (2 × 20 mL). The combined organic layers were washed with brine (20 mL), then treated as usual. Column chromatography (CH_2Cl_2/EtOAc 98:2 then 92:8 then 90:10) gave 28 mg of a 1:1 mixture of the desired product **4** and phthalimide which was used as obtained in the next step.

^1H NMR (500 MHz, $CD_3OD/CDCl_3$) δ 7.86 – 7.78 (m, 4H, H_{Ar}), 7.78 – 7.71 (m, 4H, H_{Ar}), 5.36 (br s, 2H, CH_2O), 5.34 – 5.27 (m, 1H, CH), 5.18 (br s, 2H, CH_2O), 4.08 (d, $^3J_{HH}$ = 6.9 Hz, 2H, CH_2N). ^{13}C NMR (126 MHz, $CD_3OD/CDCl_3$) δ 170.5 (2C), 168.8 (2C), 140.2, 135.0 (2C), 134.8 (2C), 133.6 (2C), 132.6 (2C), 123.9 (2C), 123.8 (2C), 114.1, 79.8, 79.4, 35.60.

[i] Wolin, R.; Connolly, M.; Afonso, A.; Hey, J. A.; She, H.; Rivelli, M. A.; Willams, S. M.; West, R. E. *Bioorg. Med. Chem. Lett.* **1998**, *8* (16), 2157–2162. doi:10.1016/S0960-894X(98)00379-5

3-Decylideneoxetane **(5)**

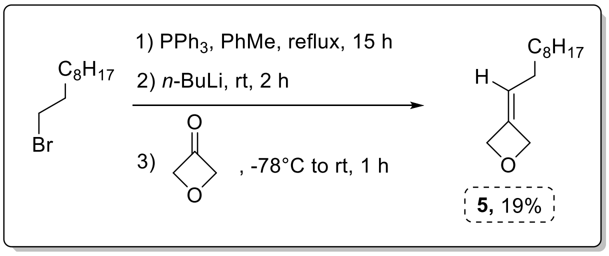

In a 50 mL 2-neck round-bottomed flask, a mixture of decyl bromide (0.938 mL, 4.52 mmol, 1 equiv) and triphenylphosphine (1.19 g, 4.52 mmol, 1 equiv) in toluene (2 mL) was refluxed for 15 h. The reaction mixture was cooled to rt before the addition of Et_2O (15 mL) upon which a white gum formed. *n*-BuLi (2.5 M in hexane, 1.81 mL, 4.52 mmol, 1 equiv) was then added dropwise at rt upon which a red-orange color developed. The reaction mixture was stirred for 2 h then cooled to −78 °C and 3-oxetanone (0.265 mL, 4.52 mmol, 1 equiv) solution in Et_2O (5 mL) were added dropwise while keeping the temperature below −55 °C. The mixture was allowed to warm to rt and stirred for 1 h after which the precipitate was filtered and rinsed with Et_2O followed by concentration of the filtrate. Column chromatography (pentane/Et_2O 95:5) gave 165 mg (0.840 mmol, 19%) of **5** as colorless oil and contaminated with approximately 40% of an unidentified compound bearing a three-carbon alkyl chain.

^{1}H NMR (600 MHz, $CDCl_3$) δ 5.24 – 5.21 (m, 2H, CH_2O), 5.20 – 5.17 (m, 2H, CH_2O), 5.15 – 5.10 (m, 1H, $CH_2C\underline{H}$), 1.87 – 1.80 (m, 2H, $C\underline{H_2}CH$), 1.40 – 1.21 (m, 14H + 2.9H), 0.89 (t, $^3J_{HH}$ = 7.1 Hz, 3H + 1.7H). ^{13}C NMR (126 MHz, $CDCl_3$) δ 133.5, 119.8, 79.5, 79.0, 31.9, 29.6, 29.5, 29.3, 29.2, 29.1, 28.2, 22.7, 14.1. Peaks for the impurity: 34.1, 22.4, 14.1.

5. Ring-opening reaction of oxetanes 4 and 5

N-[(*E*)-3-(Bromomethyl)-4-hydroxy-but-2-en-1-yl] phthalimide **(*E*-4d)**

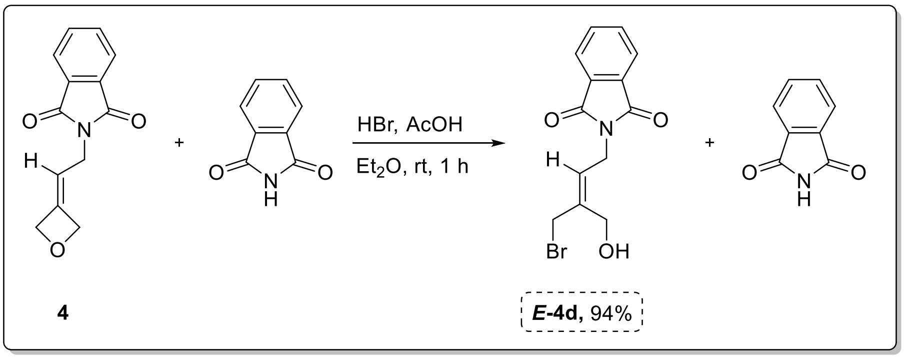

To the mixture obtained above of oxetane **4** (17 mg, 0.074 mmol, 1 equiv) and phthalimide (11 mg, 0.074 mmol, 1 equiv) in Et_2O (2 mL) at 0 °C was added a solution of HBr (33 wt % in AcOH, 0.030 mL, 0.371 mmol, 5 equiv) diluted in Et_2O (0.12 mL). The mixture was stirred at rt for 1 h then usual work-up procedure was followed with EtOAc (1 vol. = 10 mL). NMR analysis of the crude mixture showed a 1:1 mixture of *E*-**4d** (single isomer) and phthalimide. Column chromatography (pentane/EtOAc 70:30) gave 21 mg (0.069 mmol, 94%) of *E*-**4d** as a white solid. Further crystallization gave an analytically pure sample for X-ray analysis page **S21**.

^{1}H NMR (600 MHz, $CDCl_3$) δ 7.87 – 7.83 (m, 2H, H_{Ar}), 7.77 – 7.72 (m, 2H, H_{Ar}), 5.71 (t, $^3J_{HH}$ = 7.8 Hz, 1H, CH), 4.49 (br s, 2H, CH_2O), 4.43 (d, $^3J_{HH}$ = 7.8 Hz, 2H, CH_2N), 4.07 (s, 2H, CH_2Br). ^{13}C NMR (151 MHz, $CDCl_3$) δ 168.1 (2C), 141.2, 134.3 (2C), 131.9, 125.1 (2C), 123.5 (2C), 58.0, 35.40, 34.80.

(*E*)-2-(Bromomethyl)dodec-2-en-1-ol **(*E*-5d)**

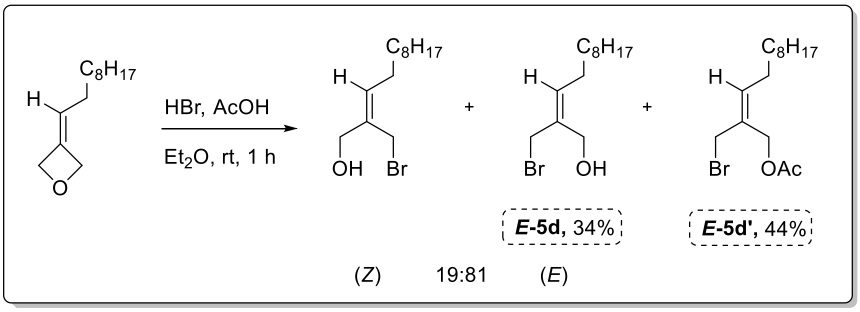

To the impure oxetane **5** obtained above (55 mg, 0.280 mmol, 1 equiv) in Et_2O (3.35 mL) at 0 °C was added a solution of HBr (33 wt % in AcOH, 0.161 mL, 0.924 mmol, 3.3 equiv) diluted in Et_2O (0.5 mL). The mixture was stirred at rt for 1 h then usual work-up procedure was followed (1 vol. = 10 mL). NMR analysis of the crude mixture showed a complete conversion into the products **5d** (*E*/*Z* ratio 81:19) and **5d'** (*E*/*Z* ratio 100:0). Column chromatography (pentane/Et_2O 98:2 then 85:15) gave 40 mg (0.12 mmol, 44%) of *E*-**5d'** and 27 mg (0.01 mmol, 34%) of *E*-**5d** as colorless oil. Note: The product is not stable and degrades at rt overnight.

^{1}H NMR (600 MHz, $CDCl_3$) δ 5.77 (t, $^3J_{HH}$ = 7.5 Hz, 1H, $CH_2C\underline{H}$), 4.34 (s, 2H, CH_2O), 4.15 (s, 2H, CH_2Br), 2.11 (dt, appears as q, $^3J_{HH}$ = 7.5 Hz, 2H, $C\underline{H_2}CH$), 1.42 – 1.19 (m, 14H), 0.89 (t, $^3J_{HH}$ = 7.0 Hz, 3H, CH_3). ^{13}C NMR (151 MHz, $CDCl_3$) δ 135.1, 135.0, 58.5, 37.3, 31.9, 29.5, 29.4, 29.3, 29.22, 29.19, 27.9, 22.7, 14.1. HRMS (ASAP+) *m*/*z* calcd for $[M-H]^+$ $C_{13}H_{24}O^{79}Br$ 275.1011, found 275.1013, $[M-OH^-]^+$ $C_{13}H_{24}{}^{79}Br$ 259.1061, found 259.1063

[(*E*)-2-(Bromomethyl)dodec-2-enyl] acetate **(*E*-5d')**

^{1}H NMR (500 MHz, $CDCl_3$) δ 5.86 (t, $^3J_{HH}$ = 7.5 Hz, 1H, $CH_2C\underline{H}$), 4.75 (s, 2H, CH_2O), 4.06 (s, 2H, CH_2Br), 2.13 (dt, appears as br q, $^3J_{HH}$ = 7.4 Hz, 2H, $C\underline{H_2}CH$), 2.08 (s, 3H, CH_3), 1.42 – 1.33 (m, 2H), 1.33 – 1.22 (m, 12H), 0.88 (t, J = 6.6 Hz, 3H, CH_3).

^{13}C NMR (126 MHz, $CDCl_3$) δ 170.8, 137.5, 131.0, 59.7, 36.5, 31.8, 29.5, 29.4, 29.3, 29.1, 29.0, 28.1, 22.6, 20.9, 14.1. Note: The ^{13}C spectrum was obtained from another batch than the ^{1}H spectrum. For this batch, *E*-**5d'** was obtained with some Et_2O and the same impurity present with oxetane **5** hence the presence of the same extra peaks. HRMS (ASAP+) *m*/*z* calcd for $[M+H]^+$ $C_{15}H_{28}O_2{}^{79}Br$ 319.1273, found 319.1270, $[M-AcO^-]^+$ $C_{13}H_{24}{}^{79}Br$ 259.1061, found 259.1061.

6. Functionalization of compound 1d

N-[(*E*)-2-Fluoro-3-(fluoromethyl)-4-hydroxy-but-2-enyl] phthalimide **(*E*-23)**

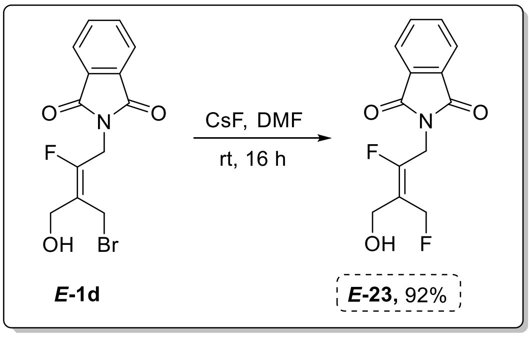

Cesium fluoride (93 mg, 0.61 mmol, 2 equiv) was added to a solution of *E*-**1d** (100 mg, 0.30 mmol, 1 equiv) in DMF (5 mL). The mixture was stirred overnight at room temperature. DMF was evaporated under reduced pressure and the residue was taken up in dichloromethane and water. The organic layer was separated, washed with brine, dried over $MgSO_4$, filtered, and concentrated to afford compound *E*-**23** (75 mg, 92%) as white crystal.

^{1}H NMR ($CDCl_3$, 400 MHz) δ 7.87-7.83 (m, 2H), 7.76-7.72 (m, 2H), 5.29 (dd, $^2J_{HF}$ = 47.7 Hz, $^4J_{HF}$ = 1.8 Hz, 2H), 4.55 (dd, $^3J_{HF}$ = 20.6 Hz, $^5J_{HF}$ = 2.2 Hz, 2H), 4.36-4.31 (m, 2H), 1.91 (brt, $^3J_{HH}$ = 6.2 Hz, 1H). **^{13}C NMR** ($CDCl_3$, 100 MHz) δ 167.4 (2C), 155.1 (dd, $^1J_{CF}$ = 262.7 Hz, $^3J_{CF}$ = 12.1 Hz), 134.4 (2C), 131.8 (2C), 123.6 (2C), 117.3 (dd, $^2J_{CF}$ = 15.2 Hz, $^2J_{CF}$ = 13.4 Hz), 78.8 (dd, $^1J_{CF}$ = 163.2 Hz, $^3J_{CF}$ = 10.4 Hz), 56.5 (d, $^3J_{CF}$ = 8.9 Hz), 35.1 (dd,

$^{2}J_{CF}$ = 29.7 Hz, $^{4}J_{CF}$ = 1.4 Hz). **^{19}F NMR** ($CDCl_3$, 376 Hz) δ -107.1 (t, $^{3}J_{FH}$ = 20.6 Hz), -212.3 (t, $^{2}J_{FH}$ = 47.7 Hz). **HRMS** ES+ (m/z) [M + H]$^+$ calculated for $C_{13}H_{11}NO_3F_2Na^+$ 290.0605, found 290.0608.

N-[(*Z*)-3-(Azidomethyl)-2-fluoro-4-hydroxy-but-2-enyl] phthalimide (*Z*-**24)**

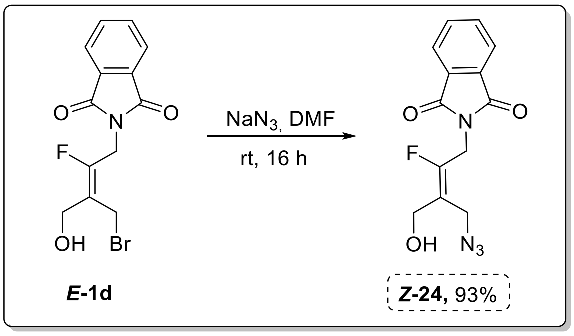

Sodium azide (40 mg, 0.61 mmol, 2 equiv) was added to a solution of *E*-**1d** (100 mg, 0.30 mmol, 1 equiv) in DMF (5 mL). The mixture was stirred overnight at room temperature. DMF was evaporated under reduced pressure and the residue was taken up in dichloromethane and water. The organic layer was isolated, washed with brine, dried over $MgSO_4$, filtered, and concentrated. Purification by flash chromatography on silica gel (CH_2Cl_2/MeOH, 95:5) afforded compound *Z*-**24** (82 mg, 93%) as white solid.

^{1}H NMR ($CDCl_3$, 400 MHz) δ 7.90-7.82 (m, 2H), 7.78-7.72 (m, 2H), 5.46 (d, $^{3}J_{HF}$ = 30.2 Hz, 2H), 4.33 (s, 2H), 4.16 (d, $^{4}J_{HF}$ = 2.5 Hz, 1H), 4.12 (d, $^{4}J_{HF}$ = 5.3 Hz, 1H), 2.99 (s, 1H).^{13}C NMR ($CDCl_3$, 100 MHz) δ 167.9 (2C), 142.8 (d, $^{2}J_{CF}$ = 23.7 Hz), 134.5 (2C), 131.6 (2C), 123.8 (2C), 118.2 (d, $^{3}J_{CF}$ = 8.6 Hz), 106.1 (d, $^{1}J_{CF}$ = 226.3 Hz), 62.4 (d, $^{3}J_{CF}$ = 4.1 Hz), 43.2 (d, $^{2}J_{CF}$ = 30.6 Hz).^{19}F NMR ($CDCl_3$, 376 Hz) δ -122.66 (m). HRMS (ES+) *m/z* [M + Na]$^+$ calculated for $C_{13}H_{11}N_4O_3FNa$ 313.0713, found 313.0716.

N-[(Z)-2-Fluoro-3-(hydroxymethyl)-4-pyrrolidin-1-yl-but-2-enyl] phthalimide **(*Z*-25)**

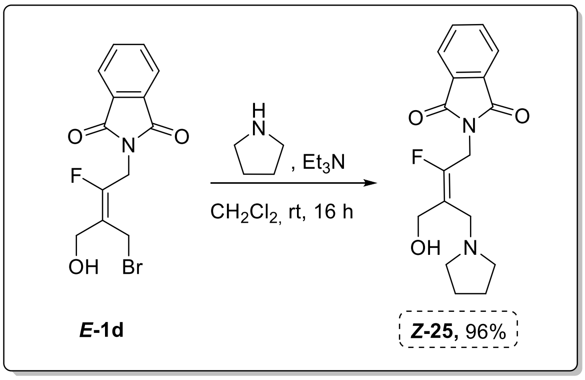

Triethylamine (82 µL, 0.61 mmol, 2 equiv) was added to a solution of bromo-alcohol (100 mg, 0.30 mmol, 1 equiv) and pyrrolidine (38 µL, 0.46 mmol, 1.5 equiv) in dichloromethane (5 mL). The mixture was stirred overnight at room temperature. Dichloromethane was evaporated under reduced pressure and the residue was taken up in dichloromethane and water. The organic layer was separated, washed with brine, dried over $MgSO_4$, filtered, and concentrated. Purification by flash chromatography on silica gel (CH_2Cl_2/MeOH, 95:5) afforded compound *Z*-**25** (93 mg, 96%) as a white solid. Further crystallization gave an analytical sample for X-ray analysis page **S23**.

^{1}H NMR ($CDCl_3$, 400 MHz) δ 7.88-7.82 (m, 2H), 7.76-7.70 (m, 2H), 5.37 (s, 1H), 4.52 (d, $^{3}J_{HF}$ = 19.9 Hz, 2H), 4.39 (d, $^{4}J_{HF}$ = 3.0 Hz, 2H), 3.59 (s, 2H), 2.79-2.70 (m, 4H), 1.88-1.81 (m, 4H).^{13}C NMR ($CDCl_3$, 100 MHz) *δ* 167.5 (2C), 150.1 (d, $^{1}J_{CF}$ = 254.2 Hz), 134.3 (2C), 131.9 (2C), 123.5 (2C), 117.1 (d, $^{2}J_{CF}$ = 13.9 Hz), 59.5 (d, $^{3}J_{CF}$ = 10.6 Hz), 54.3 (d, $^{3}J_{CF}$ = 7.0 Hz), 53.9 (2C), 34.7 (d, $^{2}J_{CF}$ = 29.9 Hz), 23.5 (2C).^{19}F NMR ($CDCl_3$, 376 Hz) δ -110.6 (m). HRMS (ESI) *m/z* [M + H]$^+$ calculated for $C_{17}H_{20}N_2O_3F$ 319.1458, found 319.1462.

N-[(*E*)-3-(1,3-Benzothiazol-2-ylsulfanylmethyl)-2-fluoro-4-hydroxy-but-2-enyl] phthalimide **(*E*-1c)**

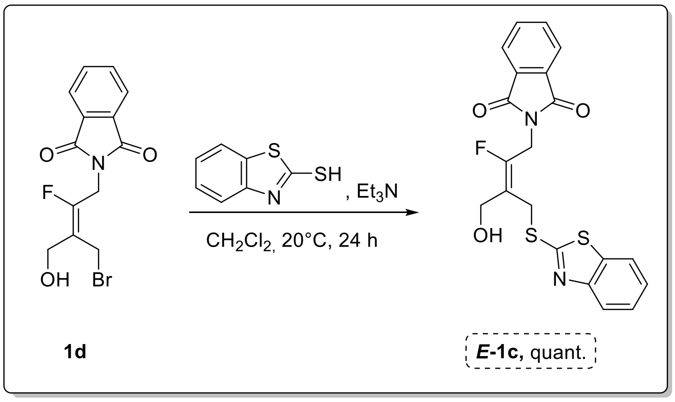

Triethylamine (82 µL, 0.61 mmol, 2 equiv) was added to a solution of bromo-alcohol **1d** (100 mg, 0.30 mmol, 1 equiv) and 2-mercaptobenzothiazol (76 mg, 0.46 mmol, 1.5 equiv) in dichloromethane (5 mL). The mixture was stirred overnight at room temperature. Dichloromethane was evaporated under reduced pressure and the residue was taken up in dichloromethane and water. The organic layer was isolated, washed with brine, dried over $MgSO_4$, filtered, and concentrated. Purification by flash chromatography on silica gel (CH_2Cl_2/MeOH, 95:5) afforded compound *E*-**1c** in quantitative yield as white solid.

^{1}H NMR ($CDCl_3$, 400 MHz) δ 7.86-7.45 (m, 3H), 7.72-7.64 (m, 3H), 7.40-7.33 (m, 1H), 7.29-7.23 (m, 1H), 4.73 (s, 1H), 4.55 (d, $^3J_{HF}$ = 21.8 Hz, 2H), 4.43 (s, 2H), 4.32 (d, $^4J_{HF}$ = 2.5 Hz, 2H).^{13}C NMR ($CDCl_3$, 100 MHz) δ 168.5, 167.5 (2C), 152.1, 151.2 (d, $^1J_{CF}$ = 256.7 Hz), 135.1, 134.3 (2C), 131.9 (2C), 126.4, 124.8, 123.6 (2C), 121.5, 121.1, 119.2 (d, $^2J_{CF}$ = 15.0 Hz), 55.5 (d, $^3J_{CF}$ = 9.9 Hz), 35.4 (d, $^2J_{CF}$ = 31.0 Hz), 30.5 (d, $^3J_{CF}$ = 7.0 Hz).^{19}F NMR ($CDCl_3$, 376 Hz) δ -108.7 (t, $^3J_{HF}$ = 21.8 Hz, 1F). HRMS (ESI) *m/z* [M + H]$^+$ calculated for $C_{20}H_{16}N_2O_3FS_2^+$ 415.0586, found 415.0591.

7. *Synthesis of benzyloxy-substituted fluoroethylideneoxetane derivative 8.*

2-(2-Benzyloxy-1-fluoro-ethyl)sulfonyl-1,3-benzothiazole **(7)**

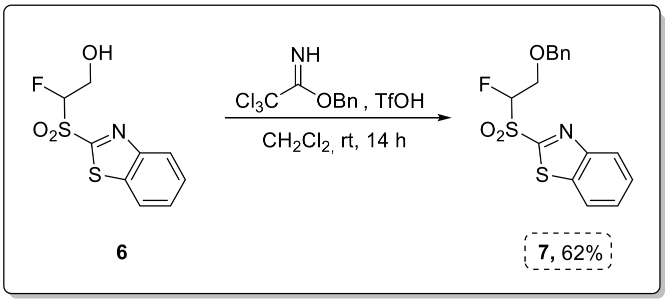

To a mixture of compound **6** (0.370 g, 1,416 mmol) and benzyl trichloroacetimidate (0.395 mL, 2,124 mmol) in CH_2Cl_2 (10 mL) at rt was added dropwise TfOH (0.026 mL, 0.284 mmol). The reaction mixture was stirred at room temperature for 14 h, then quenched with saturated $NaHCO_3$ aqueous solution (10 mL). The layers were separated and the aqueous phase was extracted with CH_2Cl_2. The organic phase was dried with $MgSO_4$, filtered, and concentrated in vacuo. The crude product was purified by column chromatography using pentane/AcOEt (90/10) as eluent. Compound **7** (0.311 g, 0.885 mmol) was obtained with 62% yield.

^{1}H NMR (500 MHz, $CDCl_3$) δ 8.29 – 8.23 (m, 1H), 8.04 – 8.00 (m, 1H), 7.70 – 7.60 (m, 2H), 7.35 – 7.27 (m, 5H), 5.85 (ddd, $^2J_{HF}$ = 47.9 Hz, $^3J_{HH}$ = 6.4 Hz, $^3J_{HH}$ = 2.8 Hz, 1H), 4.63 (s, 2H), 4.31 (ddd, $^3J_{HF}$ = 27.2 Hz, $^2J_{HH}$ =12.4 Hz, $^3J_{HH}$ = 2.8 Hz, 1H), 4.15 (ddd, $^3J_{HF}$ = 21.1 Hz, $^2J_{HH}$ =12.4 Hz, $^3J_{HH}$ =6.4 Hz, 1H).^{13}C NMR (126 MHz, $CDCl_3$) δ 162.5, 152.9, 137.6, 136.7, 128.7 (2C), 128.6, 128.3, 128.0, 127.9 (2C), 126.0, 122.5, 101.1 (d, $^1J_{CF}$ = 226.8 Hz), 74.2, 65.9 (d, $^2J_{CF}$ = 20.2 Hz).^{19}F NMR (471 MHz, $CDCl_3$) δ -182.49 (ddd, $^2J_{FH}$ = 47.9 Hz, $^3J_{FH}$ = 27.2 Hz, $^3J_{FH}$ = 21.1 Hz). HRMS-ESI (m/z) [M+H]$^+$ calculated for $C_{16}H_{15}NO_3S_2F^+$ 352.0477, found 352.0479.

3-(2-Benzyloxy-1-fluoro-ethylidene)oxetane **(8)**

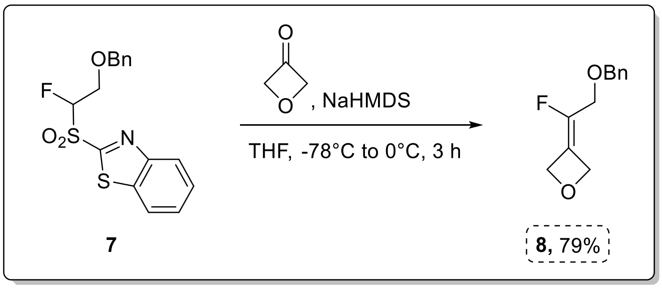

To a mixture of compound **7** (0.310 g, 0.882 mmol) and 3-oxetanone (0.062 mL, 0.970 mmol) in THF (9 mL) at −78 °C was added dropwise a solution of 1 M of NaHMDS in THF (1.323 mL, 1.323 mmol). Te reaction mixture was allowed to reach room temperature, then quenched with saturated NH_4Cl aqueous solution (10 mL). The mixture was extracted with Et_2O. The organic phase was washed with brine, dried with $MgSO_4$, filtered, and concentrated in vacuo. The crude product was purified by column chromatography using pentane/Et_2O (90/10) as eluent. Compound **8** (0.146 g, 0.701 mmol) was obtained with 79% yield.

^{1}H NMR (600 MHz, $CDCl_3$) δ 7.40 – 7.28 (m, 5H), 5.26 (dtt, $^4J_{HF}$ = 4.2 Hz, $^4J_{HH}$ = 2.6 Hz, $^5J_{HH}$ = 1.4 Hz, 2H), 5.18 (dtt, $^4J_{HF}$ = 4.2 Hz, $^4J_{HH}$ = 2.6 Hz, $^5J_{HH}$ = 1.4 Hz, 2H), 4.55 (s, 2H), 3.96 (dquint, $^3J_{HF}$ = 13.1 Hz, $^5J_{HH}$ = 1.4 Hz, 2H).^{19}F NMR (471 MHz, $CDCl_3$) δ -124.42 (dquint, $^3J_{FH}$ = 13.1 Hz, $^4J_{FH}$ = 4.2 Hz).^{13}C NMR (151 MHz, $CDCl_3$) δ 145.99 (d, $^1J_{CF}$ = 250.7 Hz), 137.33, 128.63 (2C), 128.08, 127.79 (2C), 115.60 (d, $^2J_{CF}$ = 22.1 Hz), 75.45 (d, $^3J_{CF}$ = 7.2 Hz), 74.93 (d, $^3J_{CF}$ = 11.7 Hz), 72.72, 65.09 (d, $^2J_{CF}$ = 31.2 Hz). HRMS-ESI (m/z) $[M+H]^+$ calculated for $C_{12}H_{14}O_2F^+$ 209.0978, found 209.0972.

8. *Ring-opening reaction of the benzyloxy-substituted fluoroethylideneoxetane 8*

4-Benzyloxy-2-(bromomethyl)-3-fluoro-but-2-en-1-ol **(9)**

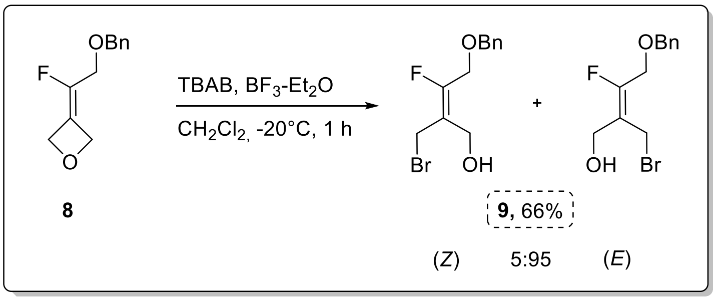

To the oxetane **8** (132 mg, 0.634 mmol, 1 equiv) and tetrabutylammonium bromide (511 mg, 1.59 mmol, 2.5 equiv) in CH_2Cl_2 (5 mL) at −20 ºC was added $BF_3 \cdot Et_2O$ (0.086 mL, 0.107 mmol, 1.1 equiv). The mixture was stirred at -20°C for 1 h then usual work-up procedure was followed with Et_2O. NMR analysis of the crude mixture showed a complete conversion into the products ***E*-9** and ***Z*-9** (*E*/*Z* ratio 90:10). Column chromatography pentane/EtOAc (90/10) gave a *E*/*Z* ratio (95:5) of compound **9** (183 mg, 66%) as colorless oil.

^{1}H NMR (500 MHz, $CDCl_3$) δ 7.40 – 7.30 (m, 5H), 4.59 (s, 2H), 4.39 (d, $^4J_{HF}$ = 3.2 Hz, 2H), 4.19 (d, $^3J_{HF}$ = 21.1 Hz, 2H), 4.16 (d, $^4J_{HF}$ = 1.7 Hz, 2H), 1.94 (s, 1H).^{19}F NMR (471 MHz, $CDCl_3$) δ -105.00 (t, $^3J_{FH}$ = 21.1 Hz).^{13}C NMR (151 MHz, $CDCl_3$) δ 156.06 (d, $^1J_{CF}$ = 262.9 Hz), 137.23, 128.67 (2C), 128.20, 128.02 (2C), 119.58 (d, $^2J_{CF}$ = 16.2 Hz), 72.90, 64.51 (d, $^2J_{CF}$ = 30.5 Hz), 56.48 (d, $^3J_{CF}$ = 8.6 Hz), 27.74 (d, $^3J_{CF}$ = 9.8 Hz). HRMS-ESI (m/z) $[M+H]^+$ does not succeed probably due to the non-ionization of the sample.

(4-Fluoro-2,5-dihydrofuran-3-yl)methanol **(10)**

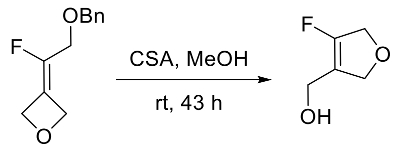

Camphorsulfonic acid (80 mg, 0.346 mmol, 1 equiv) was added to a solution of **8** (72 mg, 0.346 mmol, 1 equiv) in MeOH (1 mL, 23 mmol, 66 equiv). The mixture was stirred at room temperature for 43 h, then usual work up was followed with Et_2O. NMR analysis of the crude mixture showed a 95% conversion into the product **10** and **11** (90/10, see the main text). Column chromatography (CH_2Cl_2/EtOAc 60/40) gave the compound **10** (30 mg, 75%) as colorless liquid.

^{1}H NMR (500 MHz, $CDCl_3$) δ 4.76 – 4.66 (m, 2H), 4.58 – 4.51 (m, 2H), 4.29 (s, 2H), 2.04 (s, 1H).^{19}F NMR (471 MHz, $CDCl_3$) δ -141.14 – -141.22 (m).^{13}C NMR (126 MHz, $CDCl_3$) δ 151.79 (d, $^1J_{CF}$ = 278.1 Hz), 111.04 (d, $^2J_{CF}$ = 7.1 Hz), 73.51 (d, $^3J_{CF}$ = 9.4 Hz), 68.90 (d, $^2J_{CF}$ = 28.1 Hz), 53.82.

9. *Synthesis of the acetate derivatives 12 and 16*

Ethyl 2-fluoro-2-(oxetan-3-ylidene)acetate **(12)**

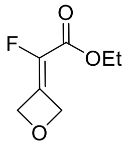

Compound **12** was synthesized according to the procedure by Thierry et al. starting from 1.67 mL (27.25 mmol) of 3-oxetanone to offer 3.97 g product (20.82 mmol, 84%). The spectral data matched with the literature.[i]

Oxetan-3-ylidene-acetic acid ethyl ester **(16)**

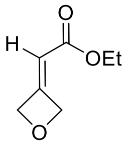

Compound **16** was synthesized according to the procedure of Wuitschik et al. starting from 0.181 mL (3.01 mmol) of 3-oxetanone to offer 411 mg product (2.89 mmol, 96%). The spectral data matched with the literature.[ii]

10. Ring–opening reaction from acetate derivatives 12 and 16

Ethyl (*Z*)-4-bromo-2-fluoro-3-(hydroxymethyl)but-2-enoate **(*Z*-13)**

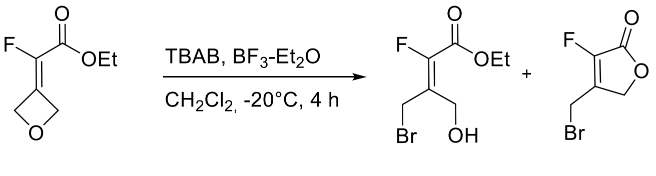

Procedure for Table 4, entry 4: To the oxetane **12** (200 mg, 1.25 mmol, 1 equiv) in CH_2Cl_2 (6 mL) at −40 °C was added TBAB (805 mg, 2.50 mmol, 2 equiv) followed by BF_3·OEt_2 (0.23 mL, 1.87 mmol, 1.5 equiv). The mixture was stirred at −20 °C for 4 h after which only *Z*-**13** was observed by TLC analysis. Usual work-up procedure was followed with Et_2O (1 vol. = 10 mL). During the second extraction, a solid formed (probably TBAB) which was discarded. The combined organic layers were washed with water

[i] Thierry, T.; Lebargy, C.; Pfund, E.; Lequeux, T. *J. Org. Chem.* **2019**, *84* (9), 5877–5885. doi:10.1021/acs.joc.9b00244.
[ii] Wuitschik, G.; Rogers- Evans, M.; Müller, K.; Fischer, H.; Wagner, B.; Schuler, F.; Polonchuk, L.; Carreira, E. M. *Angewandte Chemie International Edition* **2006**, *45* (46), 7736–7739. doi:10.1002/anie.200602343

(2 × 10 mL) then dried ($MgSO_4$), filtered, and concentrated to give 274 mg of crude. NMR analysis of the crude mixture showed a complete conversion into the products *Z*-**13** and **15** with a 78:22 ratio. Calculated yields gave 72% for *Z*-**13** and 21% for **15**. The product was used without further purification.

^{1}H NMR (500 MHz, $CDCl_3$) δ 4.58 (dd, $^{3}J_{HH}$ = 7.0 Hz, $^{4}J_{HF}$ = 2.9 Hz, 2H, CH_2O), 4.35 (q, $^{3}J_{HH}$ = 7.1 Hz, 2H, C<u>H$_2$</u>CH_3), 4.25 (d, $^{4}J_{HF}$ = 3.4 Hz, 2H, CH_2Br), 2.71 (t, $^{3}J_{HH}$ = 7.1 Hz, 1H, OH), 1.38 (t, $^{3}J_{HH}$ = 7.1 Hz, 3H, CH_2C<u>H$_3$</u>). ^{13}C NMR (126 MHz, $CDCl_3$) δ 160.7 (d, $^{2}J_{CF}$ = 33.8 Hz), 146.0 (d, $^{1}J_{CF}$ = 269.3 Hz), 130.7 (d, $^{2}J_{CF}$ = 8.1 Hz), 62.3, 58.5 (d, $^{3}J_{CF}$ = 3.5 Hz), 25.0 (d, $^{3}J_{CF}$ = 10.4 Hz), 13.9.^{19}F NMR (471 MHz, $CDCl_3$) δ -119.47 – -119.56 (m, 1F).$^{19}F\{^{1}H\}$ NMR (471 MHz, $CDCl_3$) δ -119.52 (s, 1F).

4-(Hydroxymethyl)-3-fluoro-2(5*H*)-furanone **(14)**

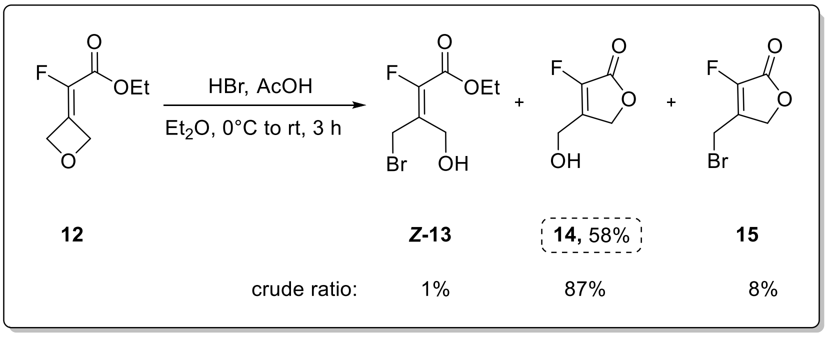

Procedure for Table 4, entry 1: To the oxetane **12** (55 mg, 0.34 mmol, 1 equiv) in Et_2O (3.4 mL) at 0 °C was added a solution of HBr (33 wt % in AcOH, 0.14 mL, 0.81 mmol, 2.3 equiv) diluted in Et_2O (0.6 mL). The mixture was stirred at 0 °C for 2 h, then allowed to reach rt over 1 h. After 0.5 h at rt, usual work-up procedure was followed with Et_2O (1 vol. = 5 mL). NMR analysis of the crude mixture showed a 92% conversion into the products **14**, **15** and *Z*-**13** with a ratio of 87:8:1 with the remaining 4% probably being acetylated products. Column chromatography (pentane/acetone 90:10, 80:20 then 70:30) gave 4 mg of an 80:20 mixture of **15**/*Z*-**13** and 26 mg (0.20 mmol, 58%) of lactone **14** as a colorless oil. The spectral data matched with the literature.[i] Product **14** (24 mg, 67%) was also obtained starting from **12** by using $BF_3{\cdot}OEt_2$ (0.23 mL, 1.87 mmol, 1.5 equiv) in CH_2Cl_2 at −20 °C during 2 h, followed by column chromatography (pentane/acetone, 80:20).

4-(Bromomethyl)-3-fluoro-2(5*H*)-furanone **(15)**

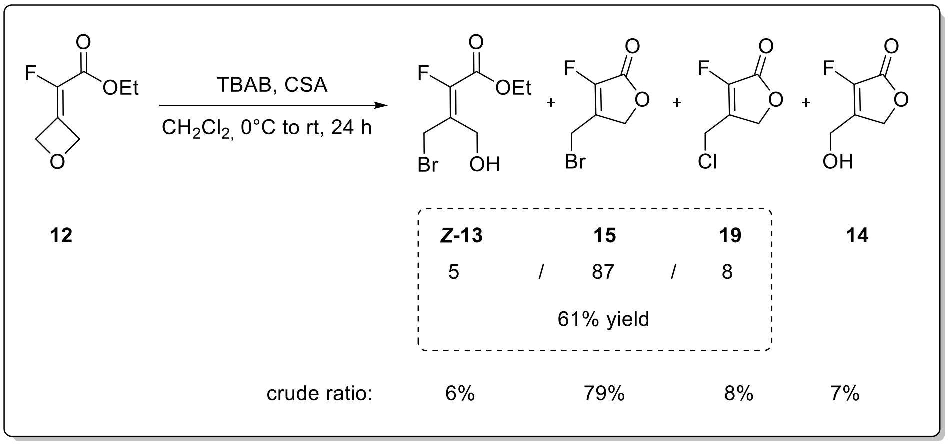

Procedure for Table 4, entry 3: To the oxetane **12** (150 mg, 0.937 mmol, 1 equiv) in CH_2Cl_2 (5 mL) at 0 ºC was added TBAB (755 mg, 2.34 mmol, 2.5 equiv) followed by CSA (218 mg, 0.937 mmol, 1 equiv). The mixture was stirred at 0 °C for 1.5 h, then allowed to reach rt. After 24 h, CSA (218 mg, 0.937 mmol, 1 equiv) was added and the resulting mixture was stirred at rt for 6 h. Usual work-up procedure was followed with Et_2O (1 vol. = 5 mL). The combined organic layers were washed with brine (10 mL), then dried ($MgSO_4$), filtered, and concentrated to give 156 mg of crude. NMR analysis of the crude mixture showed an 83% conversion into the products **15**, **19**, **14** and *Z*-**13** with a

[i] A. Fort, D.; J. Woltering, T.; M. Alker, A.; Bach, T. *Heterocycles* **2014**, *88* (2), 1079. doi:10.3987/COM-13-S(S)67

ratio of 79:8:7:6. Column chromatography (pentane/Et_2O 75:25 then 70:30) gave 112 mg of an 87:8:5 mixture of products **15**/**19**/*Z*-**13** (61% yield). The spectral data for **15** matched with the literature.[i]

Data for 4-(chloromethyl)-3-fluoro-2(5*H*)-furanone **(19)**

^{1}H NMR (600 MHz, $CDCl_3$) δ 4.91 (d, $^4J_{HF}$ = 5.7 Hz, 2H, CH_2O), 4.21 (br s, 2H, CH_2Cl). ^{13}C NMR (126 MHz, $CDCl_3$) δ 164.1 (d, $^2J_{CF}$ = 30.5 Hz), 144.0 (d, $^1J_{CF}$ = 279.1 Hz), 132.8 (d, $^2J_{CF}$ = 5.5 Hz), 66.7 (d, $^3J_{CF}$ = 5.7 Hz), 32.9 (d, $^3J_{CF}$ = 2.0 Hz). ^{19}F NMR (471 MHz, $CDCl_3$) δ -143.54 – -143.60 (m, 1F).

11. Opening reaction of ethyl 2-(oxetanyl-3-idene)acetate 16

4-(Bromomethyl)-2(5*H*)-furanone **(18)**

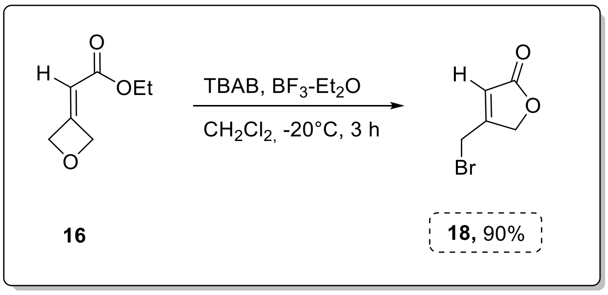

To the oxetane **16** (132 mg, 0.92 mmol, 1 equiv) in CH_2Cl_2 (4 mL) at -20 ºC was added TBAB (592 mg, 1.84 mmol, 2 equiv) followed by $BF_3{\cdot}OEt_2$ (0.17 mL, 1.38 mmol, 1.5 equiv). The mixture was stirred at −20 °C for 3 h, then usual work-up procedure was followed with Et_2O (1 vol. = 5 mL). Crude product was washed with a NH_4Cl saturated solution, affording the product **18** (146 mg, 90% yield). Spectral data matched with the literature.[i]

12. Functionalization of bromomethyllactone 15 and its analogues

4-(Azidomethyl)-3-fluoro-2(5*H*)-furanone **(20)**

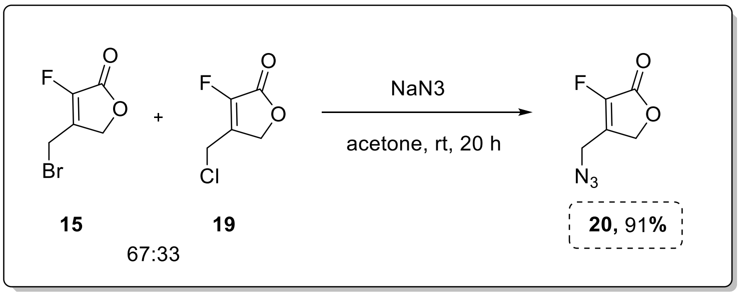

To a 67:33 mixture of bromide **15** (121 mg, 0.621 mmol, 0.67 equiv) and chloride **19** (47 mg, 0.31 mmol, 0.33 equiv) in acetone (5 mL) was added sodium azide (94 mg, 0.96 mmol, 1.03 equiv). After 20 h of stirring at rt, the reaction mixture was filtered over a pad of celite® then concentrated. Column chromatography (pentane/Et_2O 50:50) afforded 133 mg (0.847 mmol, 91%) of **20** as a colorless oil.

^{1}H NMR (500 MHz, $CDCl_3$) δ 4.84 (d $^4J_{HF}$ = 5.8 Hz, 2H, CH_2O), 4.36 (br s, 2H, CH_2N). ^{13}C NMR (126 MHz, $CDCl_3$) δ 164.1 (d, $^2J_{CF}$ = 30.7 Hz), 144.4 (d, $^1J_{CF}$ = 277.2 Hz), 132.0 (d, $^2J_{CF}$ = 5.6 Hz), 66.6 (d, $^3J_{CF}$ = 6.2 Hz), 44.0 (d, $^3J_{CF}$ = 2.8 Hz). ^{19}F NMR (471 MHz, $CDCl_3$) -145.05 – -145.11 (m, 1F).

Diethyl [(4-fluoro-2,5-dihydro-5-oxo-3-furanyl)methyl]-phosphonate **(21)**

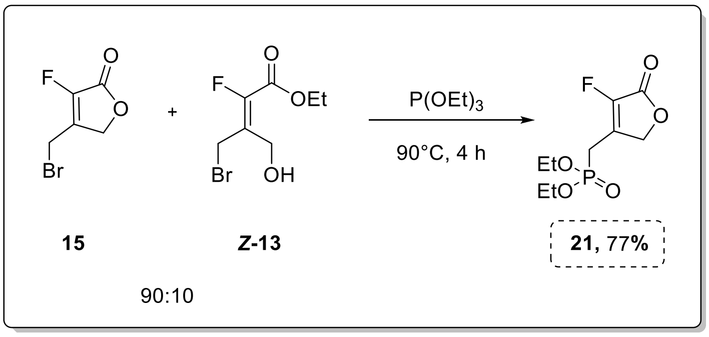

A 90:10 mixture of **15**/*Z*-**13** (230 mg, 1.18 mmol, 1 equiv) and triethyl phosphite (1.0 mL, 5.9 mmol, 5 equiv) was heated at 90 °C for 4 h and excess phosphite was evaporated under vacuum. Column chromatography (pentane/acetone 85:15 to 60:40) afforded 228 mg (0.905 mmol, 77%) of the phosphonate **21** as a colorless oil.

^{1}H NMR (600 MHz, $CDCl_3$) δ 4.85 (d $^4J_{HF}$ = 4.8 Hz, 2H, CH_2O), 4.19 – 4.08 (m, 4H, $OC\underline{H_2}CH_3$), 2.95 (d, $^2J_{HP}$ = 22.5 Hz, 2H, CH_2P), 1.32 (t, $^3J_{HH}$ = 7.1 Hz, 6H,

[i] A. Fort, D.; J. Woltering, T.; M. Alker, A.; Bach, T. *Heterocycles* **2014**, *88* (2), 1079. doi:10.3987/COM-13-S(S)67

$OCH_2C\underline{H_3}$). ^{13}C NMR (151 MHz, $CDCl_3$) δ 164.4 (dd, $^2J_{CF}$ = 30.4, $^4J_{CP}$ = 3.2 Hz), 145.1 (dd, $^1J_{CF}$ = 273.9, $^3J_{CP}$ = 11.5 Hz), 129.9 (dd, $^2J_{CP}$ = 10.9, $^2J_{CF}$ = 6.7 Hz), 67.9 (dd, $^3J_{CF}$ = 6.1, $^3J_{CP}$ = 2.0 Hz), 62.9 (d, $^2J_{CP}$ = 6.7 Hz, 2C), 23.3 (dd, $^1J_{CP}$ = 140.7 Hz, $^3J_{CF}$ = 2.6 Hz), 16.2 (d, $^3J_{CP}$ = 6.0 Hz, 2C). ^{19}F NMR (565 MHz, $CDCl_3$) -147.8 (br s, 1F). ^{31}P NMR (243 MHz, $CDCl_3$). 20.5 (br s, simplifies to d, $^4J_{PF}$ = 12.2 Hz upon ^1H decoupling, 1P).

Diethyl (*Z*)-[3-fluoro-4-hydroxy-2-(hydroxymethyl)-but-2-en-1-yl]-phosphonate **(22)**

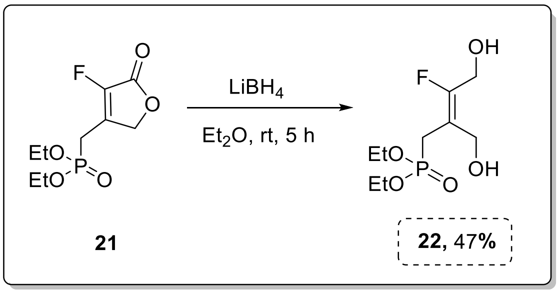

To a suspension of $LiBH_4$ (22 mg, 1.0 mmol, 2.2 equiv) in Et_2O (1.5 mL) at 0 ºC was added dropwise a solution of lactone **21** (115 mg, 0.456 mmol, 1 equiv) in Et_2O (1.5 mL). The reaction mixture was stirred at rt for 5 h then quenched with sat. aq. NH4Cl. The aqueous layer was extracted with EtOAc (3 × 15 then 2 × 25 mL). The combined organic layers were dried ($MgSO_4$), filtered, and concentrated. Column chromatography (CH_2Cl_2/MeOH 98:2 to 93:7) afforded 56 mg (0.222 mmol, 49%) of starting lactone **21** and 55 mg (0.215 mmol, 47%) of the desired diol **22** as colorless oil.

^1H NMR (500 MHz, $CDCl_3$) δ 4.47 (br s, 1H, OH), 4.27 (d $^3J_{HF}$ = 22.6 Hz, 2H, $CFCH_2O$), 4.20 (br s, 2H, CCH_2OH), 4.10 (dq appears as quint, $^3J_{HP}$ = $^3J_{HH}$ = 7.2 Hz, 4H, $OC\underline{H_2}CH_3$), 3.96 (br s, 1H, OH), 2.78 (d, $^2J_{HP}$ = 22.0 Hz, 2H, CH_2P), 1.32 (t, $^3J_{HH}$ = 7.1 Hz, 6H, $OCH_2C\underline{H_3}$). ^{13}C NMR (151 MHz, $CDCl_3$) δ 159.3 (dd, $^1J_{CF}$ = 260.4, $^3J_{CP}$ = 13.3 Hz), 111.0 (dd appears as t, $^2J_{CP}$ = $^2J_{CF}$ = 11.2 Hz), 62.7 (d, $^2J_{CP}$ = 6.8 Hz, 2C), 60.6 (dd, $^3J_{CF}$ = 7.5, $^3J_{CP}$ = 1.6 Hz), 57.4 (dd, $^2J_{CF}$ = 30.7, $^4J_{CP}$ = 2.6 Hz), 25.2 (dd, $^1J_{CP}$ = 139.2 Hz, $^3J_{CF}$ = 6.9 Hz), 16.3 (d, $^3J_{CP}$ = 6.1 Hz, 2C). ^{19}F NMR (471 MHz, $CDCl_3$) -107.9 – -108.2 (m, 1F). ^{31}P NMR (202 MHz, $CDCl_3$). 27.6 (br s, 1P).

13. Preparation of tetrasubstituted fluoroalkenes

[(*E*)-4-Benzyloxy-3-fluoro-2-(hydroxymethyl)but-2-enyl] acetate **(26)**

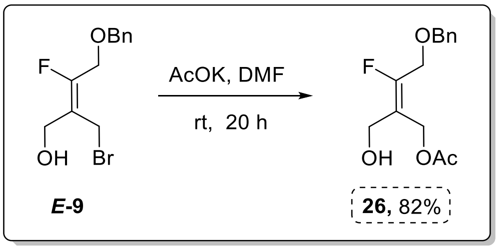

To a solution of *E*-**9** (0.200 g, 0.692 mmol) in DMF (4 mL) was added KOAc (149 mg, 1.522 mmol). The reaction mixture was stirred during 16 hours then DMF was removed in vacuo. CH_2Cl_2 (20 mL) and water (20 mL) were added to the residue. The layers were separated and the aqueous layer was extracted with CH_2Cl_2. The organic phase was washed with brine, dried with $MgSO_4$, filtered, and concentrated in vacuo. The crude product was purified by column chromatography using pentane/AcOEt (80/20) as eluent. Compound **26** (0.152 g, 0.567 mmol) was obtained as colorless oil with 82% yield.

^1H NMR (500 MHz, $CDCl_3$) δ 7.39 – 7.12 (m, 5H), 4.66 (s, 2H), 4.51 (s, 2H), 4.24 (s, 2H), 4.17 (d, $^3J_{HF}$ = 22.0 Hz, 2H), 2.37 (s, 1H), 1.99 (s, 3H).^{19}F NMR (471 MHz, $CDCl_3$) δ -106.28 (t, $^3J_{FH}$ = 22.0 Hz).^{13}C NMR (151 MHz, $CDCl_3$) δ 171.34, 157.22 (d, $^1J_{CF}$ = 261.3 Hz), 137.36, 128.55 (2C), 128.04, 127.90 (2C), 117.27 (d, $^2J_{CF}$ = 14.7 Hz), 72.72, 64.61 (d, $^2J_{CF}$ = 29.7 Hz), 59.66 (d, $^3J_{CF}$ = 9.8 Hz), 56.43 (d, $^3J_{CF}$ = 9.4 Hz), 20.92. HRMS-ESI (m/z) $[M+Na]^+$ calculated for $C_{14}H_{17}O_4FNa^+$ 291.1009, found 291.1001.

[(*E*)-2-(Azidomethyl)-4-benzyloxy-3-fluoro-but-2-enyl] acetate **(28)**

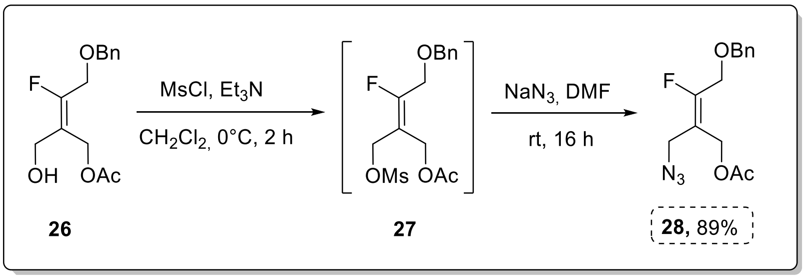

To a solution of compound **26** (0.200 g, 0.745 mmol) in CH_2Cl_2 (5 mL) was added MsCl (144 µL, 1.86 mmol) and Et_3N (258 µL, 1.86 mmol) at 0 °C. Reaction mixture was stirred during 2 h, then NH_4Cl (5 mL) was added. Medium was extracted with Et_2O, then washed with saturated $NaHCO_3$ aqueous solution, and dried on $MgSO_4$. After filtration and concentration in vacuo, compound **27** was dissolved in DMF (4 mL), and sodium azide (0.120 g, 1.84 mmol) was added to the medium. After 16 h of stirring, DMF was evaporated and the mixture was dissolved in water and CH_2Cl_2. Layers were separated then aqueous layer was extracted with CH_2Cl_2. Organic phase was washed with brine, dried with $MgSO_4$, filtered, and concentrated in vacuo. The crude product was purified by column chromatography using pentane/AcOEt (70/30) as eluent. Compound **28** (0.192 g, 0.518 mmol) was obtained with 89% yield.

^{1}H NMR (500 MHz, $CDCl_3$) δ 7.39 – 7.29 (m, 5H), 4.67 (d, $^{4}J_{HF}$ = 1.7 Hz, 2H), 4.58 (s, 2H), 4.28 (d, $^{3}J_{HF}$ = 21.4 Hz, 2H), 4.00 (d, $^{4}J_{HF}$ = 3.0 Hz, 2H), 2.06 (s, 3H). ^{19}F NMR (471 MHz, $CDCl_3$) δ -102.72 (t, $^{3}J_{FH}$ = 21.4 Hz). ^{13}C NMR (126 MHz, $CDCl_3$) δ 170.72, 159.21 (d, $^{1}J_{CF}$ = 264.6 Hz), 137.23, 128.65 (2C), 128.17, 128.01 (2C), 112.68 (d, $^{2}J_{CF}$ = 14.9 Hz), 72.79, 64.41 (d, $^{2}J_{CF}$ = 29.5 Hz), 60.14 (d, $^{3}J_{CF}$ = 9.2 Hz), 46.57 (d, $^{3}J_{CF}$ = 8.1 Hz), 20.91. HRMS-ESI (m/z) $[M+Na]^+$ calculated for $C_{14}H_{16}N_3O_3FNa^+$ 316.1073, found 316.1064.

[(*Z*)-4-Benzyloxy-2-(diethoxyphosphorylmethyl)-3-fluoro-but-2-enyl] acetate **(29)**

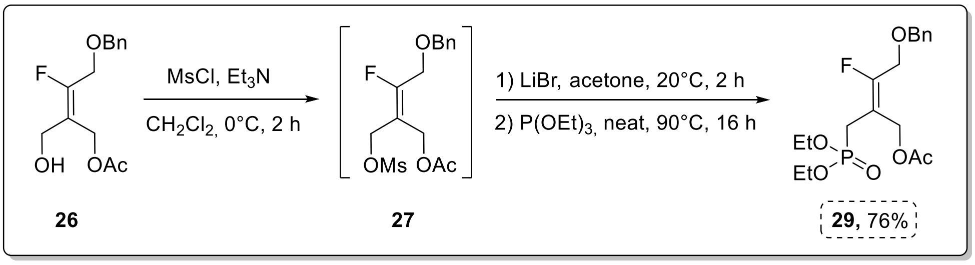

To a solution of compound **26** (0.077 g, 0.289 mmol) in CH_2Cl_2 (5 mL) was added MsCl (55 µL, 0.723 mmol) and Et_3N (100 µL, 1.86 mmol) at 0 °C. The reaction mixture was stirred during 2 h, then NH_4Cl (5 mL) was added. The medium was extracted with Et_2O, then washed with saturated $NaHCO_3$ aqueous solution, and dried on $MgSO_4$. After filtration and concentration in vacuo, compound **27** was dissolved in acetone (4 mL) and LiBr (0.125 g, 1.445 mmol) was added to the medium. After 3 h of stirring, the medium was filtered and concentrated. The mixture was dissolved in $P(OEt)_3$ (5 mL, 29 mmol) and heated at 90 °C for 16 h. After concentration in vacuo, the crude product was purified by column chromatography using pentane/AcOEt (50:50) as eluent. Compound **29** (0.086 g, 0.221 mmol) was obtained with 76% yield.

^{1}H NMR (500 MHz, $CDCl_3$) δ 7.37 – 7.21 (m, 5H), 4.72 – 4.69 (br t, $^{4}J_{HH}$ =2.9 Hz, 2H), 4.54 (s, 2H), 4.24 (dd, $^{3}J_{HF}$ = 21.9 Hz, $^{4}J_{HH}$ =2.9 Hz, 2H), 4.15 – 4.06 (m, 4H), 2.78 (dd, $^{2}J_{HP}$ = 22.0 Hz, $^{4}J_{HF}$ = 2.7 Hz, 2H), 2.03 (s, 3H), 1.31 (t, $^{3}J_{HH}$ = 7.1 Hz, 6H). ^{19}F NMR (471 MHz, $CDCl_3$) δ -103.26 (td, $^{3}J_{FH}$ = 21.9 Hz, $^{4}J_{FP}$ = 11.8 Hz). ^{13}C NMR (151 MHz, $CDCl_3$) δ 170.74, 157.84 (dd, $^{1}J_{CF}$ = 261.2, $^{3}J_{CP}$ =12.8 Hz), 137.46, 128.57 (2C), 128.03, 127.89 (2C), 109.86 (dd, $^{2}J_{CF}$ =16.3 Hz , $^{2}J_{CP}$ = 11.4 Hz), 72.27, 64.24 (dd, $^{2}J_{CF}$ = 29.0, $^{4}J_{CP}$ = 2.5 Hz), 62.23 (d, $^{2}J_{CP}$ = 6.6 Hz, 2C), 61.40 (dd, $^{3}J_{CF}$ = 9.4, $^{3}J_{CP}$ = 1.5 Hz), 24.29 (dd, $^{1}J_{CP}$ =139.5, $^{3}J_{CF}$ =6.4 Hz), 20.95, 16.46 (d, $^{3}J_{CP}$ = 6.2 Hz, 2C). ^{31}P NMR (243 MHz, $CDCl_3$) δ 25.31 (td, $^{2}J_{PH}$ = 22.0 Hz, $^{4}J_{PF}$ =11.8 Hz). HRMS-ESI (m/z) $[M+H]^+$ calculated for $C_{18}H_{27}O_6FP^+$ 389.1529, found 389.1532.

14. Structure determination

a) X-Ray structures for compound E-1d, E-4d and Z-25

X-ray analysis of compound *E*-1d (Deposition Number 2005891)

```
Bond precision:   C-C = 0.0032 A          Wavelength=0.71073

Cell:              a=22.9860(8)      b=13.5062(4)      c=8.2942(3)
                   alpha=90          beta=90           gamma=90
Temperature:       291 K

                Calculated                 Reported
Volume          2574.96(15)                2574.96(15)
Space group     P b c n                    P b c n
Hall group      -P 2n 2ab                  -P 2n 2ab
Moiety formula  C13 H11 Br F N O3          C13 H11 Br F N O3
Sum formula     C13 H11 Br F N O3          C13 H11 Br F N O3
Mr              328.13                     328.14
Dx,g cm-3       1.693                      1.693
Z               8                          8
Mu (mm-1)       3.208                      3.208
F000            1312.0                     1312.0
F000'           1310.41
h,k,lmax        32,19,11                   32,19,11
Nref            3938                       3928
Tmin,Tmax       0.410,0.478
Tmin'           0.379

Correction method= Not given

Data completeness= 0.997          Theta(max)= 30.522

R(reflections)= 0.0371( 2704)     wR2(reflections)= 0.0993( 3928)

S = 1.029                  Npar= 176
```

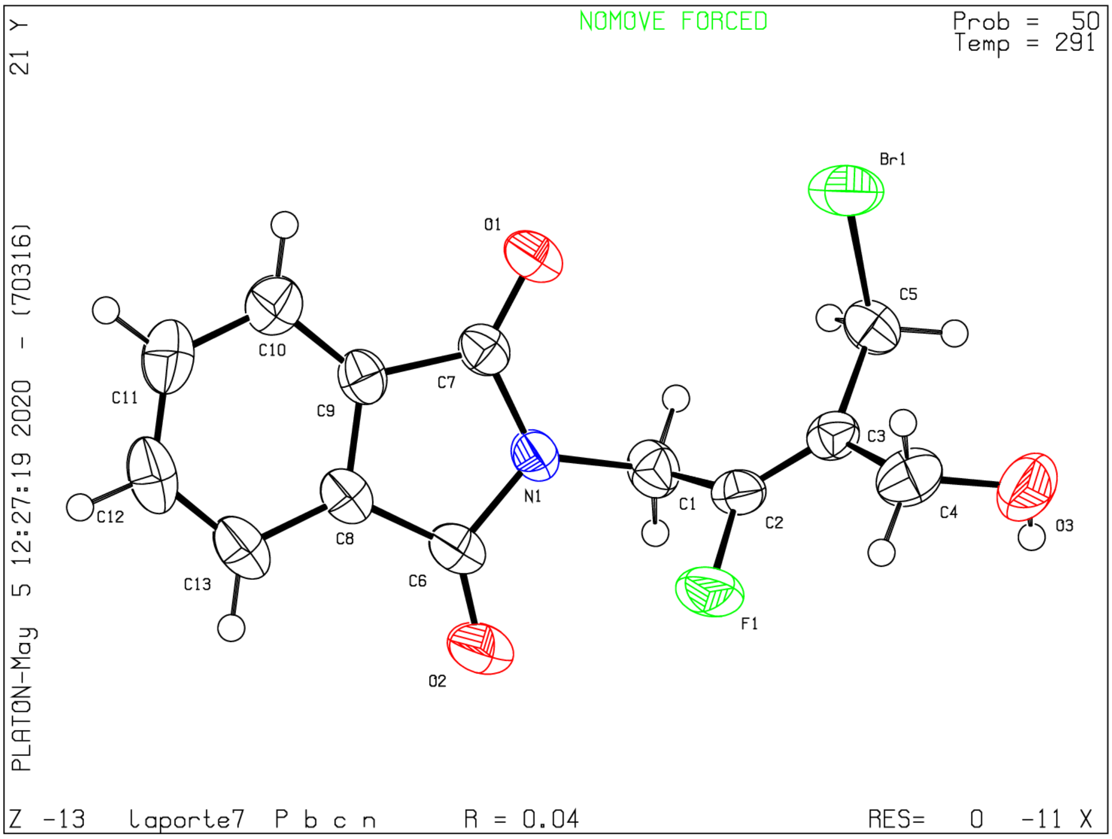
NOMOVE FORCED
Prob = 50
Temp = 291
21 Y
PLATON-May 5 12:27:19 2020 - (70316)
Br1
O1
C5
C10
C7
C11
C9
C3
N1
C1
C2
C4
O3
C12
C8
C6
C13
F1
O2
Z -13 Laporte7 P b c n R = 0.04 RES= 0 -11 X

X-ray analysis of compound *E*-4d (Deposition Number 2005890)

```
Bond precision:   C-C = 0.0029 A          Wavelength=0.71073

Cell:             a=28.7619(18)     b=11.7589(7)        c=7.2979(5)
                  alpha=90          beta=101.821(4)     gamma=90
Temperature:      150 K

                  Calculated                  Reported
Volume            2415.9(3)                   2415.9(3)
Space group       C 2/c                       C 2/c
Hall group        -C 2yc                      -C 2yc
Moiety formula    C13 H12 Br N O3             C13 H12 Br N O3
Sum formula       C13 H12 Br N O3             C13 H12 Br N O3
Mr                310.14                      310.15
Dx,g cm-3         1.705                       1.705
Z                 8                           8
Mu (mm-1)         3.402                       3.402
F000              1248.0                      1248.0
F000'             1246.29
h,k,lmax          42,17,10                    42,17,10
Nref              4138                        4091
Tmin,Tmax         0.388,0.600                 0.635,0.738
Tmin'             0.359

Correction method= # Reported T Limits: Tmin=0.635 Tmax=0.738
AbsCorr = MULTI-SCAN

Data completeness= 0.989           Theta(max)= 31.794

R(reflections)= 0.0379( 3253)      wR2(reflections)= 0.0924( 4091)

S = 1.030                  Npar= 164
```

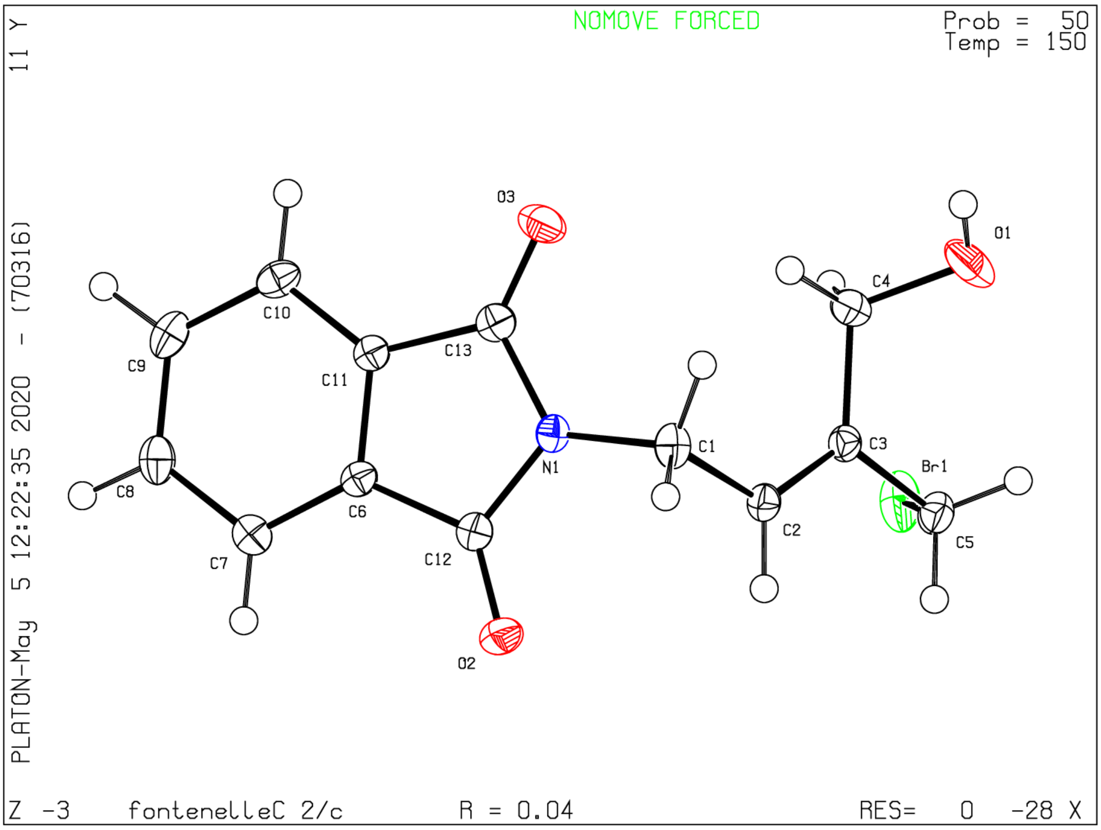
NOMOVE FORCED
Prob = 50
Temp = 150
11 Y
PLATON-May 5 12:22:35 2020 - (70316)
O3
O1
C4
C10
C9
C13
C11
N1
C1
C3
Br1
C8
C6
C2
C5
C7
C12
O2
Z -3 fontenelleC 2/c R = 0.04 RES= 0 -28 X

X-ray analysis of compound *Z*-25 (Deposition Number 2005889)

Bond precision: C-C = 0.0019 A Wavelength=0.71073

Cell:	a=7.3172(4)	b=8.3142(5)	c=13.7228(8)
	alpha=89.474(3)	beta=74.891(3)	gamma=80.312(3)
Temperature:	291 K		

	Calculated	Reported
Volume	793.97(8)	793.97(8)
Space group	P -1	P -1
Hall group	-P 1	-P 1
Moiety formula	C17 H19 F N2 O3	?
Sum formula	C17 H19 F N2 O3	C17 H19 F N2 O3
Mr	318.34	318.34
Dx,g cm-3	1.332	1.332
Z	2	2
Mu (mm-1)	0.100	0.100
F000	336.0	336.0
F000’	336.18	
h,k,lmax	10,12,19	10,12,19
Nref	5058	4981
Tmin,Tmax		
Tmin’		

Correction method= Not given

Data completeness= 0.985 Theta(max)= 30.998

R(reflections)= 0.0492(3841) wR2(reflections)= 0.1624(4981)

S = 1.036 Npar= 209

[redacted]

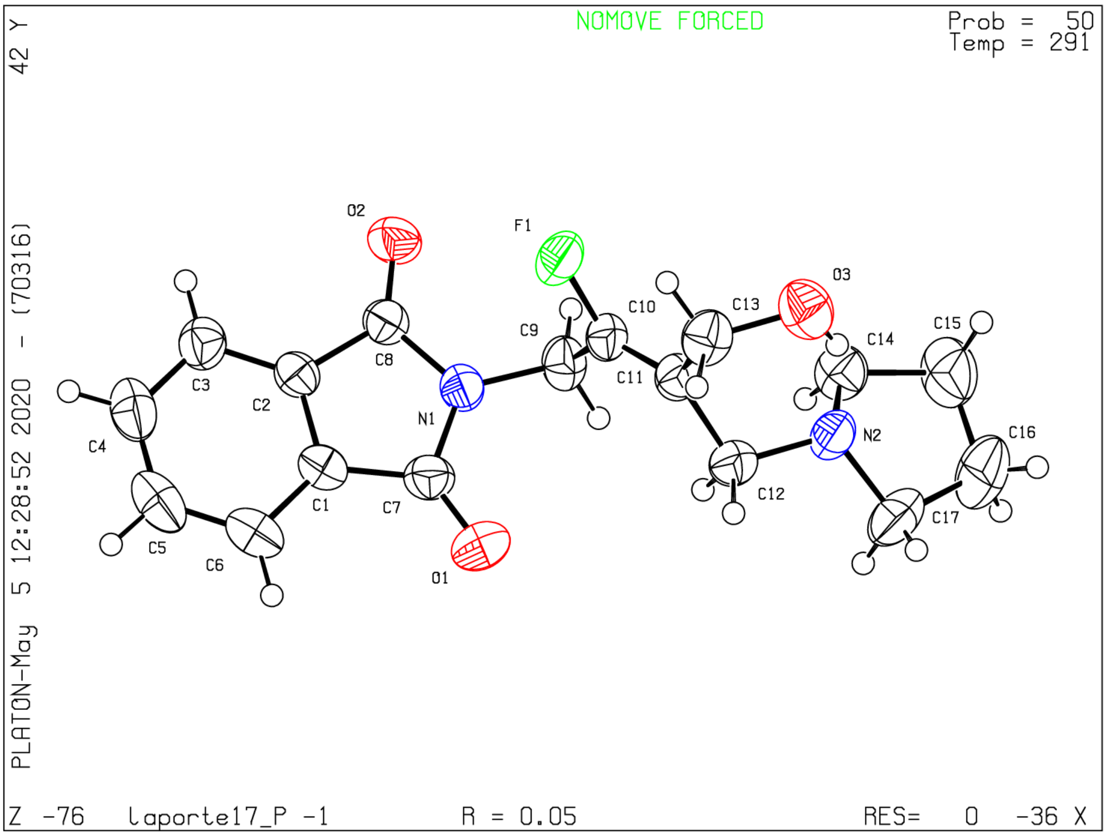
NOMOVE FORCED
Prob = 50
Temp = 291
42 Y
PLATON-May 5 12:28:52 2020 - (70316)
Z -76 Laporte17_P -1 R = 0.05 RES= 0 -36 X
O2
F1
O3
C13
C15
C14
C10
C9
C8
C3
C2
C11
N1
N2
C16
C4
C12
C1
C7
C17
C5
C6
O1

b) NOESY and HOESY experiments for compound E-3d, E-5d and E-2d

Starting from the alkene **3**, the selective oxetane opening reaction led to the formation of the *E*-isomer **3d** determined by 1D-^{1}H-NOESY and 2D-^{1}H-^{19}F-HOESY experiment. Irradiation of the green proton gave response for both red and blue protons (Figure S1-S2), and HOESY experiment revealed correlations between the fluorine atom and the yellow protons, confirming the *E*-stereochemistry of the alkene.

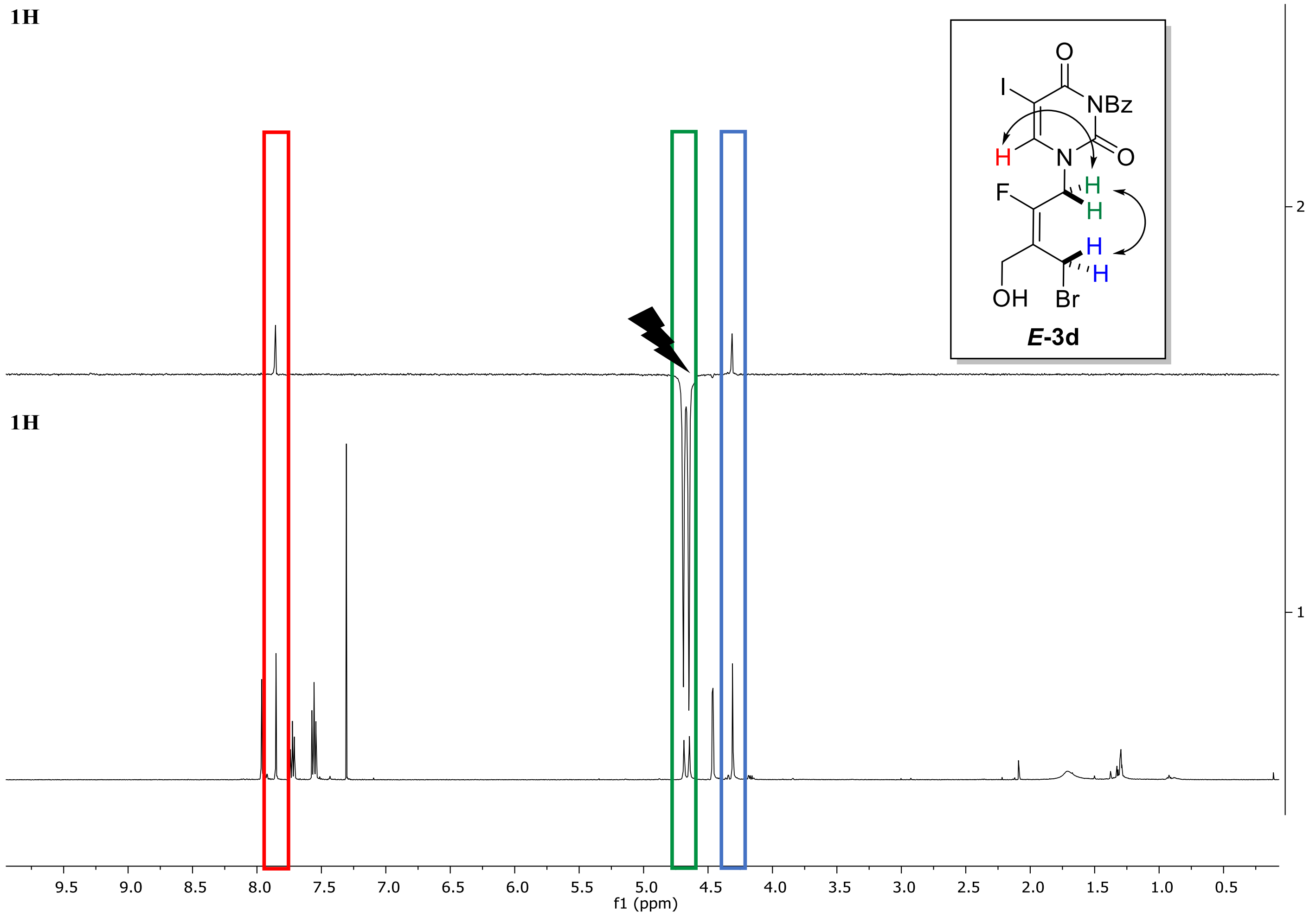

Figure S1. 1D-NOESY experiment for the compound **3d**

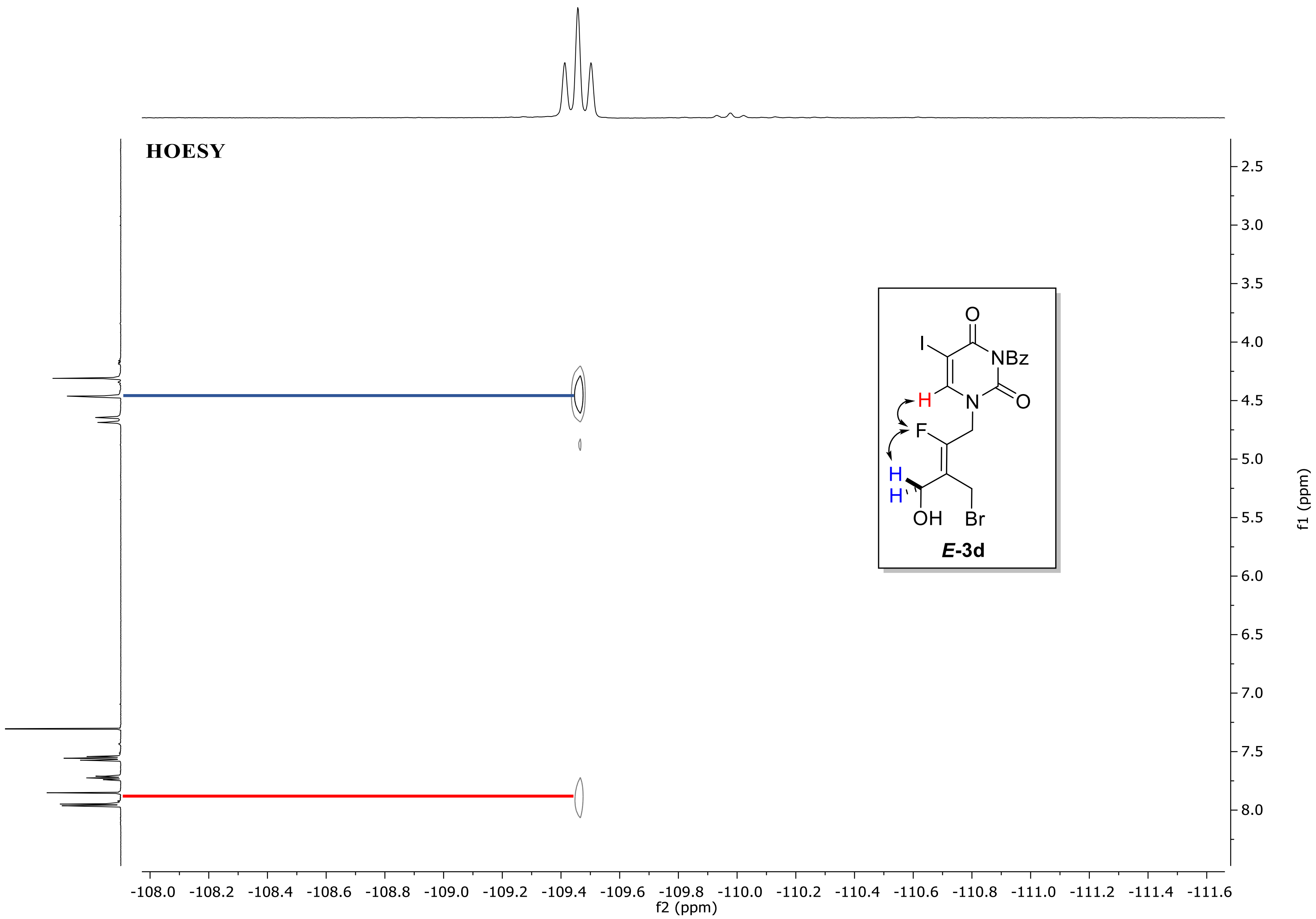

Figure S2. 2D-^{1}H (vertical)- ^{19}F (horizontal)-HOESY experiment for the compound **3d**

Starting from the alkene **5**, the selective oxetane opening reaction following led to the formation of the *E*-isomer **5d** determined by 1D-^{1}H-NOESY experiment (Figure S3). Irradiation of the green proton (line 1) gave response for both red and yellow protons, while irradiation on the red protons (line 2) gave response for the purple and green protons, confirming the *E*-stereochemistry of the alkene.

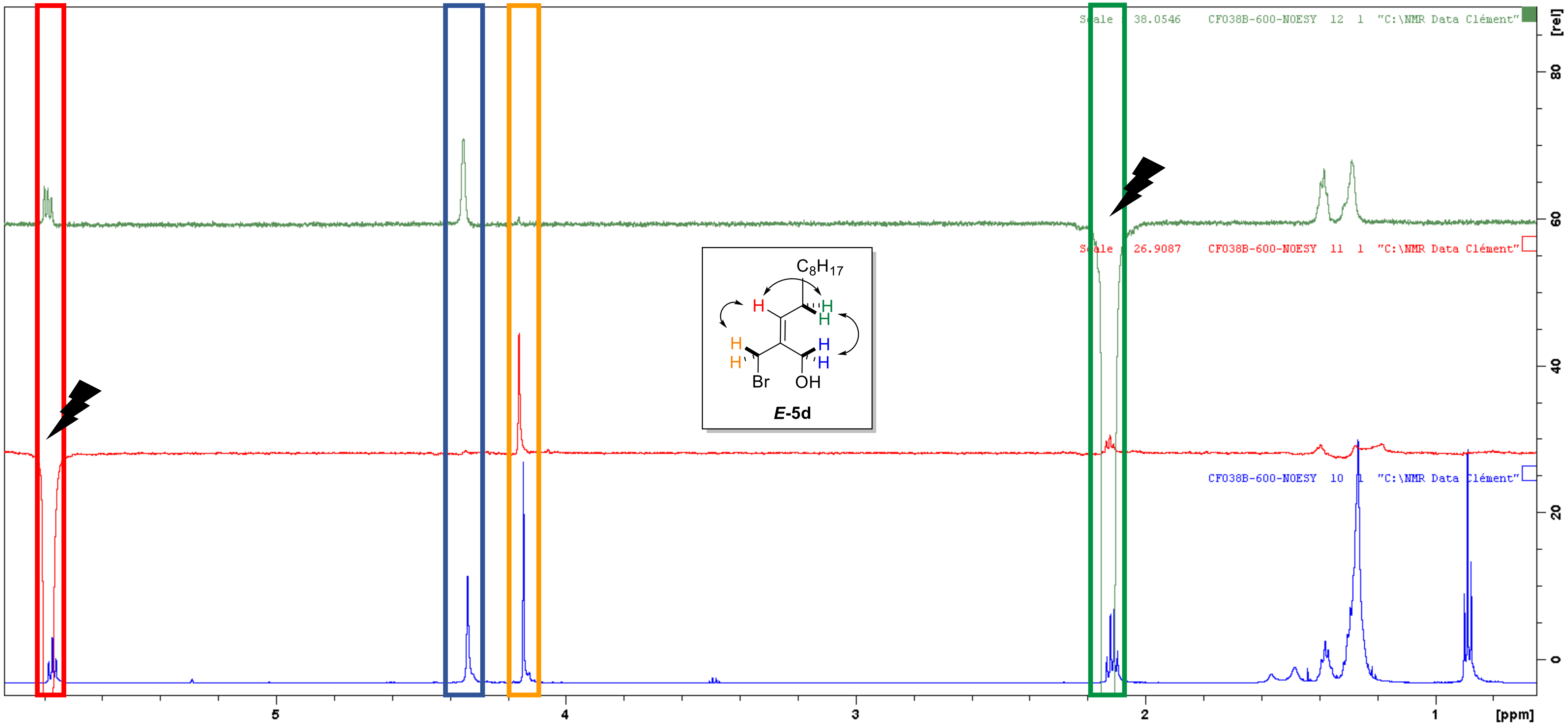

Figure S3: NOESY experiments for the compound **5d**

Starting from the alkene **2**, the selective oxetane opening reaction following led to the formation of the *E*-isomer **2d** determined by 1D-^{1}H-NOESY experiment (Figure S4). Irradiation of the red protons (line 1) gave response for green protons and no response for the blues protons, confirming the *E*-stereochemistry of the alkene.

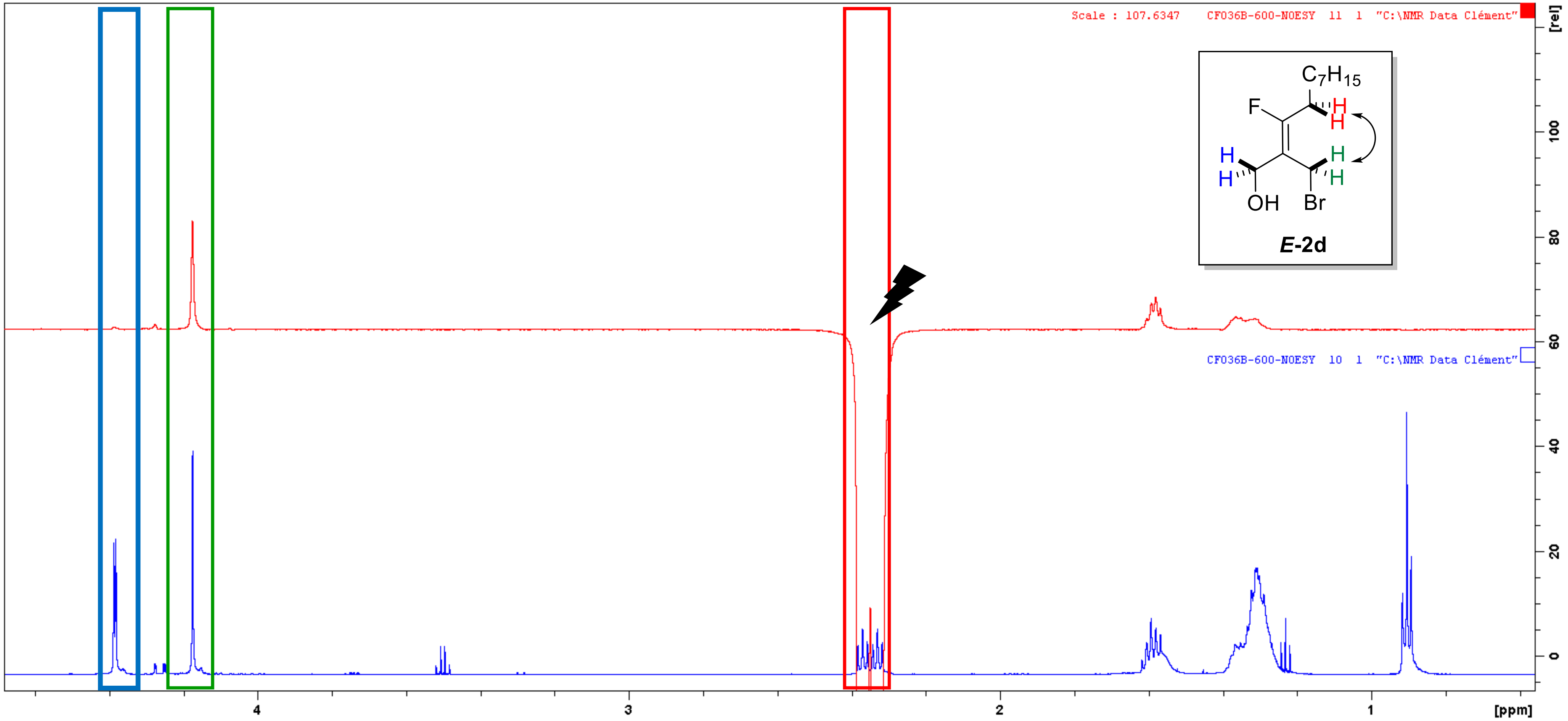

Figure S4: NOESY experiment for the compound **2d**

^{1}H, ^{13}C, ^{19}F and ^{31}P NMR for new compounds

N-[2-Fluoro-2-(oxetan-3-ylidene)ethyl] phthalimide **(1)**

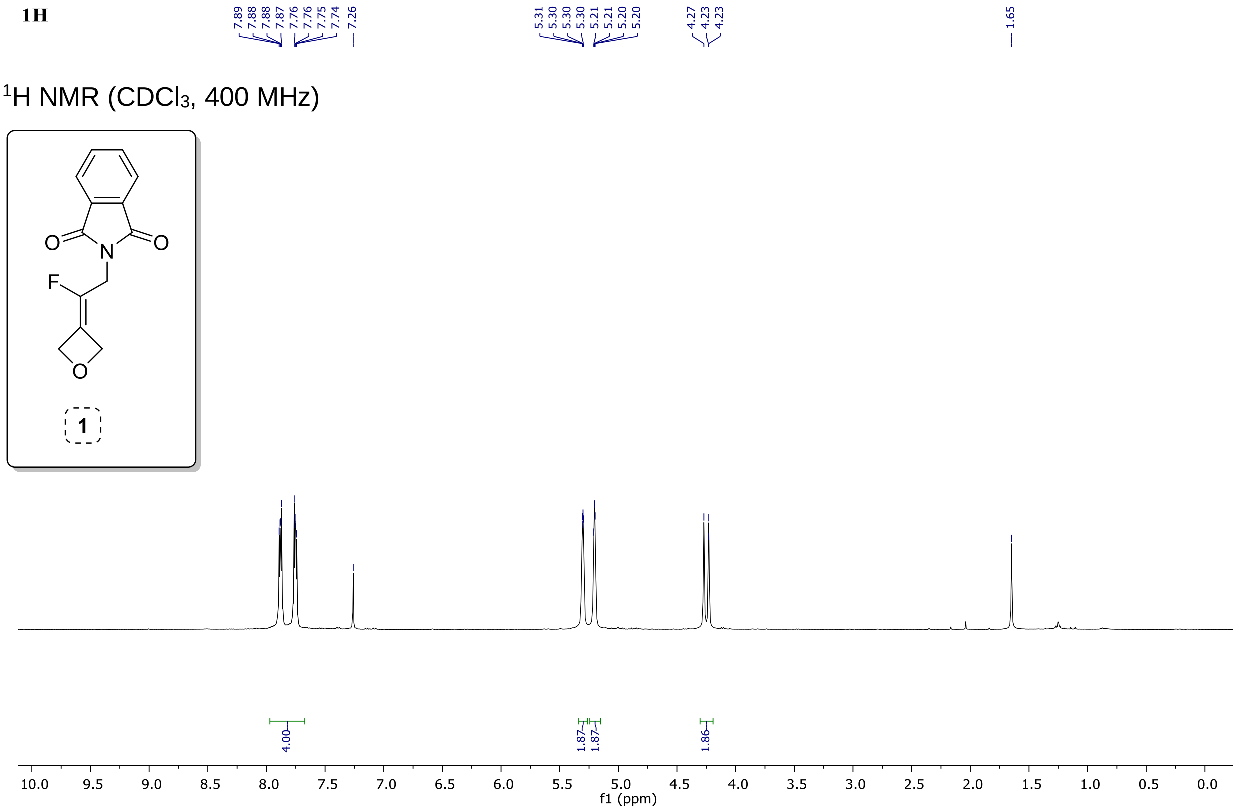

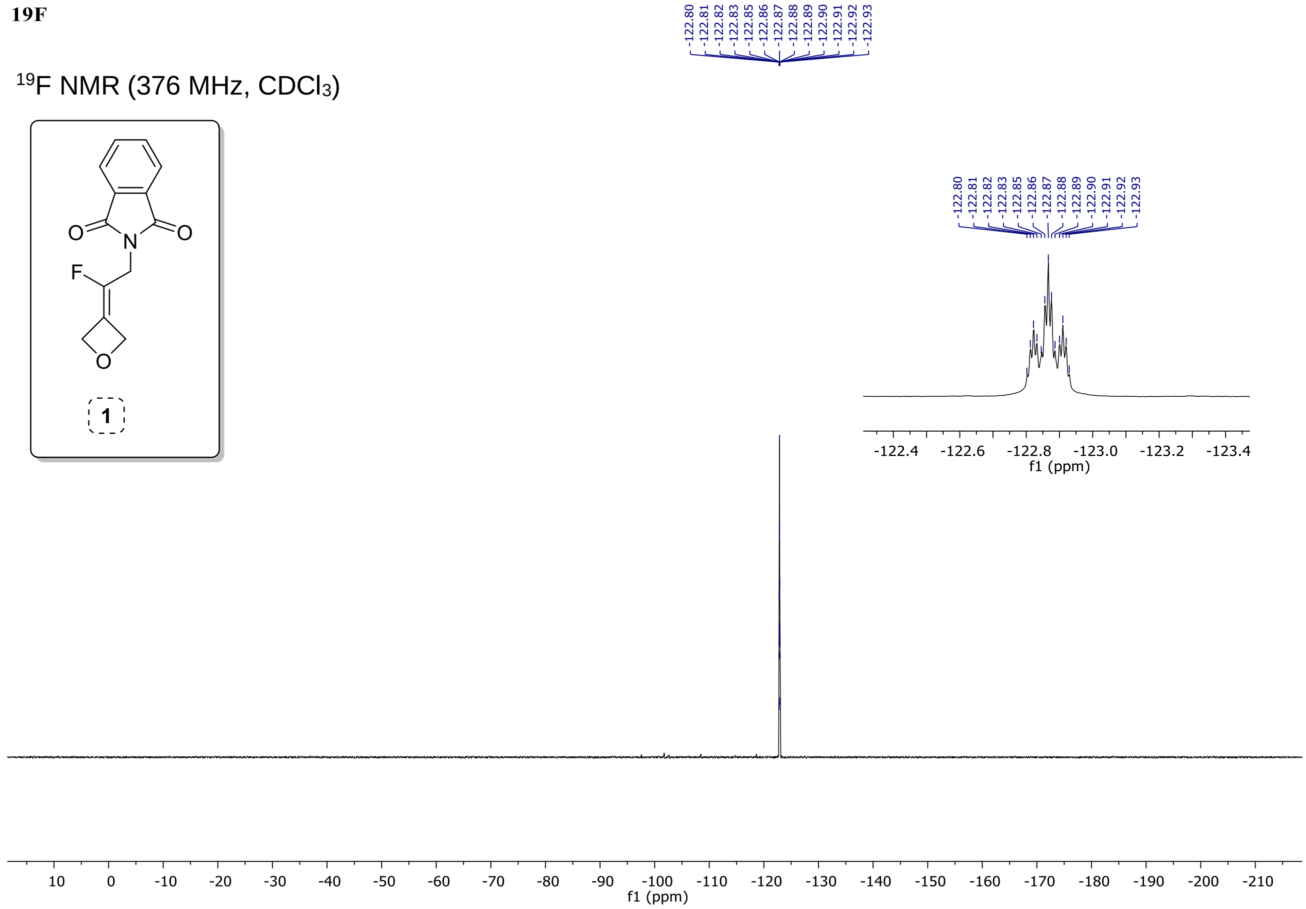

N-[2-Fluoro-3-(hydroxymethyl)-4-methoxy-but-2-enyl] phthalimide **(1a)**

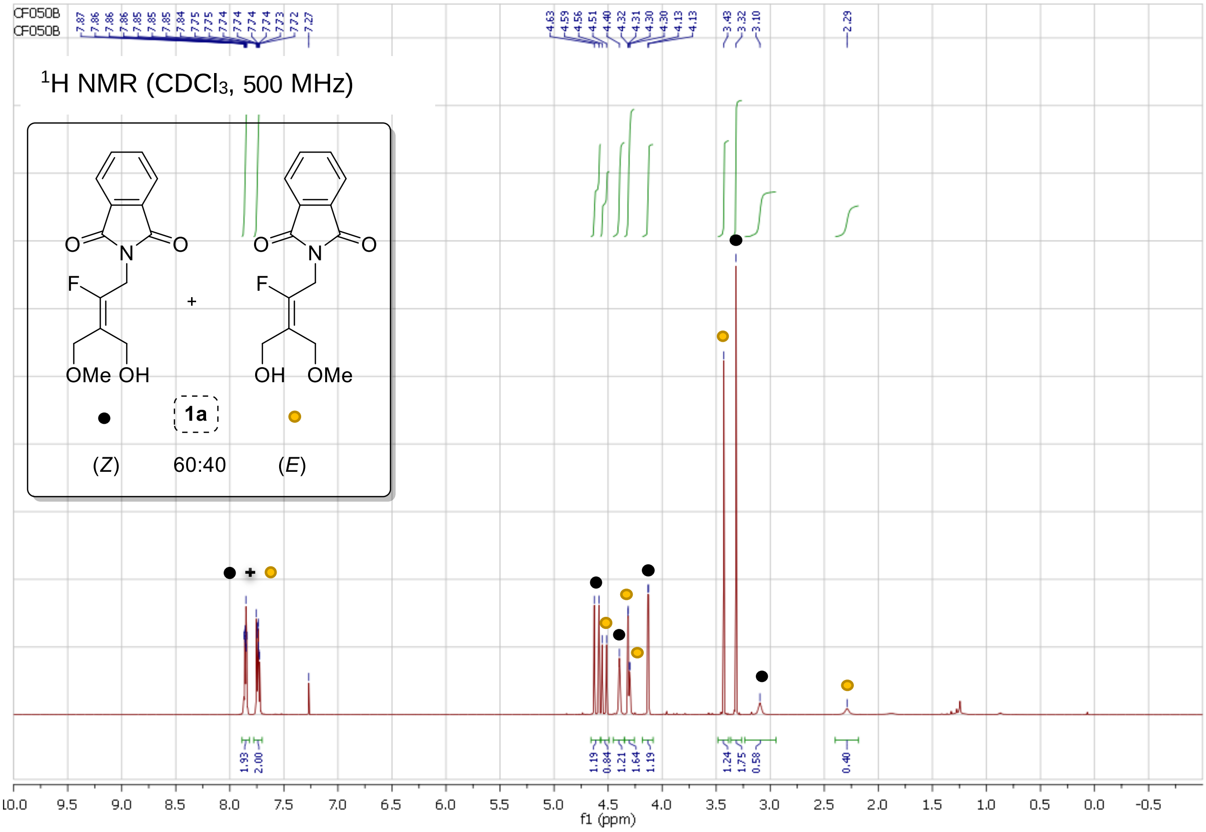

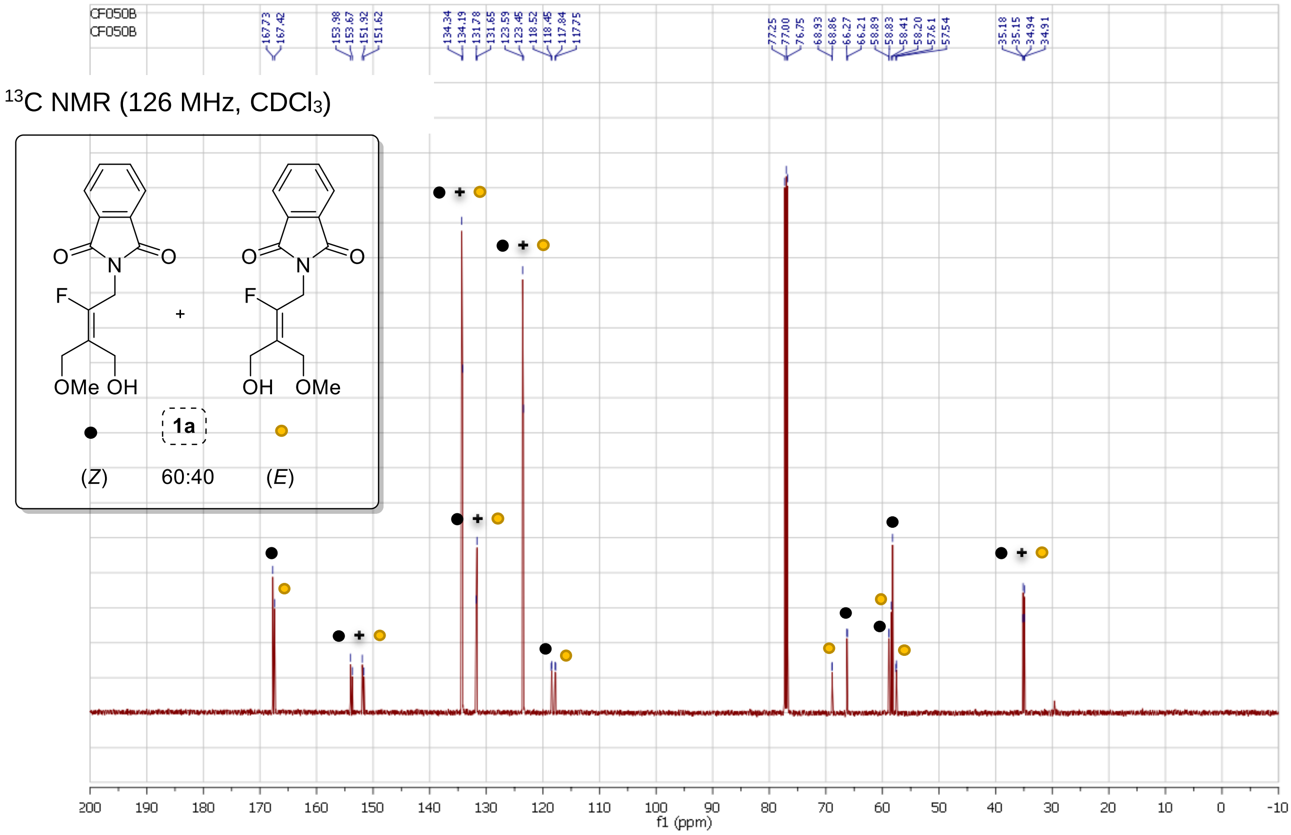

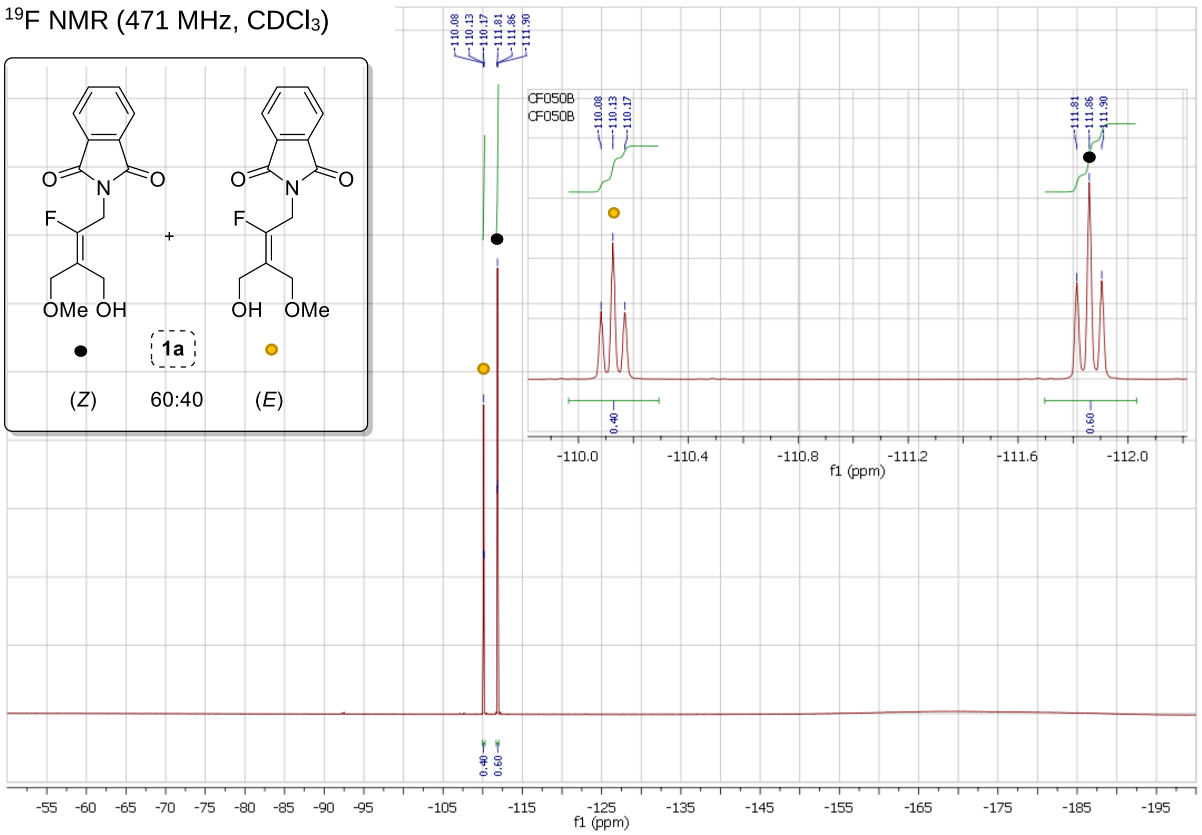

N-[3-(Benzyloxymethyl)-2-fluoro-4-hydroxy-but-2-enyl] phthalimide **(1b)**

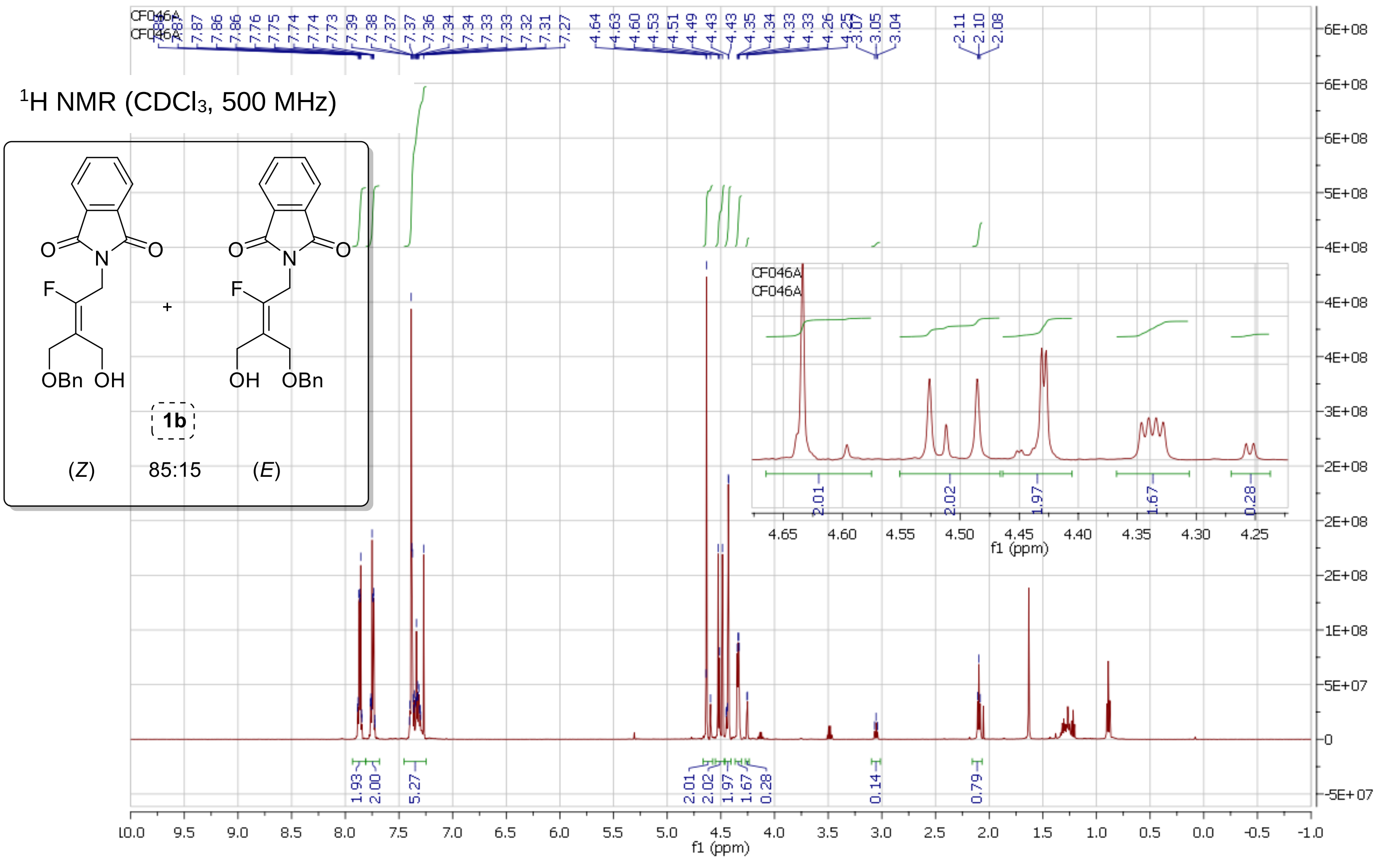

^{13}C NMR (126 MHz, $CDCl_3$)

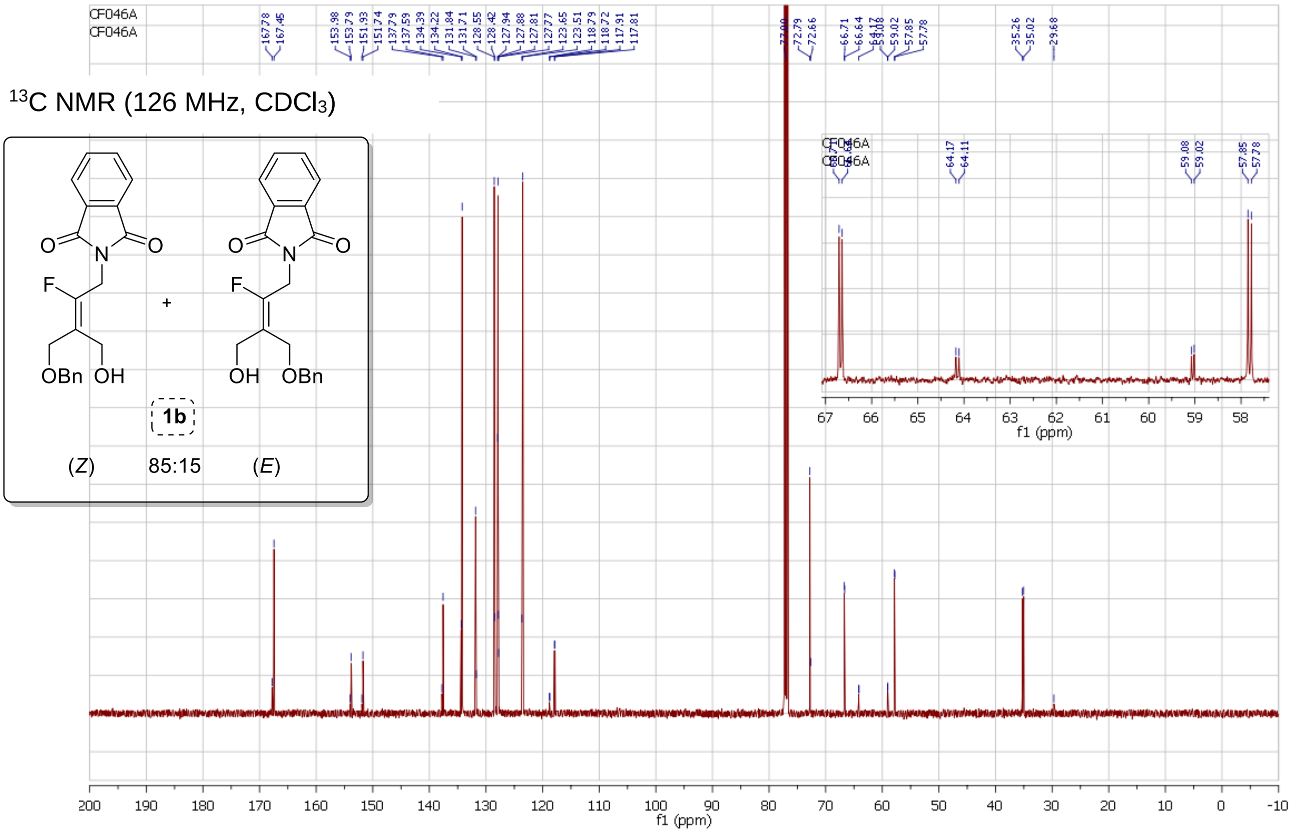

^{19}F NMR (471 MHz, $CDCl_3$)

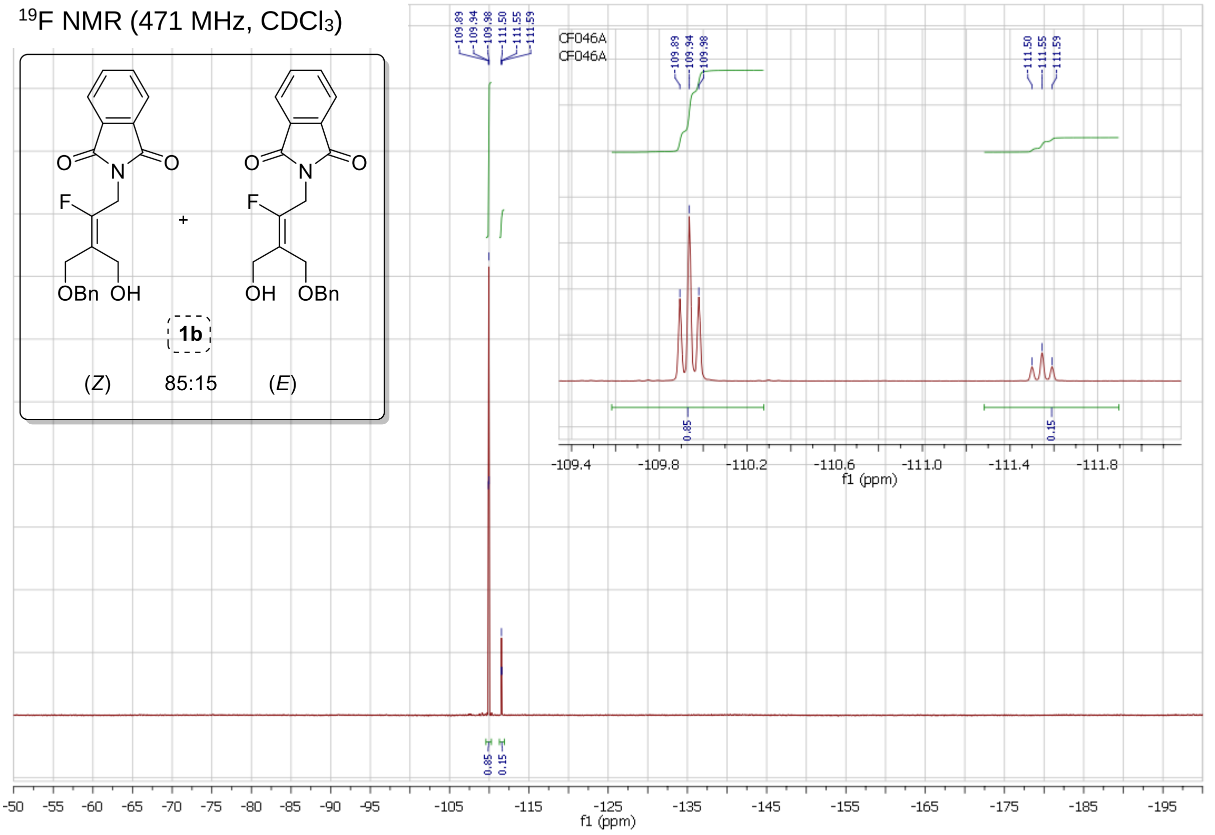

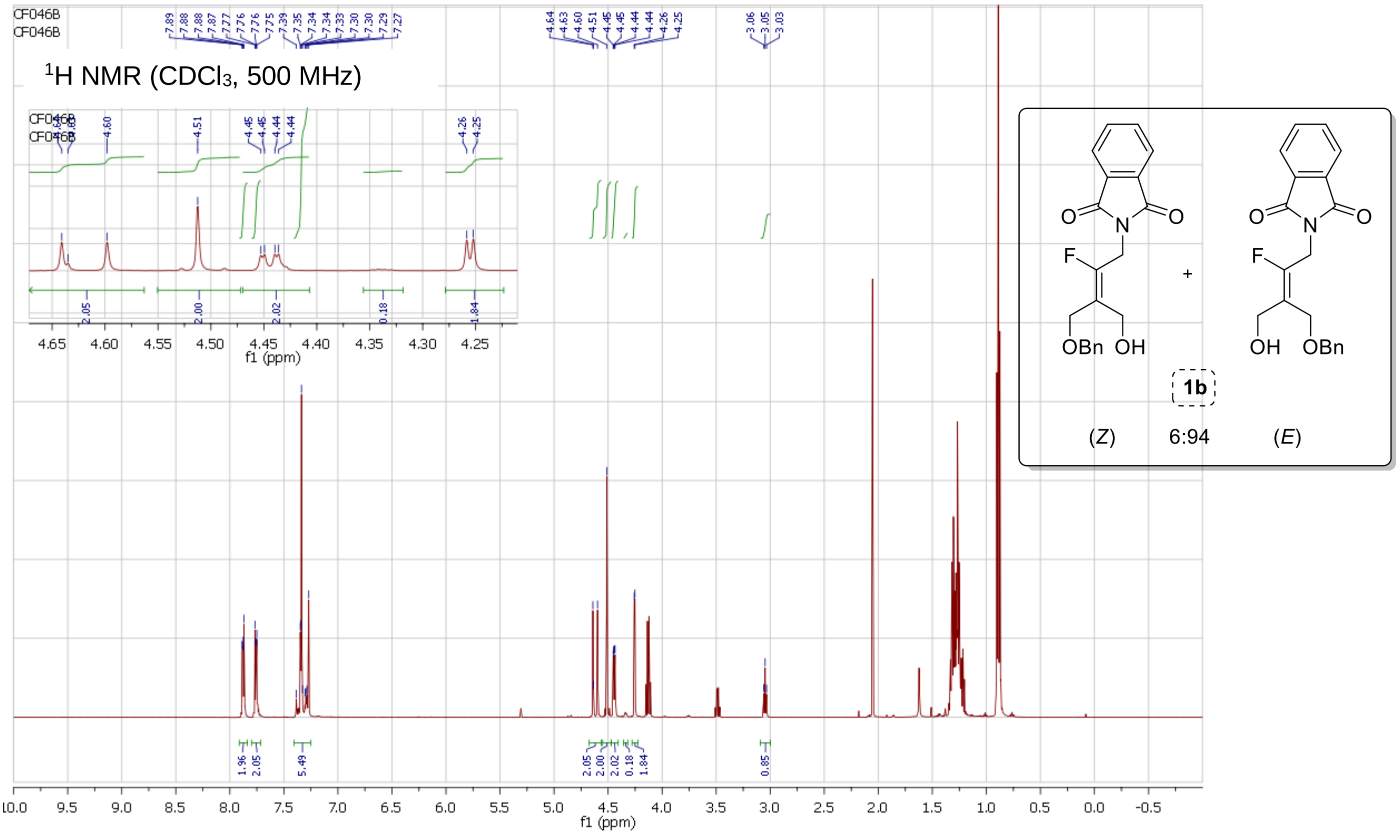

1H NMR (CDCl3, 500 MHz)
OBn OH
OH OBn
1b
(Z) 6:94 (E)
f1 (ppm)

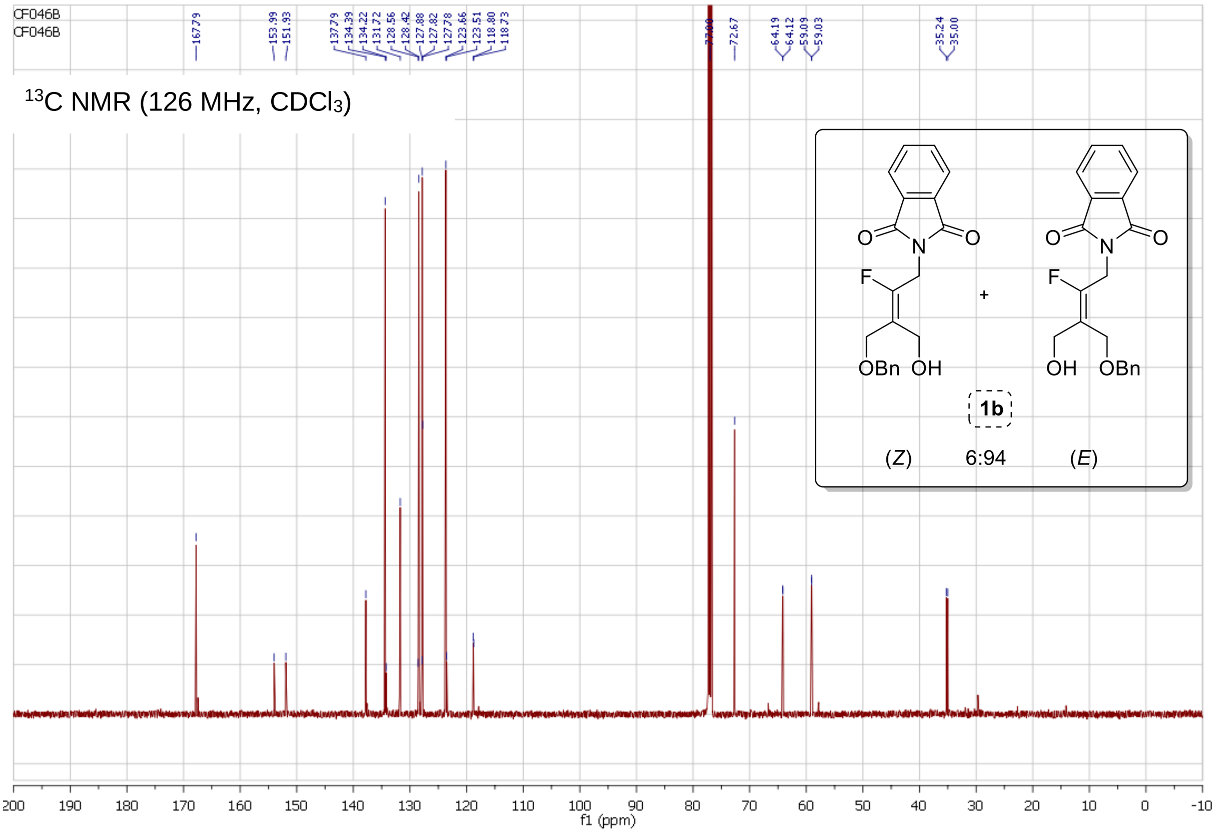

13C NMR (126 MHz, CDCl3)
OBn OH
OH OBn
1b
(Z) 6:94 (E)
f1 (ppm)

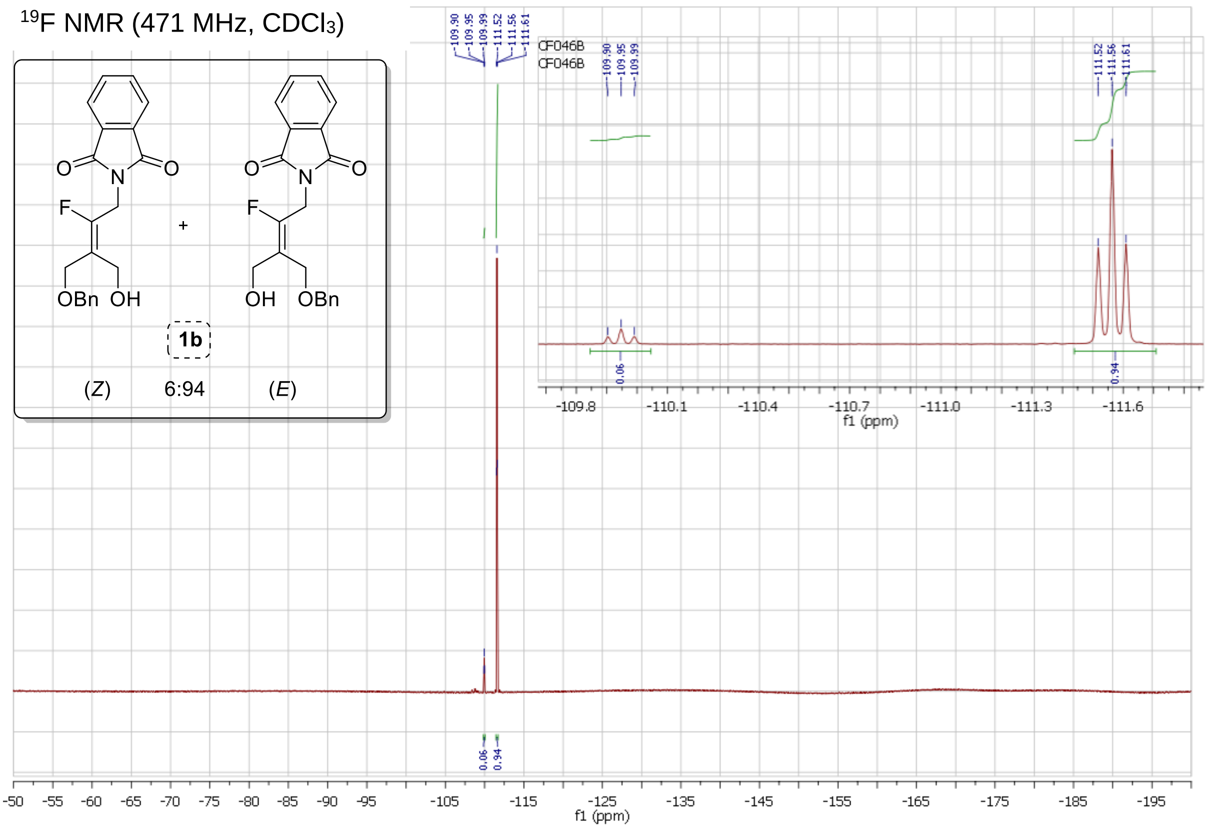

N-[(*Z*)-3-(1,3-Benzothiazol-2-ylsulfanylmethyl)-2-fluoro-4-hydroxy-but-2-enyl] phthalimide **(*Z*-1c)**

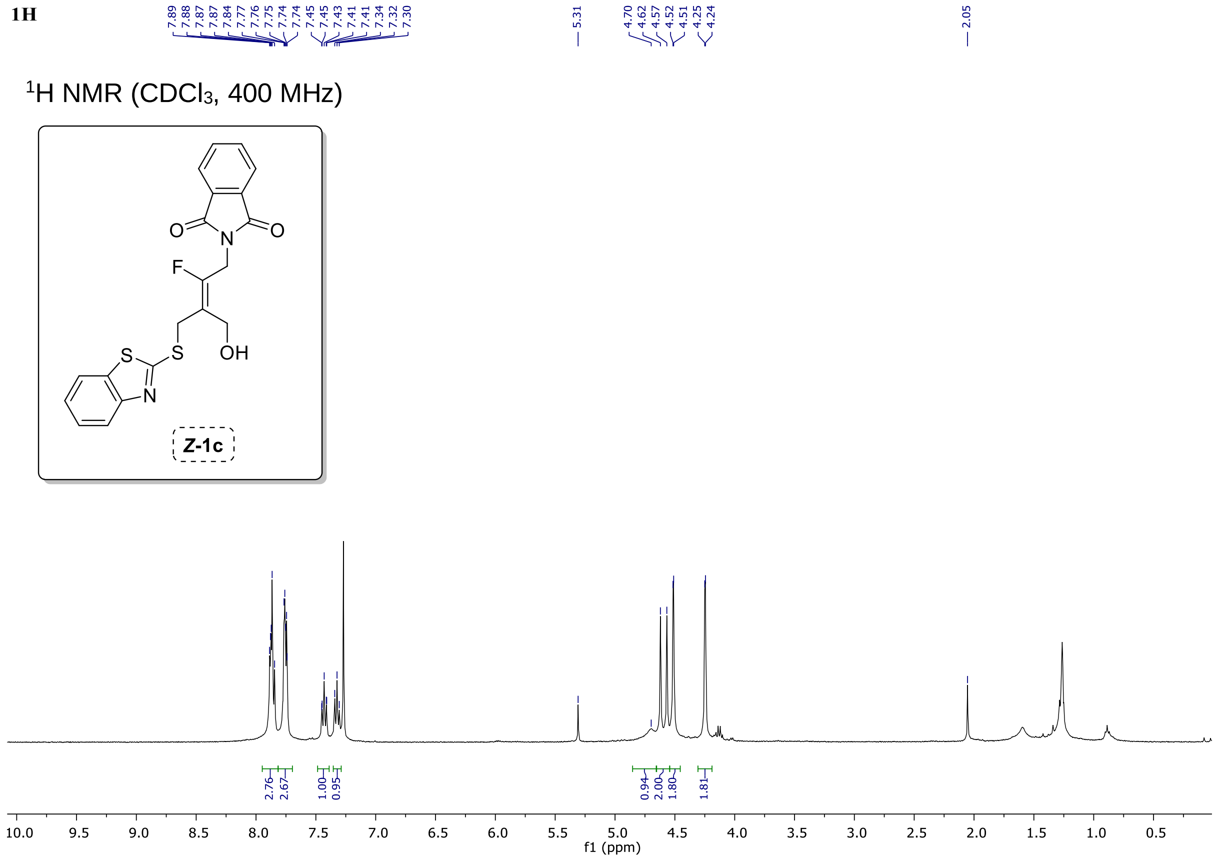

13C

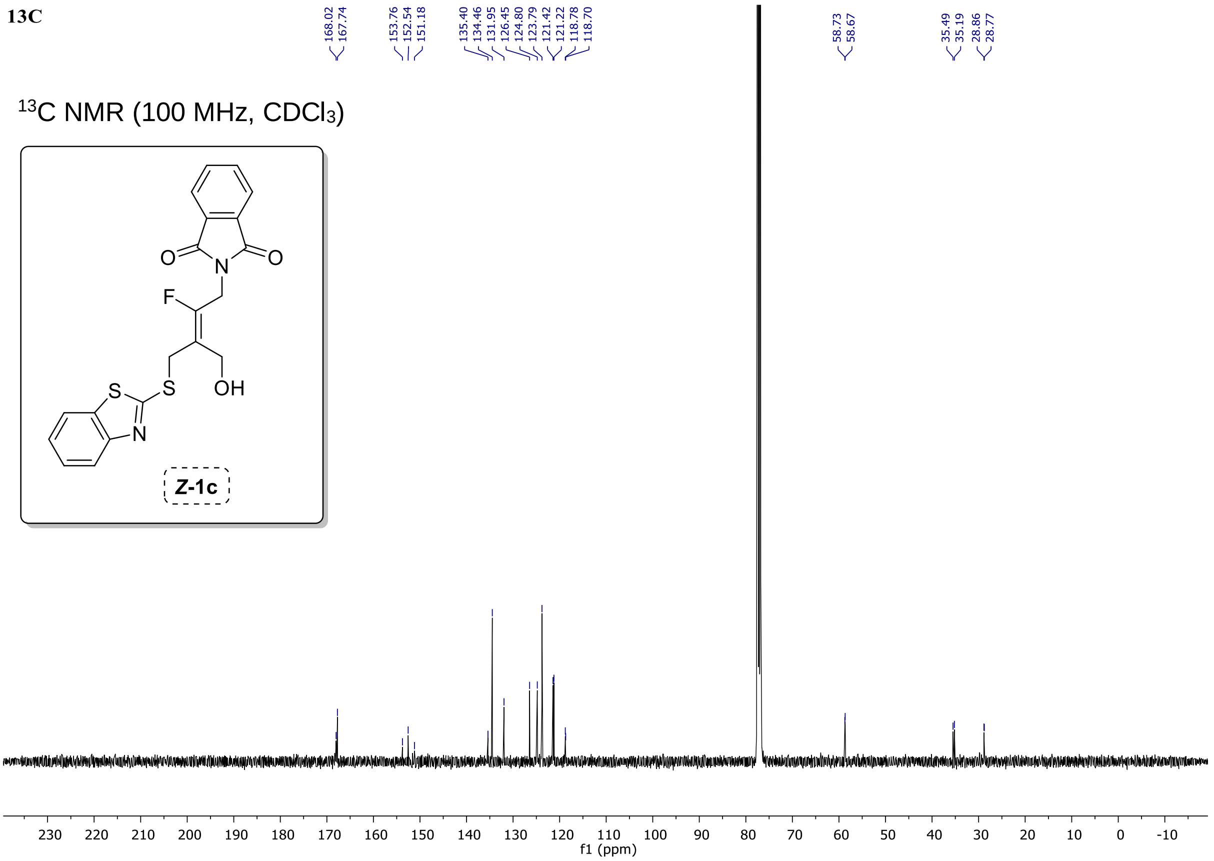
168.02
167.74
153.76
152.54
151.18
135.40
134.46
131.95
126.45
124.80
123.79
121.42
121.22
118.78
118.70
58.73
58.67
35.49
35.19
28.86
28.77
^{13}C NMR (100 MHz, CDCl$_3$)
O
N
O
F
S
S
OH
N
Z-1c
f1 (ppm)

19F

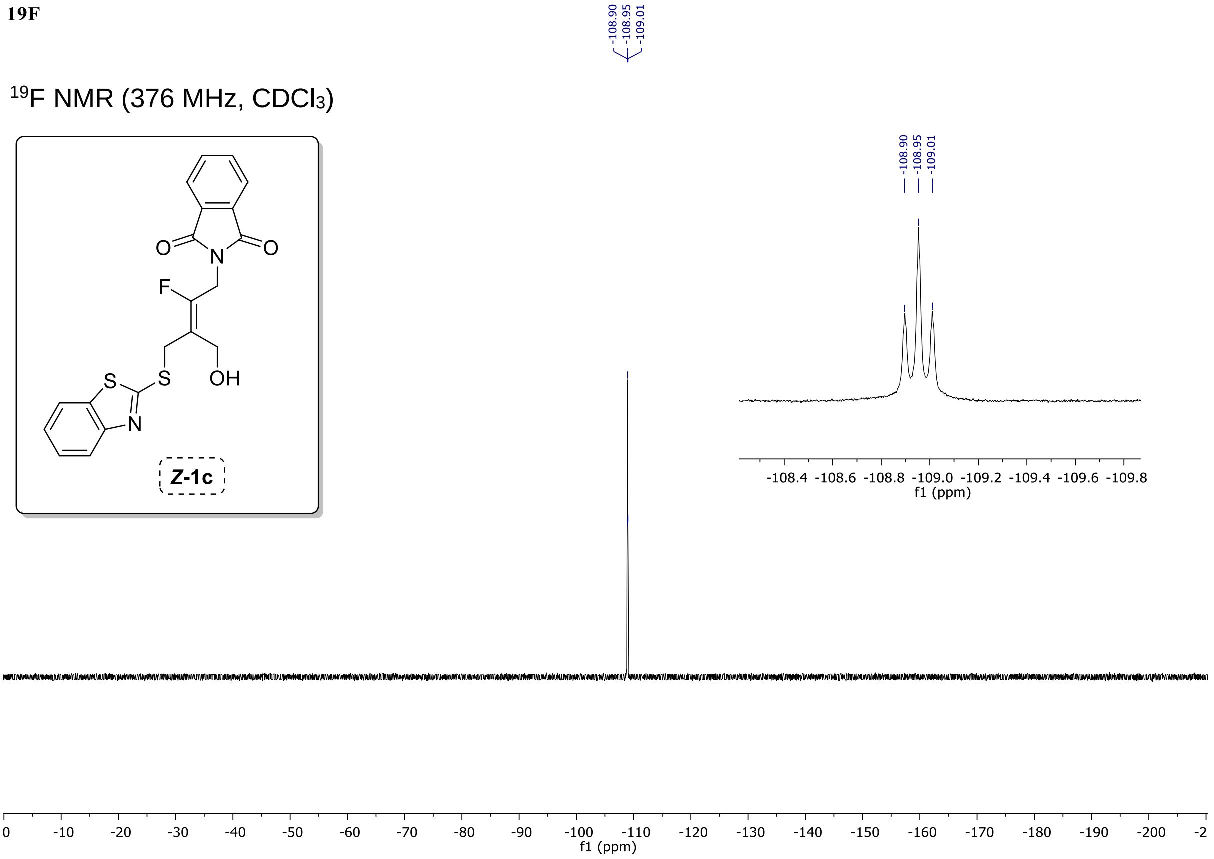
-108.90
-108.95
-109.01
^{19}F NMR (376 MHz, CDCl$_3$)
O
N
O
F
S
S
OH
N
Z-1c
f1 (ppm)

N-[(*E*)-3-(1,3-Benzothiazol-2-ylsulfanylmethyl)-2-fluoro-4-hydroxy-but-2-enyl] phthalimide **(*E*-1c)**

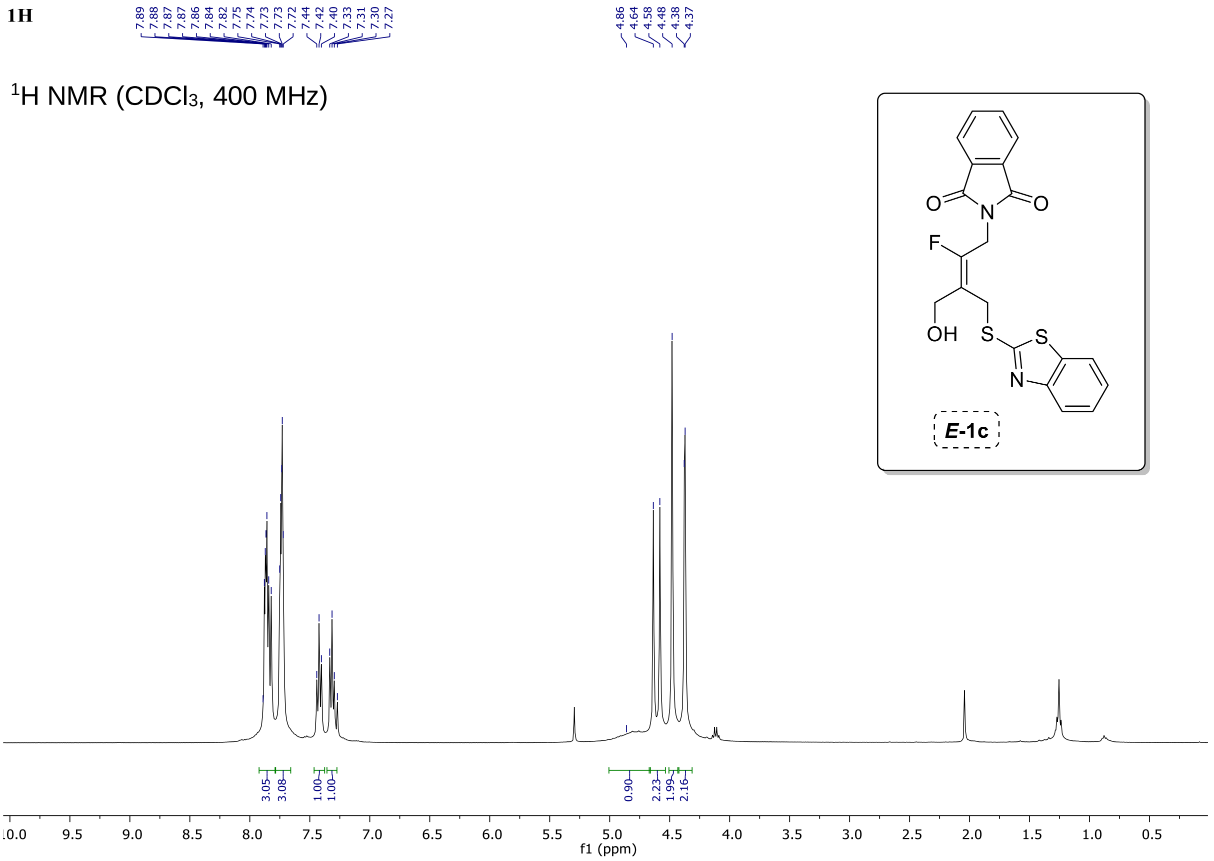

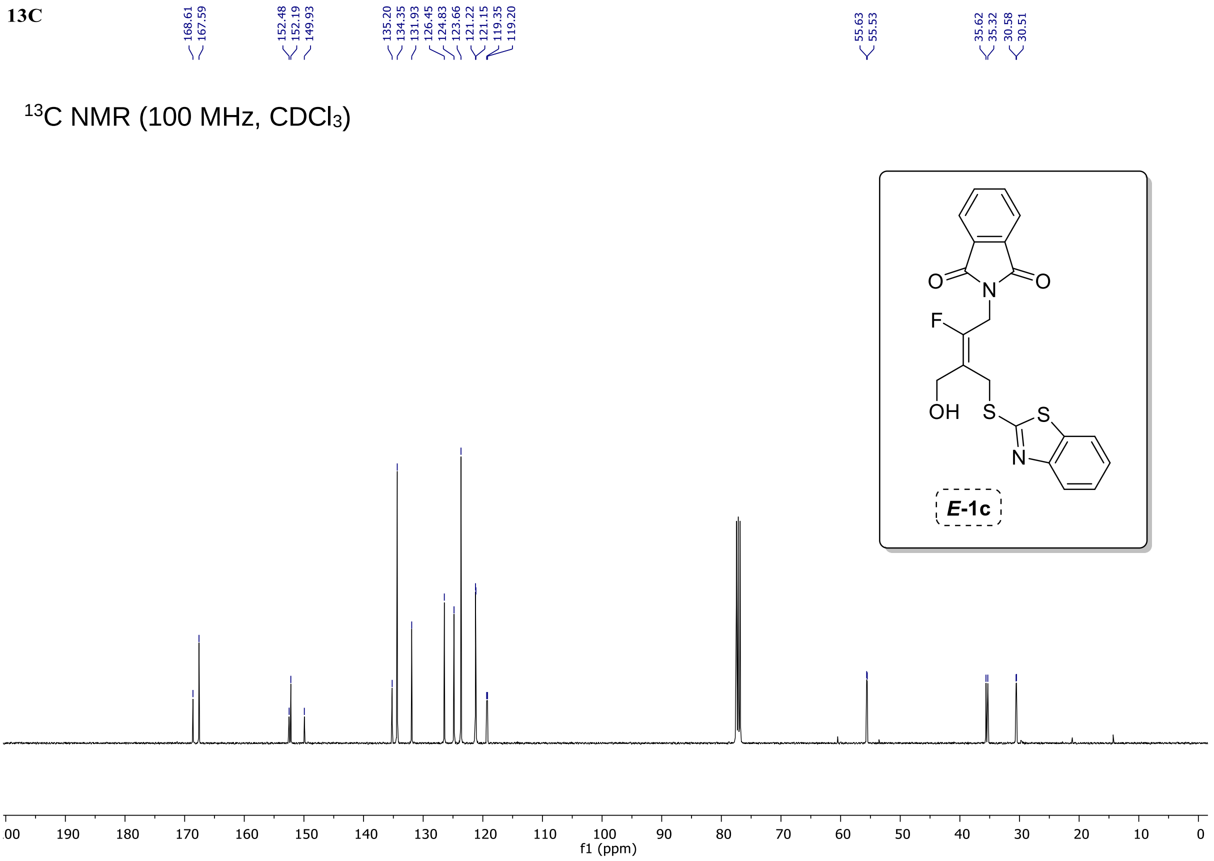

19F

^{19}F NMR (376 MHz, CDCl$_3$)

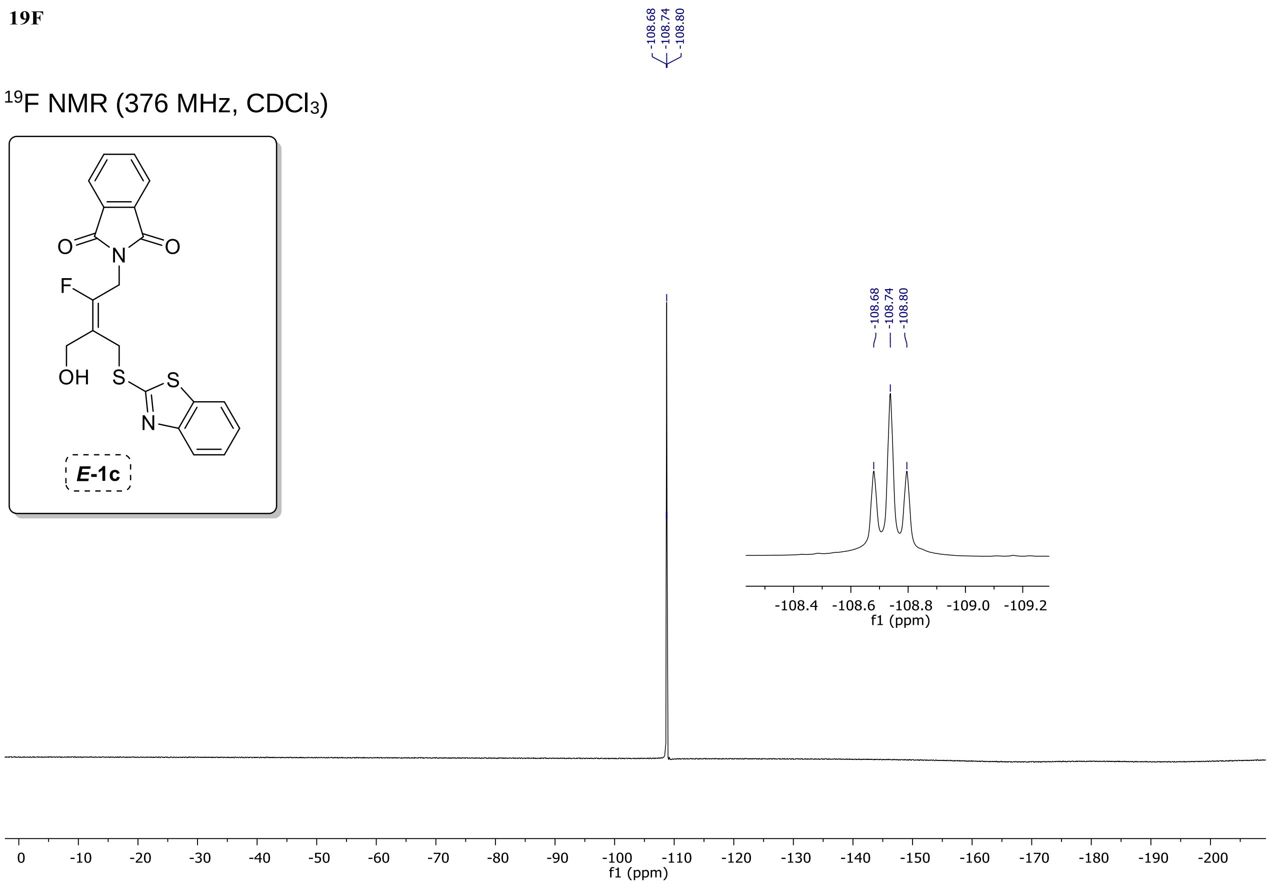

N-[3-(Bromomethyl)-2-fluoro-4-hydroxy-but-2-enyl] phthalimide **(1d)**

1H

^{1}H NMR (CDCl$_3$, 400 MHz)

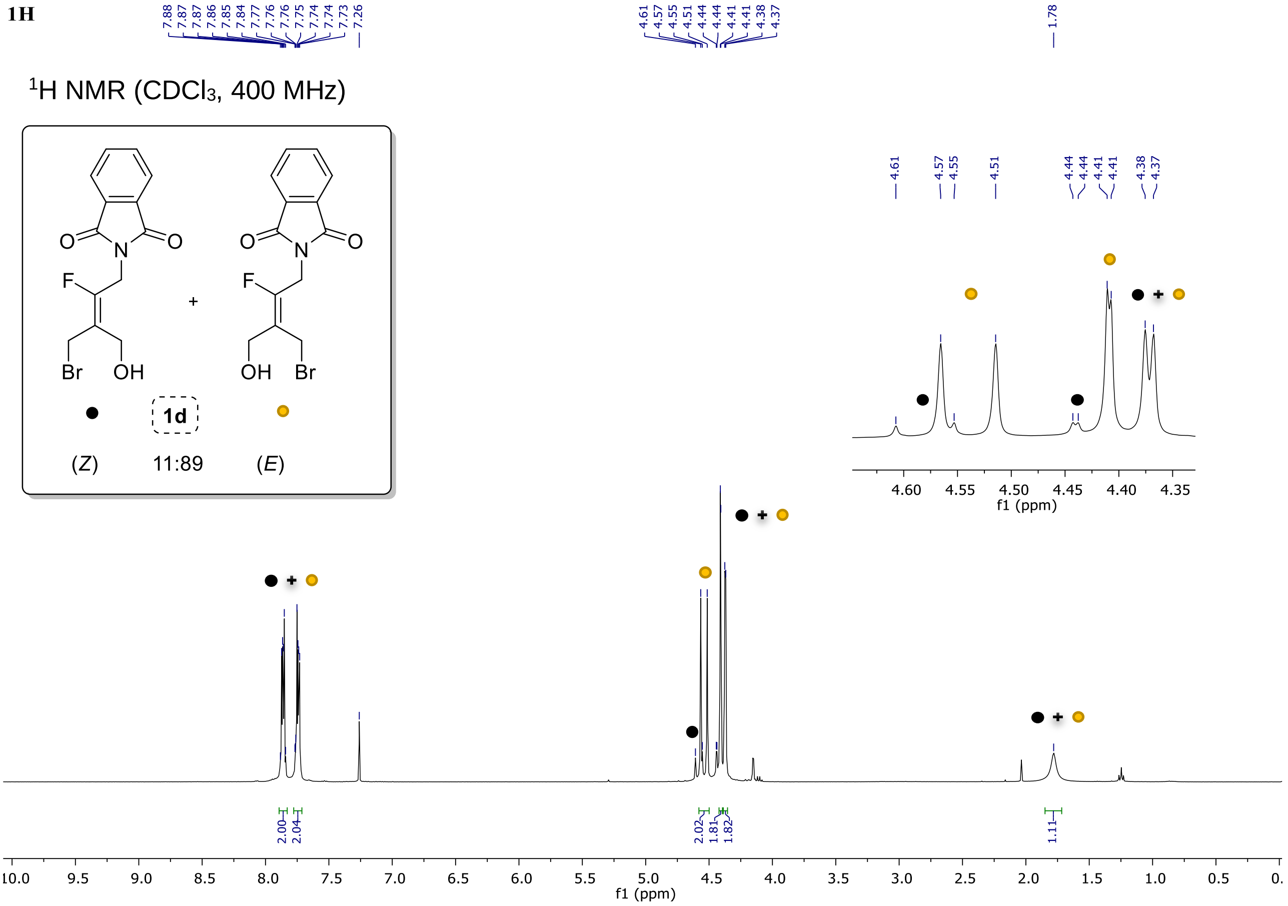

13C

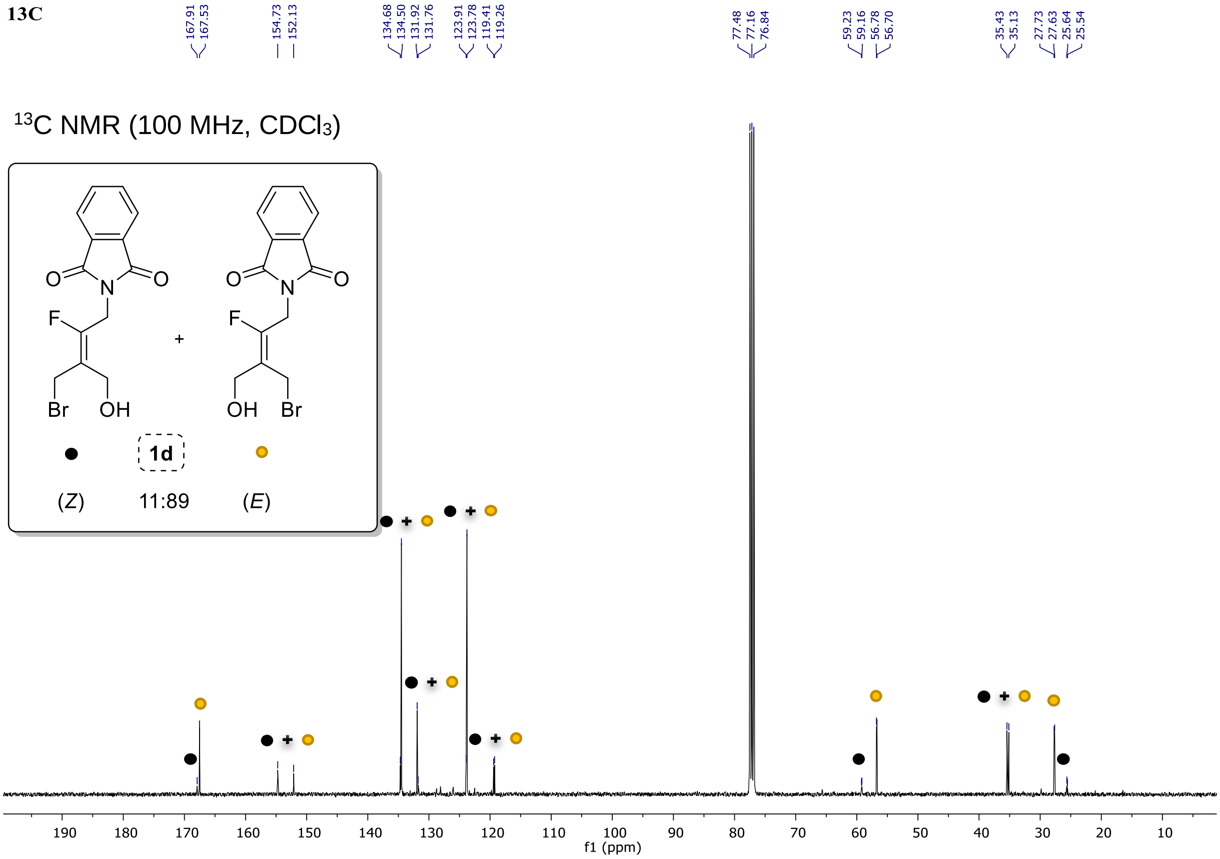

167.91
167.53
154.73
152.13
134.68
134.50
131.92
131.76
123.91
123.78
119.41
119.26
77.48
77.16
76.84
59.23
59.16
56.78
56.70
35.43
35.13
27.73
27.63
25.64
25.54
^{13}C NMR (100 MHz, CDCl$_3$)
F
O
N
O
Br
OH
OH
Br
+
1d
(Z)
11:89
(E)
f1 (ppm)

19F

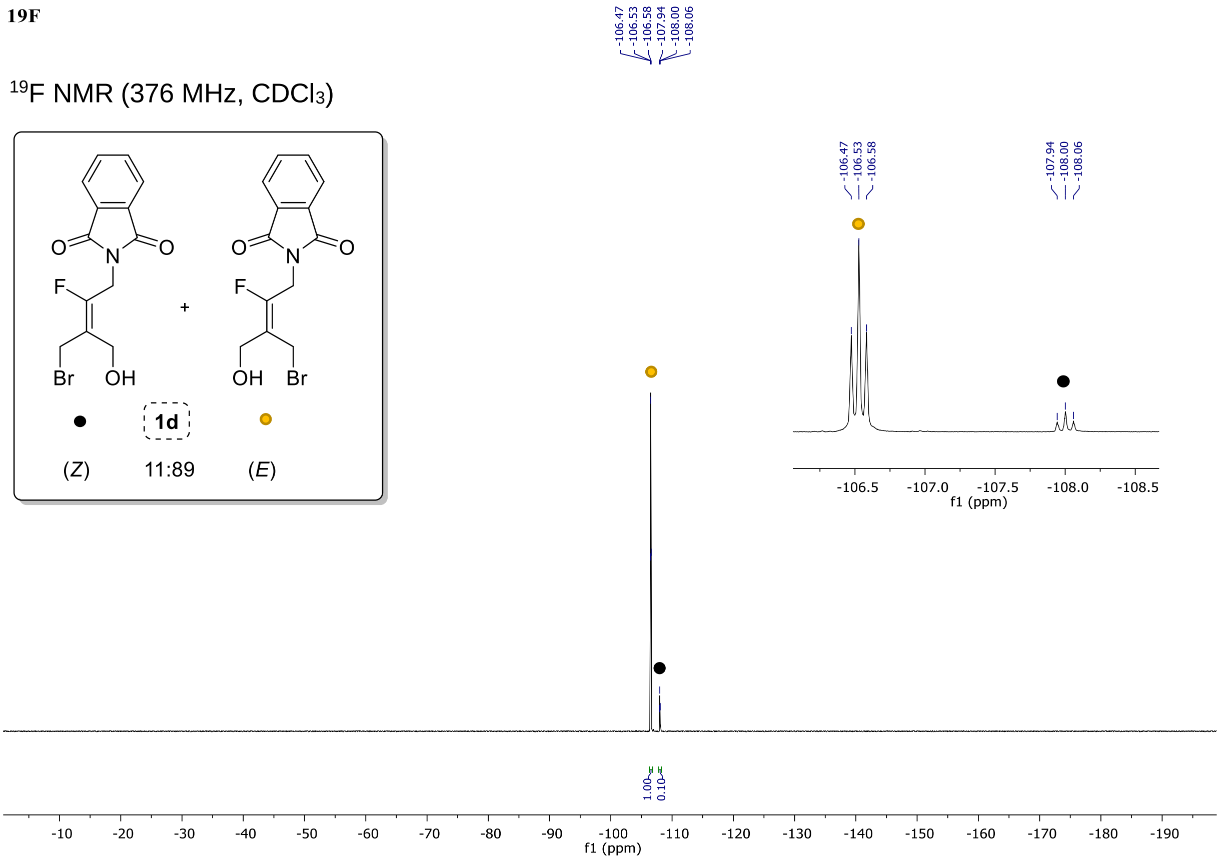

-106.47
-106.53
-106.58
-107.94
-108.00
-108.06
^{19}F NMR (376 MHz, CDCl$_3$)
F
O
N
O
Br
OH
OH
Br
+
1d
(Z)
11:89
(E)
1.00
0.10
f1 (ppm)

3-(1-Fluorononylidene)oxetane **(2)**

^{1}H NMR ($CDCl_3$, 500 MHz)

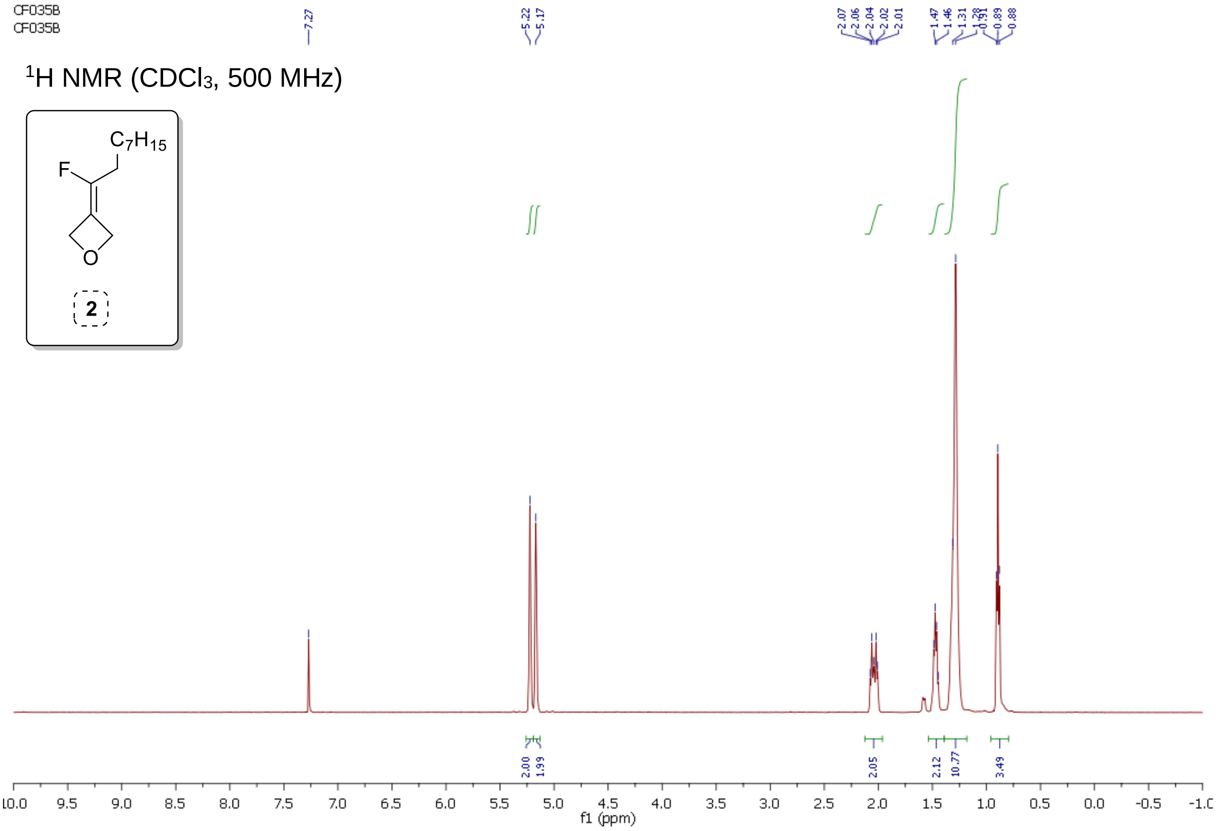

^{13}C NMR (126 MHz, $CDCl_3$)

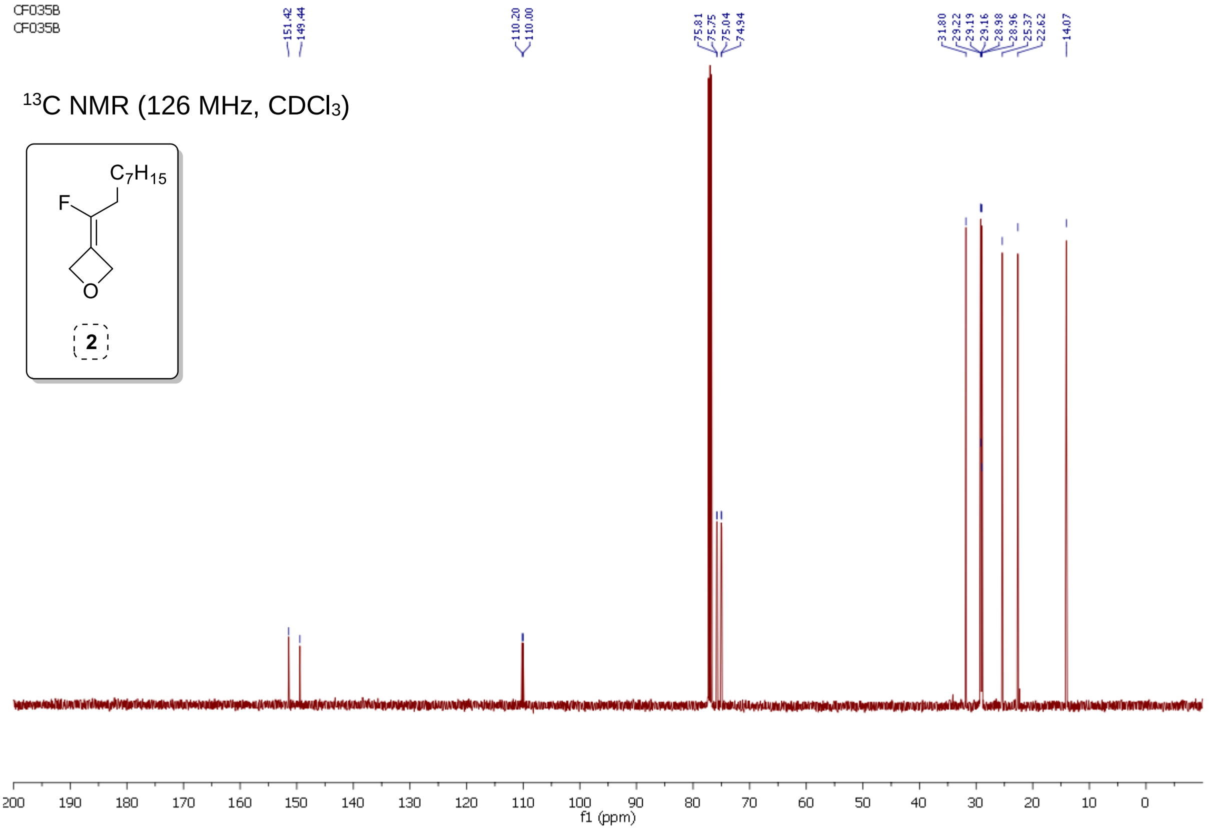

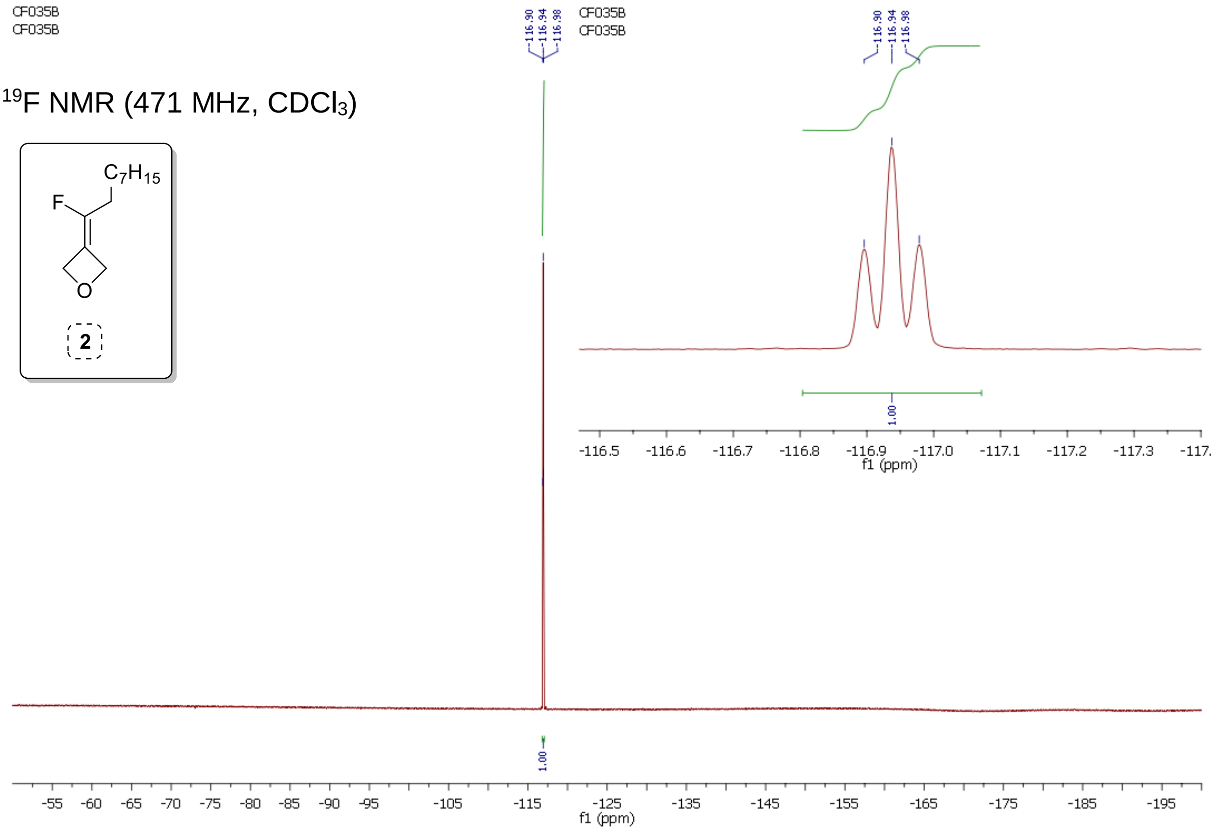

2-(Bromomethyl)-3-fluoro-undec-2-en-1-ol **(2d)**

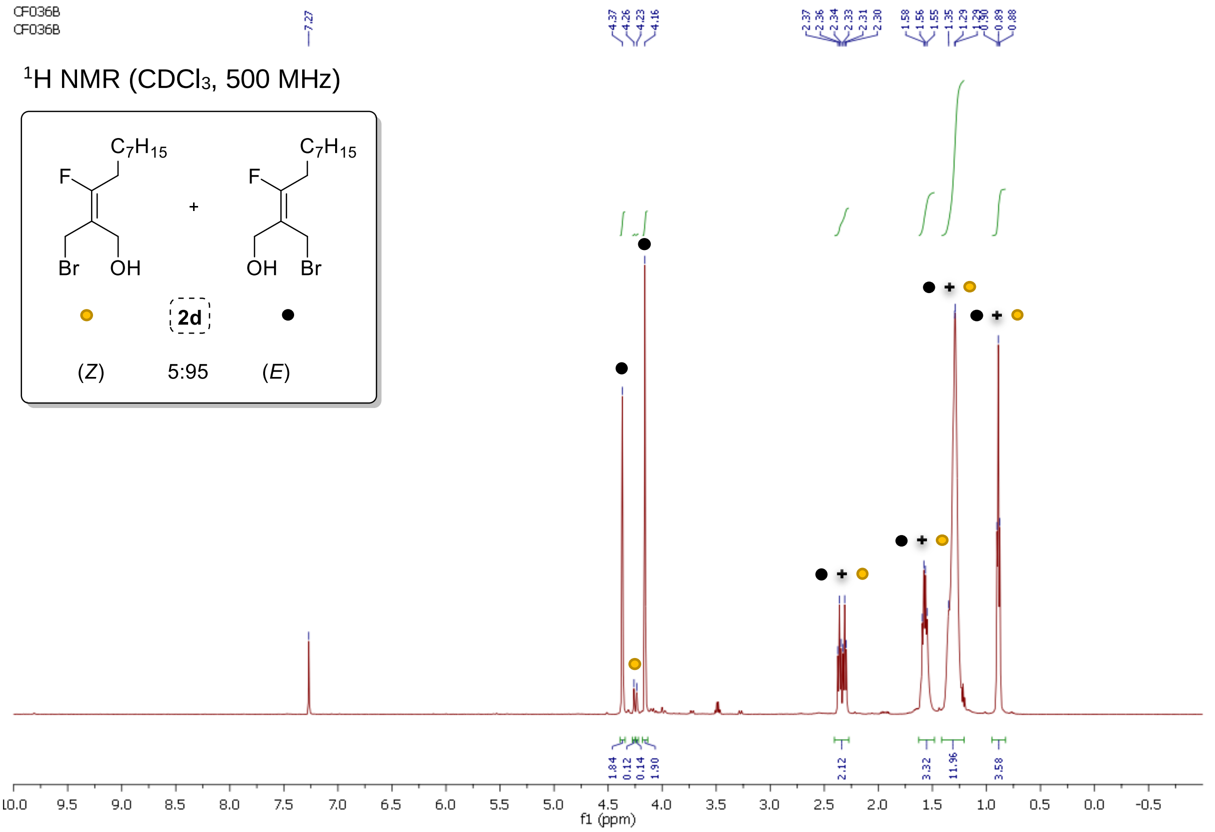

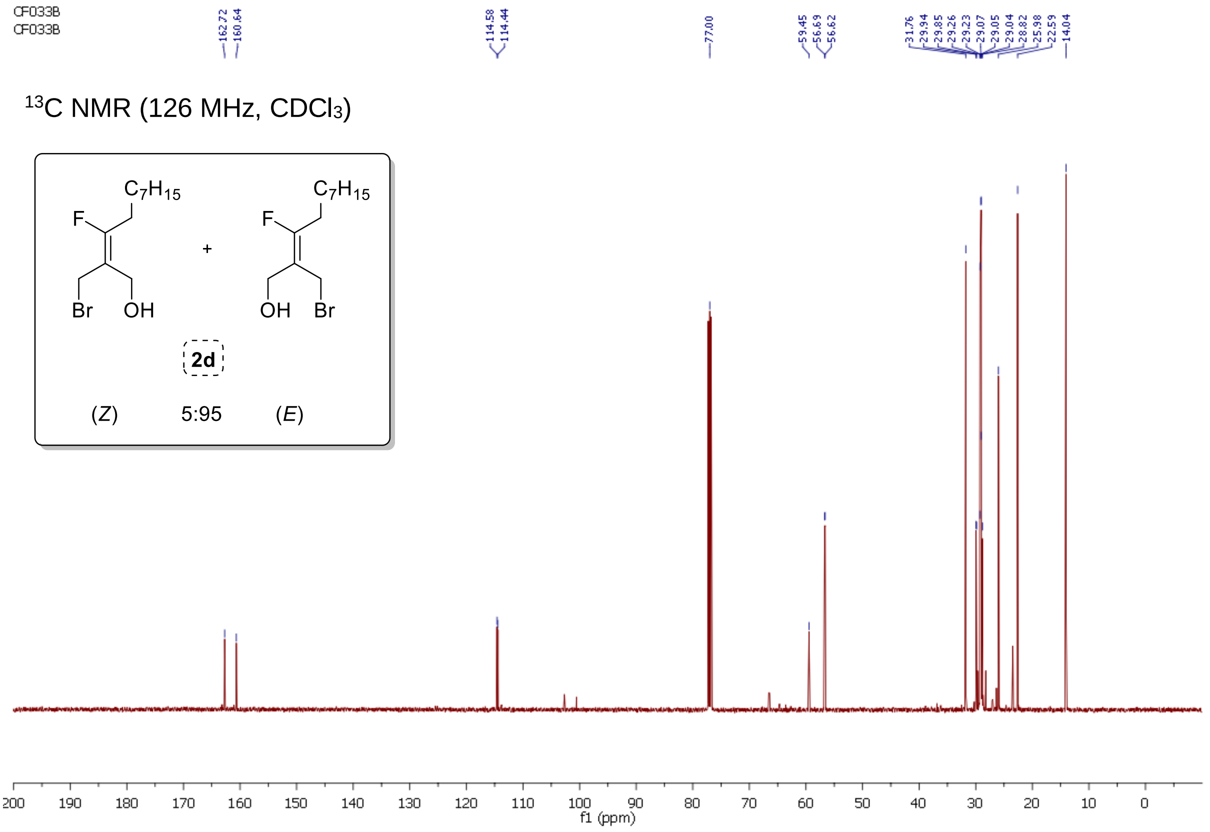
CF033B
CF033B
162.72
160.64
114.58
114.44
77.00
59.45
56.69
56.62
31.76
29.94
29.85
29.26
29.23
29.07
29.05
29.04
28.82
25.98
22.59
14.04
¹³C NMR (126 MHz, CDCl₃)
C₇H₁₅
F
Br
OH
+
2d
(Z)
5:95
(E)
f1 (ppm)

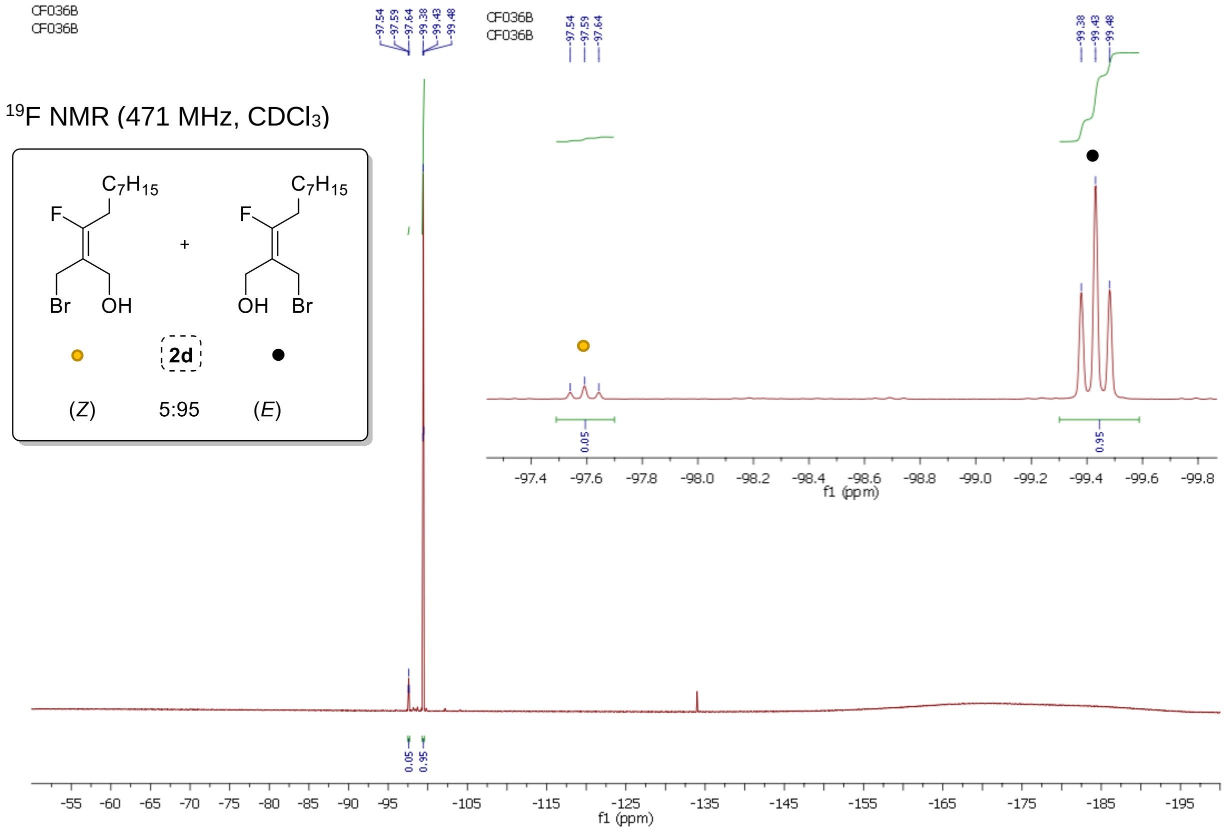
CF036B
CF036B
-97.54
-97.59
-97.64
-99.38
-99.43
-99.48
¹⁹F NMR (471 MHz, CDCl₃)
C₇H₁₅
F
Br
OH
+
2d
(Z)
5:95
(E)
0.05
0.95
f1 (ppm)

(2-(Bromomethyl)-3-fluoro-undec-2-enyl) acetate **(2d')**

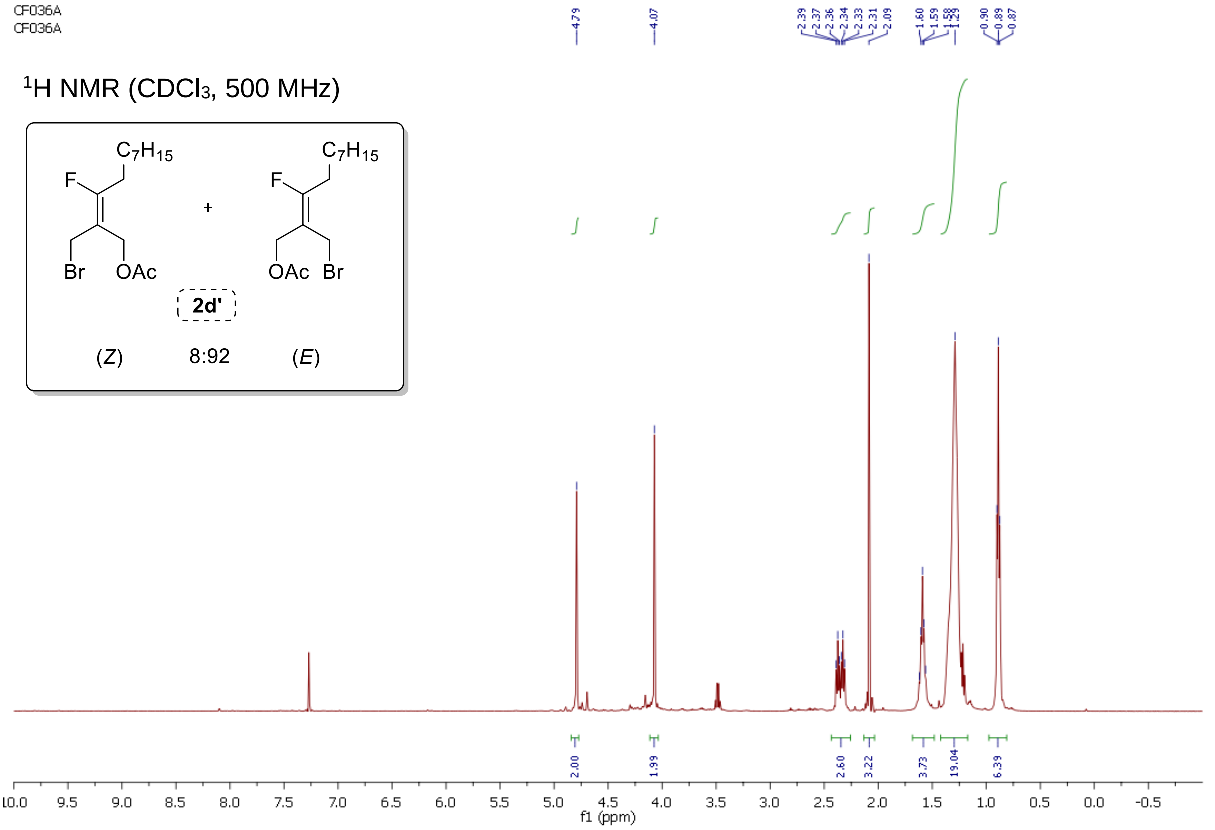

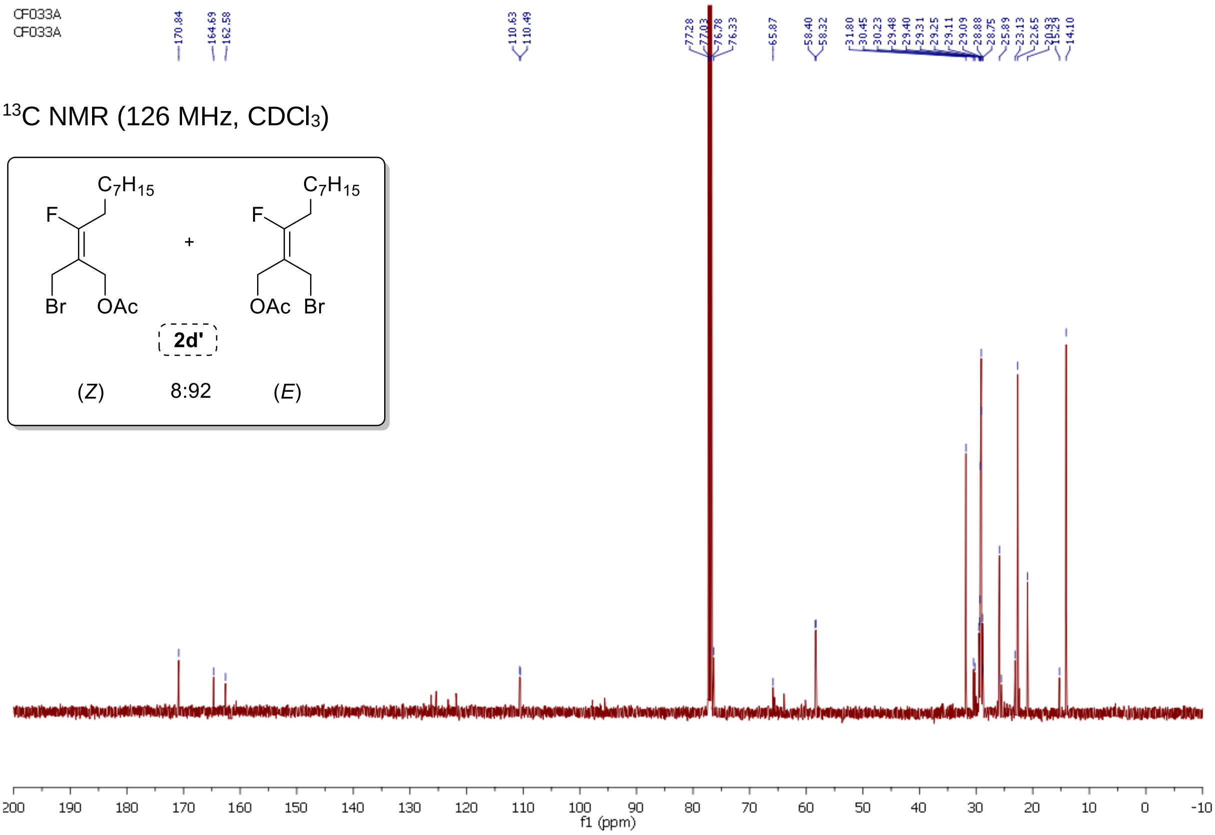

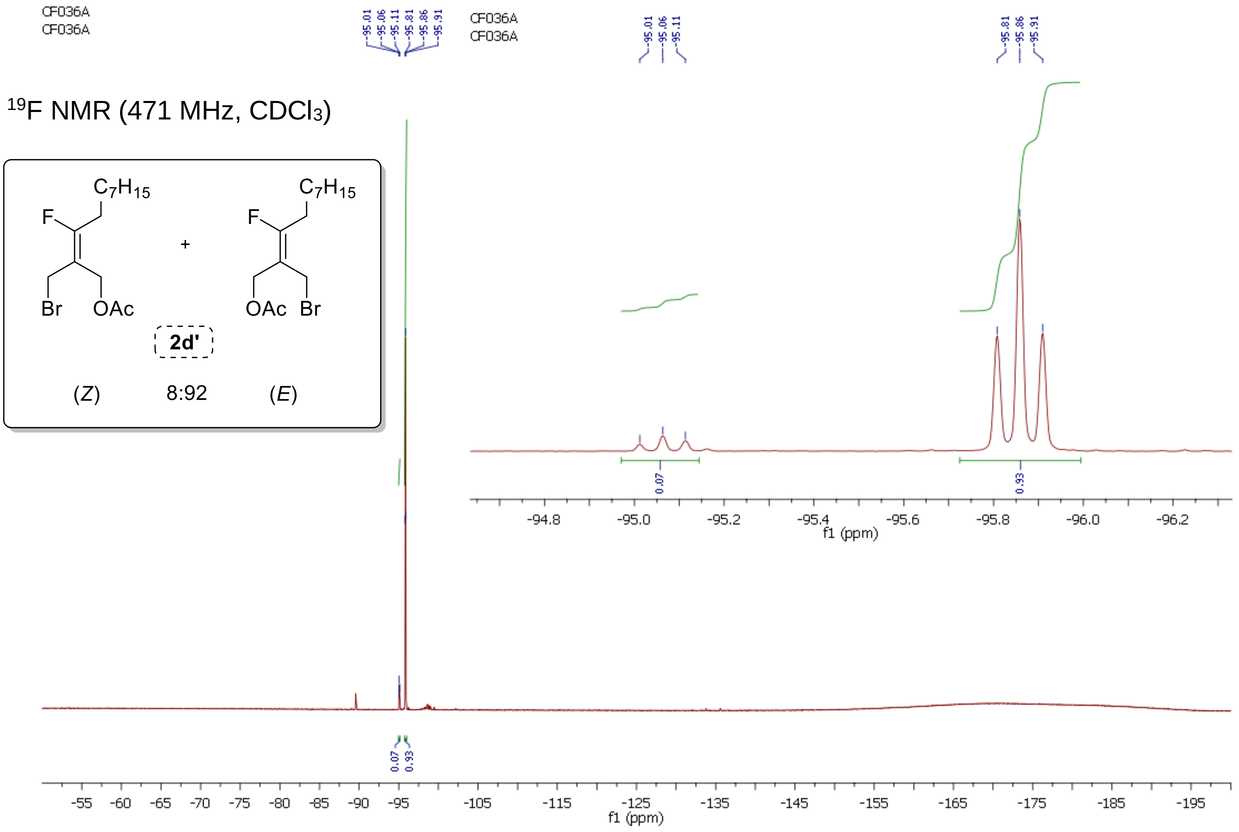

3-Benzoyl-1-[2-fluoro-2-(oxetan-3-ylidene)ethyl]-5-iodo-pyrimidine-2,4-dione **(3)**

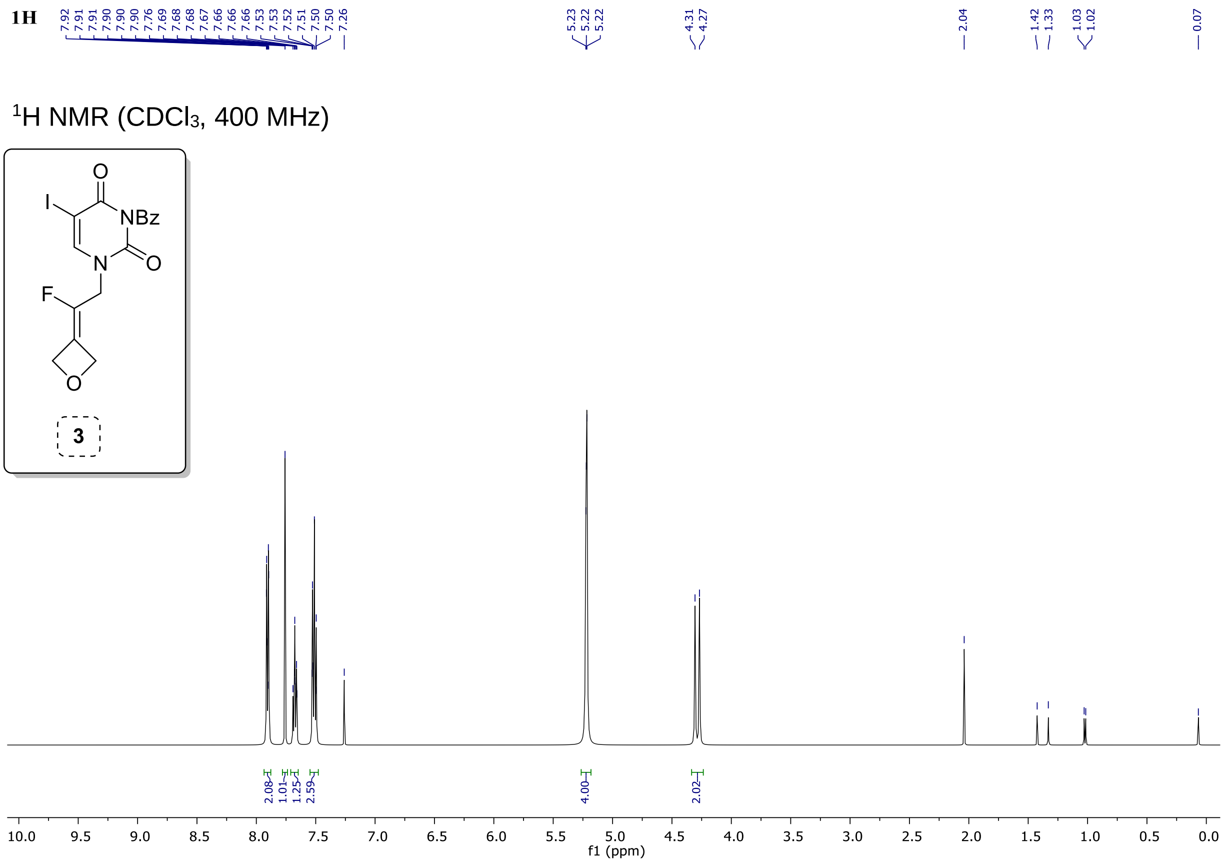

19F

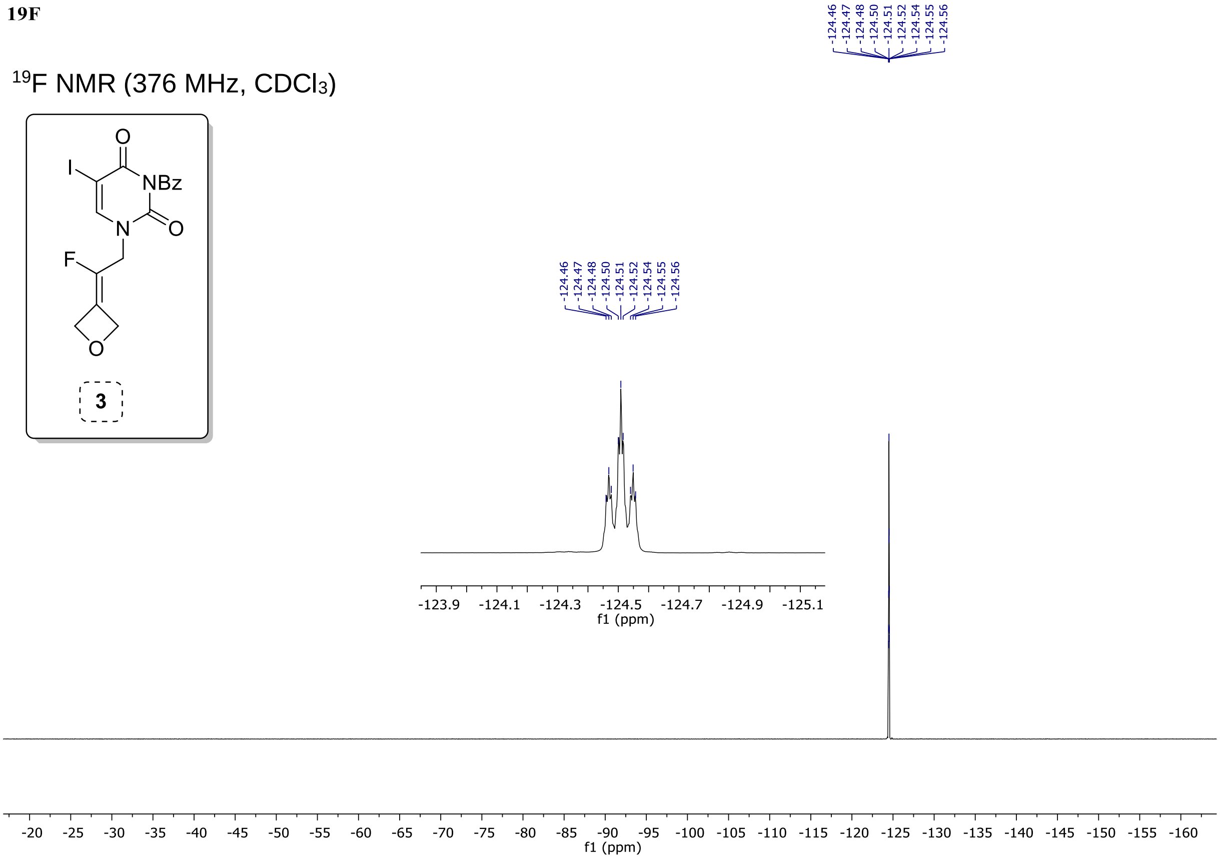

3-Benzoyl-1-[3-(bromomethyl)-2-fluoro-4-hydroxy-but-2-enyl]-5-iodo-pyrimidine-2,4-dione **(3d)**

1H

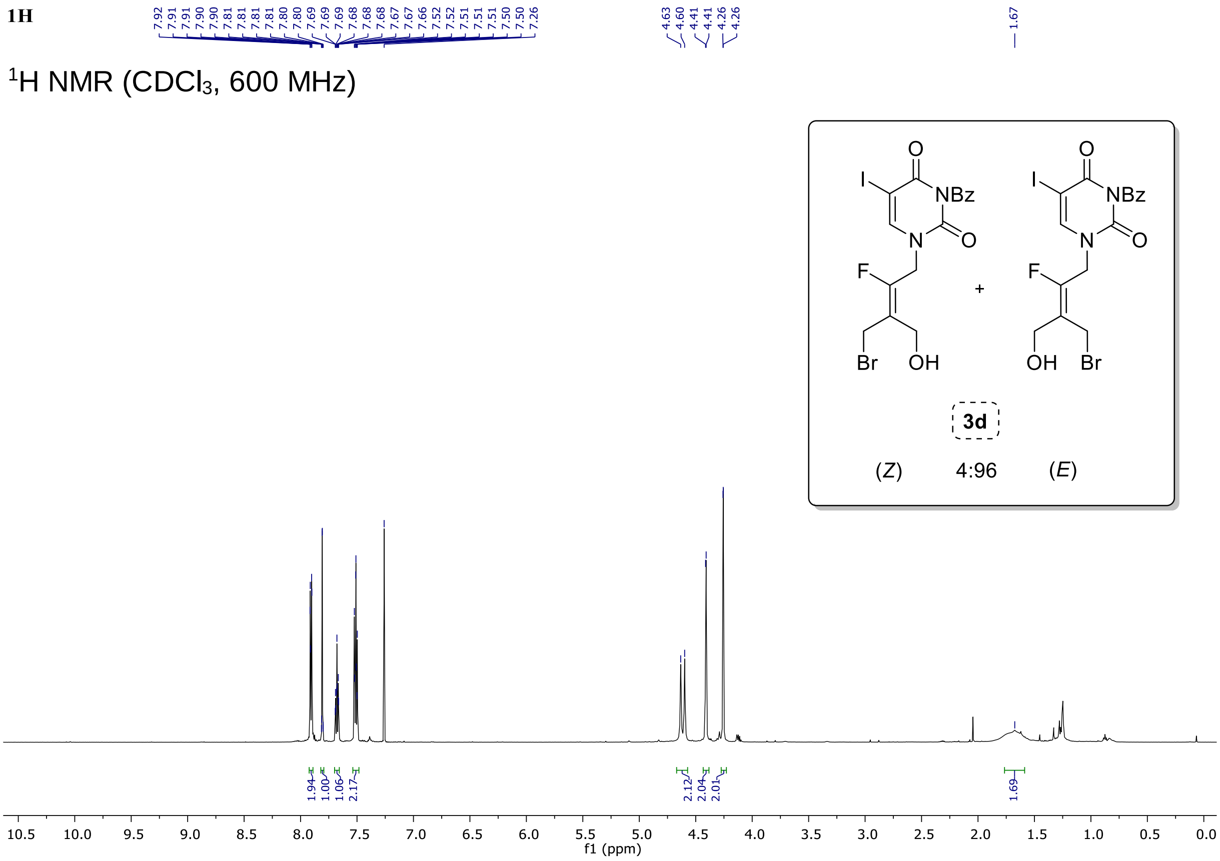

13C

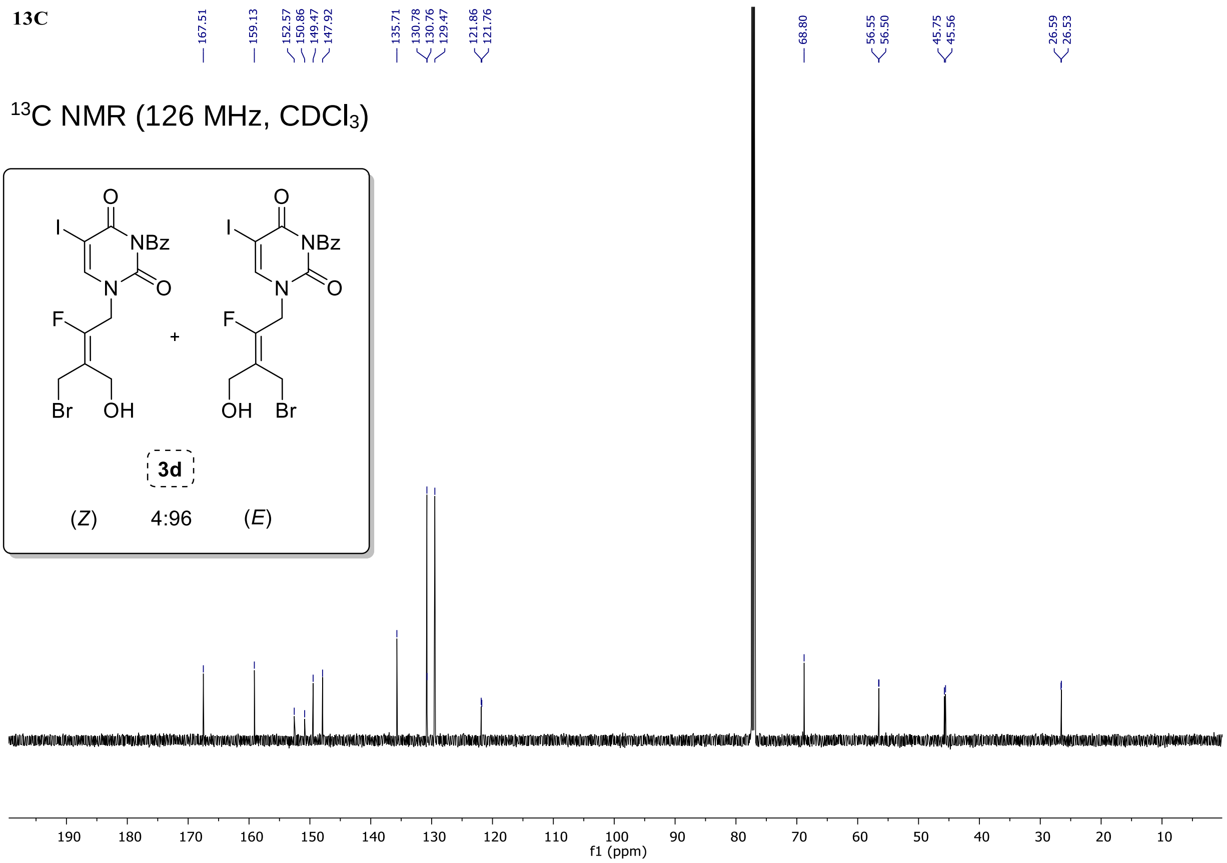

167.51
159.13
152.57
150.86
149.47
147.92
135.71
130.78
130.76
129.47
121.86
121.76
68.80
56.55
56.50
45.75
45.56
26.59
26.53
^{13}C NMR (126 MHz, CDCl$_3$)
3d
(Z) 4:96 (E)
f1 (ppm)

19F

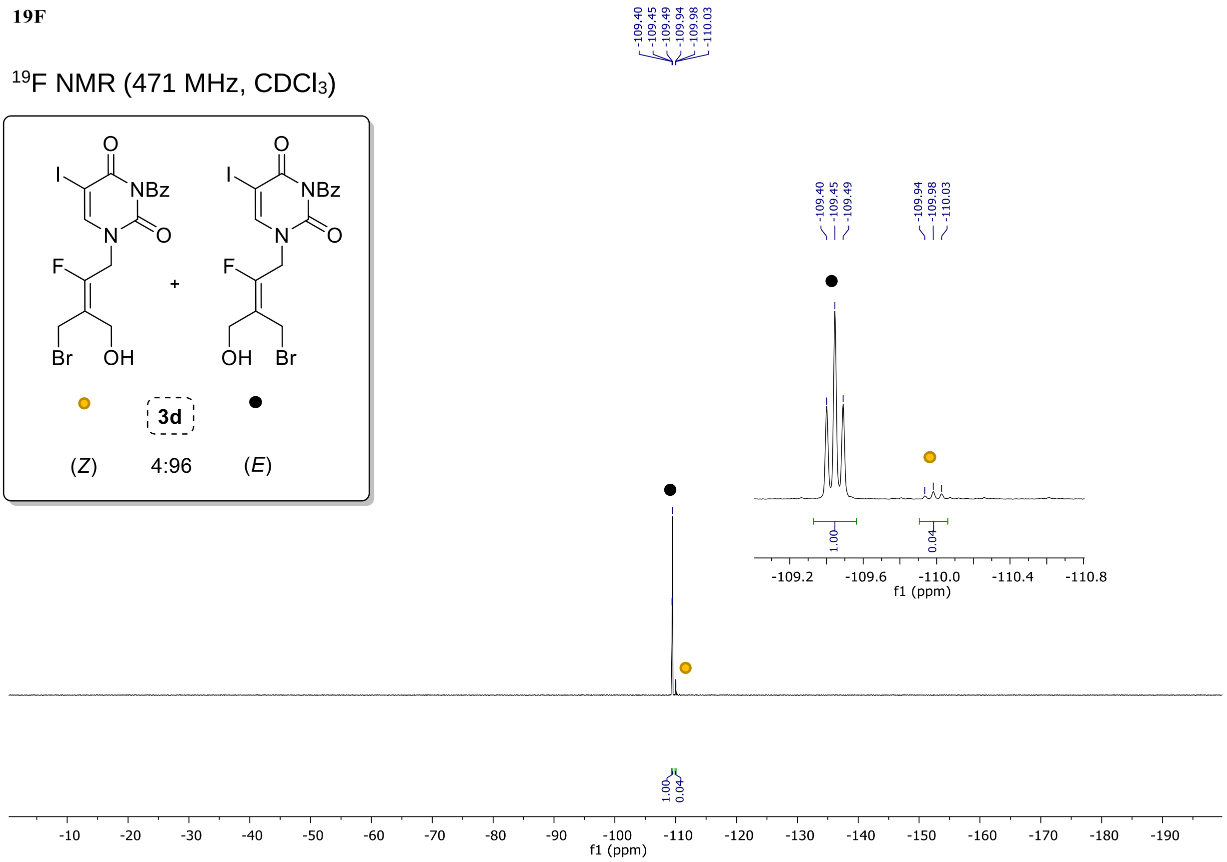

-109.40
-109.45
-109.49
-109.94
-109.98
-110.03
^{19}F NMR (471 MHz, CDCl$_3$)
3d
(Z) 4:96 (E)
1.00
0.04
f1 (ppm)

N-[2-(Oxetan-3-ylidene)ethyl] phthalimide **(4)**

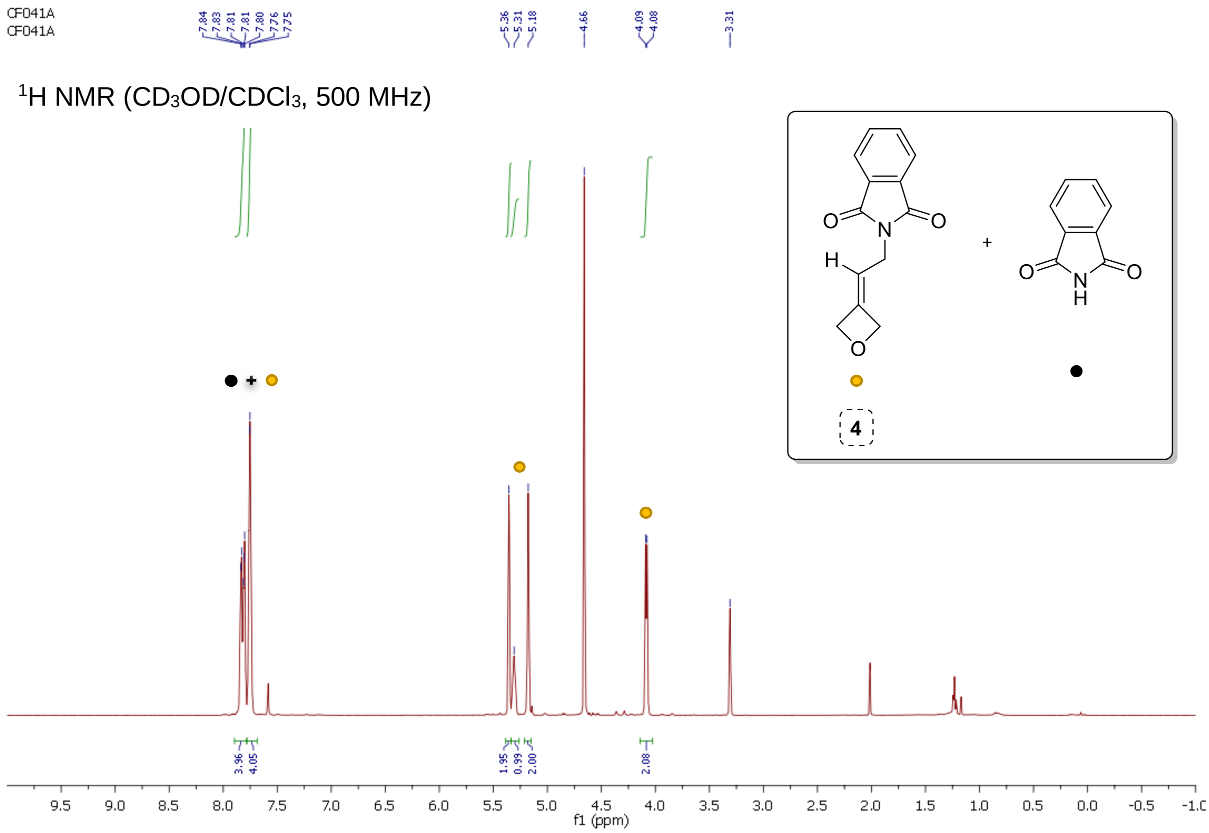

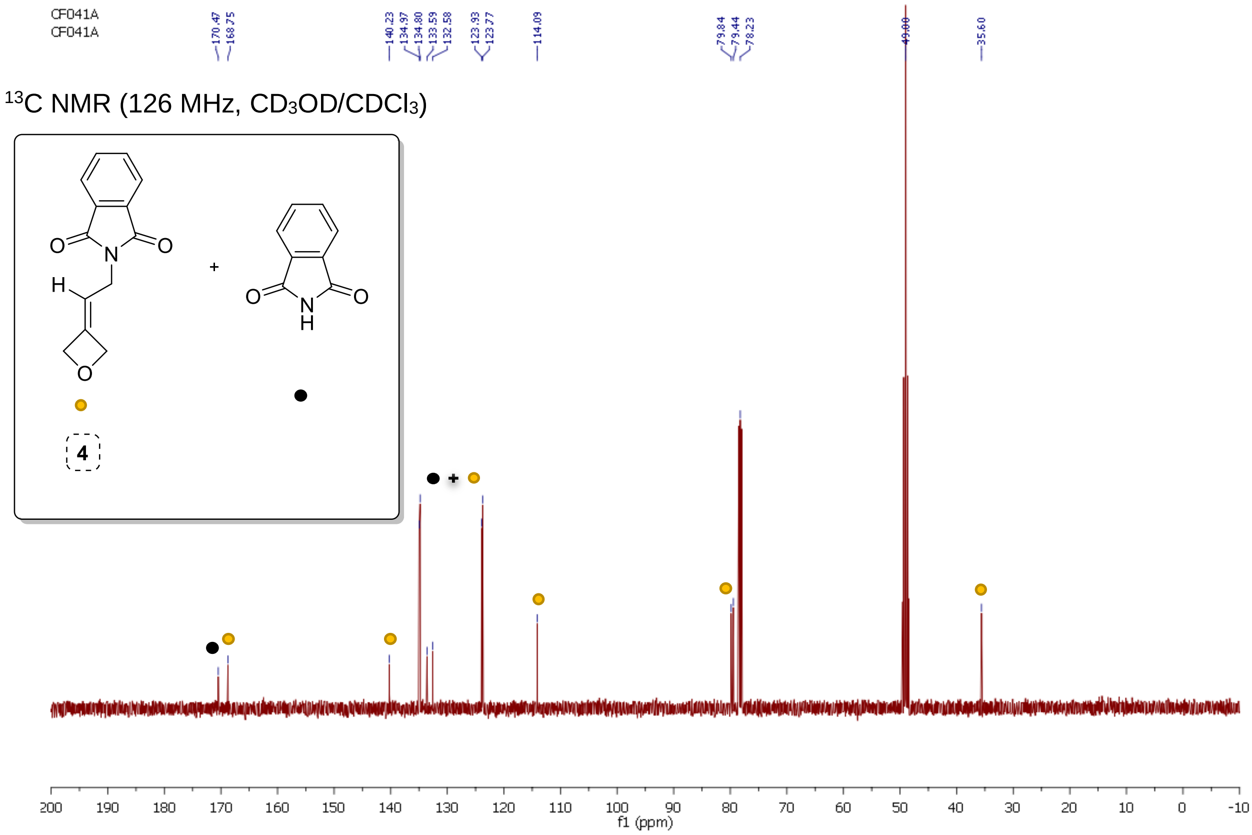

N-[(*E*)-3-(Bromomethyl)-4-hydroxy-but-2-en-1-yl] phthalimide **(*E*-4d)**

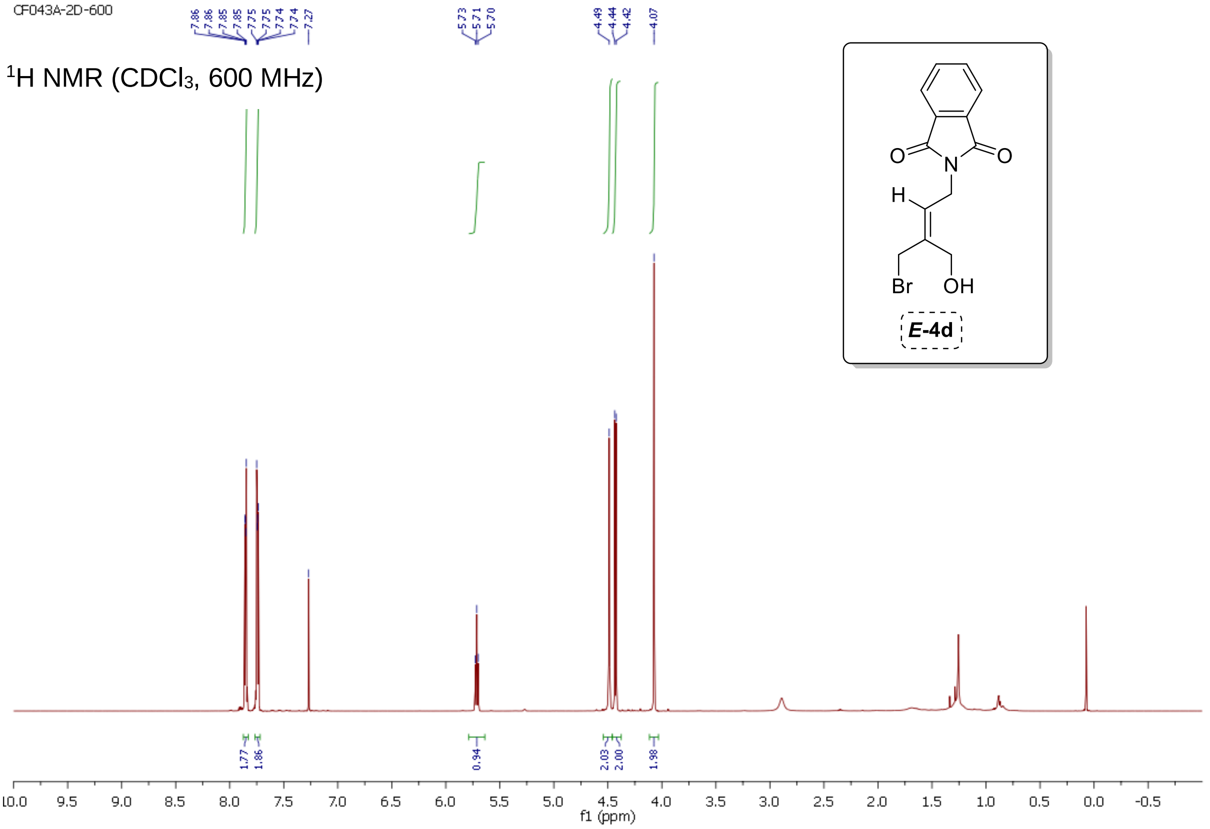

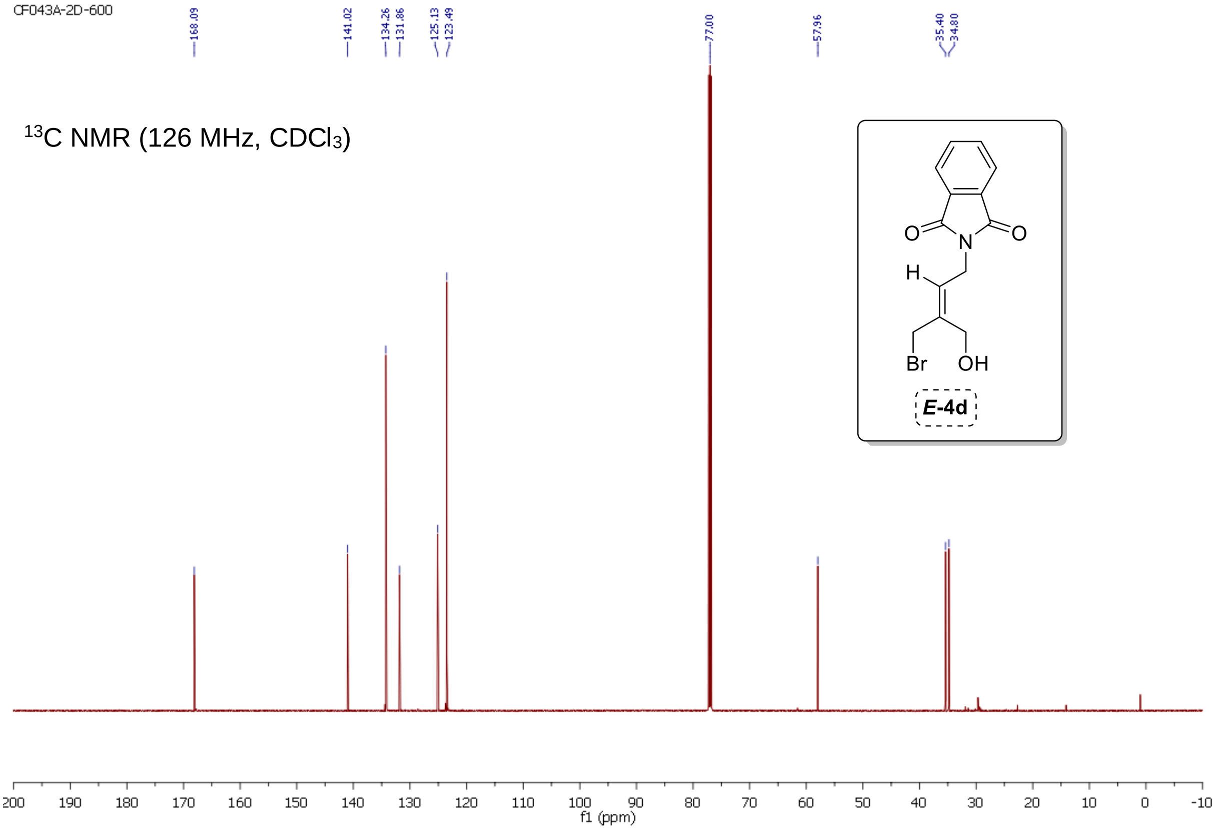

3-Decylideneoxetane **(5)**

^{1}H NMR ($CDCl_3$, 600 MHz)

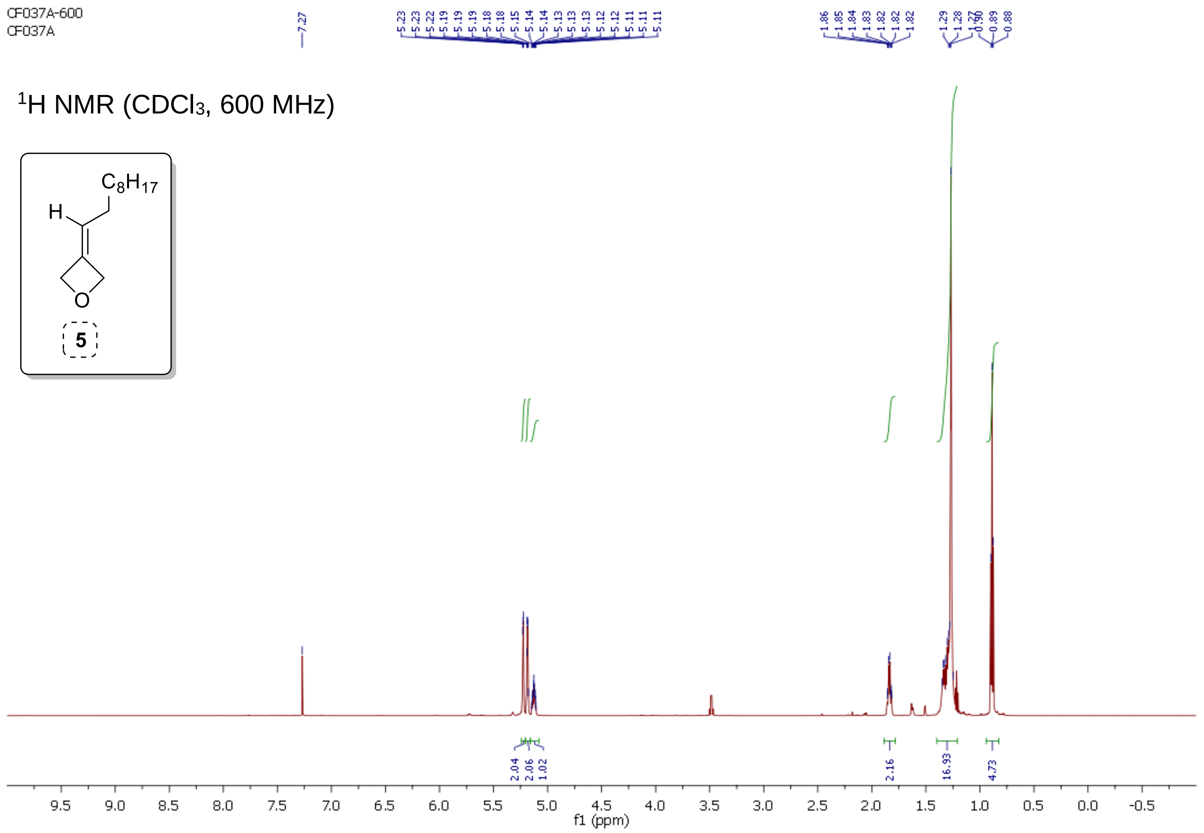

^{13}C NMR (126 MHz, $CDCl_3$)

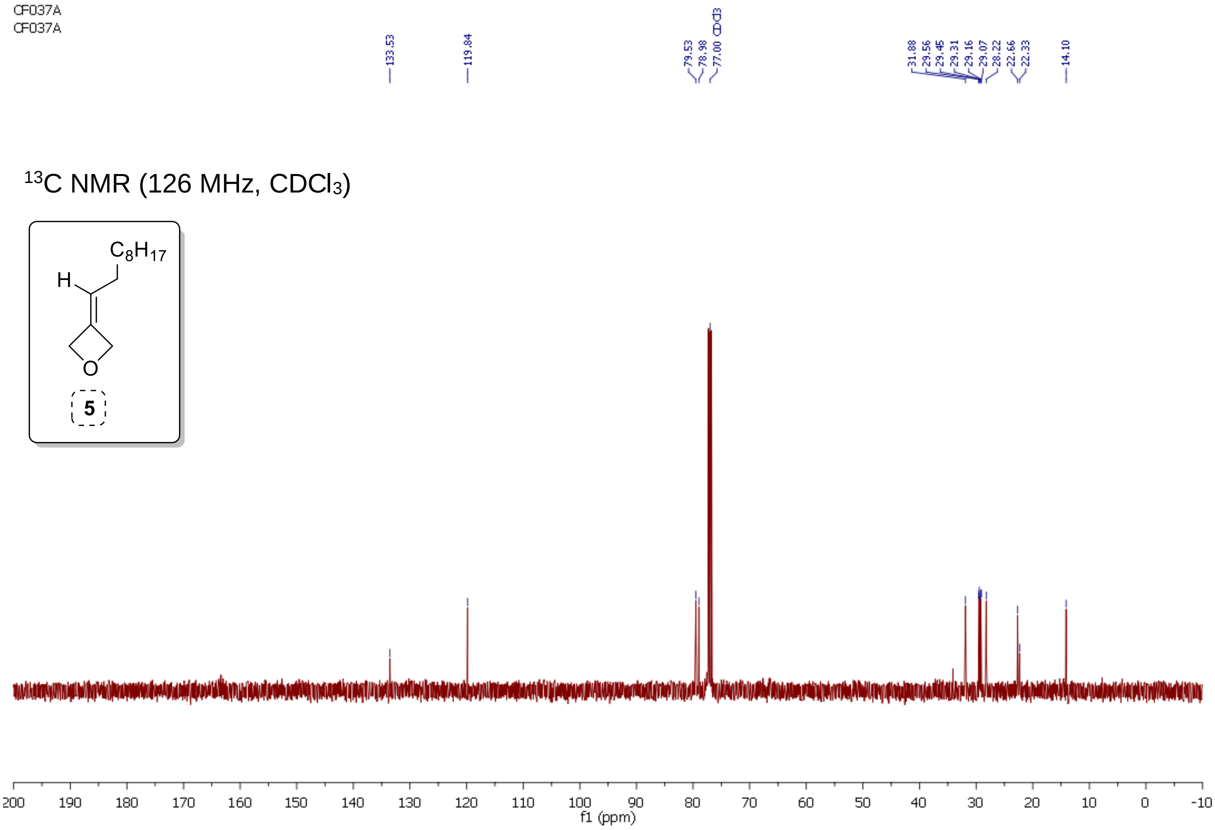

(*E*)-2-(Bromomethyl)dodec-2-en-1-ol **(*E*-5d)**

^1H NMR ($CDCl_3$, 600 MHz)

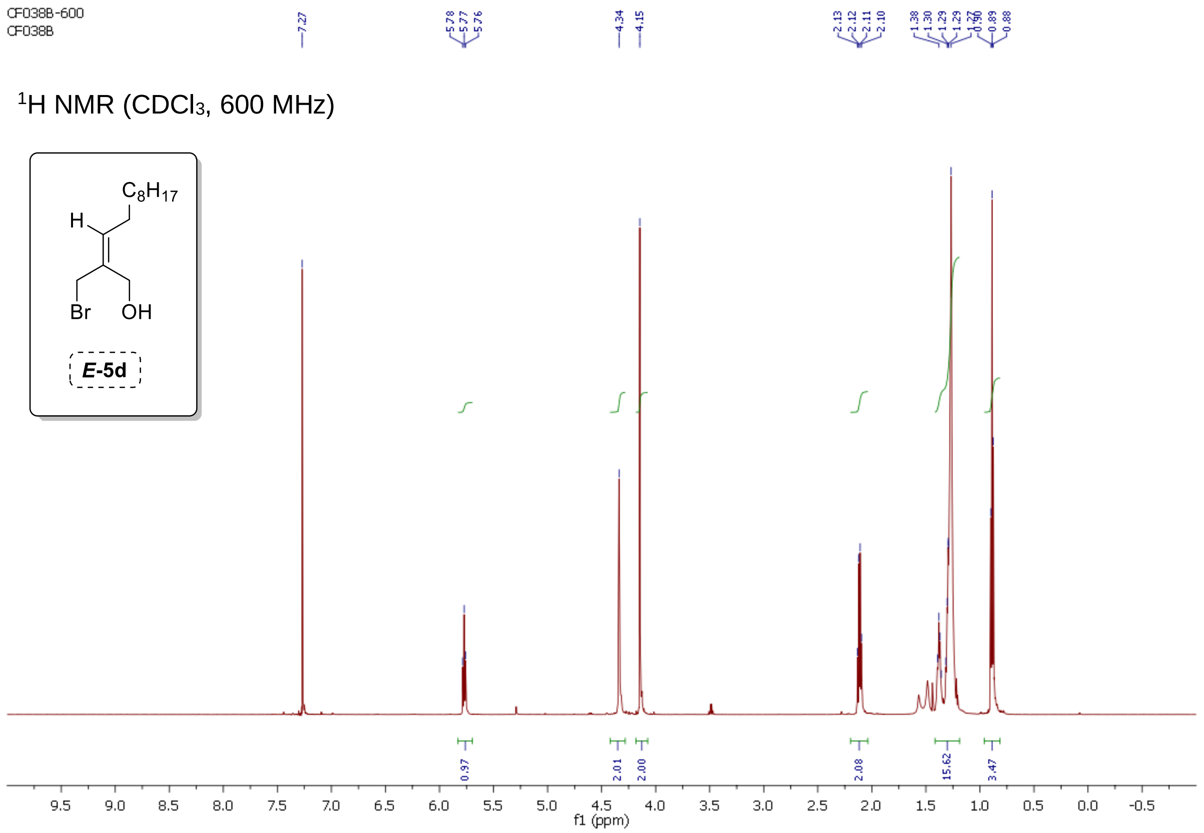

^{13}C NMR (126 MHz, $CDCl_3$)

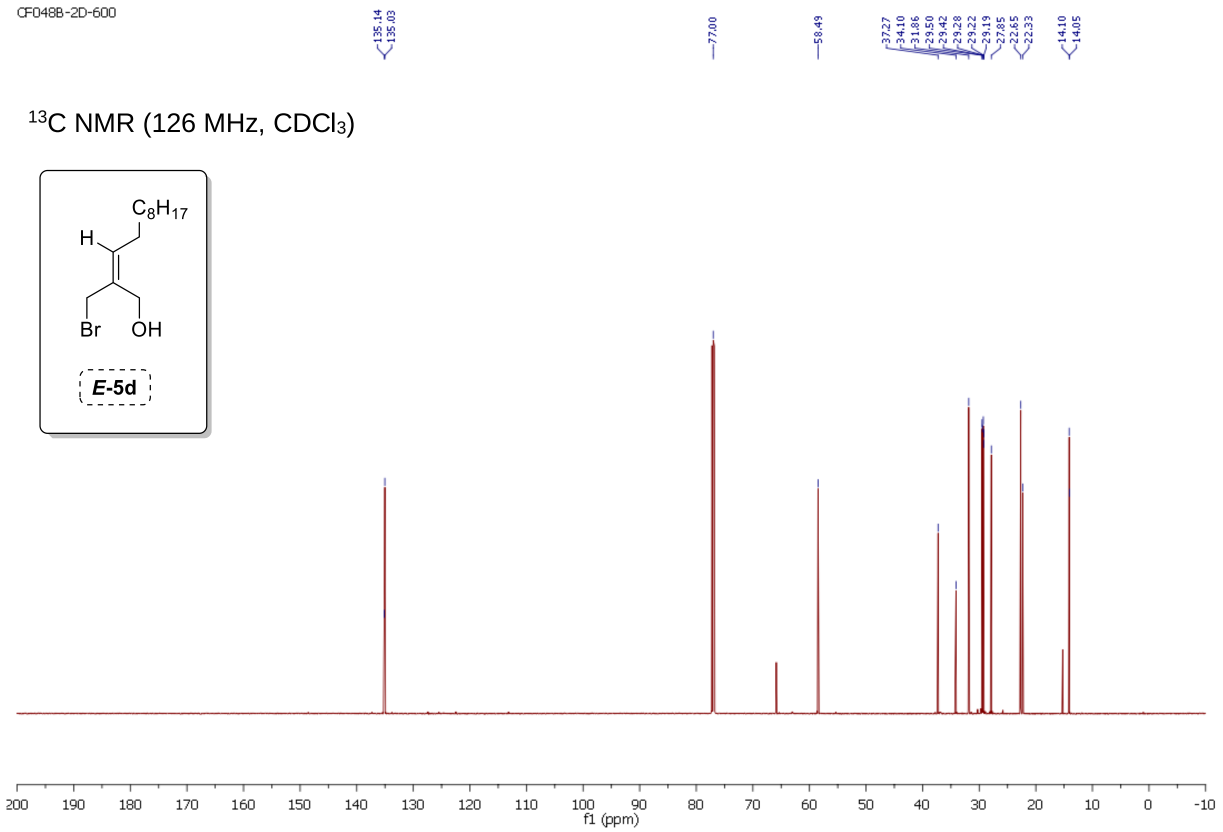

[(*E*)-2-(Bromomethyl)dodec-2-enyl] acetate (***E*-5d'**)

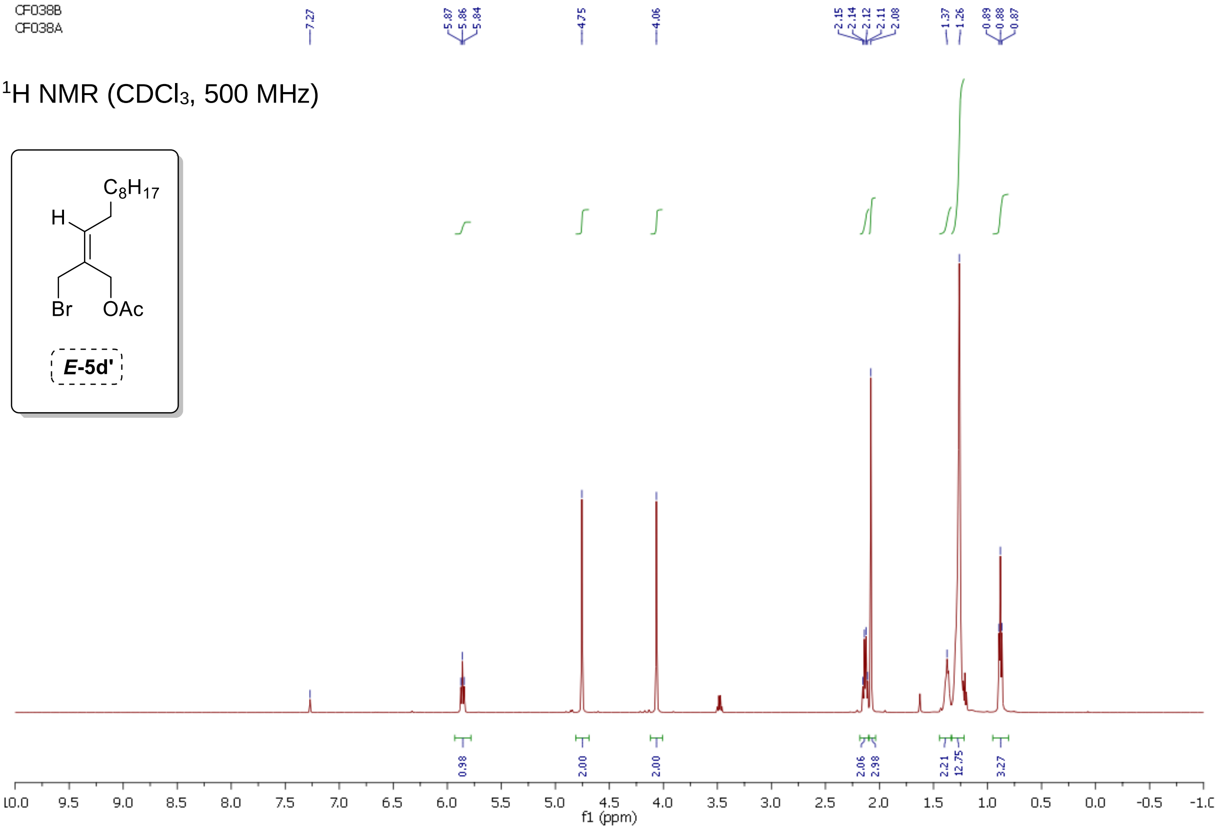

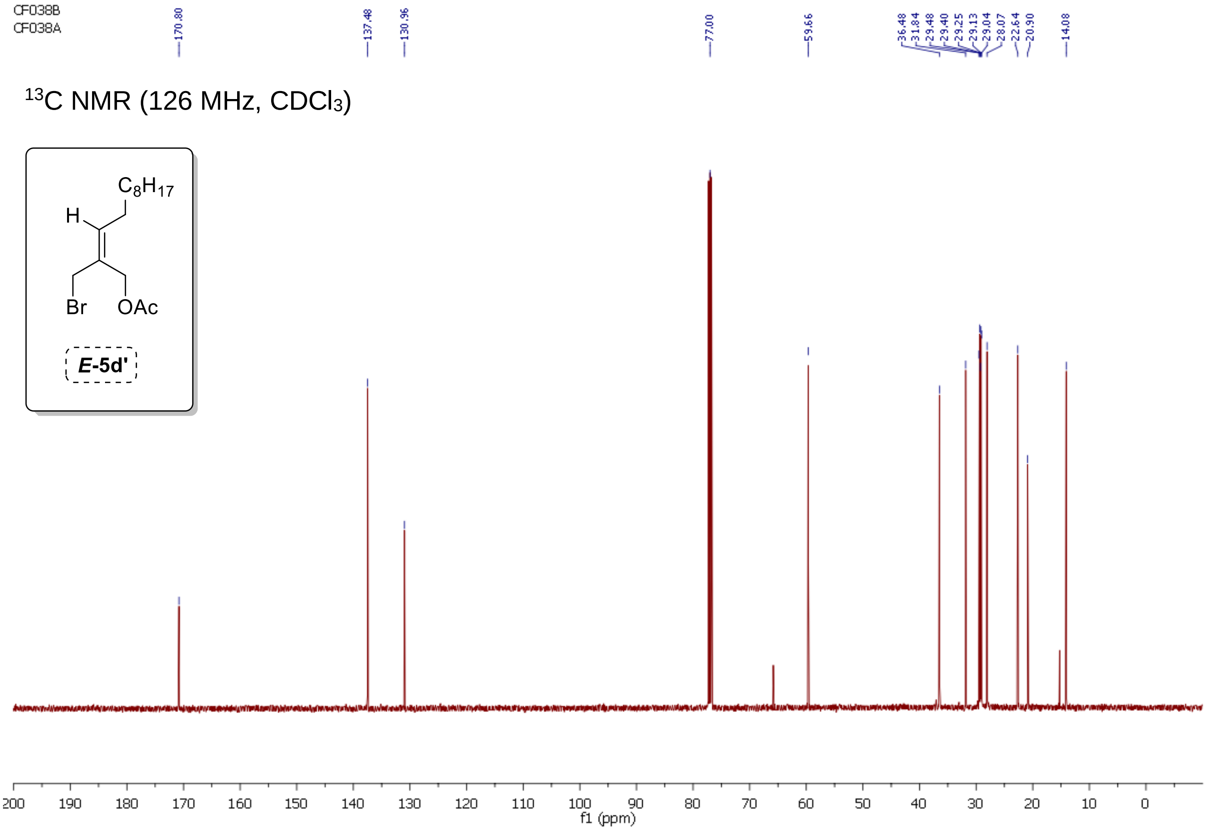

2-(2-Benzyloxy-1-fluoro-ethyl)sulfonyl-1,3-benzothiazole **(7)**

1H

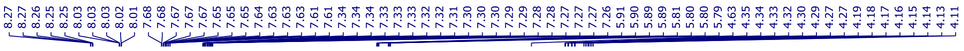

^{1}H NMR ($CDCl_3$, 500 MHz)

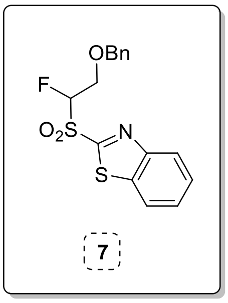

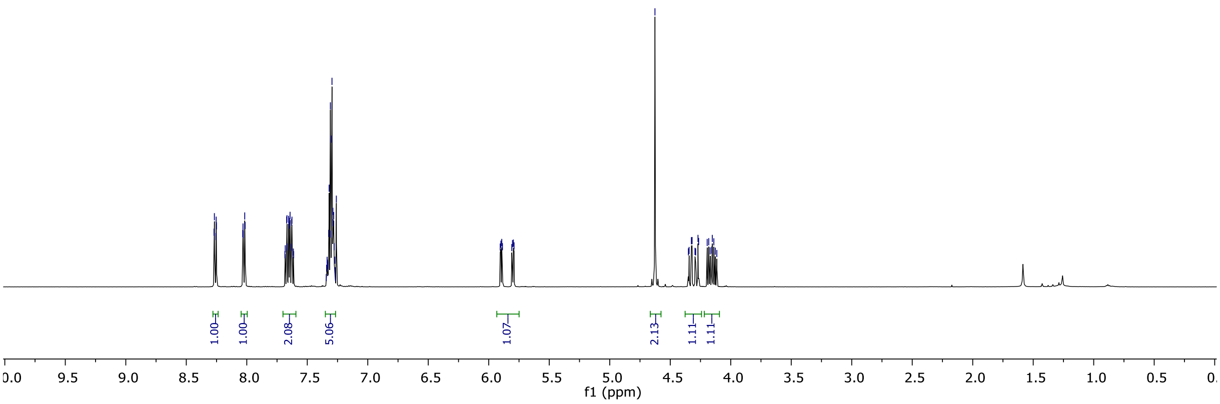

13C

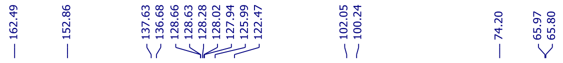

^{13}C NMR (126 MHz, $CDCl_3$)

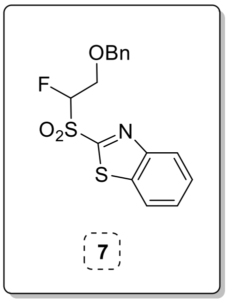

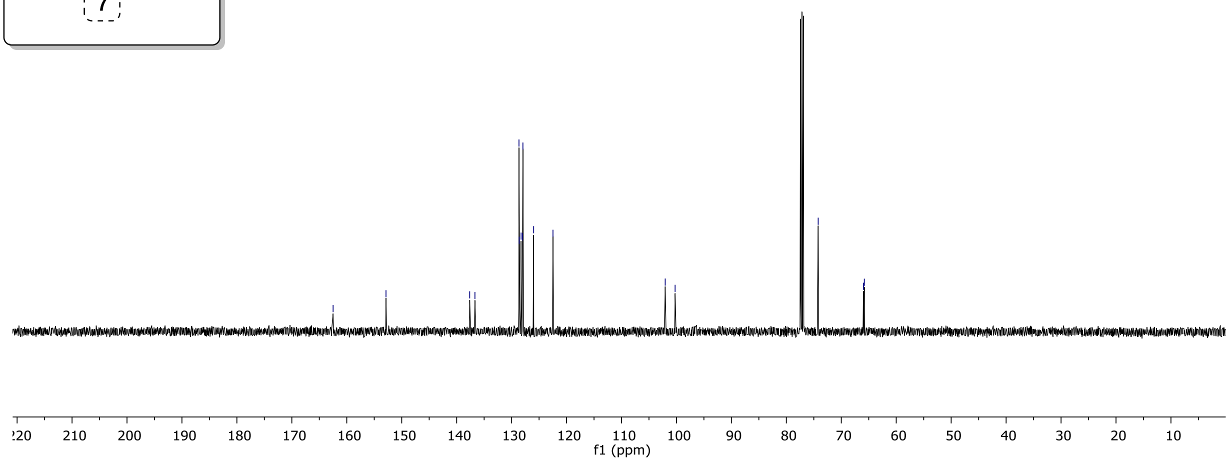

19F

^{19}F NMR (471 MHz, $CDCl_3$)

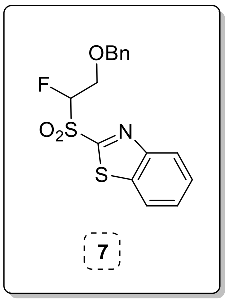

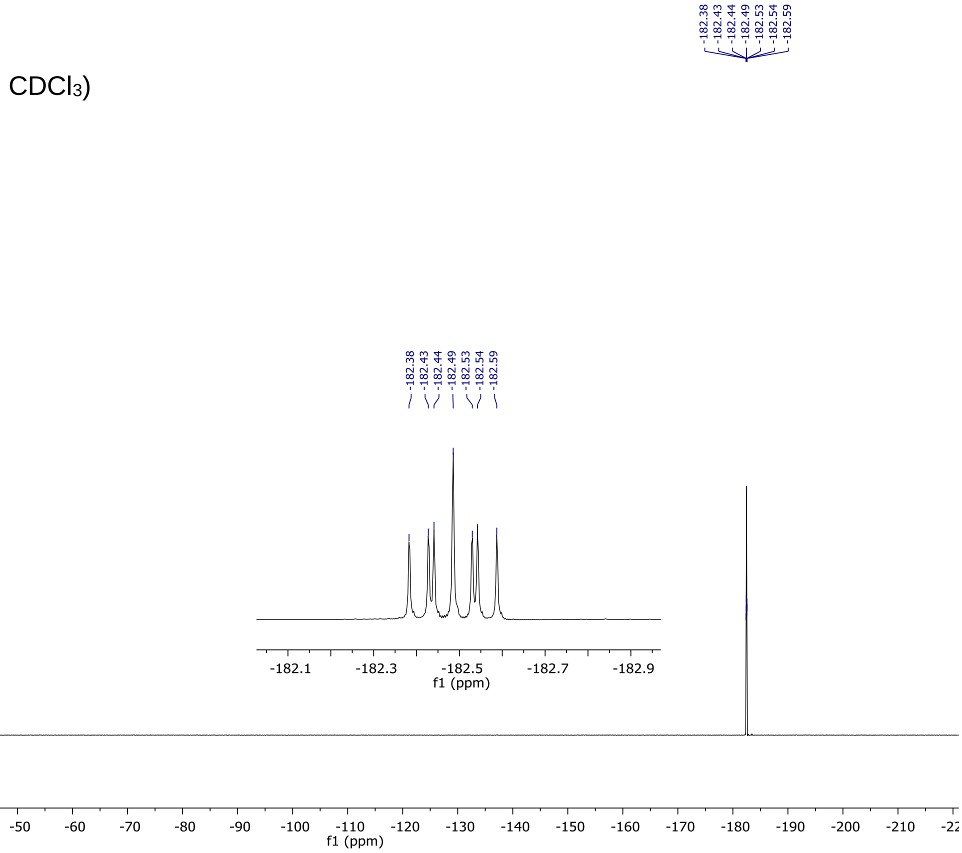

3-(2-Benzyloxy-1-fluoro-ethylidene)oxetane **(8)**

1H

^{1}H NMR ($CDCl_3$, 600 MHz)

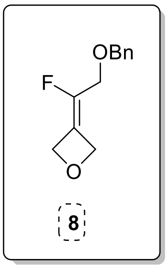

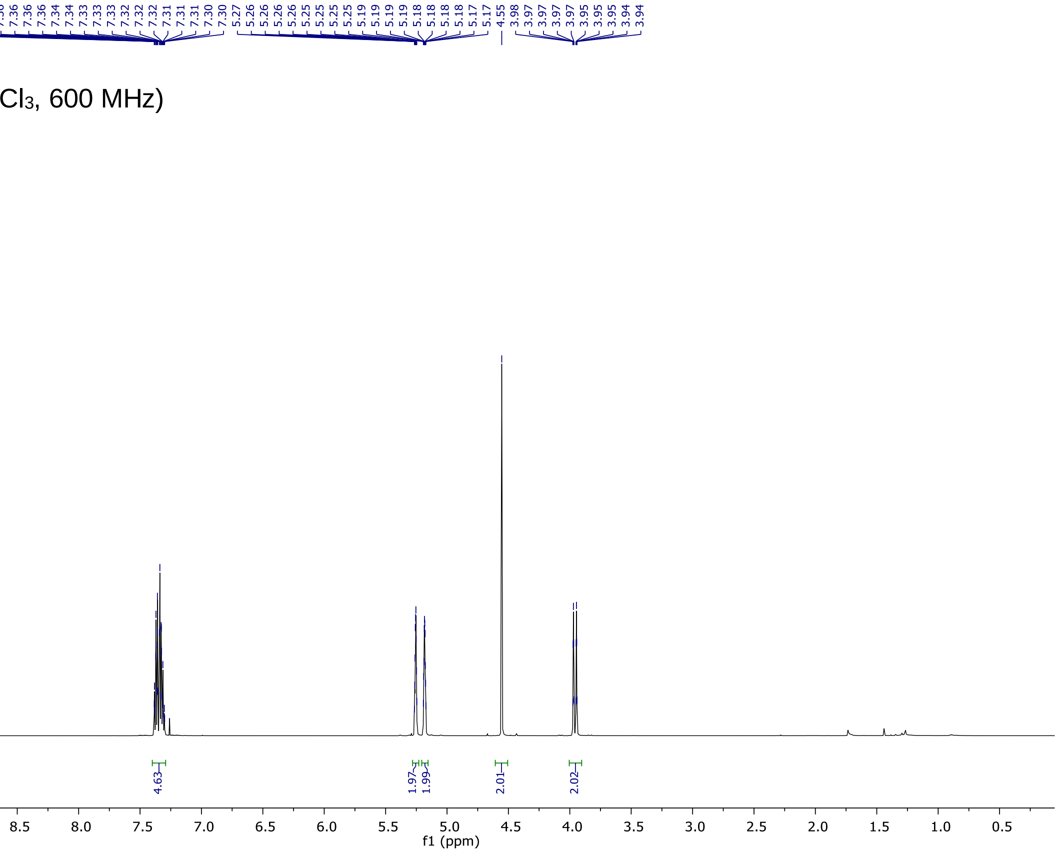

13C

^{13}C NMR (151 MHz, $CDCl_3$)

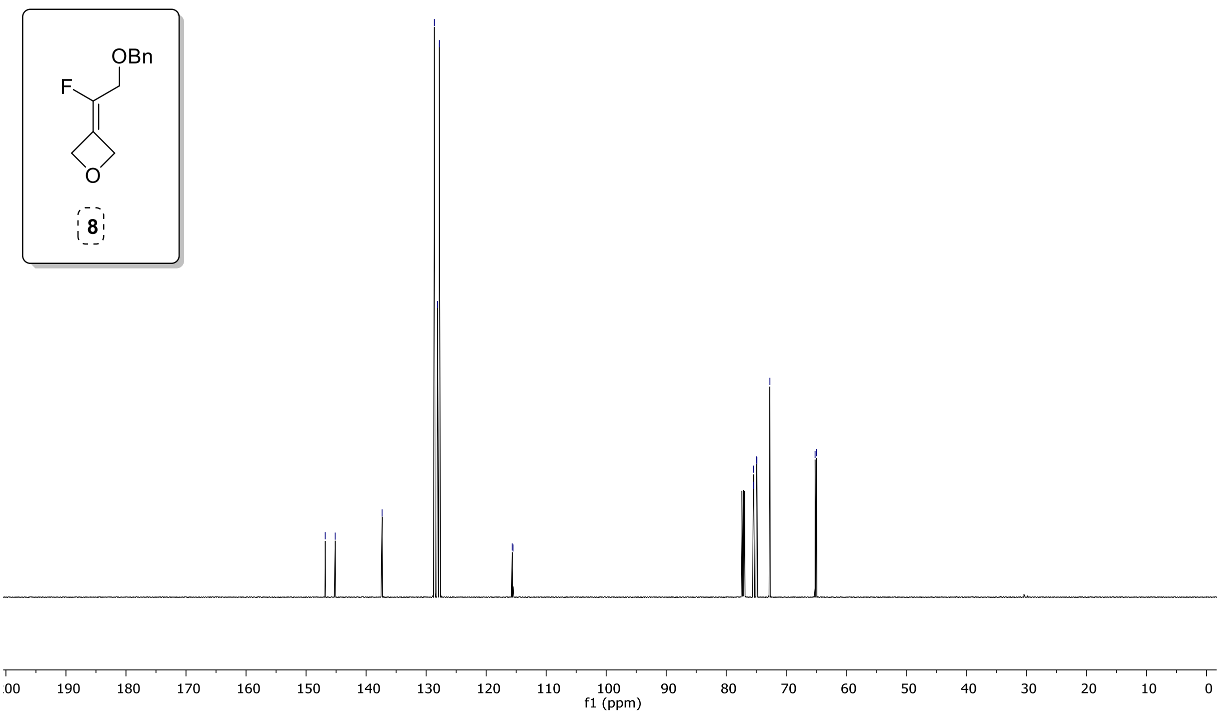

19F

^{19}F NMR (471 MHz, $CDCl_3$)

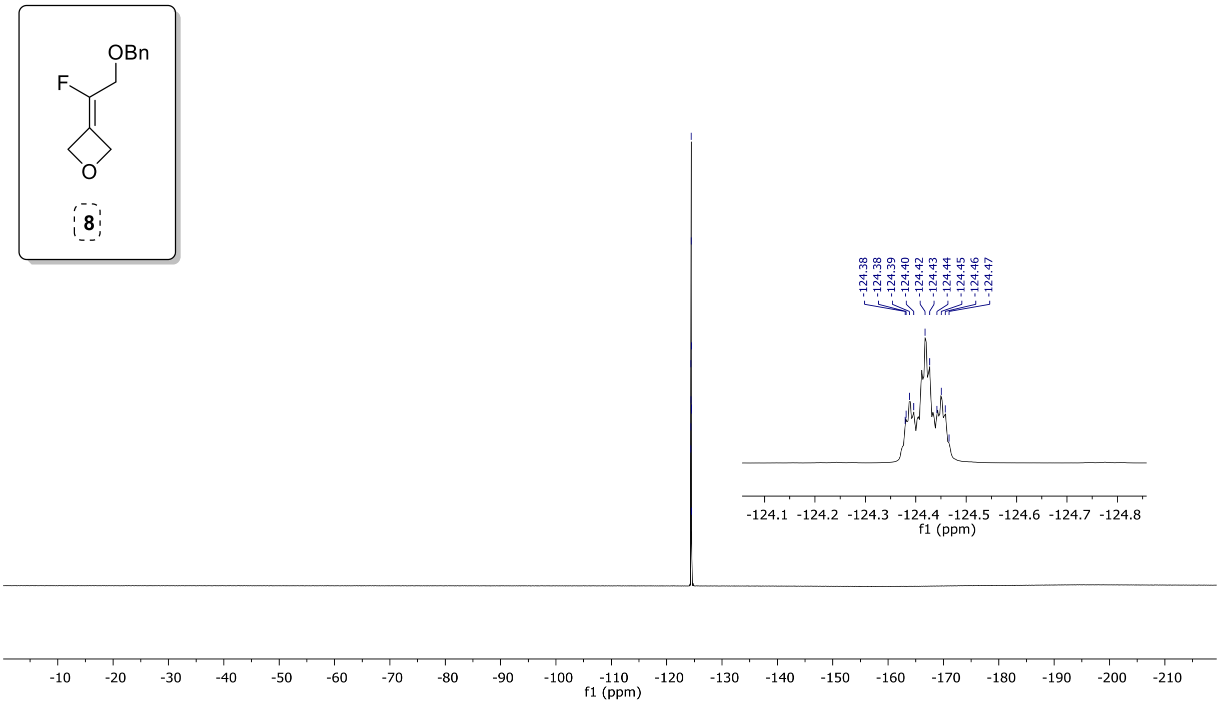

4-Benzyloxy-2-(bromomethyl)-3-fluoro-but-2-en-1-ol **(9)**

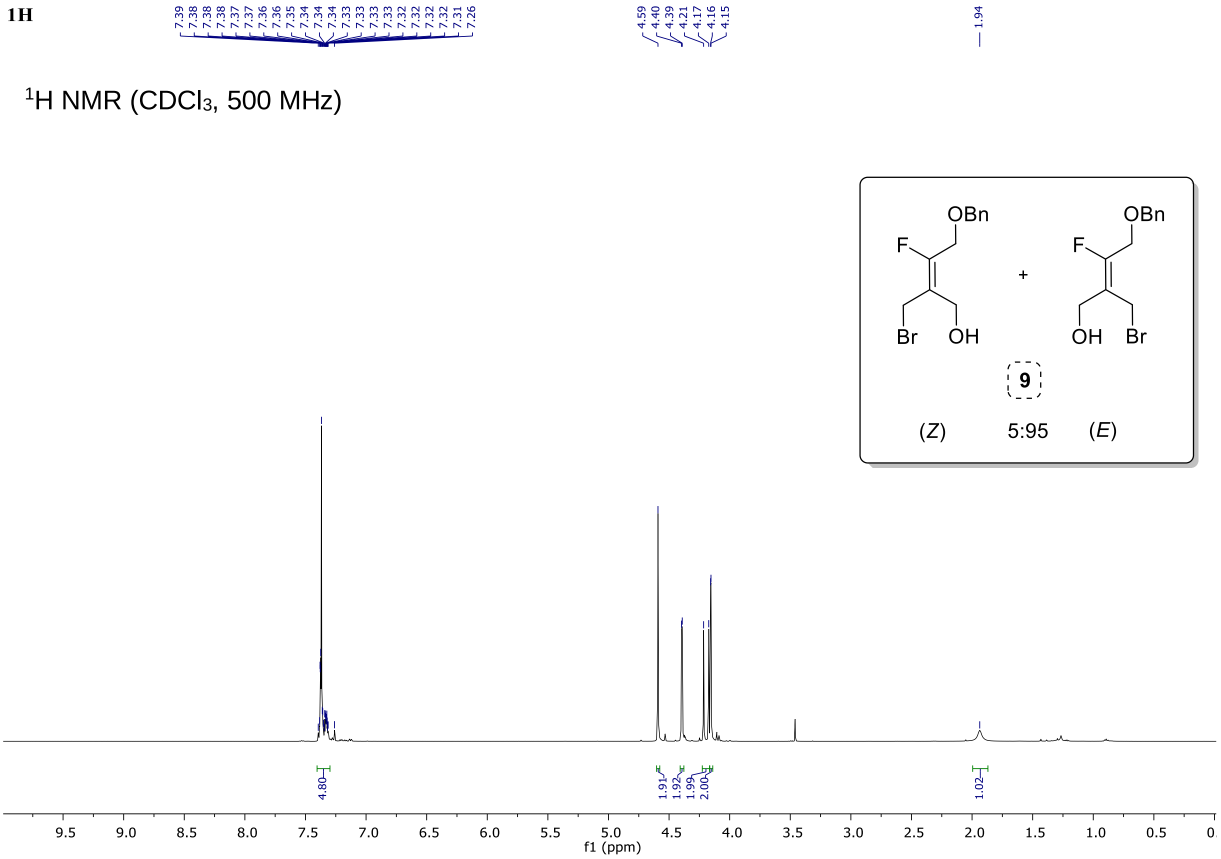

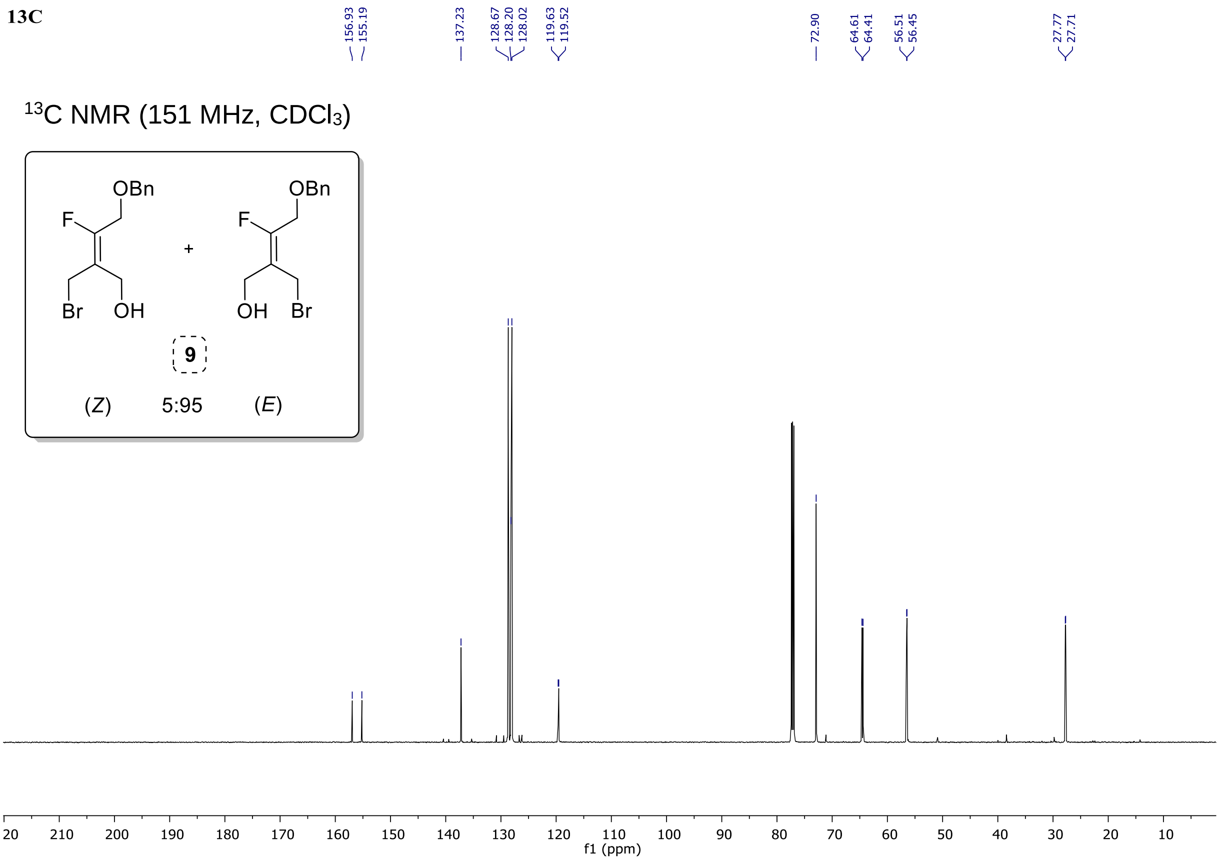

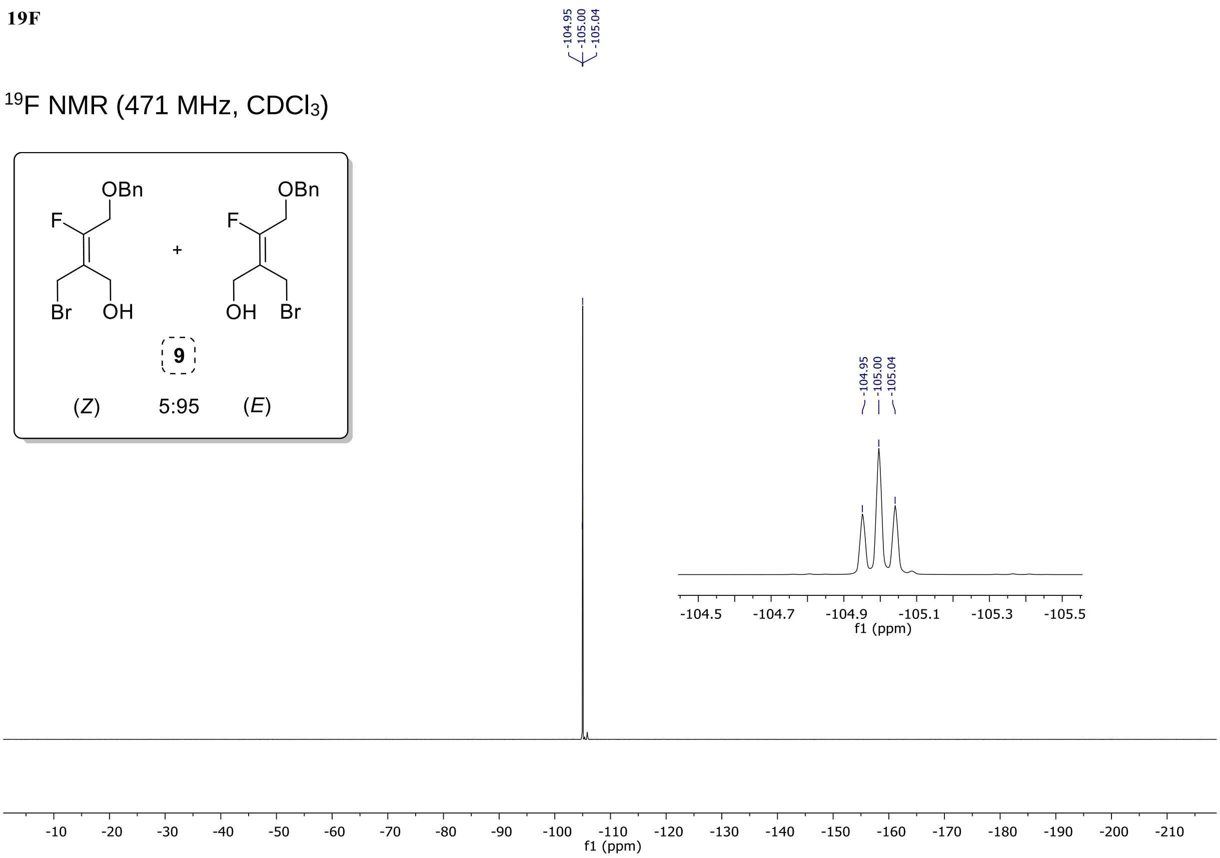

(4-Fluoro-2,5-dihydrofuran-3-yl)methanol **(10)**

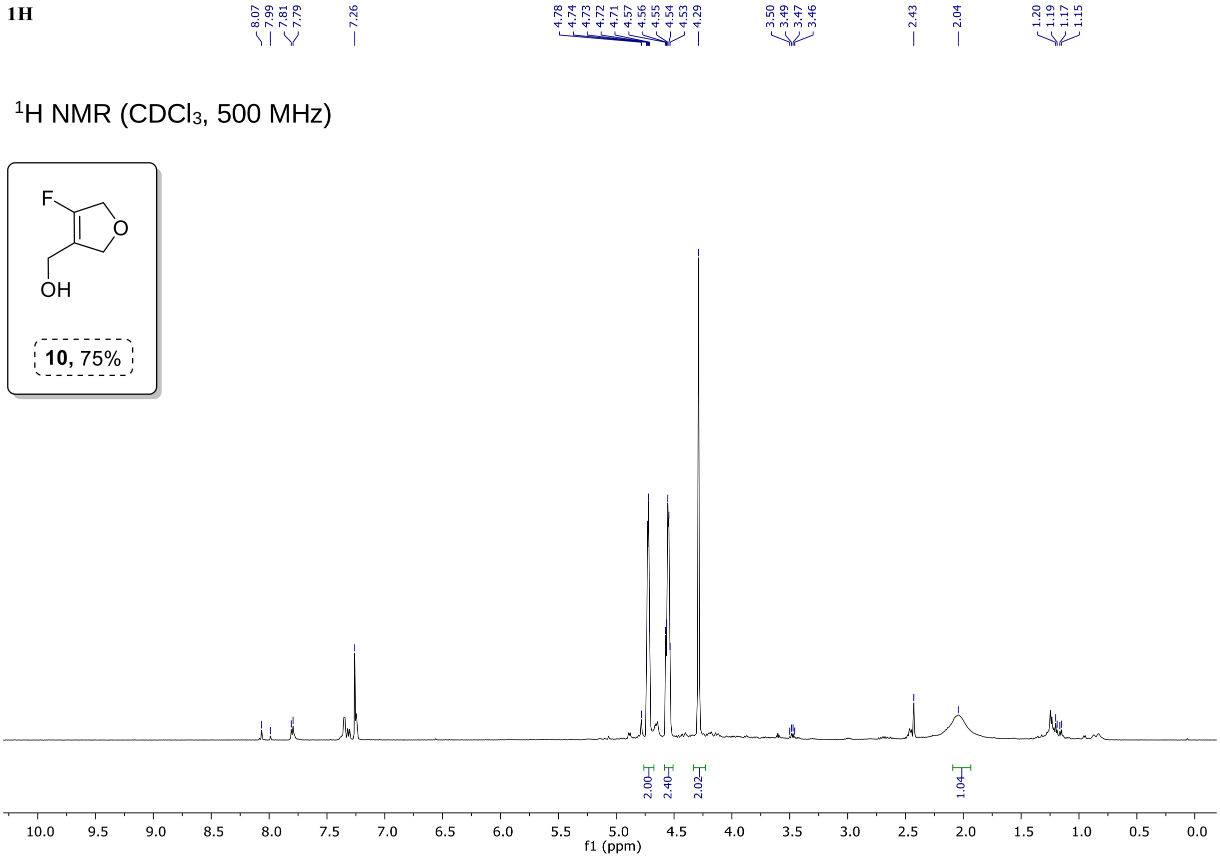

13C

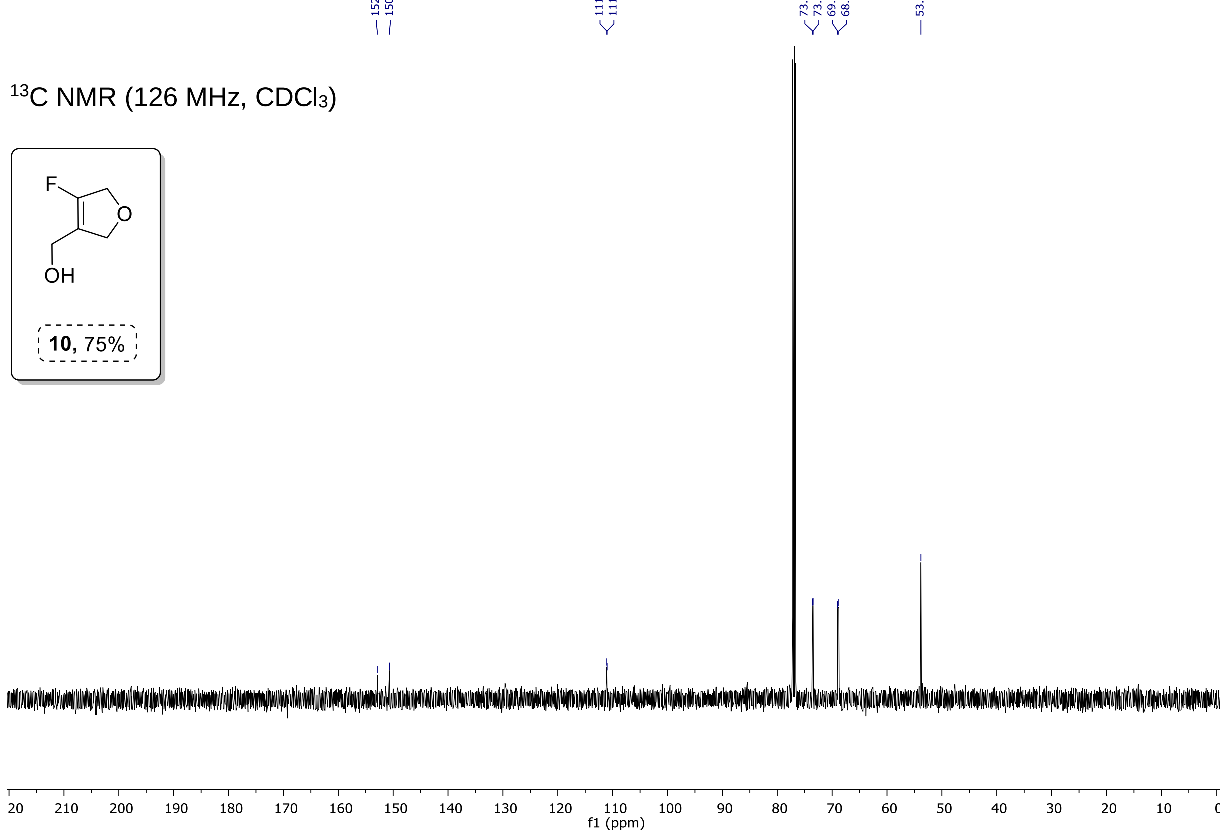
152.90
150.69
111.07
111.01
73.54
73.47
69.01
68.79
53.82
^{13}C NMR (126 MHz, $CDCl_3$)
F
O
OH
10, 75%
f1 (ppm)

19F

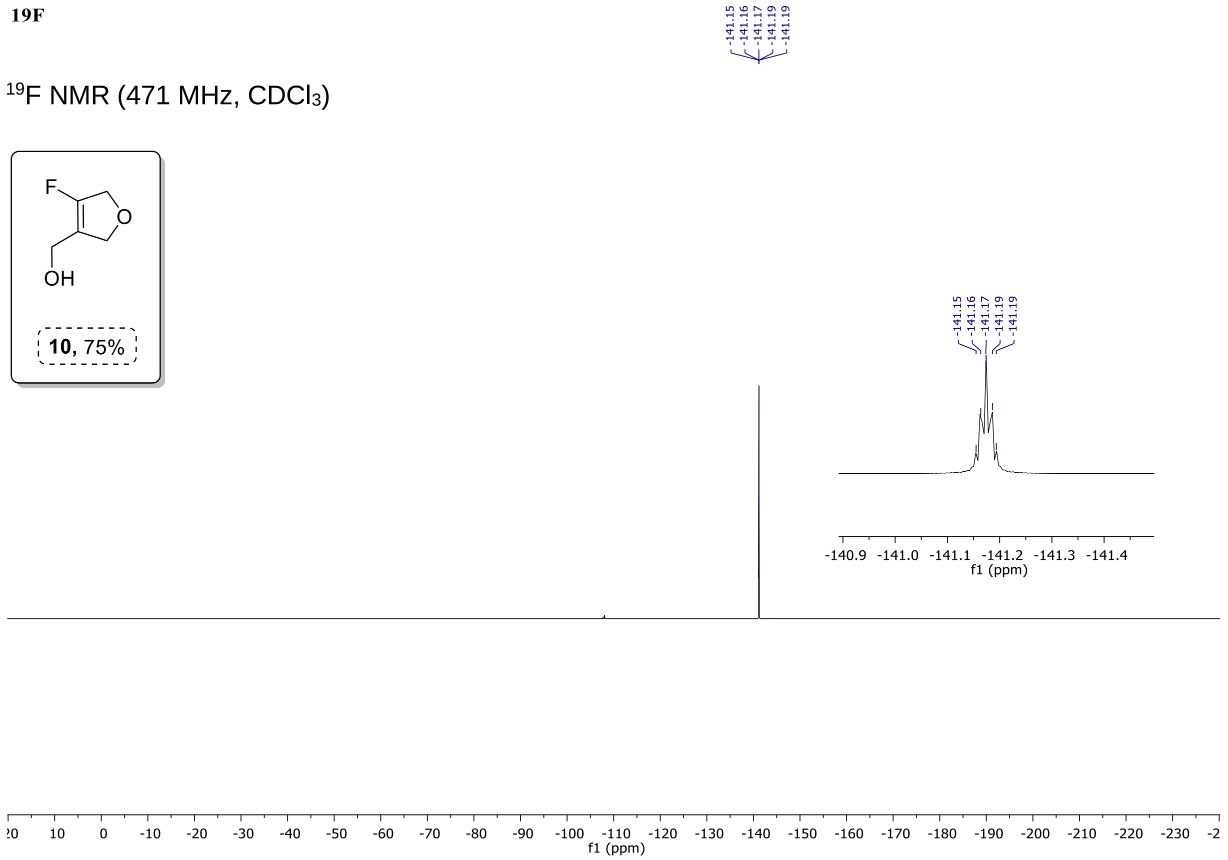
-141.15
-141.16
-141.17
-141.19
-141.19
^{19}F NMR (471 MHz, $CDCl_3$)
F
O
OH
10, 75%
-140.9 -141.0 -141.1 -141.2 -141.3 -141.4
f1 (ppm)
f1 (ppm)

Ethyl (*Z*)-4-bromo-2-fluoro-3-(hydroxymethyl)but-2-enoate **(*Z*-13)**

^{1}H NMR ($CDCl_3$, 500 MHz)

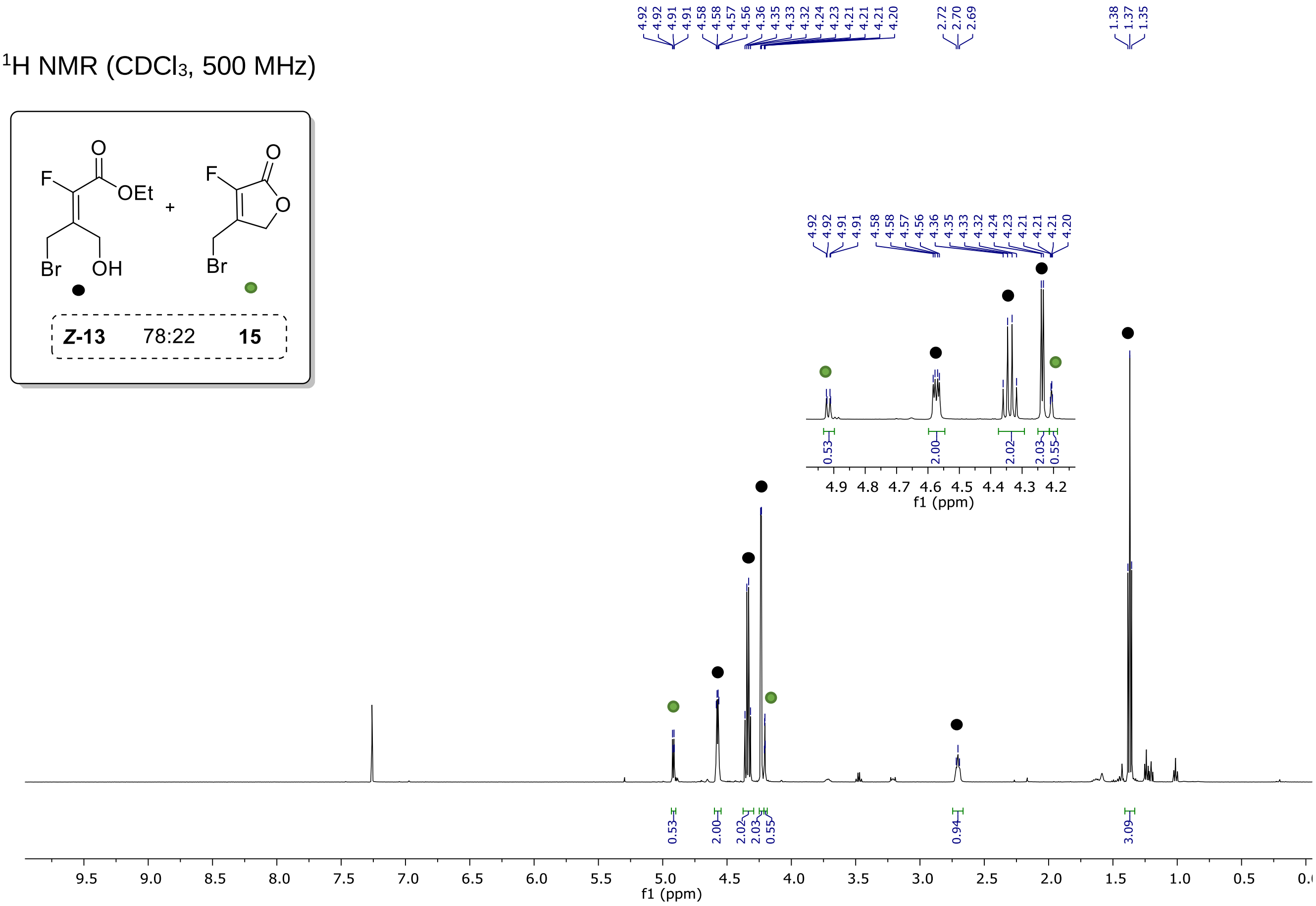

^{19}F NMR (471 MHz, $CDCl_3$)

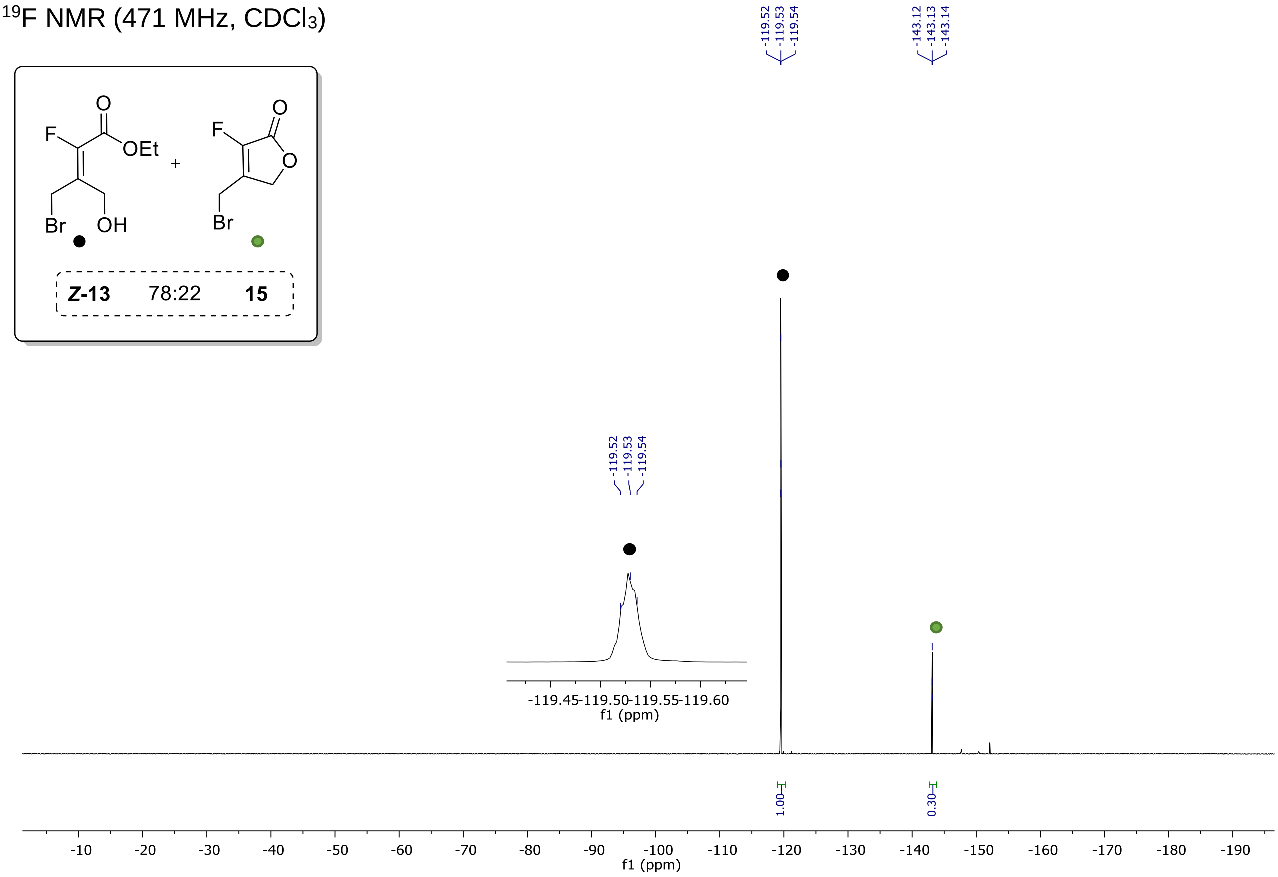

$^{19}F\{^1H\}$ NMR (471 MHz, $CDCl_3$)

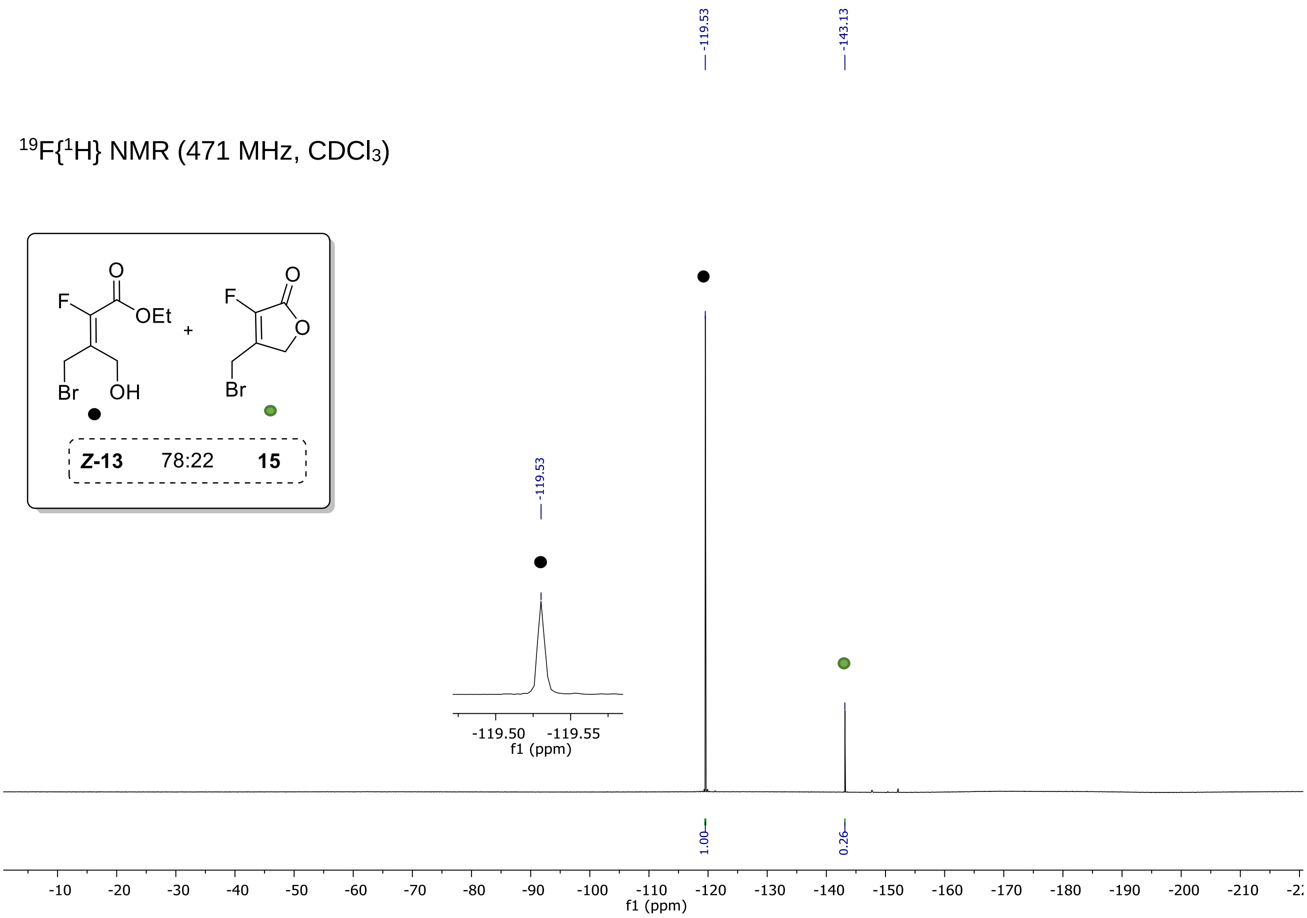

^{13}C NMR (126 MHz, $CDCl_3$)

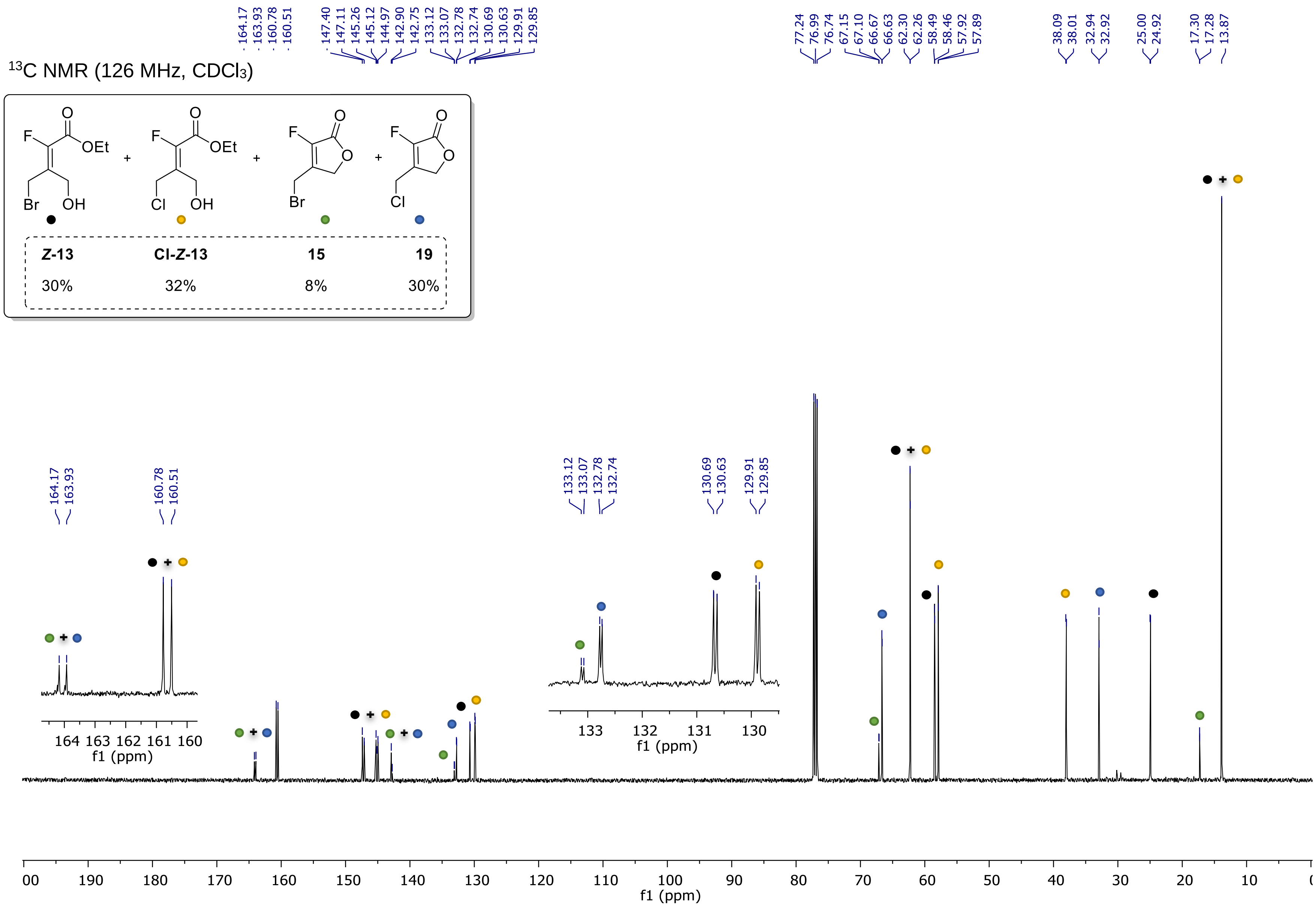

Note: the ^{13}C NMR spectrum for ***Z*-13** and **19** was obtained after an opening reaction with TBAB and HBr/AcOH that gave a mixture of ***Z*-13**, **15**, **19**, and a fourth compound which was identified as **Cl-*Z*-13**.

4-(Chloromethyl)-3-fluoro-2(5*H*)-furanone **(19)**

^{1}H NMR (CDCl$_3$, 500 MHz)

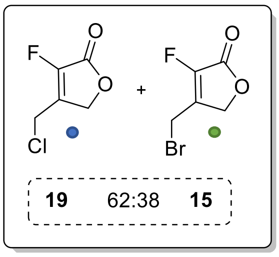

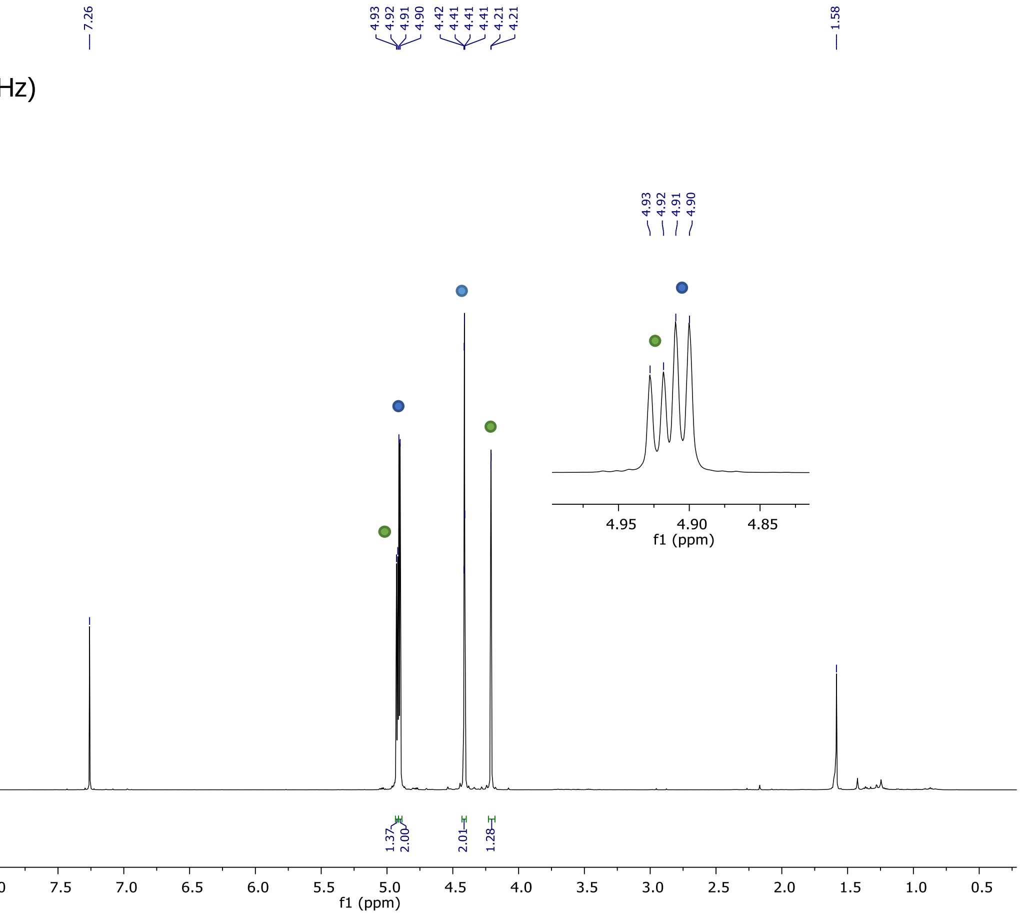

^{19}F NMR (471 MHz, CDCl$_3$)

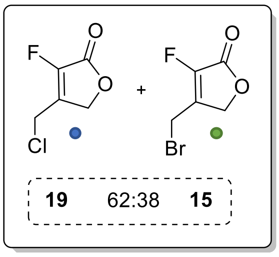

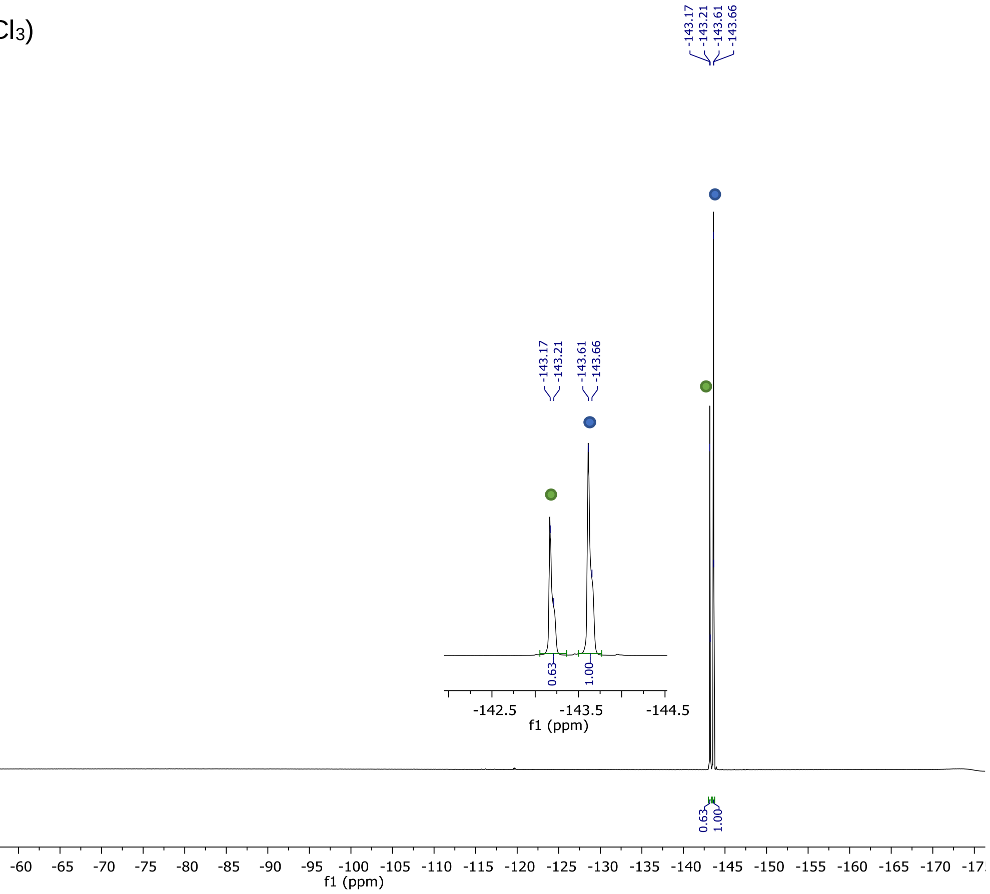

4-(Azidomethyl)-3-fluoro-2(5*H*)-furanone **(20)**

^{1}H NMR ($CDCl_3$, 500 MHz)

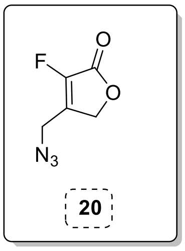

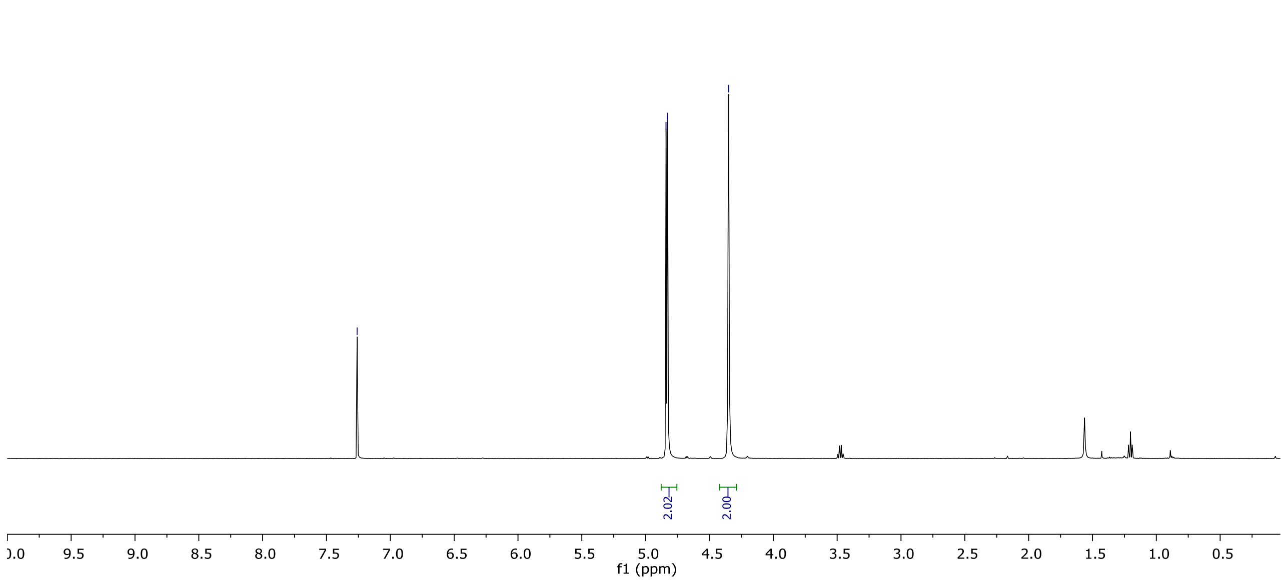

^{13}C NMR (126 MHz, $CDCl_3$)

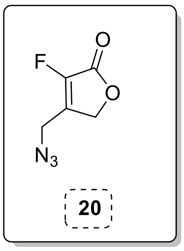

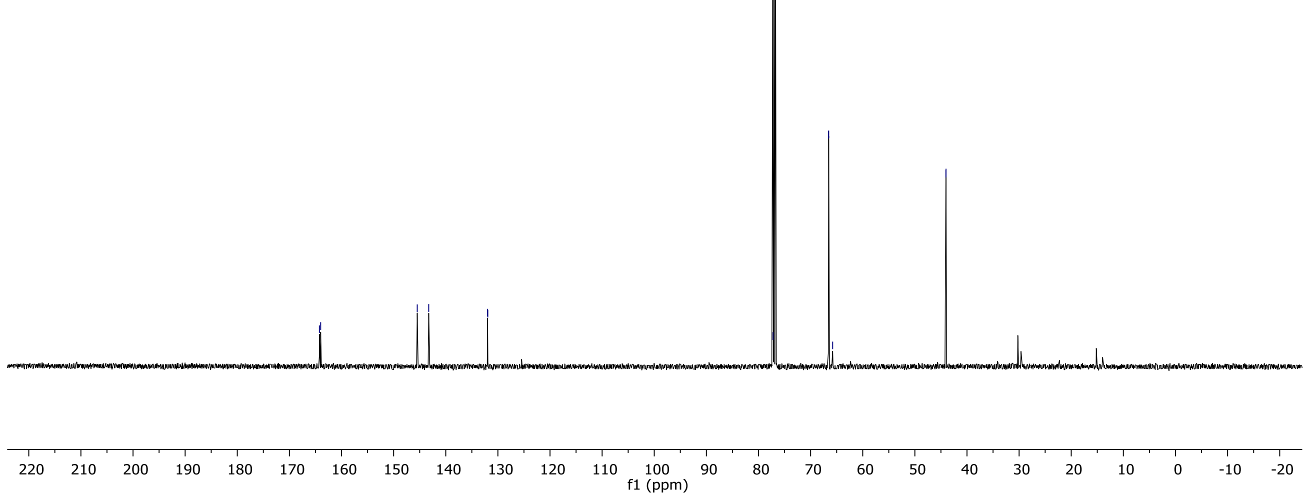

^{19}F NMR (471 MHz, $CDCl_3$)

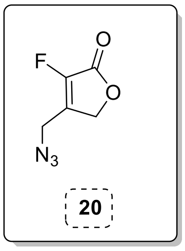

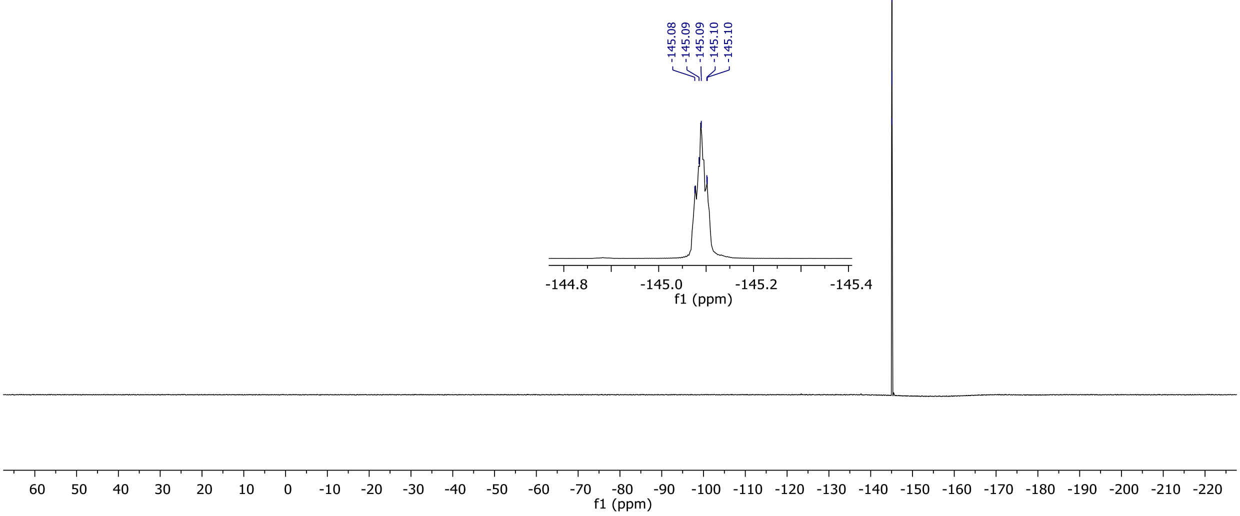

Diethyl [(4-fluoro-2,5-dihydro-5-oxo-3-furanyl)methyl]-phosphonate **(21)**

^{1}H NMR ($CDCl_3$, 600 MHz)

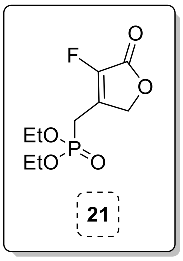

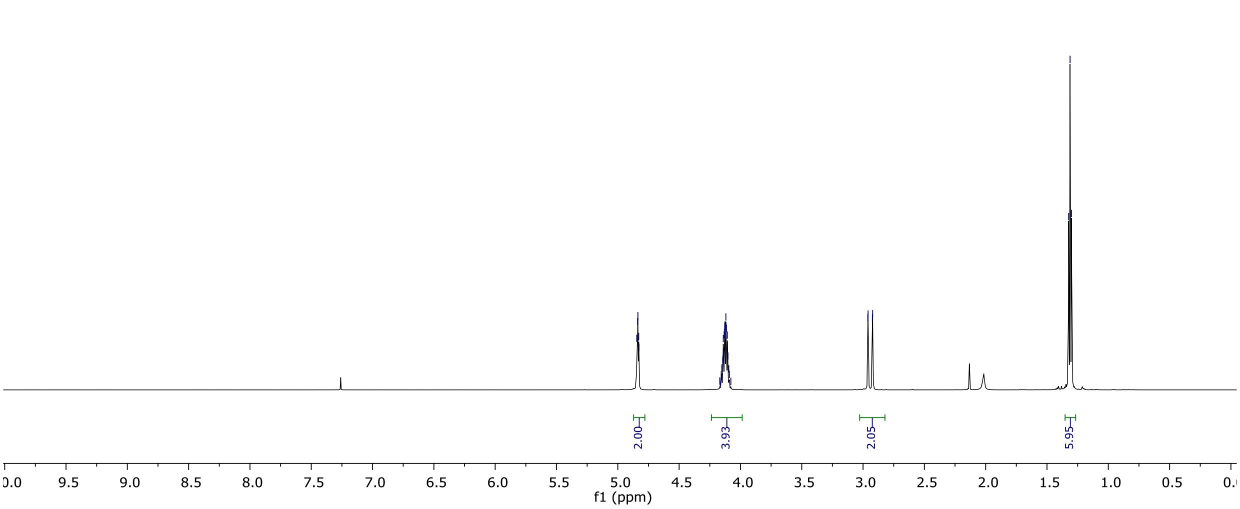

^{13}C NMR (151 MHz, $CDCl_3$)

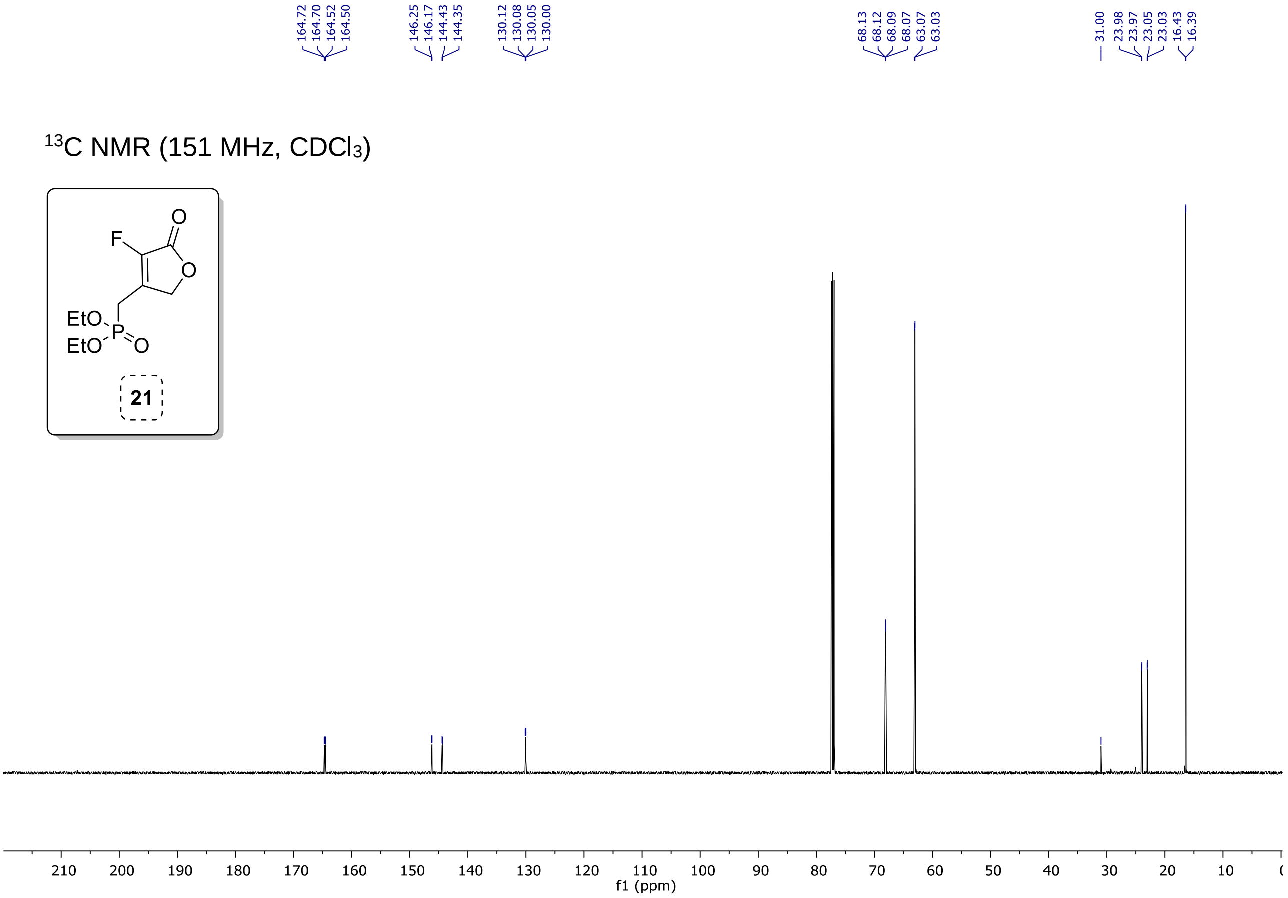

^{19}F NMR (565 MHz, $CDCl_3$)

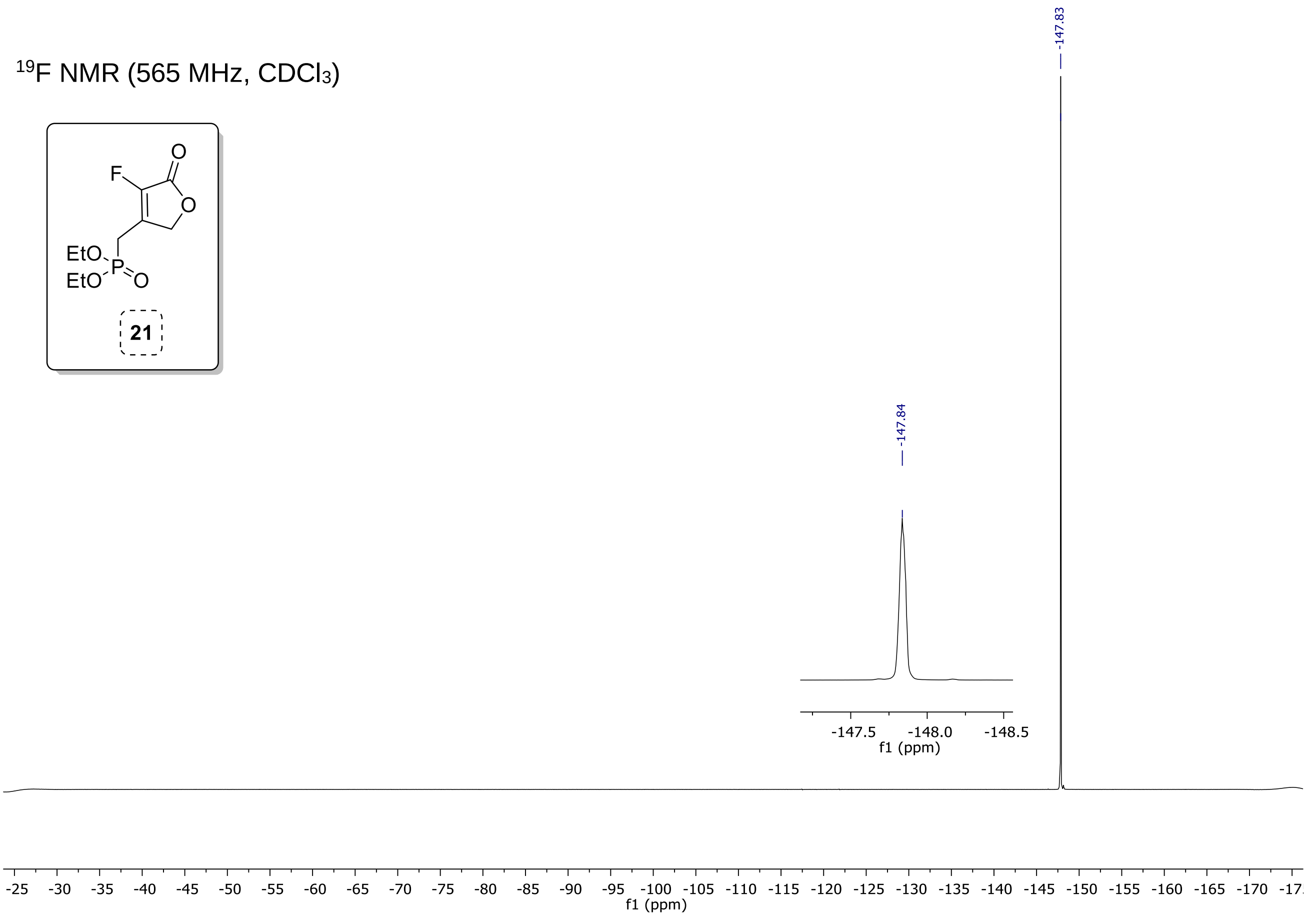

^{31}P NMR (243 MHz, $CDCl_3$)

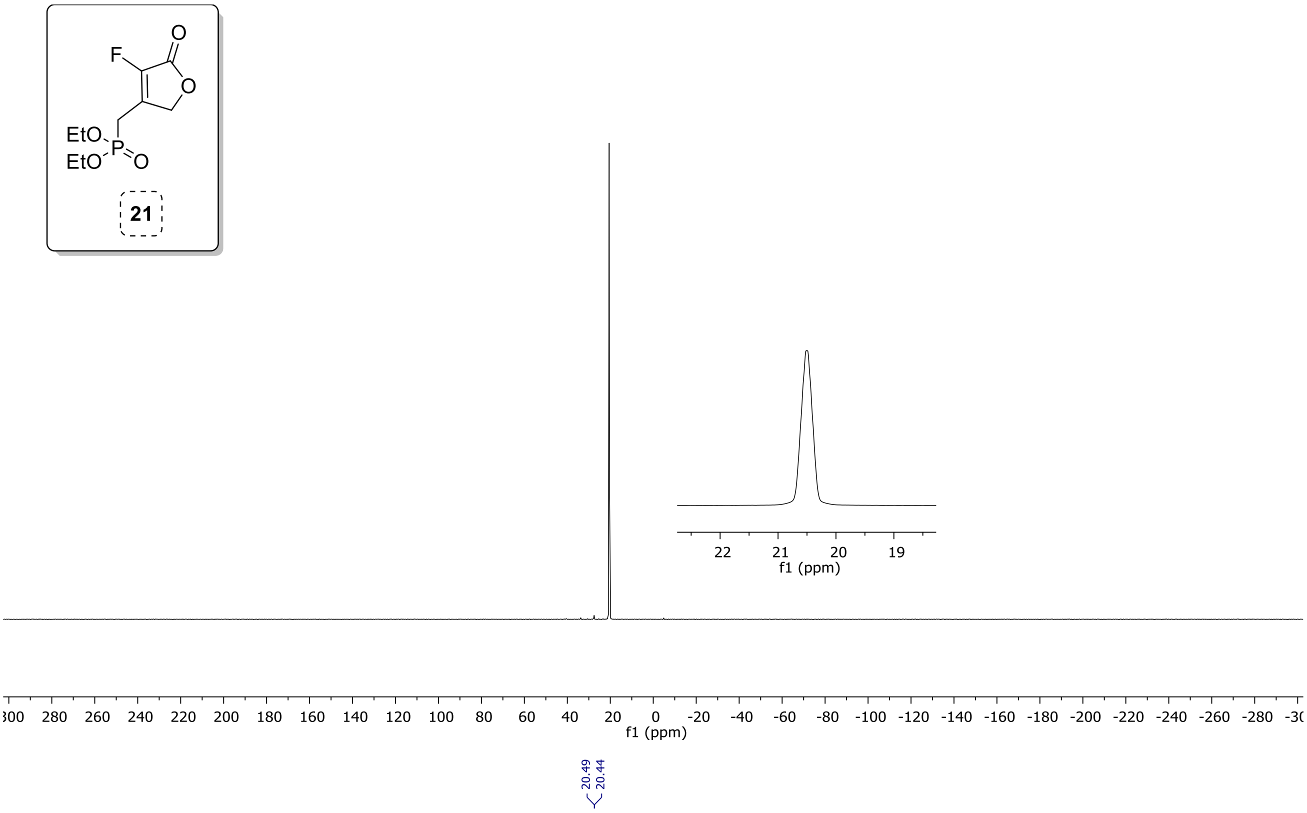

$^{31}P\{^1H\}$ NMR (243 MHz, $CDCl_3$)

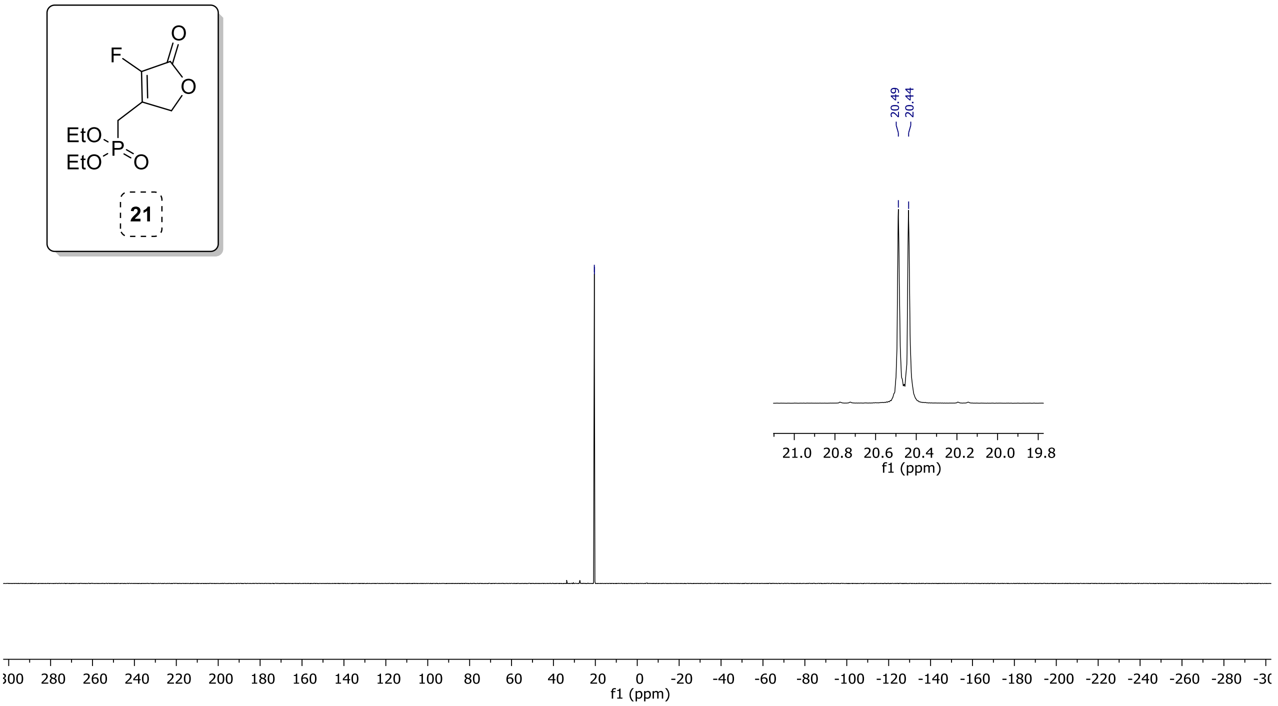

Diethyl (*Z*)-[3-fluoro-4-hydroxy-2-(hydroxymethyl)-but-2-en-1-yl]-phosphonate **(22)**

^{1}H NMR ($CDCl_3$, 500 MHz)

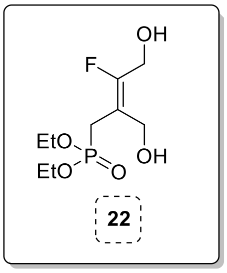

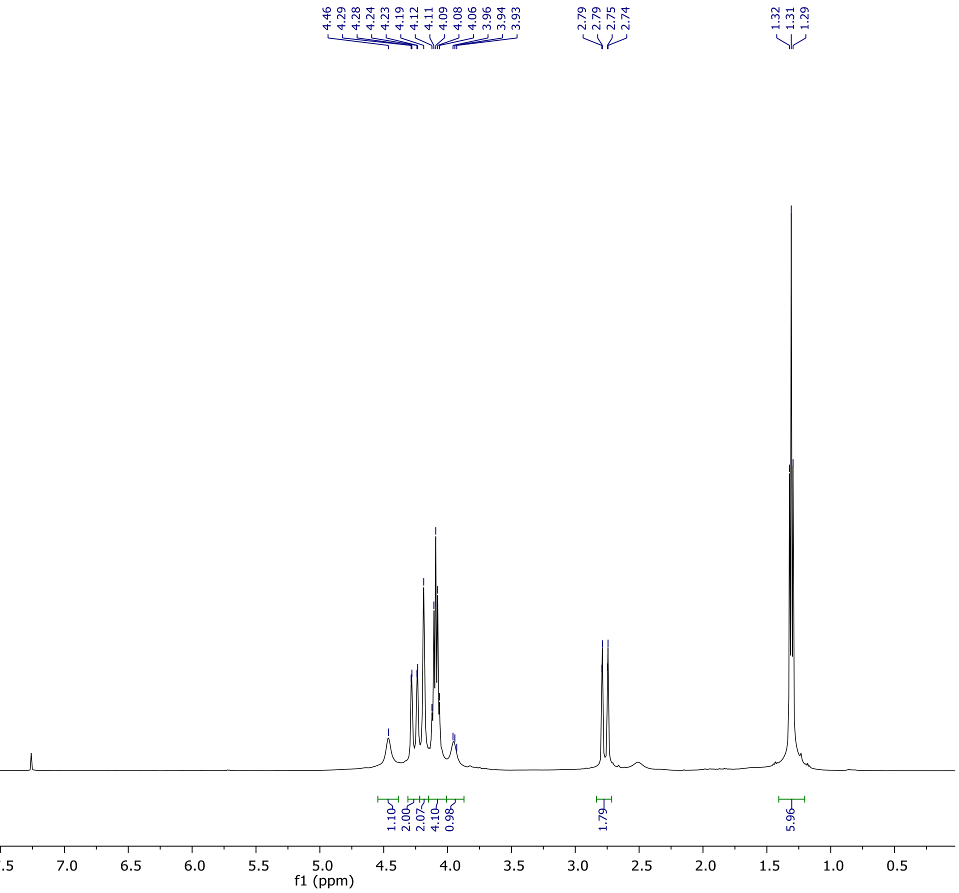

^{13}C NMR (126 MHz, $CDCl_3$)

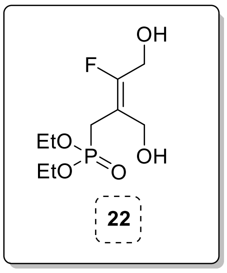

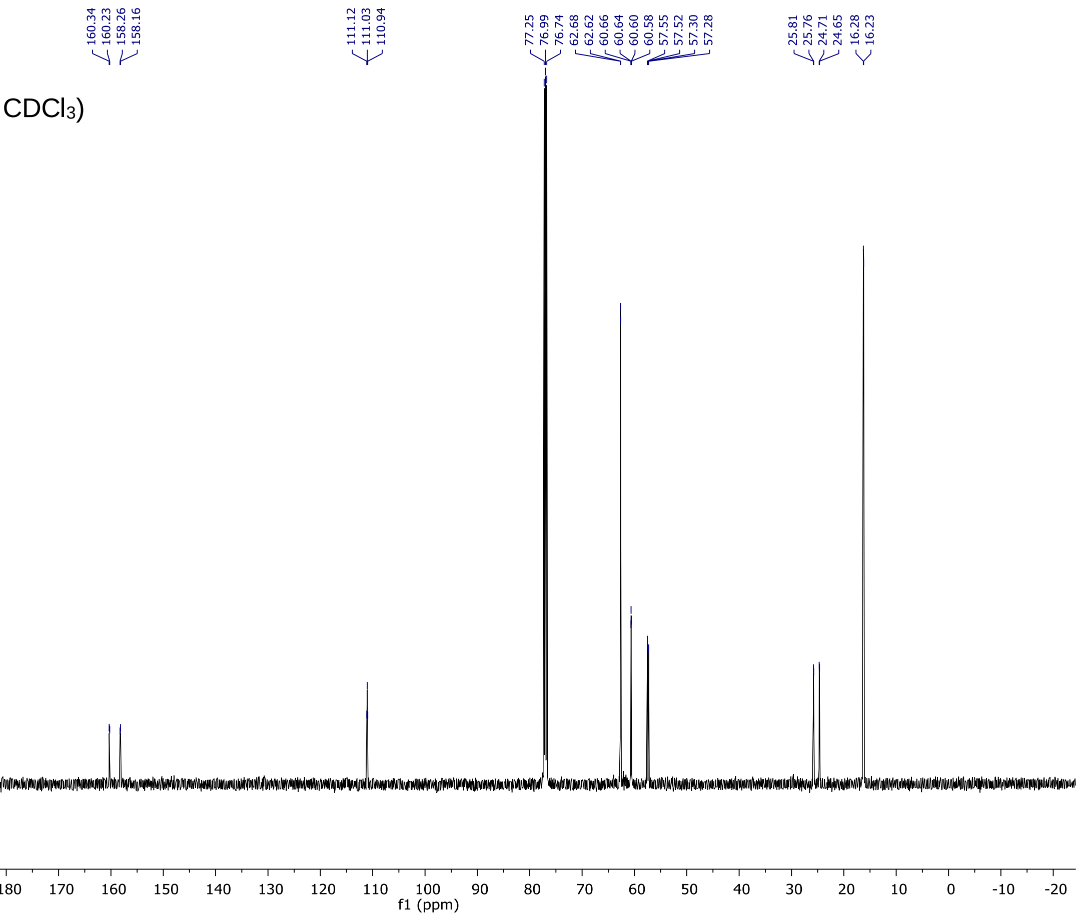

^{19}F NMR (471 MHz, $CDCl_3$)

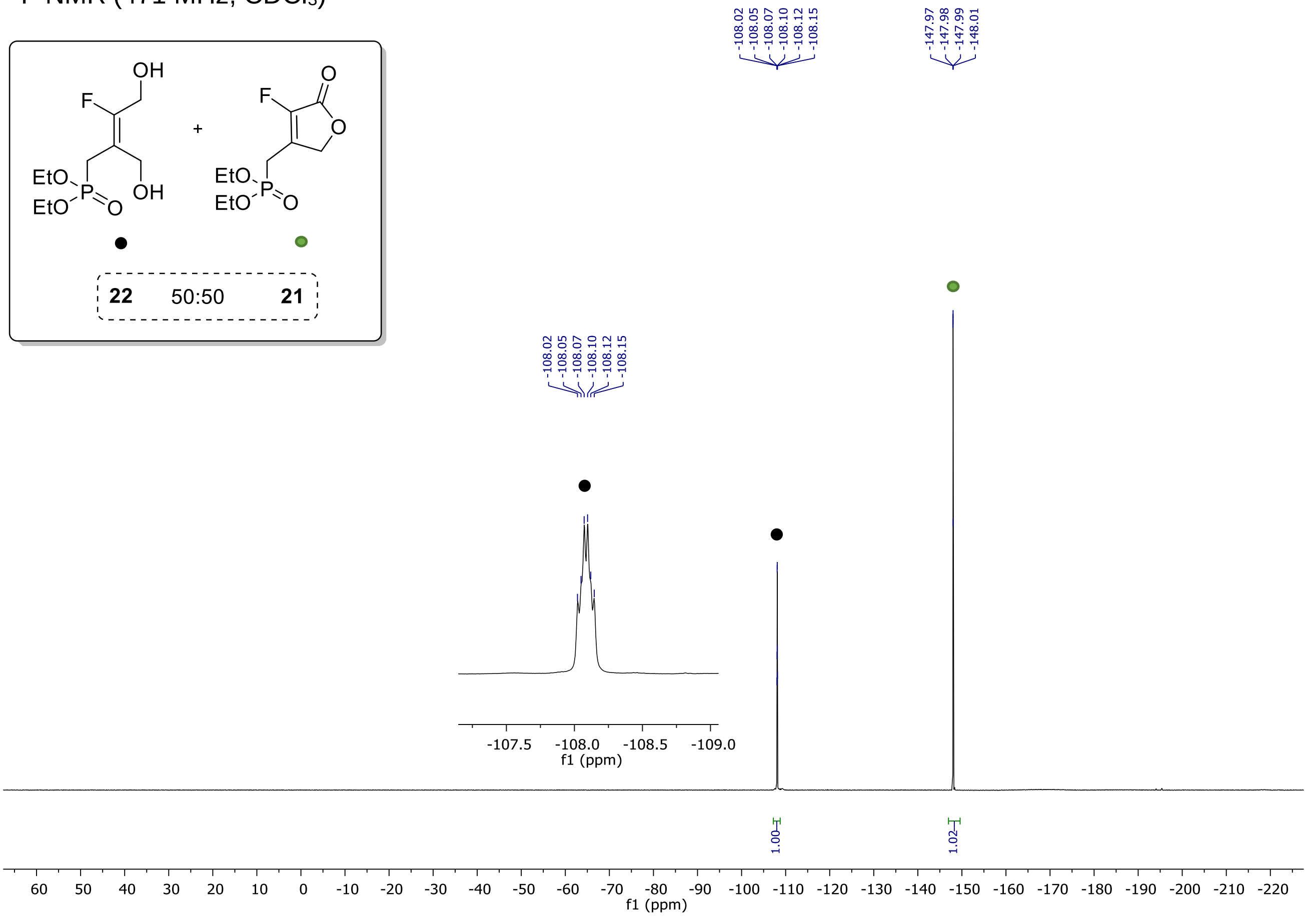

$^{31}P\{^1H\}$ NMR (243 MHz, $CDCl_3$)

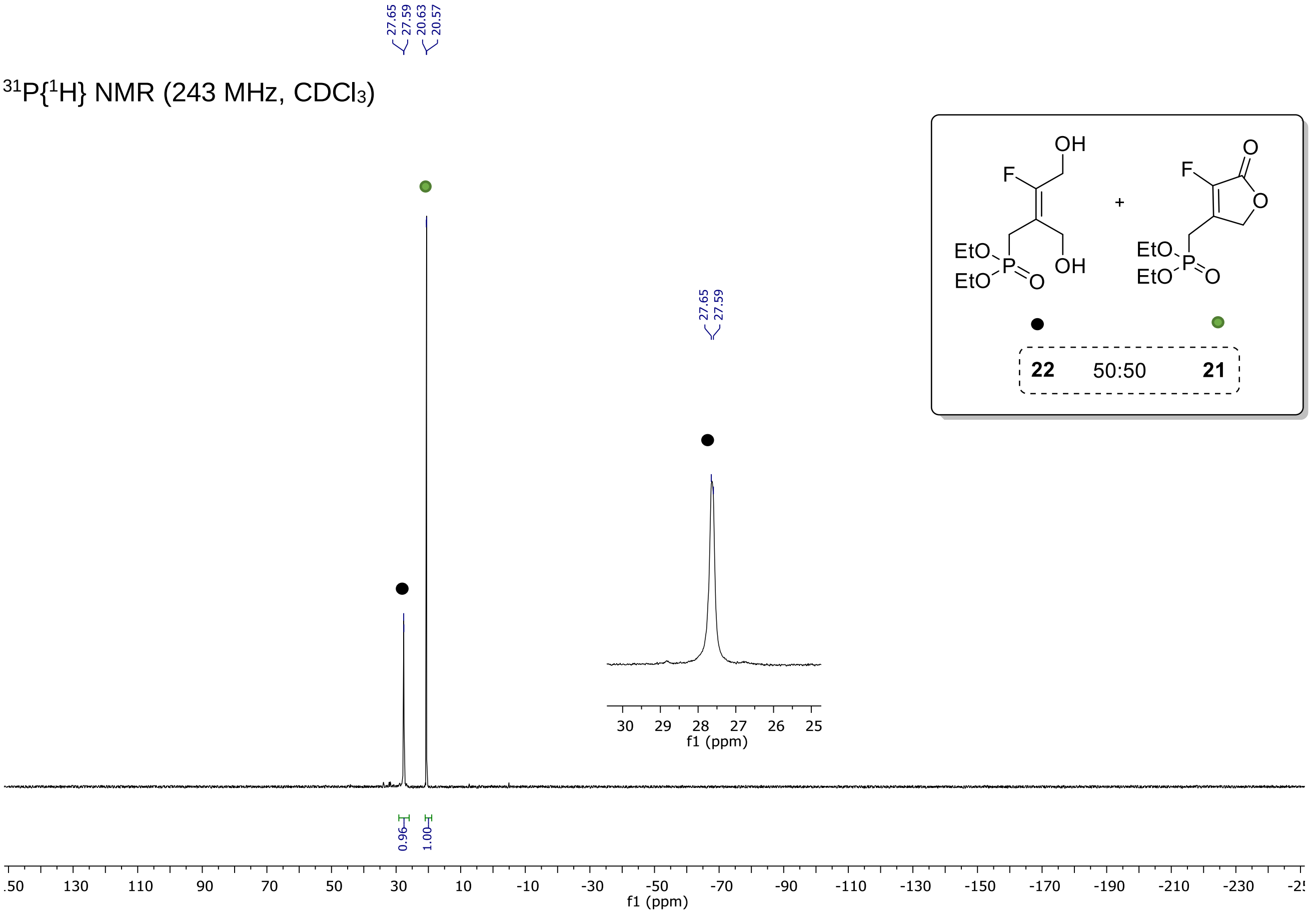

N-[(*E*)-2-Fluoro-3-(fluoromethyl)-4-hydroxy-but-2-enyl] phthalimide **(*E*-23)**

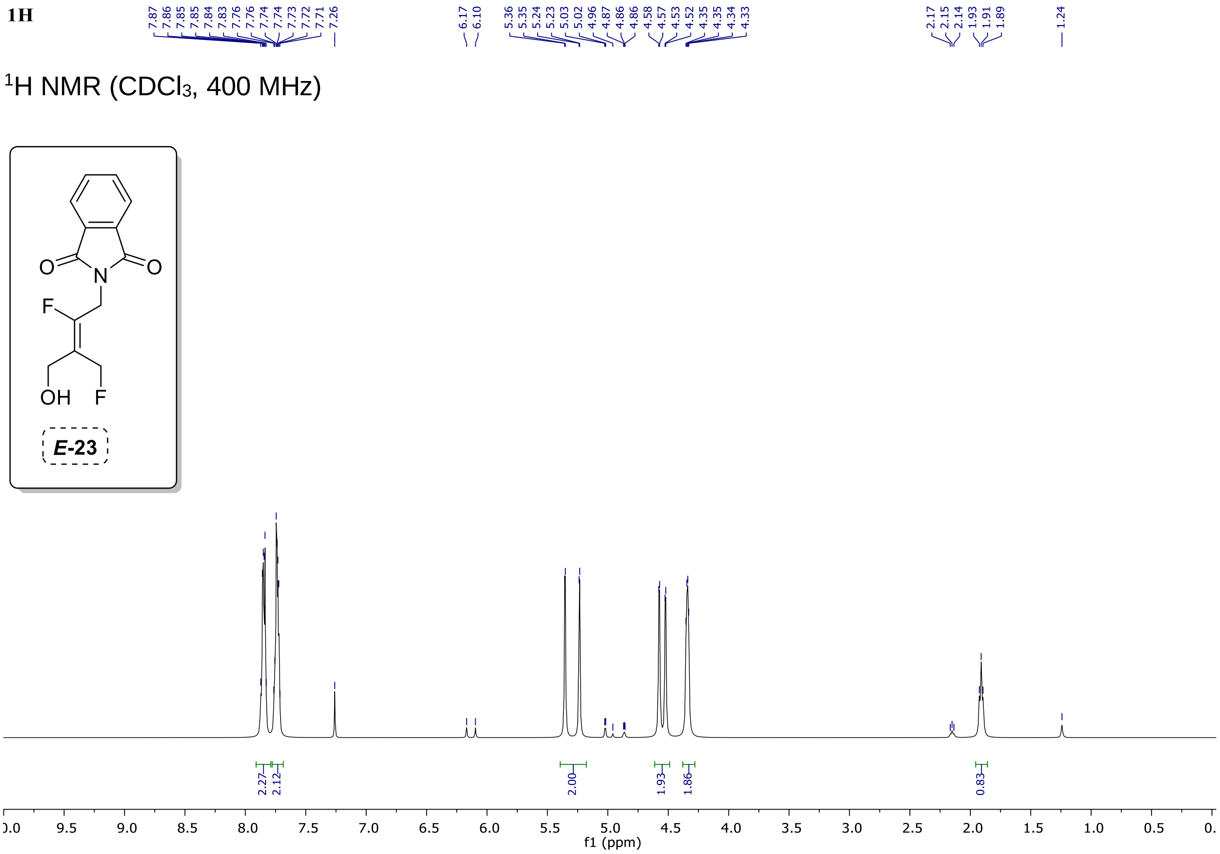

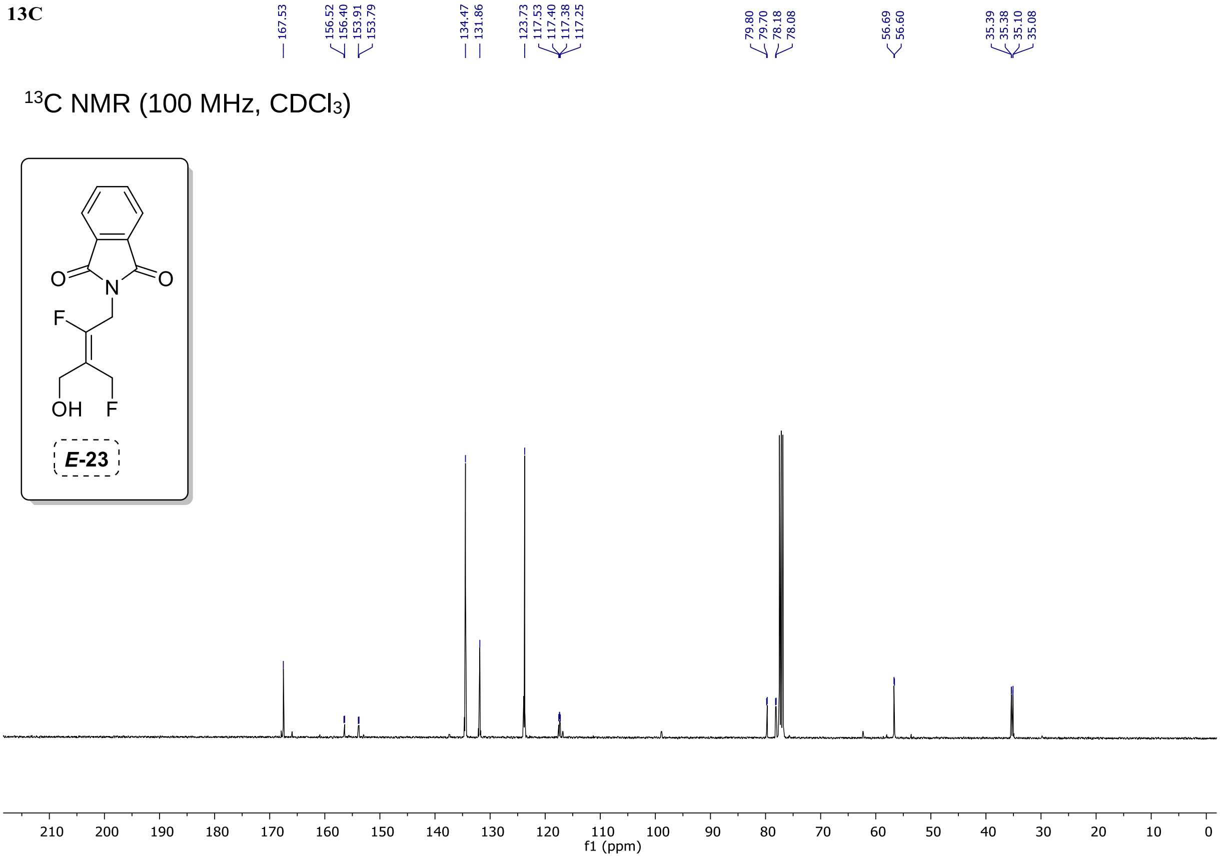

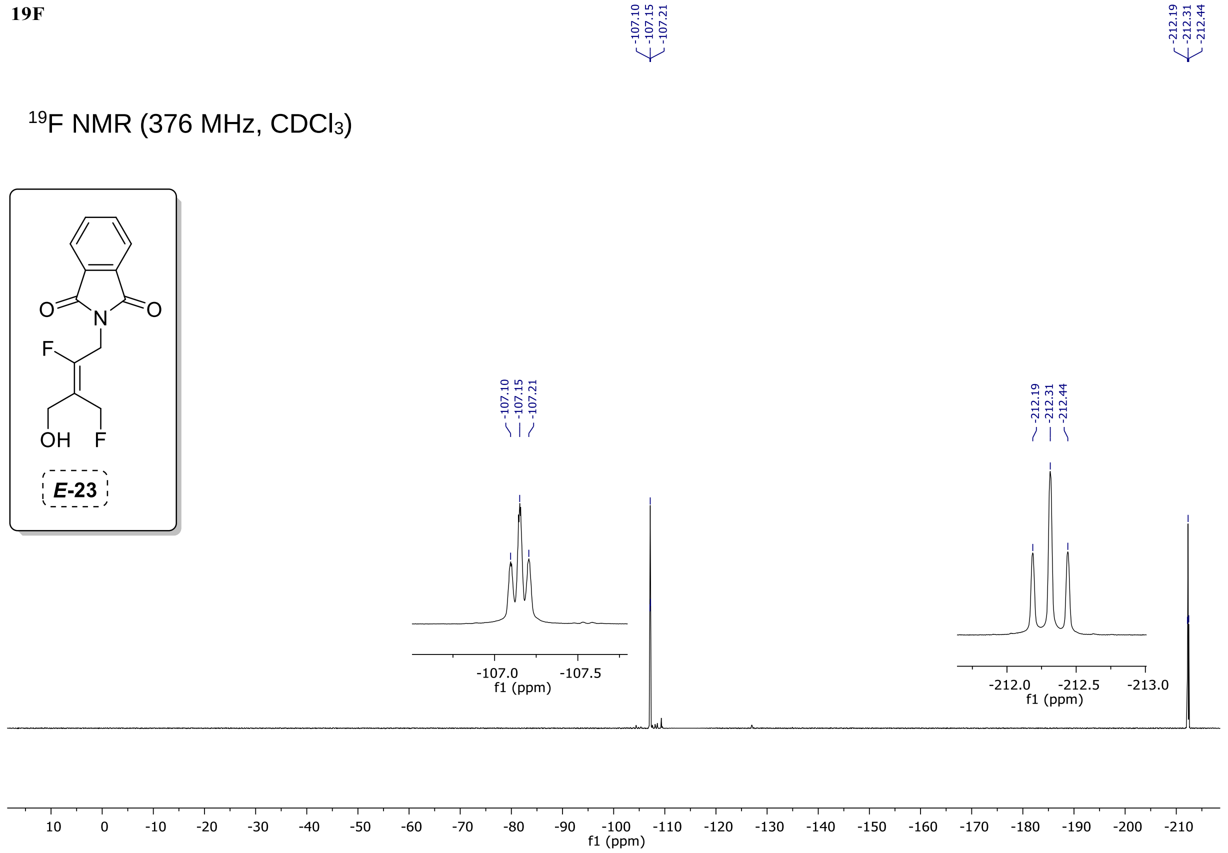

N-[(*Z*)-3-(Azidomethyl)-2-fluoro-4-hydroxy-but-2-enyl] phthalimide (***Z*-24)**

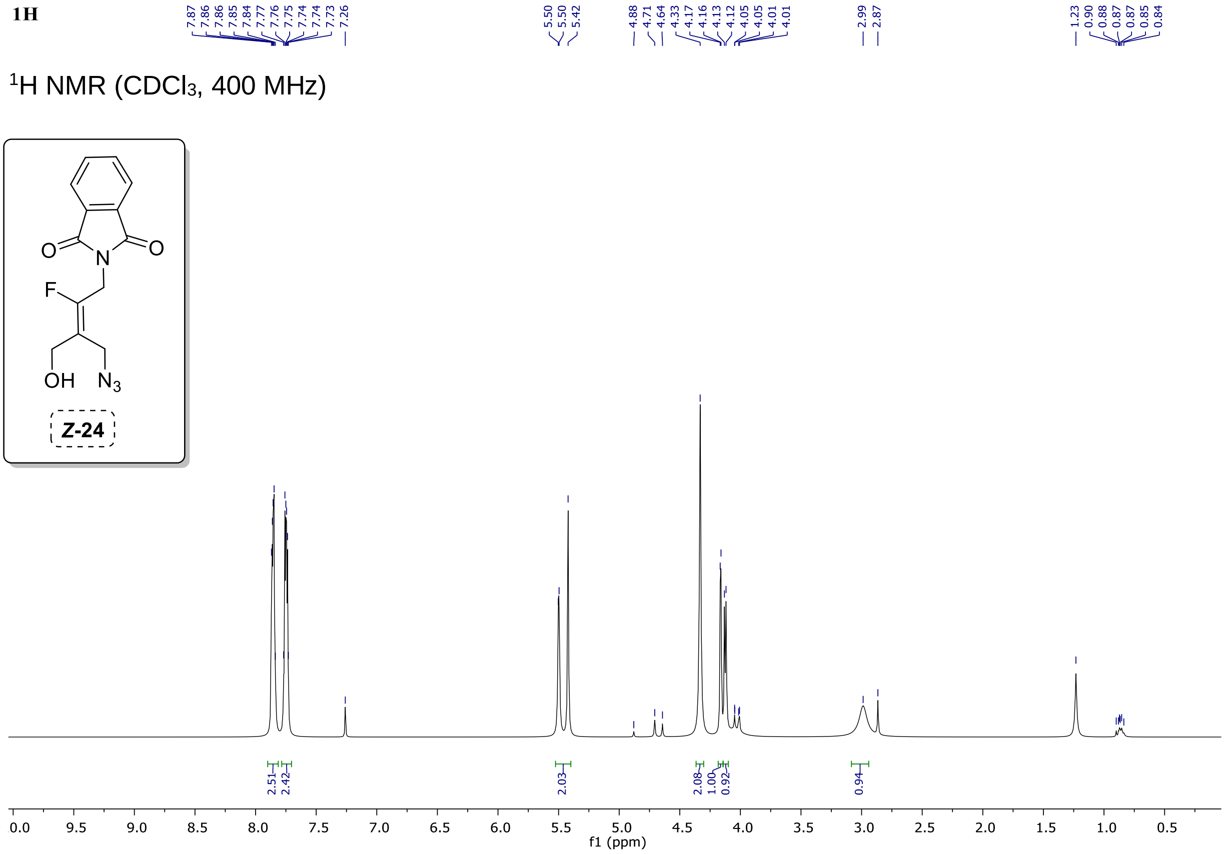

13C

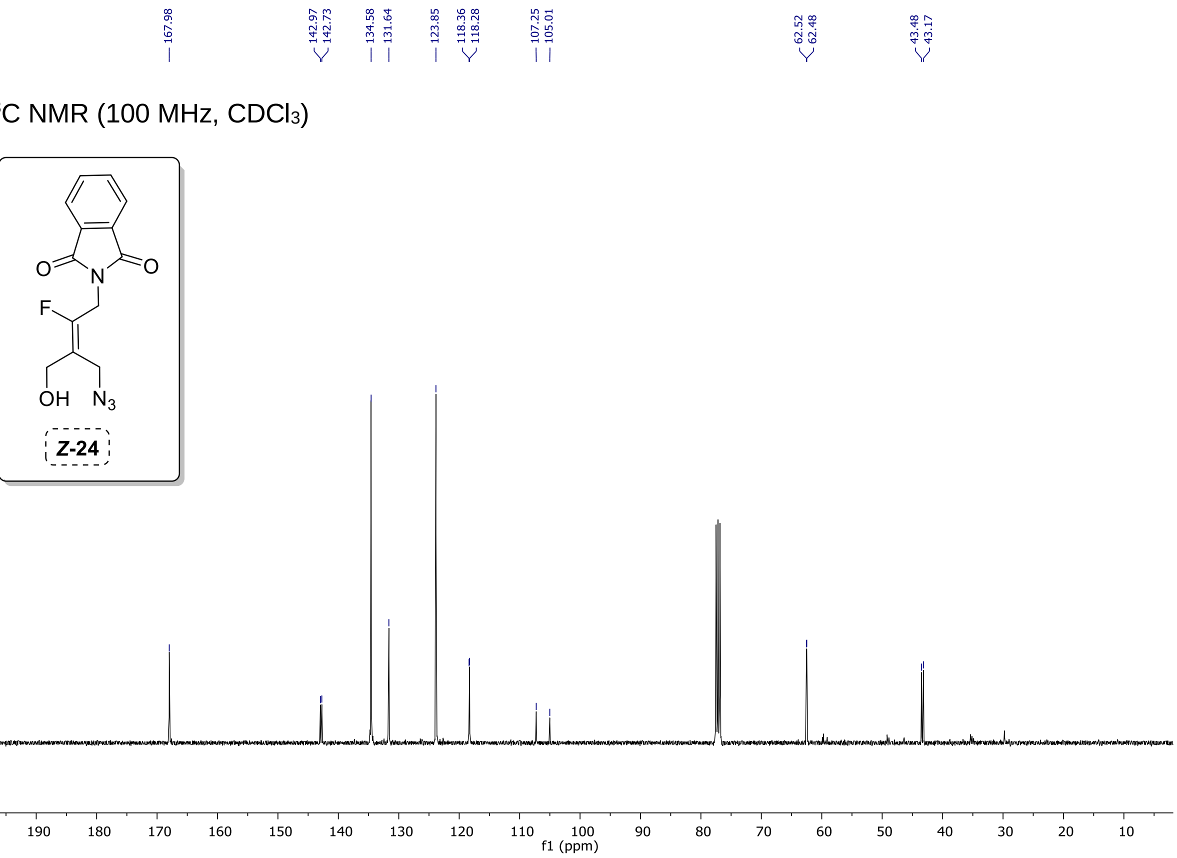

167.98
142.97
142.73
134.58
131.64
123.85
118.36
118.28
107.25
105.01
62.52
62.48
43.48
43.17
^{13}C NMR (100 MHz, $CDCl_3$)
O
N
O
F
OH
N_3
Z-24
f1 (ppm)

19F

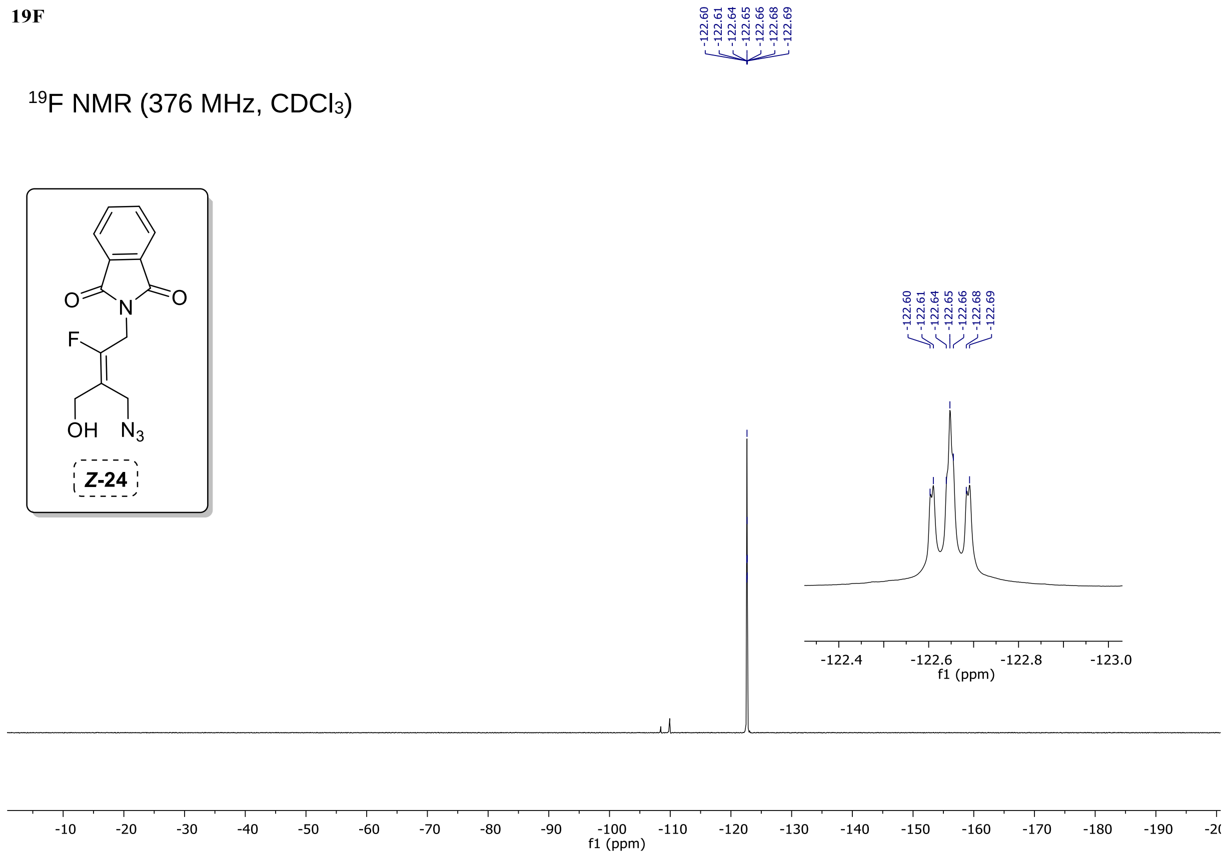

-122.60
-122.61
-122.64
-122.65
-122.66
-122.68
-122.69
^{19}F NMR (376 MHz, $CDCl_3$)
O
N
O
F
OH
N_3
Z-24
-122.4
-122.6
-122.8
-123.0
f1 (ppm)

N-[(Z)-2-Fluoro-3-(hydroxymethyl)-4-pyrrolidin-1-yl-but-2-enyl] phthalimide **(*Z*-25)**

1H

7.86 7.85 7.84 7.84 7.74 7.73 7.72 7.72 7.26 4.54 4.49 4.39 4.38 3.59 2.74 2.73 1.86 1.85 1.84

^{1}H NMR ($CDCl_3$, 400 MHz)

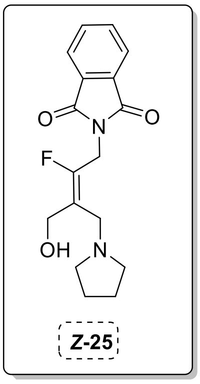

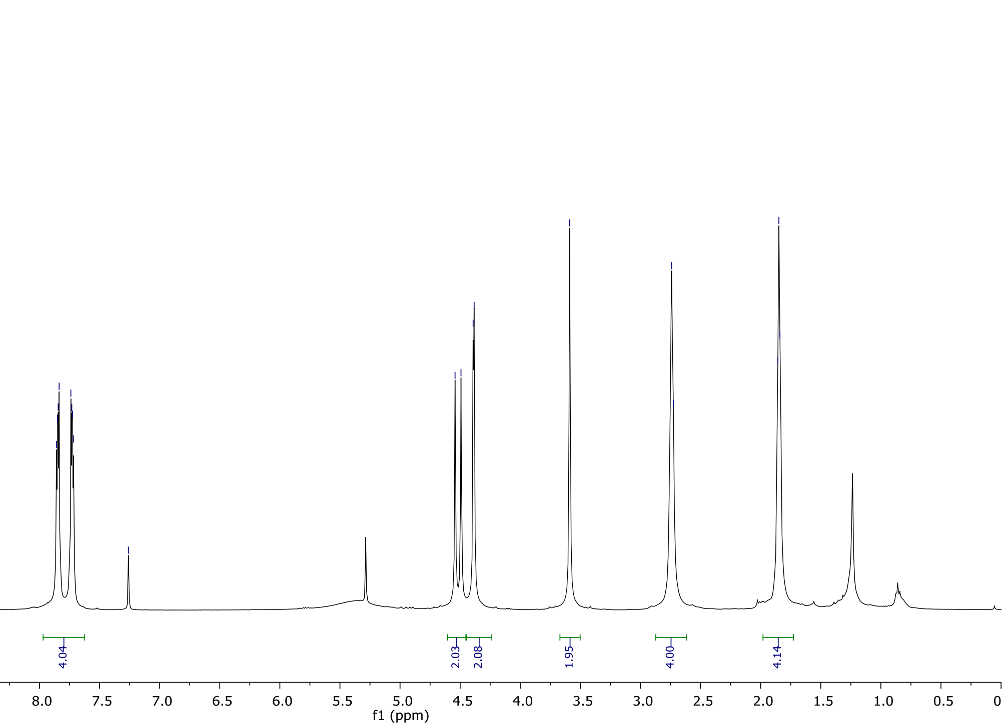

13C

167.62 151.47 148.94 134.37 131.96 123.63 117.22 117.08 77.48 77.16 76.84 59.65 59.54 54.49 54.42 54.03 34.99 34.69 23.66

^{13}C NMR (100 MHz, $CDCl_3$)

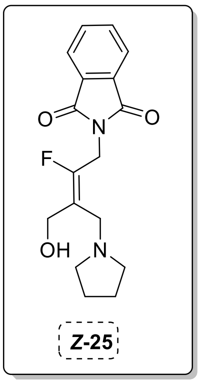

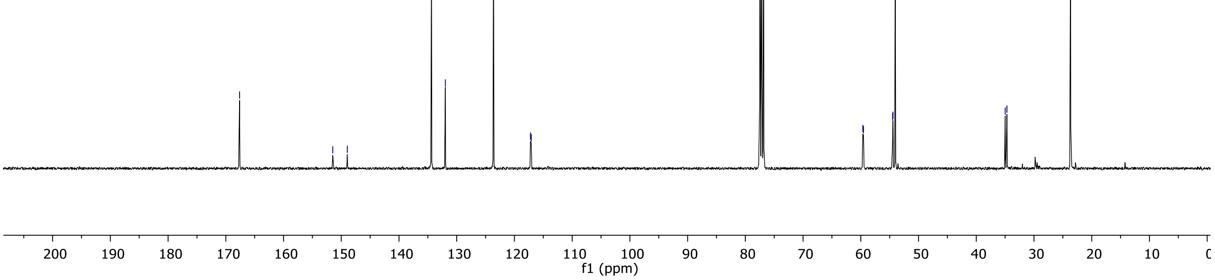

19F

—-110.64

^{19}F NMR (376 MHz, CDCl$_3$)

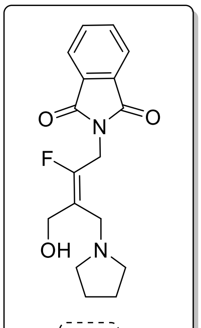

Z-**25**

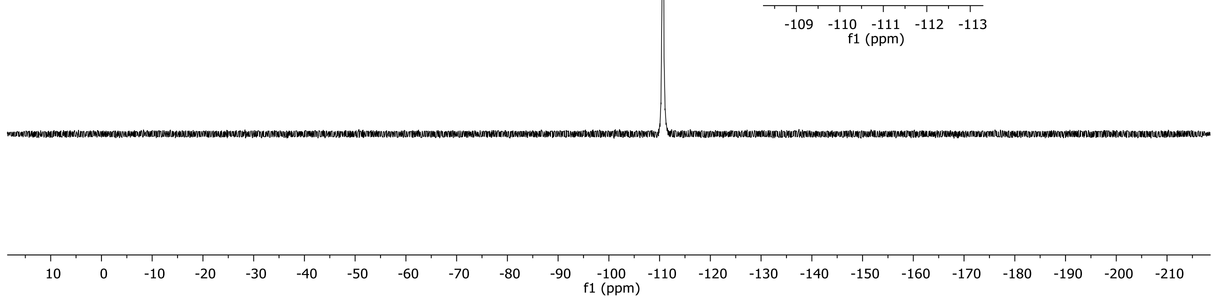

[(*E*)-4-Benzyloxy-3-fluoro-2-(hydroxymethyl)but-2-enyl] acetate **(26)**

1H

7.30 7.29 7.26 7.24 7.21 7.19 7.18 — 4.66 — 4.51 — 4.24 4.19 4.15 — 2.37 — 2.10 — 1.99

^{1}H NMR (CDCl$_3$, 500 MHz)

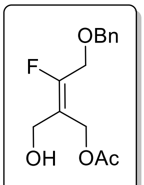

26

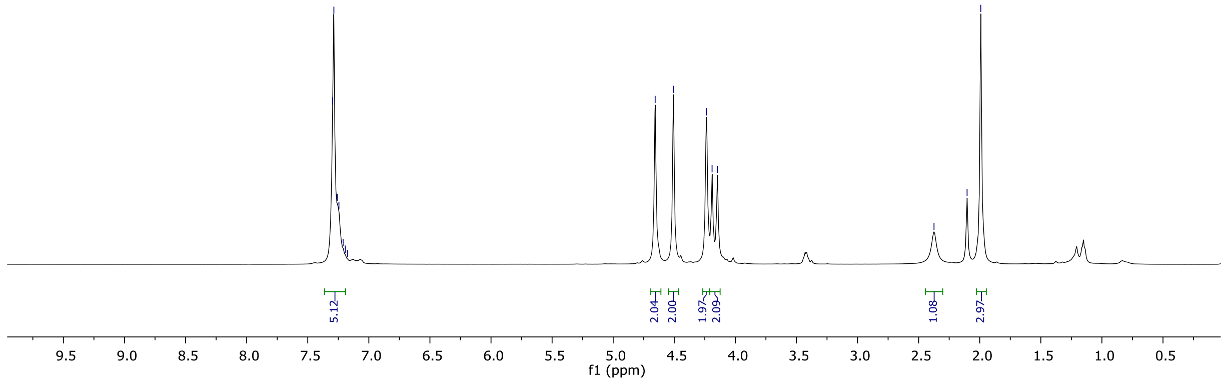

13C

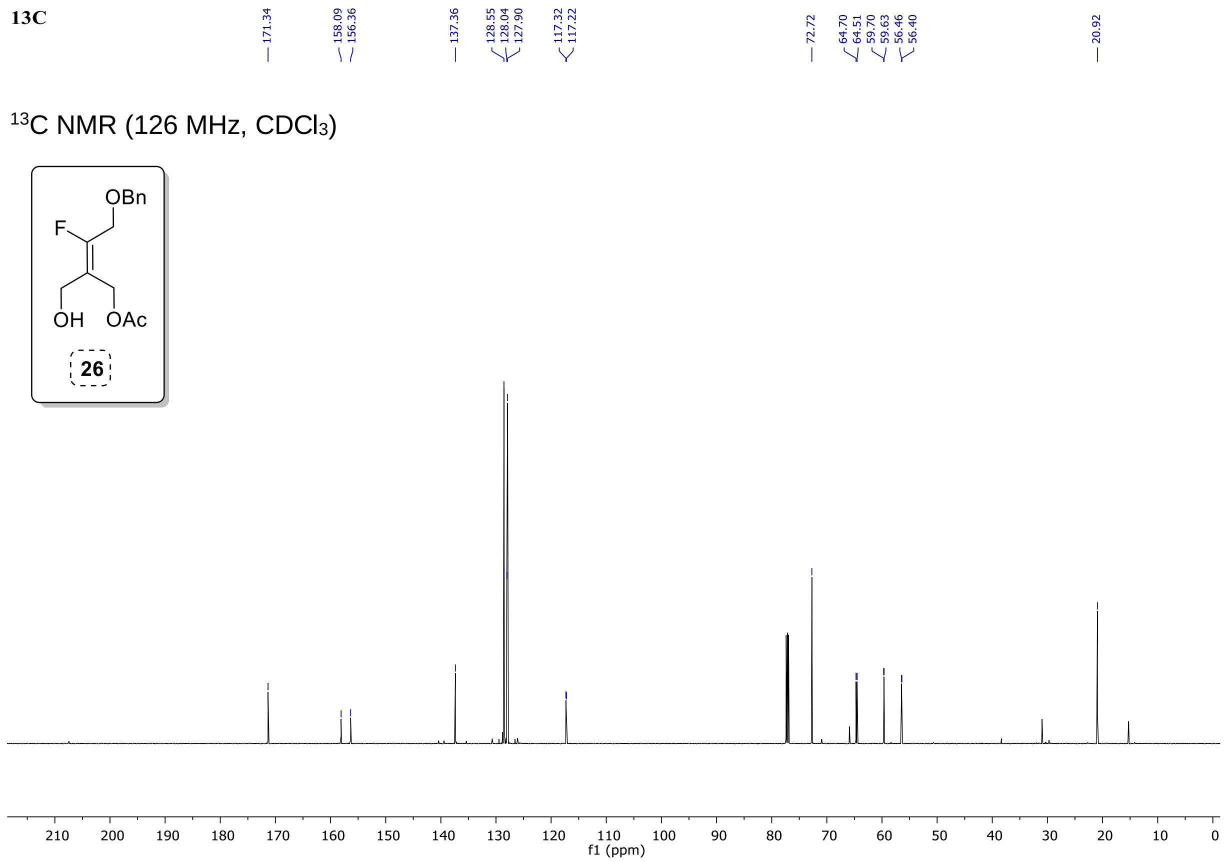
171.34
158.09
156.36
137.36
128.55
128.04
127.90
117.32
117.22
72.72
64.70
64.51
59.70
59.63
56.46
56.40
20.92
^{13}C NMR (126 MHz, CDCl$_3$)
OBn
F
OH
OAc
26
210
200
190
180
170
160
150
140
130
120
110
100
90
80
70
60
50
40
30
20
10
0
f1 (ppm)

19F

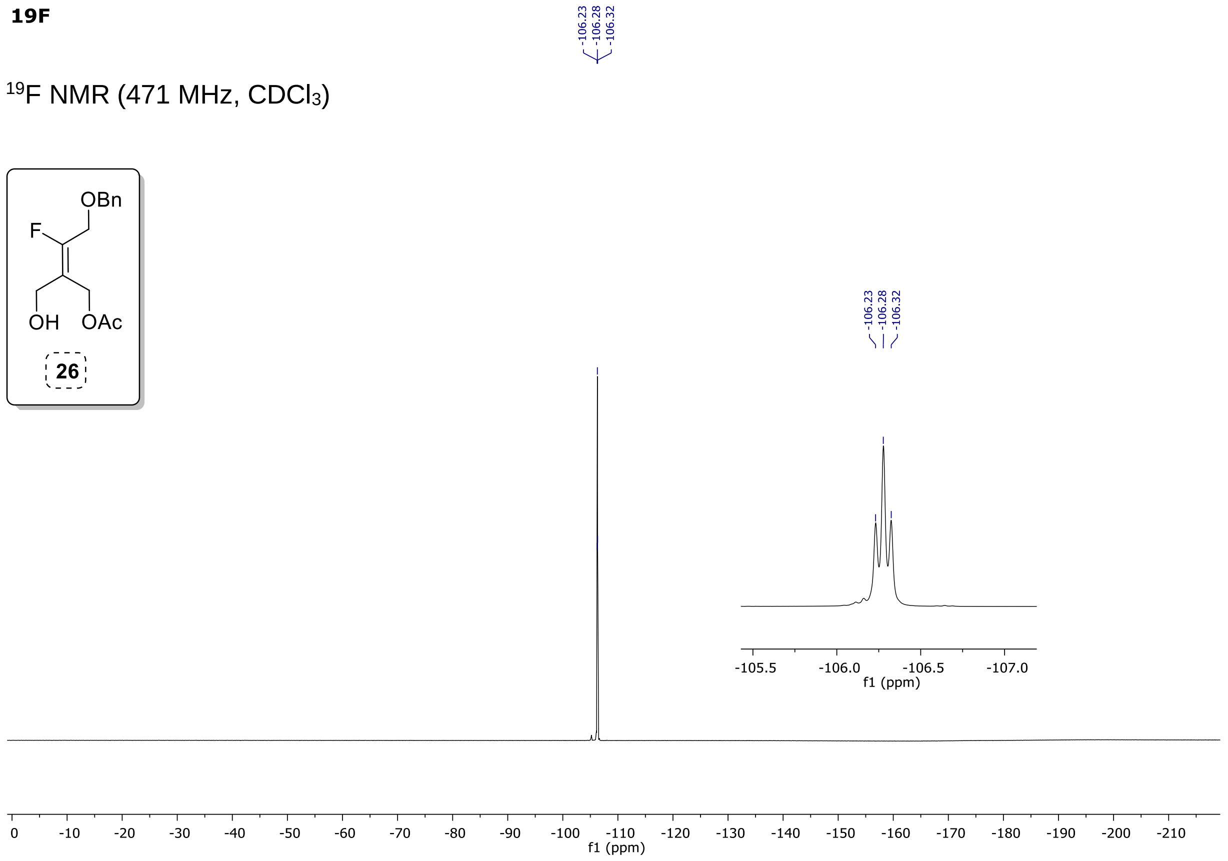
-106.23
-106.28
-106.32
^{19}F NMR (471 MHz, CDCl$_3$)
OBn
F
OH
OAc
26
-105.5
-106.0
-106.5
-107.0
f1 (ppm)
0
-10
-20
-30
-40
-50
-60
-70
-80
-90
-100
-110
-120
-130
-140
-150
-160
-170
-180
-190
-200
-210
f1 (ppm)

[(*E*)-2-(Azidomethyl)-4-benzyloxy-3-fluoro-but-2-enyl] acetate **(28)**

1H

7.38 7.38 7.38 7.37 7.37 7.36 7.35 7.35 7.34 7.34 7.33 7.33 7.32 7.32 7.31 7.30 7.30 7.29 4.67 4.66 4.58 4.30 4.26 4.00 4.00 2.06

^1H NMR ($CDCl_3$, 500 MHz)

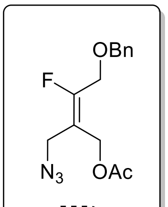

28

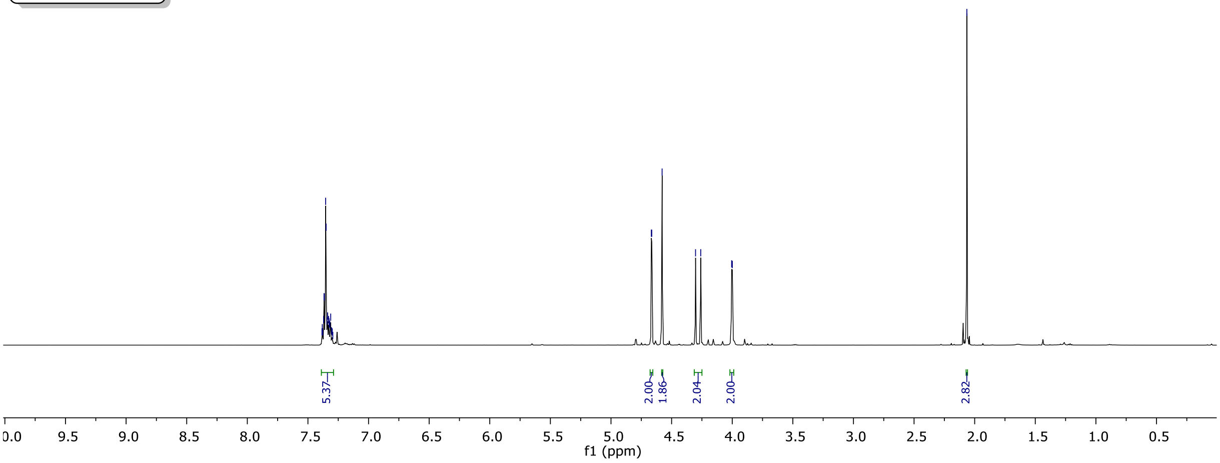

13C

170.72 160.26 158.15 137.23 128.65 128.17 128.01 112.74 112.62 72.79 64.52 64.29 60.18 60.11 46.60 46.53 20.91

^{13}C NMR (126 MHz, $CDCl_3$)

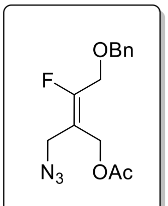

28

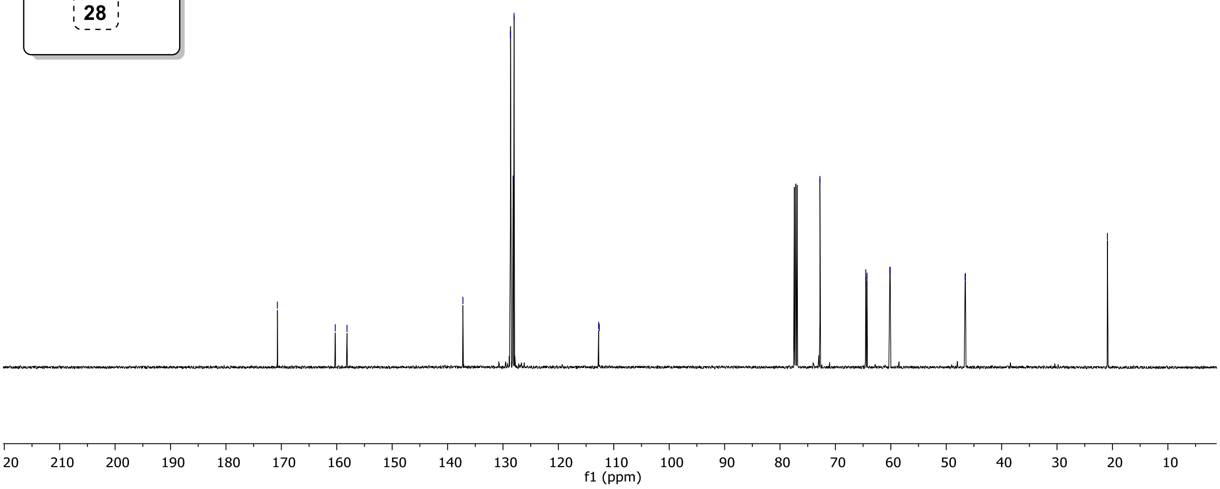

19F

^{19}F NMR (471 MHz, CDCl$_3$)

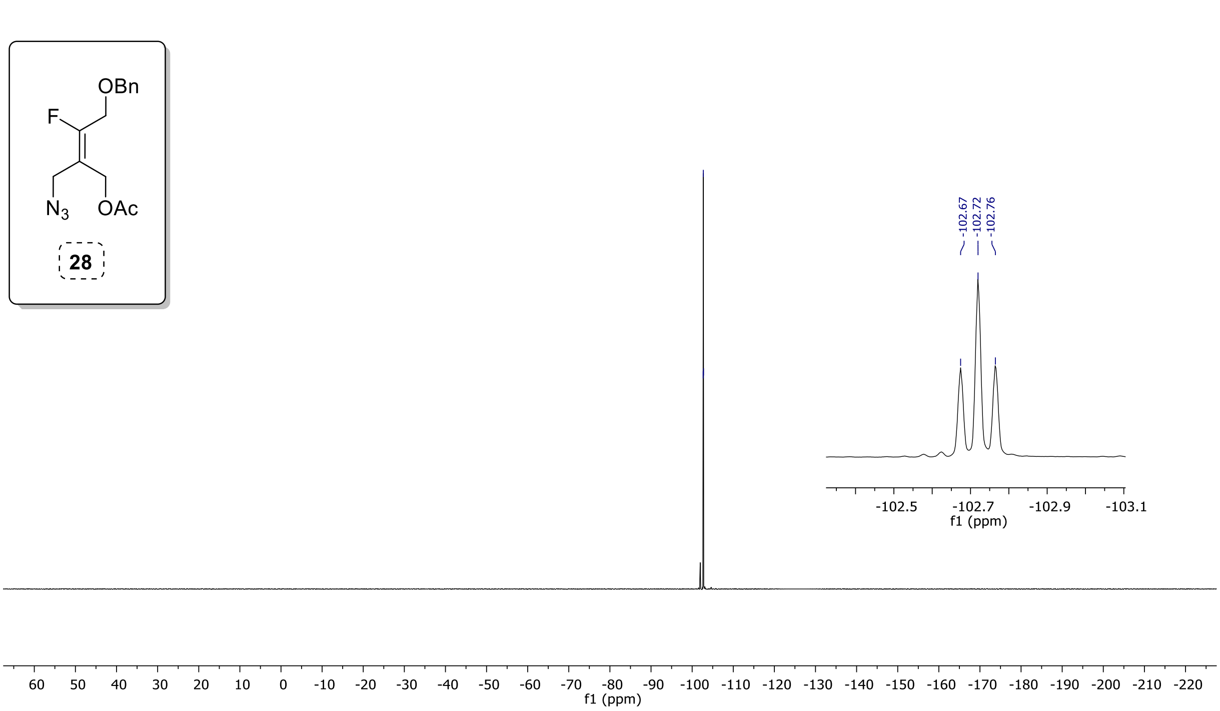

[(*Z*)-4-Benzyloxy-2-(diethoxyphosphorylmethyl)-3-fluoro-but-2-enyl] acetate **(29)**

1H

^{1}H NMR (CDCl$_3$, 500 MHz)

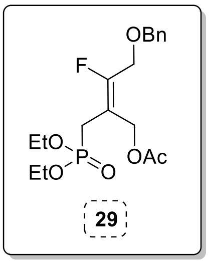

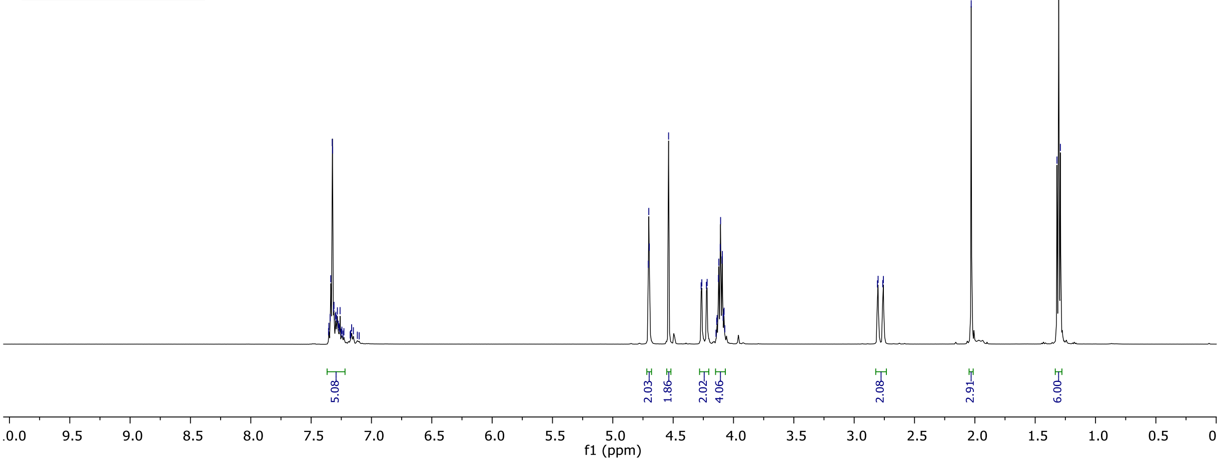

13C

^{13}C NMR (126 MHz, $CDCl_3$)

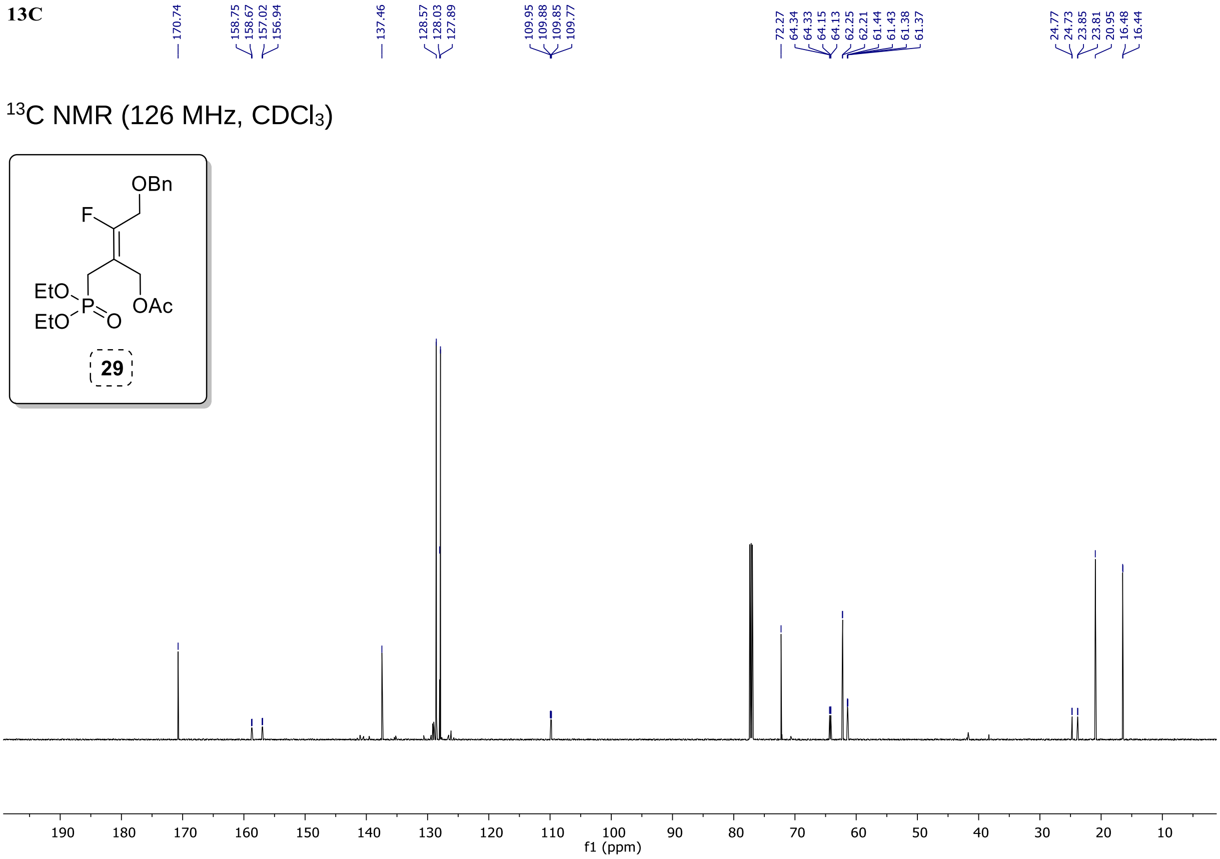

19F

^{19}F NMR (471 MHz, $CDCl_3$)

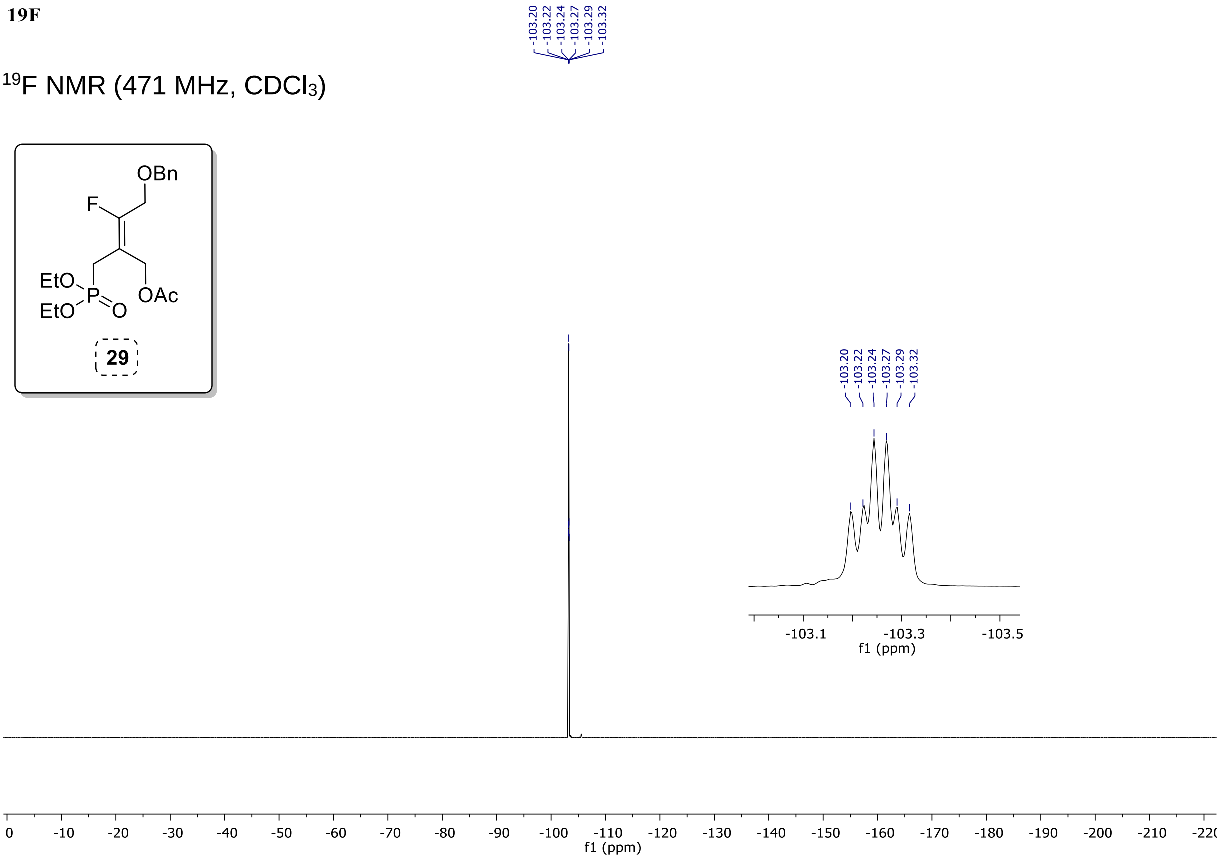

31P

^{31}P NMR (243 MHz, $CDCl_3$)

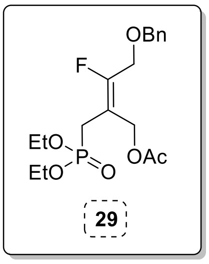

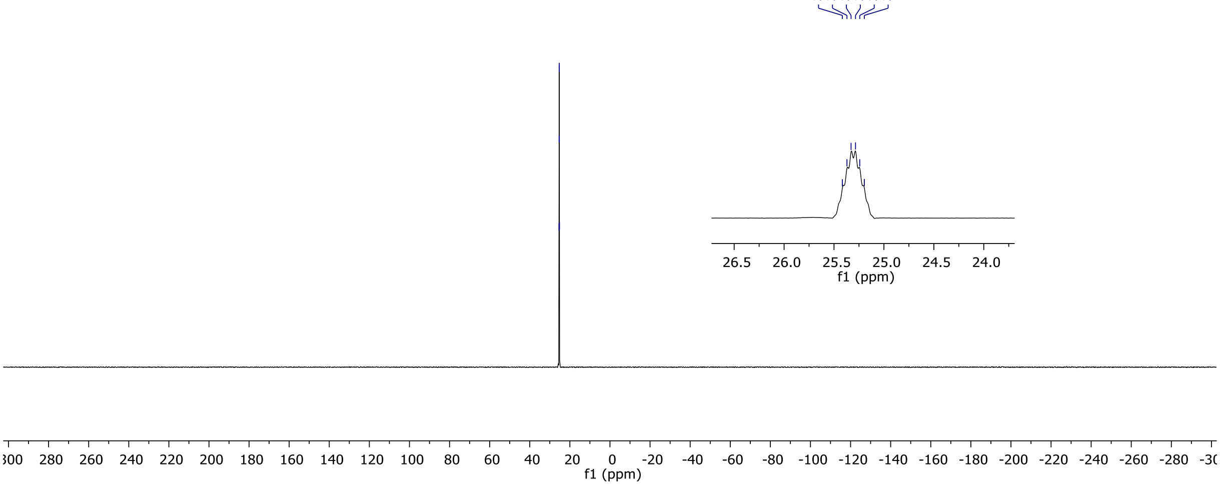

Supplement: File 1 — Experimental section and copies of spectra. [file Beilstein_J_Org_Chem-16-1936-s001.pdf]
